# Frameworks and measures for HIV‐related internalized stigma, stigma and discrimination in healthcare and in laws and policies: a systematic review

**DOI:** 10.1002/jia2.25915

**Published:** 2022-07-12

**Authors:** Laura Ferguson, Sofia Gruskin, Maria Bolshakova, Sachi Yagyu, Ning Fu, Nicky Cabrera, Mary Rozelle, Kasoka Kasoka, Tessa Oraro‐Lawrence, Lucy Stackpool‐Moore, Aneesa Motala, Susanne Hempel

**Affiliations:** ^1^ Institute on Inequalities in Global Health University of Southern California Los Angeles California USA; ^2^ Southern California Evidence Review Center Population and Public Health Sciences University of Southern California Los Angeles California USA; ^3^ School of Economics Shanghai University of Finance and Economics Shanghai China; ^4^ International AIDS Society Geneva Switzerland

**Keywords:** human rights, key and vulnerable populations, law and policy, quality of life, stigma, structural drivers

## Abstract

**Introduction:**

There is strong global commitment to eliminate HIV‐related stigma, and work in this area continues to evolve. Wide variation exists in frameworks and measures used.

**Methods:**

Building on the existing knowledge syntheses, we carried out a systematic review to identify frameworks and measures aiming to understand or assess internalized stigma, stigma and discrimination in healthcare, and in law and policy. The review addressed two questions: Which conceptual frameworks have been proposed to assess internalized stigma, stigma and discrimination experienced in healthcare settings, and stigma and discrimination entrenched in national laws and policies? Which measures of these different types of stigma and discrimination have been proposed and what are their descriptive properties? Searches, completed on 6 May 2021, cover publications from 2008 onwards. The review is registered in PROSPERO (CRD42021249348), the protocol incorporated stakeholder input, and the data are available in the Systematic Review Data Repository.

**Results and discussion:**

Sixty‐nine frameworks and 50 measures met the inclusion criteria. Critical appraisal figures and detailed evidence tables summarize these resources. We established a compendium of frameworks and a catalogue of measures of HIV‐related stigma and discrimination. Seventeen frameworks and 10 measures addressed at least two of our focus domains, with least attention to stigma and discrimination in law and policy. The lack of common definitions and variability in scope and structure of HIV‐related frameworks and measures creates challenges in understanding what is being addressed and measured, both in relation to stigma and efforts to mitigate or reduce its harmful effects. Having comparable data is essential for tracking change over time within and between interventions.

**Conclusions:**

This systematic review provides an evidence base of current understandings of HIV‐related stigma and discrimination and how further conceptual clarification and increased adaptation of existing tools might help overcome challenges across the HIV care continuum. With people living with HIV at the centre, experts from different stakeholder groups could usefully collaborate to guide a more streamlined approach for the field. This can help to achieve global targets and understand, measure and help mitigate the impact of different types of HIV‐related stigma on people's health and quality of life.

## 1 INTRODUCTION

HIV‐related stigma and discrimination constitute significant barriers to HIV responses around the world. Fragmentation of efforts to address HIV‐related stigma and discrimination has hampered progress to date. To strengthen the evidence base on HIV‐related stigma and discrimination, it is urgent to analyse the different existing conceptualizations and measures to identify lessons that can inform more effective and efficient interventions moving forward.

Stigma can be described as a dynamic process of devaluation that significantly discredits an individual in the eyes of others, such as when certain attributes are seized upon within particular cultures or settings and defined as discreditable or unworthy. Much work around HIV‐related stigma uses as its starting point Goffman's 1963 definition of stigma as “an attribute that is deeply discrediting” [1]. Socially constructed notions of difference, acceptability and fear drive evolving understandings of stigma, which now encompass notions of power and incorporate social psychological and socio‐cultural approaches [2, 3, 4]. Yet, the diversity of co‐existing definitions is important: it has spawned a multitude of conceptual frameworks around stigma and a lack of consensus on key aspects of what stigma actually is and how to measure it.

“Stigma” is often used in the literature to encompass both stigma and discrimination even as these are conceptually distinct. While stigma usually refers to an attitude or belief, discrimination is often seen as the behaviour or action that results from those attitudes or beliefs. Hence, when stigma is acted upon, the result can be discrimination. Discrimination may refer to any form of arbitrary distinction, exclusion or restriction affecting a person, usually (but not only) because of an inherent personal characteristic or perceived membership of a particular group [5].

Narrowing down to HIV‐related stigma, this has been defined by the Joint United Nations Programme on HIV/AIDS (UNAIDS) as negative beliefs, feelings and attitudes towards people living with HIV, groups associated with people living with HIV (e.g. their families) and other key populations at higher risk of HIV infection, such as people who use drugs, sex workers, men who have sex with men and transgender people [6]. Different domains have been identified in attempts to categorize HIV‐related stigma, including internalized, anticipated, perceived, enacted, externalized and structural stigma [7]. However, there is no universally agreed‐upon list of types of HIV‐related stigma and how each is defined. Working definitions adopted for this review are described in the analytical framework below.

The lack of consensus about HIV‐related stigma domains creates challenges for consistent and comparable frameworks and measures for understanding them. Given that stigma is highly contextualized, including across these different domains, it is important that frameworks and measures be appropriate to local contexts, further complicating efforts to create comparable tools.

Discrimination, as defined under international human rights law, is any distinction, exclusion or restriction based indirectly or directly on grounds prohibited under international law, which has the effect or intent of nullifying the recognition, enjoyment or exercise on an equal basis of others of all human rights and fundamental freedoms, in the political, economic, social, cultural, civil or any other field [8]. HIV‐related discrimination is, therefore, any distinction, exclusion or restriction (sometimes referred to as acts or omissions) based indirectly or directly on a person's real or perceived HIV status [9].

There is strong commitment to eliminate HIV‐related stigma, starting with global political commitments and reflected in global and national strategies as well as many organizations and collaborations working to address stigma [10]. Yet, learning across interventions designed to mitigate against the experience and harmful impacts of stigma can be hindered, in part, by the multitude of frameworks and measures in use to assess its different dimensions.

Experiences of stigma for people living with and most affected by HIV can occur at many levels. This review systematically identifies and assesses frameworks and measures of HIV‐related internalized stigma; stigma and discrimination within healthcare settings; and in laws and policies. These focus areas were selected as each requires a very different response, suggesting that, even as one might expect strong similarities within each of these domains, there might be substantial heterogeneity in frameworks and measures across them. Recognizing that to reduce stigma at scale, synergistic attention is required across all three domains, this is the first systematic review to look across them systematically.

The co‐existence and potential interrelationship between HIV‐related stigma and other devaluing attitudes related to drug use, sex work, sexual orientation and/or gender identity that affect populations disproportionately affected by HIV is critical, but beyond the scope of this review. The review focuses on conceptual frameworks and measures of HIV‐related stigma itself, acknowledging as possible where additional types of stigma are addressed.

The systematic review was guided by two key questions:

1. Which conceptual frameworks have been proposed to assess internal stigma, stigma and discrimination experienced in healthcare settings, and stigma and discrimination entrenched in national laws and policies?
2. Which measures (e.g. assessment scales) of these different types of stigma and discrimination have been proposed and what are their descriptive properties?

## 2 METHODS

The systematic review followed a detailed protocol (CRD42021249348) [11]. Part of a larger project undertaken by the IAS—International AIDS Society, this systematic review is accompanied by four national efforts exploring stigma and discrimination in Kenya, Malawi, South Africa and Zambia.

We searched multiple disciplinary and interdisciplinary sources. Citations and full‐text publications were screened by independent literature reviewers, and eligibility decisions, including reasons for exclusions, were tracked in citation management software. Data abstraction and critical appraisal was conducted in online software designed for systematic reviews using detailed, pilot‐tested forms. Given the complexity of the frameworks and measures, data were abstracted by one reviewer and checked by a second experienced systematic reviewer. The collected data are accessible in a review data repository [12].

### 2.1 Analytic framework

Given the diversity of definitions in this interdisciplinary field, we established working definitions of the concepts of HIV‐related “stigma,” “internalized stigma” and “discrimination” grounded in existing literature for the purpose of this systematic review:

- Stigma refers to beliefs and/or attitudes about HIV.
- Internalized stigma refers to a person living with HIV internalizing negative attitudes associated with HIV and accepting these as applicable to themselves.
- Discrimination refers to the behaviours that result from attitudes or beliefs about HIV.
- Stigma and discrimination in healthcare refers to negative beliefs and behaviours based on perceived or actual HIV status experienced in healthcare delivery settings.
- Stigma and discrimination in laws and policies refers to distinctions, exclusion or restriction based on perceived HIV status or membership of a group that is vulnerable to HIV.

### 2.2 Search strategy

To identify primary research studies, we searched PubMed, in particular to identify research on stigma experienced in healthcare settings, PsycINFO to identify psychological and social research on stigma, and the Web of Science to identify legal and policy analyses on stigma and discrimination. We identified government and non‐governmental organization reports indexed in the Universal Human Rights Index, HeinOnline, Public Affairs Information Service (PAIS) and HIV Legal Network.

Additional grey literature searches targeted the websites of the IAS, UNAIDS, United Nations Development Programme, STRIVE (research consortium investigating the social norms and inequalities driving HIV acquisition), Health Policy Plus and Sage (resource‐sharing community for Canadian HIV and hepatitis C service providers).

Systematic reviews were instrumental for reference‐mining to ensure that all relevant material had been considered. Systematic reviews were identified through PubMed (biomedical literature) using the systematic review filter, through PsycINFO (psychosocial literature) and Web of Science (general science literature, including legal and policy analysis), as well as through the Cochrane Database of Systematic Reviews (focus on health) and the Campbell Collaboration (focus on social sciences). Furthermore, we searched the review registries PROSPERO and Open Science Framework to ensure that all relevant registered systematic reviews had been identified.

### 2.3 Eligibility criteria

Detailed eligibility criteria are documented in the online Appendix. Briefly, publications addressing people living with or perceived to be living with HIV and people from groups who are disproportionately affected by HIV infection were eligible. Frameworks and measures had to address HIV‐related internalized stigma, stigma and discrimination in healthcare or in laws and policies. Publications introducing frameworks were included regardless of the comparator or study design. Measure research had to describe the tool in sufficient detail to be included but needed no comparator. Framework publications were included regardless of any reported outcomes. Measure research had to report a description of the measure, the development process, or the evaluation or validation of the measure. Only publications from 2008 on were included, building on the first People Living with HIV Stigma Index published in 2008, which transformed thinking around HIV‐related stigma measurement, fostering new levels of openness, nuance and confidence in stigma measures [13]. To maintain consistency in approaches to reviewing measures and frameworks, the same cut‐off date was used for searches for frameworks. Searches were completed on 6 May 2021. The review was not restricted by setting but we restricted to English language for both frameworks and measures. Measures designed for other languages were included if the publication also presented an English translation.

### 2.4 Data abstraction

For the frameworks, we abstracted the author group; publication year, scope, aim or purpose of the framework; terminology, domain of interest targeted, type, all stigma subtypes as reported by the authors, definition of the constructs stigma and/or discrimination; addressed targets; framework components; and a broad summary of the framework based on the authors’ description.

For measures, we documented the author group; publication year; name of the tool; the stigma or discrimination subtype, the underlying framework, and definitions of stigma and discrimination; the targeted population; the surveyed population used to develop or assess the measure; the scale structure of the tool, number of items and answer mode; the documented reliability; and evidence of validity.

### 2.5 Critical appraisal

**Figure 1:**
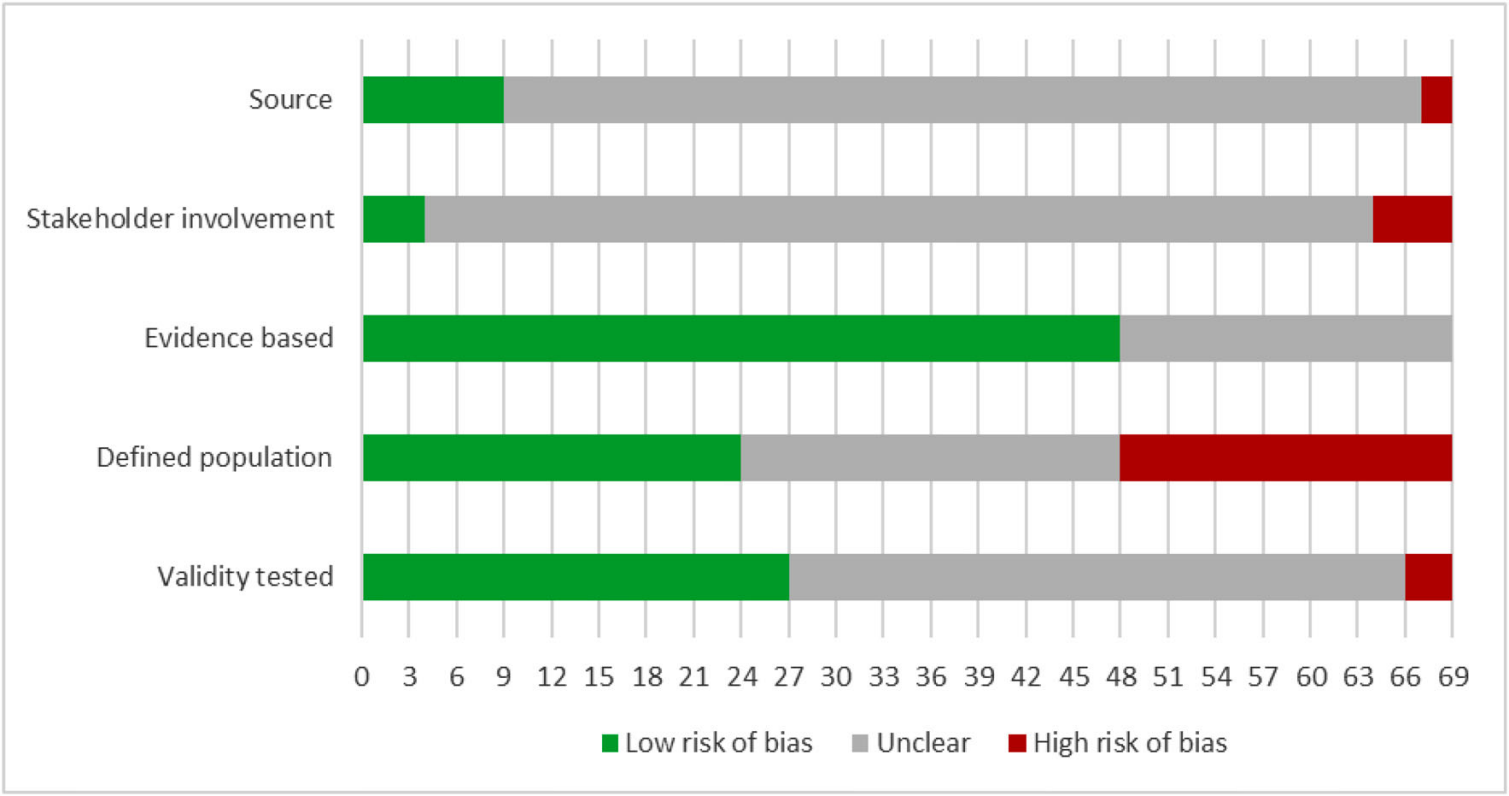
Critical appraisal summary: frameworks (*n* = 69). Source: Assesses whether the framework was published or endorsed by a relevant organization; Stakeholder involvement: Assesses whether the framework was established with relevant stakeholder input; Evidence based: Assesses whether the components of the frameworks were based on a systematic literature review or empirical data; Defined population: Assesses whether the population the framework is designed to address is clearly reported; Validity tested: Assesses whether the validity of the framework was assessed (e.g., goodness of fit to empirical data assessed, framework applied in different contexts). Low risk of bias: The potential source of bias is unlikely to distort the methodological quality of the measure; Unclear: There was insufficient detail reported to assess the potential source of bias; High risk of bias: There was evidence of bias.

**Figure 2:**
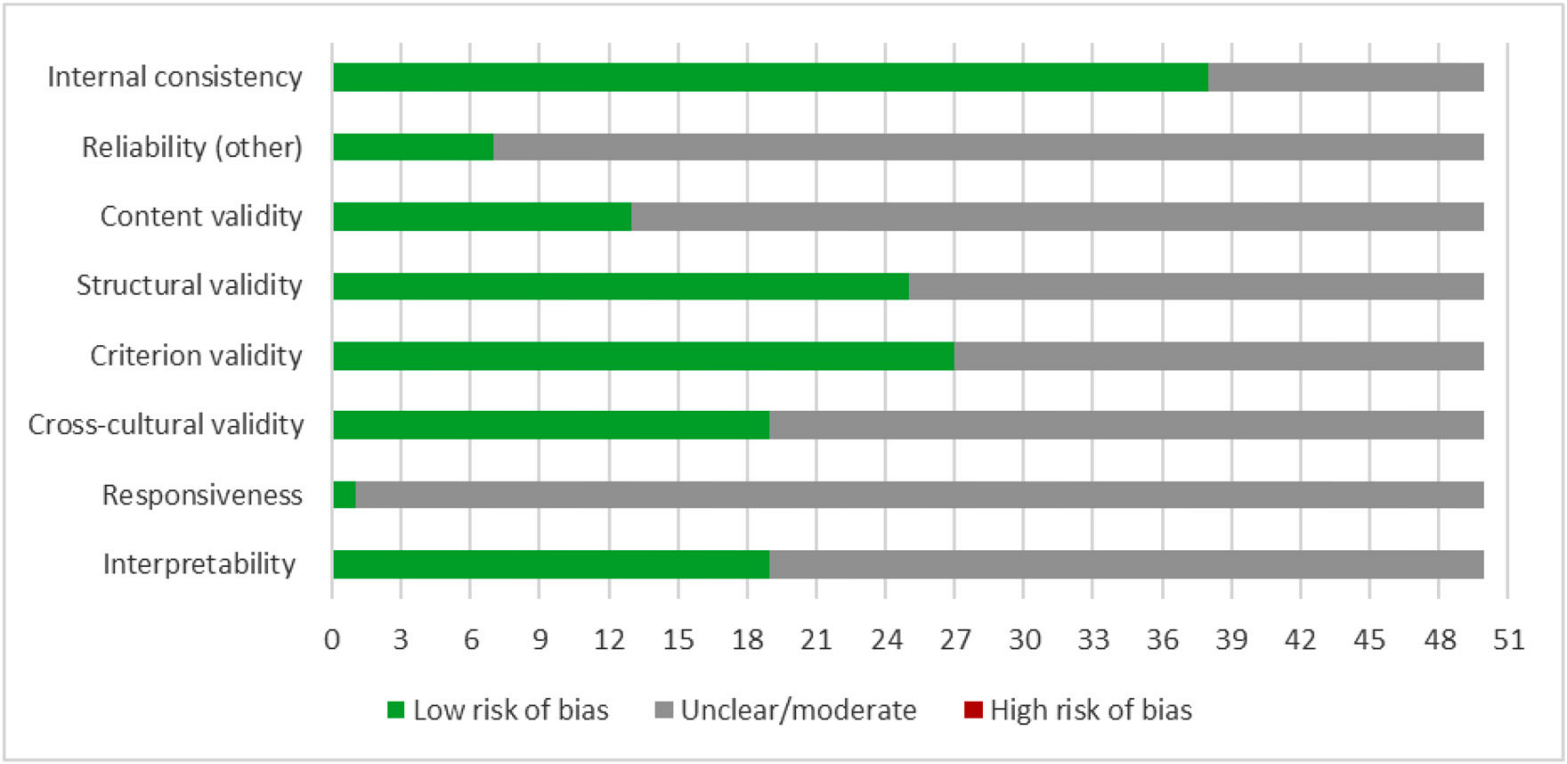
Critical appraisal: summary measures (*n* = 50). Internal consistency: Assesses whether the internal consistency of the scale was reported and it was acceptable (e.g., Cronbach's alpha >0/70); Reliability (other): Assesses whether other measures of reliability were reported and results were acceptable (e.g., test‐retest reliability, rater agreement); Content validity: Assesses whether the content of the measure was assessed for validity and the results were acceptable (e.g., face validity rated, expert review); Structural validity: Assesses whether the structural validity of the measure was assessed and the results were acceptable (e.g., through factor analysis); Criterion validity: Assesses whether convergent or discriminant validity to external criteria or other measures was determined and the results were acceptable; Cross‐cultural validity: Assesses whether measures were taken to ensure cross‐cultural validity (e.g., translation and back‐translation of items; measure exists in multiple languages or was used in multiple geographic settings); Responsiveness: Assesses whether the measure demonstrated sensitivity to change (e.g., scores changed after an intervention as predicted); Interpretability: Assesses whether guidance is reported on the interpretation of scores (e.g., minimal clinical difference). Low risk of bias: The potential source of bias is unlikely to distort the methodological quality of the measure; Unclear: There was insufficient detail reported to assess the potential source of bias; High risk of bias: There was evidence of bias.

For frameworks, we assessed the source (e.g. published by an individual author group or endorsement by a professional organization), stakeholder involvement (in the development of the framework), evidence base (components based on a systematic literature review or empirical data), defined population (framework target reported) and validity tested (e.g. goodness‐of‐fit assessed, applied in different contexts). For measures, we evaluated the demonstrated internal consistency, other reliability measures (temporal stability, rater agreement), content validity, structural validity, criterion validity, cross‐cultural validity, responsiveness and interpretability by applying relevant COSMIN (COnsensus‐based Standards for the selection of health Measurement Instruments) criteria [14]. Scoring information is provided in Figures 1 and 2.

### 2.6 Synthesis

Recognizing that multiple types of stigma and discrimination may be concurrently experienced, our narrative synthesis focuses on frameworks and measures that address more than one of our three focus domains: internalized stigma, stigma and discrimination in healthcare, and in laws and policies. This can help us move towards a more complete understanding of different types of stigma and discrimination, and inform complex interventions moving forward.

## 3 RESULTS AND DISCUSSION

The evidence review identified 69 frameworks and 50 measures. The initial searches identified 2199 citations, 1050 were obtained as full text. In total, 146 publications reported information on the included frameworks and measures. The literature flow is documented in the online Appendix (Figure S1).

### 3.1 Frameworks

To address key question 1, the evidence table in the online Appendix (Table S1) provides a concise overview of the identified frameworks. The online Appendix also provides a compendium of the included frameworks to allow a meaningful overview. The evidence table provides a weblink to the original publication for all included frameworks.

Figure 1 summarizes the critical appraisal of the identified frameworks.

As the figure shows, for many domains, studies provided insufficient information or did not meet the prespecified criteria. Just over 10% of the identified frameworks were published by a well‐known HIV‐specific source, such as UNAIDS. Identified frameworks were usually developed to provide an analytic framework for a specific research question and purpose, such as the evaluation of an association. Only 6% of the frameworks reported stakeholder input into their development, with different “stakeholders” included, such as people living with HIV, health workers and administrators. Studies either did not report on the development process and any consensus finding results, or the model appeared to be derived from empirical data without in‐depth conceptual considerations. Two thirds of the framework authors reported a literature review or referenced empirical literature to justify the framework or its components. A third of frameworks explicitly stated the population addressed. Many identified frameworks were broad and provided only minimal details on their scope. Finally, 39% of the frameworks reported a validity evaluation, by, for example, reporting on the model's goodness‐of‐fit to empirical data. Table S2 shows the number of criteria met for each identified framework.

Table 1 summarizes the 17 frameworks that address more than one stigma domain, for example both internalized stigma as well as stigma and discrimination in healthcare. The table includes information on each framework's scope, aim/purpose, other subtypes of stigma covered and a summary of the framework from the original authors’ description.

**Table 1:** Frameworks addressing multiple stigma domains (*n* = 17)

ID Framework name Domain Terminology Empirical/categorical	Scope and aim/purpose	Stigma subtype	Summary
**Frameworks addressing internalized stigma, stigma and discrimination in healthcare and law**			
Gilbert [15] Framework name: NA Domain: Internalized stigma, stigma or discrimination in healthcare settings, stigma or discrimination in law Terminology: Stigma Conceptual model	Scope: HIV‐related stigma evidenced in South Africa Aim/purpose: To examine individual and social/structural components of HIV‐related stigma in South Africa	Subtype: Internalized, enacted discrimination, by association, instrumental, symbolic	To provide a comprehensive framework that offers insights into the individual as well as the social/structural components of HIV‐related stigma in a particular context.
Pescosolido and Martin [16] Framework name: Framework Integrating Normative Influences on Stigma (FINIS) Domain: Internalized stigma, stigma or discrimination in healthcare settings, stigma or discrimination in law Terminology: Stigma, discrimination Empirical model	Scope: A multilevel approach that can be tailored to stigmatized statuses Aim/purpose: To provide a theoretical architecture of concepts, proposing a stigma complex, a system of interrelated, heterogeneous parts bringing together insights across disciplines to provide a more realistic and complicated sense of the challenge facing research and change efforts	Subtype: Perceived, endorsed, anticipated, received, enacted, self‐stigma, courtesy stigma, public stigma, prover‐based stigma, structural stigma	In essence, the FINIS is a systems science approach. The rationale for FINIS lies in evidence, reviewed here, that stigma emanates from many societal and individual systems whose interconnections cannot be divorced from one another. They coexist in a dynamic relationship in which there is an interplay across, for example, the media, the community and the individual.
Stangl et al. [17] Stangl et al. [18]; ICRW [19]; Stangl and Barre [20]; Stangl et al. [21] Framework name: The Health Stigma and Discrimination Framework Domain: Internalized stigma, stigma or discrimination in healthcare settings, stigma or discrimination in law Terminology: Stigma, discrimination Conceptual model	Scope: Health‐related stigmas Aim/purpose: To amplify our collective ability to respond effectively and at‐scale to a major driver of poor health outcomes globally	Subtype: Internalized, perceived stigma, associated stigma, experienced discrimination	The Health Stigma and Discrimination Framework is a global, crosscutting framework based on theory, research and practice, which demonstrates its application to a range of health conditions, and discusses how stigma related to race, gender, sexual orientation, class and occupation intersects with health‐related stigmas, and how the framework can be used to enhance research, programming and policy efforts.
Stevens et al. [22] Framework name: Rehabilitation framework Domain: Internalized stigma, stigma or discrimination in healthcare settings, stigma or discrimination in law Terminology: Stigma Conceptual model	Scope: Conceptualizing HIV in a rehabilitation framework Aim/purpose: To help rehabilitation professionals better understand the dynamic and nuanced forms of stigma and how they relate to rehabilitation	Subtype: Enacted, self and structural stigma	Three broad spheres of stigma are described: enacted, self and structural stigma. These three forms of stigma are then aligned in unique ways with three particular constructs of the International Classification of Functioning, Disability and Health: participation restrictions, environmental and personal contextual factors.
Thapa et al. [23] Framework name: NA Domain: Internalized stigma, stigma or discrimination in healthcare settings, stigma or discrimination in law Terminology: Stigma Empirical model	Scope: Effect of stigma‐reduction intervention strategies on HIV test uptake Aim/purpose: A conceptual framework to illustrate mechanisms involved in reducing HIV stigma and increasing HIV test uptake	Subtype: NA	A conceptual framework to illustrate the mechanisms of the effect of stigma‐reduction intervention strategies on HIV test uptake.
UNAIDS [24] Framework name: Effects of stigma and discrimination on health care access for prevention, testing and treatment Domain: Internalized stigma, stigma or discrimination in healthcare settings, stigma or discrimination in law Terminology: Stigma, discrimination Conceptual model	Scope: Stigma and discrimination as healthcare service barriers Aim/purpose: NA	Subtype: NA	Criminal laws, community attitudes, misinformation, prejudice and fear are all drivers of stigma and actual manifestations of discrimination affecting access to healthcare by people living with HIV and key populations.
UNAIDS [24] Framework name: Removing stigma and discrimination improves health care access for prevention, testing and treatment Domain: Internalized stigma, stigma or discrimination in healthcare settings, stigma or discrimination in law Terminology: Stigma, discrimination Conceptual model	Scope: Stigma and discrimination in healthcare access for prevention, testing and treatment. Confronting stigma to remove healthcare stigma barriers Aim/purpose: NA	Subtype: NA	By addressing drivers, removal of harmful laws, introduction of protective laws, education of rights holders and service providers and legal empowerment of communities to defend their rights, the gap between those who can access services and those who cannot can be closed, leading to better health for all.
Woodgate et al. [25] Framework name: Social ecological framework Domain: Internalized stigma, stigma or discrimination in healthcare settings, stigma or discrimination in law Terminology: Stigma, discrimination Conceptual model	Scope: Stigma and discrimination on the lives of Indigenous people who contracted HIV in their youth (i.e. 15–29 years of age) who are HIV positive within their various settings in Manitoba, Canada Aim/purpose: Developing a better structural understanding of the impacts of stigma and discrimination on the lives of Indigenous people who are HIV positive	Subtype: NA	Stigma and discrimination caused barriers for Indigenous people living with HIV through inhibiting their ease of access to supports, including family, peers, community and long‐ and short‐term health services.
**Frameworks addressing internalized stigma, stigma and discrimination in healthcare**			
Darlington and Hutson [26] Framework name: Current state‐of‐science of HIV‐related stigma among HIV + women in the Southern US Domain: Internalized stigma, stigma or discrimination in healthcare settings Terminology: Stigma Empirical model	Scope: HIV‐related stigma among women living in the South Aim/purpose: Understanding HIV‐related stigma among women in the Southern United States	Subtype: Perceived, experienced, internalized	A description of the current state‐of‐science of HIV‐related stigma among HIV and women in the Southern United States
Earnshaw et al. [27] Earnshaw and Chaudoir [28]; Misir [29]; Goodin et al. [30]; Reinius et al. [31] Framework name: Hypothesized associations between HIV stigma mechanisms and health and well‐being among people living with HIV Domain: Internalized stigma, stigma or discrimination in healthcare settings Terminology: Stigma Empirical model	Scope: HIV stigma mechanism and health and wellbeing Aim/purpose: To test the HIV stigma framework evaluating HIV stigma mechanisms and wellbeing	Subtype: Internalized, anticipated, enacted	Internalized stigma associates significantly with indicators of affective (i.e. helplessness regarding, acceptance of and perceived benefits of HIV) and behavioral (i.e. days in medical care gaps and ARV nonadherence) health and wellbeing. Enacted and anticipated stigma associate with indicators of physical health and wellbeing (i.e. CD4 count less than 200 and chronic illness comorbidity, respectively).
Logie et al. [32] Framework name: NA Domain: Internalized stigma, stigma or discrimination in healthcare settings Terminology: Stigma, discrimination Empirical model	Scope: Women living with HIV in Canada Aim/purpose: To integrate concepts from multiple frameworks and test pathways from intersectional stigma to HIV‐related health outcomes via interpersonal, psychological, mental health and stress mechanisms among women living with HIV in Canada	Subtype: Intersectional	Integrating concepts from multiple frameworks to examine pathways from intersectional stigma to mental health via interpersonal and institutional support, and from mental health to care engagement and HIV‐related health via coping strategies among women living with HIV.
Sen et al. [33] Framework name: Bronfenbrenner's ecological systems theory Domain: Internalized stigma, stigma or discrimination in healthcare settings Terminology: Stigma Conceptual model	Scope: Asian American and Pacific Islander men who have sex with men in the United States Aim/purpose: To explore the manifestation of HIV stigma at the micro, meso and macro levels and how these might impact on HIV testing and HIV service utilization	Subtype: Multilevel ecological framework of stigma	A model which is culturally grounded and bridges the individual, interpersonal and societal conceptualizations of stigma.
Thi et al. [34] Framework name: NA Domain: Internalized stigma, stigma or discrimination in healthcare settings Terminology: Stigma, discrimination Empirical model	Scope: Causes of HIV stigma and discrimination in Vietnam Aim/purpose: To provide a schematic diagram of stigma and discrimination against people living with HIV addressing causes, effects and relationships	Subtype: Internalized	Three main themes relating to stigma and discrimination emerged: (1) attitudes, misperceptions and negative media representations led to the stigmatization of people living with HIV; (2) acts of discrimination occurred within various sectors of Vietnamese society, including the family, community, healthcare sector and workplace; and (3) stigma and discrimination resulted in negative effects on people living with HIV.
Wardell et al. [35] Framework name: NA Domain: Internalized stigma, stigma or discrimination in healthcare settings Terminology: Stigma Empirical model	Scope: Associations among HIV‐related stigma, coping and problem drinking Aim/purpose: Examining prospective bidirectional and mediated associations among HIV‐related stigma, maladaptive coping and alcohol use severity in patients enrolled in the Ontario HIV Treatment Network Cohort study	Subtype: Enacted, internalized	Cross‐lagged panel model of the prospective associations among HIV‐related stigma, maladaptive coping strategies and alcohol use severity.
Watt et al. [36] Framework name: NA Domain: Internalized stigma, stigma or discrimination in healthcare settings Terminology: Stigma Conceptual model	Scope: Women and their partners attending a first antenatal care appointment in Tanzania Aim/purpose: Stigma visual used to discuss how issues of internalized, anticipated and enacted stigma might relate to their situation of living with HIV, and to try and reduce these as barriers of HIV care engagement	Subtype: Internalized, anticipated, enacted	Addressing HIV stigma at the first antenatal care visit can help individuals living with HIV to overcome stigma‐related barriers to the initiation and maintenance of HIV care, and can reduce stigmatizing attitudes among those who test negative for HIV.
**Frameworks addressing internalized stigma and stigma and discrimination in law**			
Turan et al. [37] Framework name: Conceptual framework for HIV‐related stigma, engagement in care, and health outcomes Domain: Internalized stigma, stigma or discrimination in law Terminology: Stigma Conceptual model	Scope: Adherence to treatment, health outcomes Aim/purpose: A conceptual framework for individual‐level dimensions of stigma and potential individual and interpersonal mechanisms explaining how stigma affects HIV‐related health	Subtype: Perceived community stigma, experienced stigma, internalized stigma, anticipated stigma, structural stigma, intersectional stigmas	In the context of intersectional and structural stigmas, individual‐level dimensions of HIV‐related stigma operate through interpersonal factors, mental health, psychological resources and biological stress pathways.
**Frameworks addressing stigma and discrimination in healthcare and law**			
UNAIDS [24] Framework name: Examples of stigma and discrimination that create gaps across the HIV prevention cascade Domain: Stigma or discrimination in healthcare settings, stigma or discrimination in law Terminology: Stigma, discrimination Conceptual model	Scope: Healthcare stigma and discrimination that create gaps across the HIV prevention cascade Aim/purpose: NA	Subtype: NA	The framework describes examples of stigma and discrimination that create gaps across the HIV prevention cascade

Abbreviations: NA, not applicable; US, United States.

Eight of these 17 frameworks encompass all three stigma domains examined. While they seek to highlight the complex web of factors affecting different types of stigma and their impacts, their reported scope varies tremendously. For example, Stangl's “Health Stigma and Discrimination Framework” is presented as global, while Woodgate's framework is specific to Indigenous people living with HIV in Canada, and Stevens’ framework focuses on how HIV‐related stigma affects rehabilitation [17, 22, 25].

Seven frameworks address internalized stigma and stigma and discrimination in healthcare, most of which have HIV‐related clinical outcomes as their primary outcomes. Factors along the named pathways to these outcomes vary but frequently include depression, self‐isolation and decreased social support.

Only Turan's framework addresses internalized stigma and stigma and discrimination in law [37]. In this framework, intersecting and structural stigmas operate through interpersonal factors, psychological resources, mental health (including internalized stigma) and stress processes to shape engagement in HIV care and HIV‐related outcomes.

The UNAIDS framework examining gaps across the HIV prevention continuum encompasses stigma and discrimination in both healthcare and law, providing examples as to how these impede HIV prevention [24].

Overall, four frameworks explicitly considered intersecting stigmas [17, 27, 32, 37] (e.g. gender‐related stigma or race‐related stigma alongside HIV‐related stigma) and four explicitly adopted a socio‐ecological framework [17, 25, 26, 33].

### 3.2 Measures

To address key question 2, we documented stigma measures in a comprehensive evidence table in the online Appendix (Table S3). It shows the type of identified stigma and discrimination measures, listing the measure details, reliability and validity. The table shows the main publication and supporting publications also reporting on the measure and contributing additional psychometric information.

Figure 2 summarizes the critical appraisal of all 50 identified measures.

Seventy‐six percent of identified measures reported on internal consistency, such as Cronbach's alpha, and all publications that reported on reliability documented acceptable reliability for the measure's final version or across most subscales. Only seven studies reported on other reliability measures, mostly temporal stability assessed in test‐retest administrations. A quarter of the measures reported on a formal analysis of content validity, for example through expert rating of the appropriateness and spectrum of items. Half of the identified measures reported structural validity, usually based on exploratory or confirmatory factor analysis. Similarly, half of the measures were able to document external validity through correlations with other measures, providing the evidence of convergent or discriminant validity. Of the identified measures, about 40% reported cross‐cultural validity, demonstrated either as part of the measure construction using forward translations in translated measures or reporting the psychometric characteristics for different geographic contexts. Despite the frequency of application of measures, we only found one study explicitly addressing responsiveness, that is documenting the sensitivity of the measure of detecting change [38]. Of the identified measures, 39% reported information of the measure's interpretability, for example documenting the distribution of scores and helping future users of the measure understand what score ranges or cut‐offs constitute a high stigma score.

Table 2 summarizes measures assessing at least two of our three domains of interest.

**Table 2:** Measures addressing multiple stigma domains (*n* = 10)

ID Framework title and type Domain	Stigma/discrimination subtype Framework	Target population Surveyed participants	Scale structure Number of items Answer mode	Reported psychometric characteristics
**Measures addressing internalized stigma, stigma and discrimination in healthcare and law**				
Stigma Index [13] Chinouya et al. [39]; UNAIDS [40] Domain: Internalized stigma, stigma or discrimination in healthcare settings, stigma or discrimination in law Measure name: Stigma Index	Stigma subtype addressed: Experiences of stigma and discrimination and their causes; access to work and services; internal stigma; rights, laws and policies; effecting change; HIV testing; disclosure and confidentiality; treatment; having children; problems and challenges for people living with HIV Underlying framework: NA	Target population: People living with HIV Surveyed participants: Varies	Scale structure: 10 areas: Experiences of stigma and discrimination and their causes; access to work and services; internal stigma; rights, laws and policies; effecting change; HIV testing; disclosure and confidentiality; treatment; having children; problems and challenges for people living with HIV Number of items: NA Answer mode: Answer mode varies by item	Reliability: NA Validity: NA
Dos Santos et al. [41] Domain: Internalized stigma, stigma or discrimination in healthcare settings, stigma or discrimination in law Measure name: People Living with HIV Stigma Index (adaptation)	Stigma subtype addressed: Internalized Underlying framework: NA	Target population: People living with HIV/AIDS Surveyed participants: People living with HIV/AIDS in South Africa	Scale structure: three subdomains covering perceptions of self and internal stigma and examples of stigma or discrimination in different settings, such as the home, community, workplace, religious or healthcare settings; small adaptations were made to the Index, including the quantifying of all qualitative responses into nominal and ordinal scales and the inclusion of South Africa's best‐known national law and policy guidelines as per the Index directives Number of items: NA Answer mode: Rating scale, Dichotomous scale, Answer mode varies by item	Reliability: NA Validity: NA
Biemba et al. [42] Domain: Internalized stigma, stigma or discrimination in healthcare settings, stigma or discrimination in law Measure name: NA	Stigma subtype addressed: Internalized, perceived, enacted Underlying framework: NA	Target population: People living with HIV Surveyed participants: People living with HIV and health workers	Scale structure: Both quantitative and qualitative questions, narrower and modified form of legal environment assessment; questions separate for people living with HIV and health workers Number of items: NR Answer mode: Rating scale, Dichotomous scale, Free text, Answer mode varies by item	Reliability: NA Validity: NA
Friedland et al. [43] HIV Stigma Index 2.0 [44] Domain: Internalized stigma, stigma or discrimination in healthcare settings, stigma or discrimination in law Measure name: People Living with HIV Stigma Index 2.0	Stigma subtype addressed: Internalized Underlying framework: Adapted from the original People Living with HIV (PLHIV) Stigma Index	Target population: People living with HIV Surveyed participants: People living with HIV at least 18 years old who had known their status for at least 1 year	Scale structure: Sections consist of: disclosure, your experience of stigma and discrimination, internalized stigma and resilience, interactions with healthcare services, human rights and effecting change, stigma and discrimination experienced for reasons other than your HIV status, personal experience related to stigma/discrimination Number of items: six items Answer mode: Rating scale, Free text, Answer mode varies by item	Reliability: Good internal consistency (Cronbach's alphas for Cameroon, Senegal and Uganda were 0.70, 0.65 and 0.75, respectively) Validity: Cognitive interview respondents indicated that most questions were well understood and focus group participants said that the Stigma Index 2.0 addressed issues that were relevant to their lives, good construct validity
**Measures addressing internalized stigma and stigma and discrimination in healthcare**				
Bogart et al. [45] Domain: Internalized stigma, stigma or discrimination in healthcare settings Measure name: Multiple Discrimination Scale	Stigma subtype addressed: Interpersonal discrimination, institutional discrimination, violent discrimination Underlying framework: NA	Target population: HIV‐positive Black and Latino men who have sex with men Surveyed participants: HIV‐positive Black and Latino men who have sex with men	Scale structure: three subdomains: discrimination events in the past year due to race/ethnicity (MDS‐Race), sexual orientation (MDS‐Gay) and HIV‐serostatus (MDS‐HIV) Number of items: 30 items total, 10 per subdomain Answer mode: Dichotomous scale	Reliability: Cronbach's alpha >0.80 for all three subscales; follow‐up scores were used to assess test–retest reliability (> 0.60 for all subscales); the three MDS subscales were significantly correlated Validity: All three MDS subscales were significantly associated with validated stigma constructs from prior research, showing high convergent validity
Earnshaw et al. [27] Earnshaw and Chaudoir [28]; Misir [29]; Goodin et al. [30]; Reinius et al. [31] Domain: Internalized stigma, stigma or discrimination in healthcare settings Measure name: HIV Stigma Mechanism Measure	Stigma subtype addressed: Internalized, anticipated, enacted Underlying framework: HIV Stigma Framework (Earnshaw and Chaudoir)	Target population: People living with HIV Surveyed participants: People living with HIV recruited from an inner‐city clinic in the Bronx, NY	Scale structure: three subdomains: internalized, anticipated and enacted Number of items: 24 items total, 6 internalized, 9 anticipated and 9 enacted Answer mode: Rating scale	Reliability: Cronbach's alpha internalized HIV stigma 0.89, anticipated HIV stigma 0.87 and enacted HIV stigma 0.87 Validity: The three scales were considered to be distinct with the majority of variability in each scale non‐overlapping; internalized HIV stigma was uniquely associated with indicators of poorer affective health and wellbeing, including greater helplessness, lower acceptance and lower perceived benefits of having HIV
Neuman et al. [46] Domain: Internalized stigma, stigma or discrimination in healthcare settings Measure name: NA	Stigma subtype addressed: Internalized stigma, interpersonal discrimination, discrimination experienced in healthcare facilities Underlying framework: NA	Target population: HIV‐positive adults Surveyed participants: HIV‐positive adults	Scale structure: three scales—interpersonal discrimination; discrimination experienced in healthcare facilities; and internalized stigma Number of items: 19 total Answer mode: Dichotomous scale	Reliability: Cronbach's alpha scores for both the interpersonal discrimination and healthcare discrimination measures were >0.8, the score for the internalized stigma score was 0.68 Validity: NA
Li et al. [47] Domain: Internalized stigma, stigma or discrimination in healthcare settings Measure name: NA	Stigma subtype addressed: Internalized, personal, occupational Underlying framework: Earnshaw and Chaudoir, 2009; Visser et al., 2008; Stein and Li, 2008	Target population: HIV‐positive patients Surveyed participants: HIV‐positive patients, non‐HIV patients and healthcare providers	Scale structure: three scales; internalized (HIV + patients) and personal stigma (non‐HIV+ patients) scale factors: guilt/blaming and being refused/refusing service; occupational stigma scale factors: blaming, professionalism and egalitarianism Number of items: 31 items across three scales Answer mode: Rating scale	Reliability: Internalized and personal stigma scales with reliability coefficients of 0.869 and 0.853; the occupational stigma scale had a three‐factor structure with a reliability coefficient of 0.839 Validity: Confirmatory factor analysis confirmed that the factors identified from the development samples fit the validation sample; however, all *p*‐values from the chi‐squared goodness‐of‐fit tests were *p* <0.001; among the three study groups, each of the subscales associated with measures of sample characteristics further validated the independence of each factor reflecting that they are representative of an independent sub‐stigma mechanism
Stangl et al. [48] Domain: Internalized stigma, stigma or discrimination in healthcare settings Measure name: NA	Stigma subtype addressed: Internalized, experienced, perceived Underlying framework: Health Stigma and Discrimination Framework (Stangl et al., 2019)	Target population: People living with HIV Surveyed participants: People living with HIV, community members and healthcare workers	Scale structure: seven scales and two experience measures (fear and judgement, internalized stigma, perceived stigma in community, experienced stigma in the community, perceived stigma in the healthcare setting, perceived co‐worker stigma and experienced stigma in healthcare settings) Number of items: 35 questions in total Answer mode: Rating scale, Dichotomous scale	Reliability: Acceptable to very good internal consistency Validity: Subgroup factor analysis confirmed acceptable reliability for all three scales by country, sex and type of health worker
**Measures addressing stigma and discrimination in healthcare and law**				
UNAIDS [49] UNAIDS, WHO [50] Domain: Stigma or discrimination in healthcare settings, stigma or discrimination in law Measure name: National Commitments and Policy Instrument	Stigma subtype addressed: NA Underlying framework: NA	Target population: People living with HIV Surveyed participants: People living with HIV	Scale structure: two parts (Part A for national authorities and Part B for non‐governmental partners) Number of items: 10 target areas Answer mode: Unclear	Reliability: NA Validity: NA

Abbreviations: NA, not applicable; NR, not reported; NY, New York.

Some measures address all three types of stigma of interest, including the People Living with HIV Stigma Index (and its version 2.0). The website for this measure states that it has been used in many more countries and languages than we found through this review, and reports are available for many countries worldwide [13].

One other measure assessed all three types of stigma: a report of the findings of an HIV‐related legal assessment in Zambia [42]. It encompassed legal and policy, survey and qualitative data, each presented separately but analysed jointly, providing an interesting model that might be adapted for use in other countries.

Five other measures were found that cover both internalized stigma and stigma and discrimination in healthcare. Each one is structured differently, capturing different elements of stigma. For example, within the Multiple Discrimination Scale, HIV‐related stigma was assessed using Kalichman's Internal AIDS‐Related Stigma Scale and the “experienced stigma” subscale from the People Living with HIV Stigma Index [45]. Alongside this, other pre‐validated scales were included to assess stigma related to race/ethnicity and sexual orientation, with findings reported individually for each type of stigma as well as aggregated into an unweighted total.

Only the National Commitments and Policy Instrument focuses on stigma and discrimination in healthcare and in law and policy. It contains a range of relevant indicators on experiences of stigma and discrimination in healthcare, laws that might be discriminatory and laws that protect against HIV‐related discrimination [49].

The scales that are most frequently adopted or adapted are those initially published by Berger (not shown in the table above as it focuses only on internalized stigma) and Earnshaw [27, 51].

The measure evidence table in the online Appendix (Table S4) documents the available measures in detail. Alongside the studies discussed above, it includes measures that assess a single stigma domain relevant to this review.

### 3.3 Discussion

This review synthesizes a complex range of data covering frameworks and measures across the three identified domains of stigma. This evidence base helps identify opportunities and challenges, with a view to stimulating further discussion and advancing the field both conceptually and practically.

### 3.4 Language of defining stigma and discrimination

There is much variation in how authors described/defined stigma. Language used included, in addition to internalized stigma, stigma and discrimination in healthcare settings and in law and policy, self‐stigma, felt stigma, enacted stigma, anticipated stigma, perceived stigma, personal stigma and more. In addition, scale components were sometimes described using language that can be interpreted to be about stigma even when stigma is not explicitly named. With this range of language, inconsistently used, it can be difficult to ascertain, at face value what a framework or measure actually captures and how comparable it might be to others.

The understandable drive towards context‐ and construct‐specific frameworks and measures has perhaps splintered the concept of stigma to such an extent that it hampers comparability, cross‐setting learning and efforts to assess progress towards global targets. Our review aims to help address this by providing an overview and compendium of existing resources. Determining a standardized nomenclature for different types of stigma for use across frameworks and measures, that can be locally tailored, might be an important next step.

### 3.5 Variety within frameworks and measures for internalized stigma

Within frameworks that address internalized stigma, this concept appears variably as the starting point of the framework, in the middle or as the outcome. The most common associations are between internalized stigma and mental health or HIV‐related clinical outcomes.

Across both frameworks and tools, some measure HIV‐related stigma broadly, with a sub‐component/scale to capture internalized stigma, while others focus only on internalized stigma.

There is variety in terms of what the measures actually measure, with regard to both content and specificity of responses: the measures include different numbers of items, some are assessed dichotomously, while others use a Likert scale, and different time periods are covered. Thus, even where content is similar, assessments can look very different. No qualitative measures were identified.

The People Living with HIV Stigma Index 2.0, in its assessment of internalized stigma, also includes a “resilience scale” [43]. Capturing concepts, such as self‐respect, self‐confidence and the ability to feel love, this scale provides a positive framing within which resilience is seen as a counter measure to internalized stigma.

### 3.6 Variety within frameworks and measures for stigma and discrimination in healthcare

Some frameworks focus exclusively on stigma and discrimination in healthcare, while others include this as component of a broader HIV‐related stigma framework. The specificity of the framework determines the degree to which stigma and discrimination are explored, with focused frameworks providing more depth. Most of the frameworks capture triggers, manifestations and impacts within healthcare. Four frameworks are designed to inform action to reduce stigma and discrimination in healthcare [52, 53, 54, 55], while the UNAIDS frameworks identify how stigma and discrimination in healthcare impact the HIV prevention and care and treatment cascades, and how interventions might help address this.

Many measures capture beliefs and practices among health workers, some capture health worker and client perspectives through separate sub‐scales and a few capture client experiences. Tested across diverse geographies and populations, these measures do not generally appear comparable. The UNAIDS indicators, included in the Global AIDS Monitoring framework, designed for use by all national governments reporting to UNAIDS, are the exception [49].

There is variety in the scope and specificity of measures: some measures of stigma and discrimination in healthcare are general, some capture something more specific, for example how stigma impacts decisions around childbearing among people living with HIV, and some also capture additional stigma.

### 3.7 Variety within frameworks and measures for stigma and discrimination in law and policy

Where stigma and discrimination in law and policy are included in frameworks, this is usually generic with laws and policies mentioned as part of the macro‐system or structural factors within a socio‐ecological model. The UNAIDS models usefully point to specific laws that can be discriminatory and affect HIV‐related outcomes, and Hagopian and colleagues provide a framework specific to how anti‐homosexuality laws affect HIV‐related stigma and outcomes [24, 56]. Stangl's framework draws attention to the existence of laws and policies as well as law enforcement practices and access to justice so as to capture information on implementation, which might also be discriminatory [17].

Three measures assessed stigma and discrimination in law and policy. The National Commitments and Policies Instrument and the Stigma Index, both of which are widely used, include quantitative measures of stigma and discrimination in law and policy, while Biemba and colleagues provide the only mixed‐methods assessment [42, 43, 49].

Overall, there is a dearth of measures relating to HIV stigma and discrimination in law and policy. This may be due to the complexity and sensitivity of measuring these topics and the extensive investment that would be required to do this effectively at scale. Data are increasingly available about the existence of discriminatory laws and policies, but additional attention is needed to measure and evaluate their implementation to identify if, when or how these processes and structures have ramifications at the healthcare and personal levels.

### 3.8 Looking across domains of stigma

Many of the frameworks that encompass different domains of stigma use variations of the socio‐ecological framework to capture relevant factors from the individual to environmental levels. However, very few operate across all levels, and none sufficiently capture the three intertwined domains of stigma studied.

There are other domains of HIV‐related stigma not included in this review as well as other types of stigma and discrimination that can intersect with HIV‐related stigma, such as stigma and discrimination based on race/ethnicity, gender, sexual orientation or gender identity. Using any of these entry points, additional frameworks and measures might be identified that might help understand the domains of stigma studied here, particularly when these different types of stigma and discrimination are concurrently experienced.

### 3.9 Looking across frameworks and measures

Focusing on those that seek to address more than one of our stigma domains of interest, frameworks are more encompassing than measures, bringing attention to the wide range of factors that influence experiences and outcomes. Understandably, no measure is sufficiently comprehensive to capture all of this. Across most of the frameworks, the components are very broad (e.g. mental health and culture), raising challenges for how each one might be measured. Further specificity and explicit definition might be required to ensure adequate measurement. This might be done as part of local adaptation, even as this may reduce comparability. Box 1 provides some guiding questions to help determine which framework and/or measures might be most useful in different situations.

**Box 1: Considerations for selecting a framework and measure**

This is not a stepwise process, simply guidance on issues to think through in trying to establish if existing HIV‐related frameworks and measures might fit well with your planned work. Attention is also needed to ensure good fit between the chosen framework and measure(s).

Selecting a framework

‐What type(s) of HIV‐related stigma do you want to address? Is there an existing framework that matches this scope?
‐What population(s) are you planning to work with and where? Have any frameworks been used in these contexts before?
‐Where on the causal pathway does this stigma sit in your work: is it a predictor, intermediate or outcome variable? Which existing frameworks mirror this? And which ones also include other variables that you already think are important?
‐Do you also want there to be attention to other, intersecting stigmas?
‐What empirical or conceptual grounding underlying these frameworks aligns with your planned work?

Selecting measures

‐Based on the scope of your work, is there an existing single measure that can cover everything you need or might you need to use multiple measures?
‐Whose perspective(s)/experience(s) does each measure capture? Does that align with what you want to learn?
‐For each measure, has it been validated? In your population, language and context of interest?
‐In each case, has its reliability, interpretability and responsiveness been assessed?
‐What time period does the measure cover (ever, last 30 days etc.)? Will this help you learn what you need in your work?
‐How specific are response options (e.g. Y/N vs. Likert scale) and does that match your needs?

### 3.10 The challenge of comparability and context specificity

A plethora of measures exist, particularly for internalized stigma and stigma and discrimination experienced in healthcare, but their comparability is limited by their diversity. A recent review of interventions to address self‐stigma did not include a formal meta‐analysis due to the heterogeneity of measures used [57], and a 2015 UNAIDS report documented over 60 tools to assess and/or address stigma and discrimination just within healthcare [58]. While the need for local adaptation is evident, having a common starting point could help promote a balance of locally tailored yet internationally comparable data.

Although Stigma Index country reports are available online, data are rarely used in peer‐reviewed literature. It would be helpful to see additional analyses of these data alongside their conceptual frameworks and information on sample sizes and sampling frames to help contextualize findings. Information on local adaptations might also help understand the comparability of findings.

### 3.11 Implications for the HIV continuum of care

Many of the frameworks reviewed illustrate how stigma and discrimination are barriers to access across different points of the HIV care continuum. Recent modelling has estimated that reaching the UNAIDS societal enabler targets (which include “less than 10% of people living with HIV and key populations experience stigma and discrimination”) will prevent 2.5 million new infections and 1.7 million AIDS‐related deaths by 2030 [59]. Understanding how stigma and discrimination are being experienced, and being able to measure the impact of interventions to reduce them all along the continuum of care is critical to achieving global HIV targets, including the “95%‐95%‐95%” targets for HIV testing, treatment and viral suppression.

### 3.12 Limitations

This review provides a comprehensive overview of existing frameworks and measures to advance the science of HIV‐related stigma research. However, some limitations should be noted. The review was limited to newer work published since the publication of the original People Living with HIV Stigma Index [13]. We analysed only scientific articles or reports so that we could critically appraise the frameworks and measures, even as this may exclude the latest developments recently published in conference abstracts. The frameworks and measures reviewed are, to varying degrees, designed for the context within which they were developed; while some aspects might be universal, others may need to be refined for use in other social, cultural and economic contexts.

## 4 CONCLUSIONS

Given the level of attention to addressing HIV‐related stigma and discrimination, this review is particularly timely and can inform responses from global to local levels. The current Global AIDS Strategy and the 2021 Political Declaration on HIV and AIDS underscore the importance of addressing HIV‐related stigma and discrimination in order to achieve global and national HIV targets. This will require rigorous measurement of stigma and discrimination across different spheres, including internalized stigma, and stigma and discrimination in healthcare and law and policy. The challenge remains how to do this with frameworks and measures that are both locally appropriate and globally comparable. Experts in the field from different stakeholder groups could usefully collaborate to guide a more streamlined approach for the field. People living with HIV must be at the centre of this work and support will be required from funders, international agencies and governments to ensure a process and outcomes that might gain broad traction.

Most importantly, the goal must be to understand, measure and help mitigate and alleviate the impact of different types of stigma. Frameworks and measures must be fit to help direct investment, prioritize appropriate actions and strengthen learning about effectiveness. This review provides a basis to seek consensus about appropriate concepts and measures to understand the experiences and drivers of stigma for different people in diverse contexts around the world. It is up to us all to ensure this consensus exercise takes place, and that ultimately the results translate into reducing stigma and enhancing the health, quality of life and human rights of all people.

## COMPETING INTERESTS

None of the authors have any competing interests.

## AUTHORS’ CONTRIBUTIONS

SH, LF and SG developed the systematic review. SH, AM, MB, SY, NF, NC and MR ran the searches and extracted the data. LF and SH wrote the first draft of the manuscript. All authors reviewed and revised the manuscript. All authors have read and approved the final manuscript. LF had final responsibility for the decision to submit for publication.

## FUNDING

This work was supported, in whole or in part, by the Bill & Melinda Gates Foundation (INV‐004364). Under the grant conditions of the Foundation, a Creative Commons Attribution 4.0 Generic License has already been assigned to the Author Accepted Manuscript version that might arise from this submission.

## Supporting information


**Figure S1**. Literature flow diagram.
**Table S1**. Evidence table for frameworks.
**Table S2**. Critical appraisal frameworks.
**Table S3**. Critical appraisal for measures.
**Table S4**. Evidence table for measures.

## Data Availability

The authors confirm that the data supporting the findings of this study are available within the article.
