# Supplementary material for: Frameworks and measures for HIV‐related internalized stigma, stigma and discrimination in healthcare and in laws and policies: a systematic review
Source: J Int AIDS Soc. 2022 Jul 12;25(Suppl 1):e25915. doi: 10.1002/jia2.25915 (PMC9274352; doi:10.1002/jia2.25915)
Supplement: Supplementary file 1 — Figure S1. Literature flow diagram. Table S1. Evidence table for frameworks. Table S2. Critical appraisal frameworks. Table S3. Critical appraisal for measures. Table S4. Evidence table for measures. [file JIA2-25-e25915-s001.docx]

# Online Appendix

**Search Strategy**

PubMed

Date: 1/11/2021

Yield: 112

Terms

("framework"[All Fields] OR "framework s"[All Fields] OR "frameworks"[All Fields] OR "logic model"[All Fields] OR "analytic model"[All Fields]) AND ("stigma"[Title] OR "discrimination"[Title]) AND ("hiv"[MeSH Terms] OR "hiv"[All Fields] OR ("acquired immunodeficiency syndrome"[MeSH Terms] OR ("acquired"[All Fields] AND "immunodeficiency"[All Fields] AND "syndrome"[All Fields]) OR "acquired immunodeficiency syndrome"[All Fields] OR "aids"[All Fields]))

OR

Date: 1/11/2021

Yield: 112

Filter: 2008-date

Terms

("measure*"[Title] OR "test"[Title] OR ("scale s"[All Fields] OR "scaled"[All Fields] OR "scaling"[All Fields] OR "scalings"[All Fields] OR "weights and measures"[MeSH Terms] OR ("weights"[All Fields] AND "measures"[All Fields]) OR "weights and measures"[All Fields] OR "scale"[All Fields] OR "scales"[All Fields]) OR "tool"[All Fields] OR "assess*"[Title] OR ("psychometrical"[All Fields] OR "psychometrically"[All Fields] OR "psychometrics"[MeSH Terms] OR "psychometrics"[All Fields] OR "psychometric"[All Fields])) AND ("stigma"[Title] OR "discrimination"[Title]) AND ("hiv"[MeSH Terms] OR "hiv"[All Fields] OR ("acquired immunodeficiency syndrome"[MeSH Terms] OR ("acquired"[All Fields] AND "immunodeficiency"[All Fields] AND "syndrome"[All Fields]) OR "acquired immunodeficiency syndrome"[All Fields] OR "aids"[All Fields]))

OR

Date: 1/11/2021

Filter: Systematic reviews

Yield: 225

Terms

("social stigma"[MeSH Terms] OR ("social"[All Fields] AND "stigma"[All Fields]) OR "social stigma"[All Fields] OR "stigma"[All Fields] OR "stigmas"[All Fields] OR "stigma s"[All Fields] OR ("discriminabilities"[All Fields] OR "discriminability"[All Fields] OR "discriminable"[All Fields] OR "discriminably"[All Fields] OR "discriminance"[All Fields] OR "discriminant"[All Fields] OR "discriminants"[All Fields] OR "discriminate"[All Fields] OR "discriminated"[All Fields] OR "discriminates"[All Fields] OR "discriminating"[All Fields] OR "discrimination, psychological"[MeSH Terms] OR ("discrimination"[All Fields] AND "psychological"[All Fields]) OR "psychological discrimination"[All Fields] OR "discrimination"[All Fields] OR "discriminations"[All Fields] OR "discriminative"[All Fields] OR "discriminatively"[All Fields] OR "discriminator"[All Fields] OR "discriminators"[All Fields])) AND ("hiv"[MeSH Terms] OR "hiv"[All Fields] OR ("acquired immunodeficiency syndrome"[MeSH Terms] OR ("acquired"[All Fields] AND "immunodeficiency"[All Fields] AND "syndrome"[All Fields]) OR "acquired immunodeficiency syndrome"[All Fields] OR "aids"[All Fields]))

OR

Date: 2/5/2021

Yield: 199 results

Filters: Randomized Controlled Trial, English language, 2008 - 2021

Terms

("social stigma"[MeSH Terms] OR ("social"[All Fields] AND "stigma"[All Fields]) OR "social stigma"[All Fields] OR "stigma"[All Fields] OR "stigmas"[All Fields] OR "stigma s"[All Fields] OR ("discriminabilities"[All Fields] OR "discriminability"[All Fields] OR "discriminable"[All Fields] OR "discriminably"[All Fields] OR "discriminance"[All Fields] OR "discriminant"[All Fields] OR "discriminants"[All Fields] OR "discriminate"[All Fields] OR "discriminated"[All Fields] OR "discriminates"[All Fields] OR "discriminating"[All Fields] OR "discrimination, psychological"[MeSH Terms] OR ("discrimination"[All Fields] AND "psychological"[All Fields]) OR "psychological discrimination"[All Fields] OR "discrimination"[All Fields] OR "discriminations"[All Fields] OR "discriminative"[All Fields] OR "discriminatively"[All Fields] OR "discriminator"[All Fields] OR "discriminators"[All Fields]))

AND

("hiv"[MeSH Terms] OR "hiv"[All Fields] OR ("acquired immunodeficiency syndrome"[MeSH Terms] OR ("acquired"[All Fields] AND "immunodeficiency"[All Fields] AND "syndrome"[All Fields]) OR "acquired immunodeficiency syndrome"[All Fields] OR "aids"[All Fields]))

OR

(systematic reviews)

Date: 5/6/2021

Yield: 235

Publication Date Range: 2008-05/06/2021

Filter: Systematic Review

Terms

Social stigma[MESH terms] OR stigma OR discrimination OR discrimination, psychological[MESH terms]

AND

HIV[Mesh terms] OR hiv OR acquired immunodeficiency syndrome[MESH terms] OR "acquired immunodeficiency syndrome" OR "AIDS"[Title/Abstract]

PsycINFO

(Frameworks; limited ‘framework’ terms to title field, 2008-2021 publication date range)

Date: 3/26/2021

Yield: 41

Terms

(ti(stigma OR discrimin*) OR ab(stigma OR discrimin*) OR su(stigma OR discrimin*)) AND (ti(HIV OR "acquired immunodeficiency syndrome") OR ab(HIV OR "acquired immunodeficiency syndrome") OR su(HIV OR "acquired immunodeficiency syndrome")) AND ti(framework OR frameworks OR "logic model" OR "analytic model") AND la.exact("ENG")

OR

(Measures; limited ‘measures’ terms to title field)

Date: 3/26/2021

Yield: 116

Terms

(ti(stigma OR discrimin*) OR ab(stigma OR discrimin*) OR su(stigma OR discrimin*)) AND (ti(HIV OR "acquired immunodeficiency syndrome") OR ab(HIV OR "acquired immunodeficiency syndrome") OR su(HIV OR "acquired immunodeficiency syndrome")) AND ti(measure OR measures OR scale OR scales OR tool OR tools) AND (la.exact("ENG") AND pd(20080101-20211231))

OR

Date: 3/19/2021

Yield: 58

Terms

((ti(stigma OR discrimin*) OR ab(stigma OR discrimin*) OR su(stigma OR discrimin*)) AND (ti(HIV OR "acquired immunodeficiency syndrome") OR ab(HIV OR "acquired immunodeficiency syndrome") OR su(HIV OR "acquired immunodeficiency syndrome")))

AND

(la.exact("ENG") AND me.exact("Prospective Study" OR "Clinical Trial")

AND

pd(20080101-20211231))

OR

Date: 5/6/2021

Yield: 100

Publication Date Range: After January 1, 2008

Methodology: Systematic Review

Terms

noft(stigma OR discriminat*) AND noft("hiv" OR "acquired immunodeficiency syndrome" OR "AIDS")

Web of Science

(Framework; omitted conference proceedings, added Research Areas, limited ‘framework’ terms to title field, 2008-2021 publication date range)

Yield: 14

Terms

(TS=(stigma OR discrim*) AND TS=(HIV OR "acquired immunodeficiency syndrome") AND TI=(framework OR frameworks OR "logic model" OR "logic models" OR "analytic model" OR "analytic models")) AND LANGUAGE: (English)

Refined by: RESEARCH AREAS: (HEALTH CARE SCIENCES SERVICES OR PSYCHOLOGY )

Indexes=SCI-EXPANDED, SSCI, BKCI-S, BKCI-SSH, ESCI Timespan=1900-2021

OR

(Measures; omitted conference proceedings, added Research Areas, limited ‘measures’ terms to title field)

Yield: 75

TS=(stigma OR discrim*) AND TS=(HIV OR "acquired immunodeficiency syndrome") AND TI=(measure OR measures OR scale OR scales OR tool OR tools)) AND LANGUAGE: (English)

Refined by: RESEARCH AREAS: ( PSYCHOLOGY OR HEALTH CARE SCIENCES SERVICES )

Indexes=SCI-EXPANDED, SSCI, BKCI-S, BKCI-SSH, ESCI Timespan=2008-2021

OR

Interventions (omitted conference proceedings, added Research Areas, limited ‘RCT’ terms to topic field)

Yield: 76

(TS=(stigma OR discrim*) AND TS=(HIV OR "acquired immunodeficiency syndrome") AND TS=("randomized controlled trial" OR "randomized controlled trials" OR RCT OR RCTs OR "intervention study" OR "intervention studies")) AND LANGUAGE: (English)

Refined by: RESEARCH AREAS: (PSYCHOLOGY OR HEALTH CARE SCIENCES SERVICES)

Indexes=SCI-EXPANDED, SSCI, BKCI-S, BKCI-SSH, ESCI Timespan=2008-2021

OR

Date: 5/6/2021

Yield: 263

Publication Date Range: 2008-2021

Terms

Indexes=SCI-EXPANDED, SSCI, A&HCI, CPCI-S, CPCI-SSH, BKCI-S, BKCI-SSH, ESCI, CCR-EXPANDED

Search Strategy

TOPIC: (stigma OR discriminat*) AND TOPIC: ("hiv" OR "acquired immunodeficiency syndrome" OR "AIDS") AND TOPIC: ("systematic review")

Campbell Collaboration

Date: 1/19/2021

Yield: 81

Campbell Systematic Reviews

Terms

stigma* OR discriminat*" and "HIV OR AIDS OR "acquired immunodeficiency"

Research evidence by topic

(stigma OR discrimination)

(HIV OR AIDS)

Cochrane Database of Systematic Reviews

Date: 1/29/2021

Yield: 6

Terms

(stigma OR discrimination):ti,ab,kw (Word variations have been searched)

AND

MeSH descriptor: [HIV] explode all trees OR MeSH descriptor: [Acquired Immunodeficiency Syndrome] 5 tree(s) exploded OR HIV OR "acquired immunodeficiency syndrome" OR "acquired immunodeficiency syndrome"):ti,ab,kw (Word variations have been searched)

PROSPERO

Date: 1/19/2021

Yield: 173

Terms

(stigma or discrimin*)

AND

MeSH DESCRIPTOR HIV EXPLODE ALL TREES OR MeSH DESCRIPTOR Acquired Immunodeficiency Syndrome EXPLODE ALL OR "acquired immunodeficiency syndrome" OR "acquired immunodeficiency" OR HIV

Open Science Framework

Date: 1/27/2021

Yield: 4 relevant projects

Terms: (stigma OR discrimination) AND (HIV OR AIDS)

Universal Human Rights Index

Date: 1/27/2021

Platform: https://www.ohchr.org/EN/Issues/HIV/Pages/Documents.aspx

Yield: 2 relevant documents

Terms: “Persons living with HIV/AIDS” AND (stigma OR discrimination)

PAIS

Date: 4/9/2021

Yield: 9

Terms: NOFT(hive and stigma and law and intervention)

International AIDS Society (IAS)

Date: 1/27/2021

Yield: 5 relevant documents

Terms: (stigma OR discrimination) AND (HIV OR AIDS)

Joint United Nations Programme on HIV/AIDS (UNAIDS)

Date: 1/27/2021

Yield: 15 relevant documents

Terms: (stigma OR discrimination) AND (HIV OR AIDS)

United Nations Development Programme (UNDP)

Date: 4/13/2021

Yield: 6 relevant documents

Terms: (stigma OR discrimination) AND (HIV OR AIDS)

STRIVE:

Date: 1/27/2021

Yield: 2 relevant documents

Terms: (stigma OR discrimination) AND (HIV OR AIDS)

Health Policy Plus

Date: 1/27/2021

Yield: 5 relevant documents

Terms: (stigma OR discrimination) AND (HIV OR AIDS)

The Center for HIV Law and Policy

https://www.hivlawandpolicy.org/

Date: 4/19/2021

Yield: 15 relevant documents

Terms: topic: “stigma”

The Global Fund

https://www.theglobalfund.org/en/funding-model/throughout-the-cycle/community-rights-gender/

Date: 4/13/2021

Yield: 20 relevant documents

Terms: “Human rights”

HIV Legal Network

http://www.hivlegalnetwork.ca/site/?lang=en

Date: 4/16/2021

Publications/Find Publications/ keyword search:

Yield: 11 relevant documents

Terms: stigma

Sage: A resource-sharing community for Canadian HIV and hepatitis C service providers

http://sagecollection.ca/en

Limited publication type: Reports

Date: 4/16/2021

Yield: 3 potentially relevant documents

Terms: STIGMA AND (LAW OR LEGAL)

HeinOnline

Date: 3/19/2021

Yield: 272

Terms

(stigma OR discrmin*) AND (HIV OR "acquired immunodeficiency syndrome" ) AND (framework OR frameworks OR "logic model" OR "logic models" OR "analytic model" OR "analytic models") Topic: AIDS AND Timespan: 2008-2021

OR (stigma OR discrmin*) AND (HIV OR "acquired immunodeficiency syndrome" ) AND (measure OR measures OR scale OR scales OR asses* OR tool OR tools) AND Topic: AIDS AND Timespan: 2008-2021

OR

(stigma OR discrmin*) AND (HIV OR "acquired immunodeficiency syndrome" ) AND ("randomized controlled trial" OR "randomized controlled trials" OR "RCT" OR "RCTs") Topic: AIDS AND Timespan: 2008-2021

OR

(stigma OR discrmin*) AND (HIV OR "acquired immunodeficiency syndrome" ) AND ("systematic review" OR "systematic reviews") Topic: AIDS AND Timespan: 2008-2021

LegalTrac

Date: 5/12/2021

Yield: 78

Terms

keyword search - ("acquired immunodeficiency syndrome" OR HIV) AND (stigma OR discriminat*)

AND

Publication Date Range: 2008 - present

AND

Document Type: Article

**Eligibility criteria**

We used a PICOTS (participant, independent variable, comparator or study design, outcome/measure, timing, and setting) framework to structure the eligibility criteria:

- Participants: People living with or perceived to be living with HIV and people from groups who are disproportionately affected by HIV infection. We excluded studies of mixed populations and other participant targets unless the study provided HIV-relevant subgroup analyses.
- Independent variable: Frameworks and measures addressing HIV-related internalized stigma, stigma and discrimination in healthcare, and stigma and discrimination in laws and policies:
  - Frameworks included work outlining and relating to multiple components of stigma and/or discrimination, including conceptual frameworks, logic models, taxonomies, and analytic models for assessment, prevention, reduction, or mitigation of stigma. We included self-identified frameworks and included publications that showed a schematic representation of stigma/discrimination in the form of a figure or structured table. The framework could be an adaptation of an existing framework but had to be specific to HIV-related stigma or discrimination.
  - Measures included self and peer report measures used for formal assessment of stigma and discrimination. Studies using a published measure were included if the development and psychometric properties of the measure were a central focus of the study; all items had to be reported to be eligible. We included conceptual and methodological adaptations and improvements of measures.
- Comparator/study design: Publications introducing frameworks were included regardless of the comparator or study design. Measure research had to describe the tool in sufficient detail to be included but needed no comparator.
- Outcome: Framework publications were included regardless of any reported outcomes. Measure research had to report a description of the measure, the development process, or the evaluation or validation of the measure.
- Timing: Only publications from 2008 on were included, building on the first People Living with HIV Stigma Index published in 2008, which transformed thinking around HIV-related stigma measurement, fostering new levels of openness, nuance and confidence in stigma measures. To maintain consistency in approaches to reviewing measures and frameworks, the same cut-off date was used for searches for frameworks.
- Setting: The review was not restricted by setting but we restricted to English language for both frameworks and measures. Measures designed for other languages were included if the publication also presented an English translation.

**Appendix Figure 1. Literature flow diagram**

Records identified through database searching
(n = 1,855)

Full-text publications assessed for eligibility
(n = 1,050)

Additional records identified through other sources
(n = 344)

Citations screened
(n = 2,199)

Excluded Citations,

not comparative study, not SR, or not on topic
(n = 1,149)

Full-text articles excluded, with reasons
(n = 488)

Exclude-Participants: n = 30

Exclude-Intervention: n = 247

Exclude-Outcome: n = 38

Exclude-Study Design: n = 49

Exclude-Language: n = 15

Exclude-Publication year: n = 35

Exclude-Not best evidence: n = 15

Duplicate: n = 59

Background

(n = 79)

Systematic reviews

(n = 190)

Intervention studies

(n = 148)

Included studies

Frameworks: n = 69 [1-69]

Measures: n = 50 [14, 70-118]

(118 studies [1-118] reported in 145 publications [1-145])

**References**

1 Boyes ME, Pantelic M, Casale M, Toska E, Newnham E, Cluver LD. Prospective associations between bullying victimisation, internalised stigma, and mental health in South African adolescents living with HIV. J Affect Disord. 2020;276:418-23.

2 Casale M, Boyes M, Pantelic M, Toska E, Cluver L. Suicidal thoughts and behaviour among South African adolescents living with HIV: Can social support buffer the impact of stigma? J Affect Disord. 2019;245:82-90.

3 Chan RCH, Mak WWS. Cognitive, Regulatory, and Interpersonal Mechanisms of HIV Stigma on the Mental and Social Health of Men Who Have Sex With Men Living With HIV. Am J Mens Health. 2019;13(5):1557988319873778.

4 Christopoulos KA, Neilands TB, Dilworth S, Lisha N, Sauceda J, Mugavero MJ, et al. Internalized HIV stigma predicts subsequent viremia in US HIV patients through depressive symptoms and ART adherence. Aids. 2020;34(11):1665-71.

5 Clum G, Chung SE, Ellen JM, Adolescent Medicine Trials Network for HIVAI. Mediators of HIV-related stigma and risk behavior in HIV infected young women. AIDS Care. 2009;21(11):1455-62.

6 Cluver L, Orkin M, Boyes ME, Sherr L, Makasi D, Nikelo J. Pathways from parental AIDS to child psychological, educational and sexual risk: developing an empirically-based interactive theoretical model. Soc Sci Med. 2013;87:185-93.

7 Colbert AM, Kim KH, Sereika SM, Erlen JA. An examination of the relationships among gender, health status, social support, and HIV-related stigma. JANAC: Journal of the Association of Nurses in AIDS Care. 2010;21(4):302-13.

8 Crockett KB, Kalichman SC, Kalichman MO, Cruess DG, Katner HP. Experiences of HIV-related discrimination and consequences for internalised stigma, depression and alcohol use. Psychol Health. 2019;34(7):796-810.

9 Darlington CK, Hutson SP. Understanding HIV-related stigma among women in the Southern United States: A literature review. AIDS and Behavior. 2017;21(1):12-26.

10 Davtyan M, Olshansky EF, Brown B, Lakon C. A Grounded Theory Study of HIV-Related Stigma in U.S.-Based Health Care Settings. J Assoc Nurses AIDS Care. 2017;28(6):907-22.

11 Deacon H. Understanding HIV/AIDS Stigma. A Theoretical and Methodological Analysis. Cape Town: HSRC Press; 2005.

12 Diiorio C, McCarty F, Depadilla L, Resnicow K, Holstad MM, Yeager K, et al. Adherence to antiretroviral medication regimens: a test of a psychosocial model. AIDS Behav. 2009;13(1):10-22.

13 Earnshaw VA, Bogart LM, Dovidio JF, Williams DR. Stigma and racial/ethnic HIV disparities: moving toward resilience. Am Psychol. 2013;68(4):225-36.

14 Earnshaw VA, Smith LR, Chaudoir SR, Amico KR, Copenhaver MM. HIV stigma mechanisms and well-being among PLWH: a test of the HIV stigma framework. AIDS Behav. 2013;17(5):1785-95.

15 Fekete EM, Williams SL, Skinta MD. Internalised HIV-stigma, loneliness, depressive symptoms and sleep quality in people living with HIV. Psychol Health. 2018;33(3):398-415.

16 Florom-Smith AL, De Santis JP. Exploring the concept of HIV-related stigma. Nurs Forum. 2012;47(3):153-65.

17 France NF, McDonald SH, Conroy RR, Byrne E, Mallouris C, Hodgson I, et al. "An unspoken world of unspoken things": a study identifying and exploring core beliefs underlying self-stigma among people living with HIV and AIDS in Ireland. Swiss Med Wkly. 2015;145:w14113.

18 Gilbert L. 'The mercurial piece of the puzzle': Understanding stigma and HIV/AIDS in South Africa. Sahara j. 2016;13(1):8-16.

19 Hagopian A, Rao D, Katz A, Sanford S, Barnhart S. Anti-homosexual legislation and HIV-related stigma in African nations: what has been the role of PEPFAR? Glob Health Action. 2017;10(1):1306391.

20 Health Policy Plus. Groundbreaking Research and Initiatives to Measure and Reduce Stigma and Discrimination. Washington, DC; 2017.

21 Ikeda DJ, Nyblade L, Srithanaviboonchai K, Agins BD. A quality improvement approach to the reduction of HIV-related stigma and discrimination in healthcare settings. BMJ Glob Health. 2019;4(3):e001587.

22 Jain A, Nyblade L. Scaling Up Policies, Interventions, and Measurement for Stigma-Free HIV Prevention, Care, and Treatment Services. Washington, DC: Futures Group, Health Policy Project; 2012.

23 Katz IT, Ryu AE, Onuegbu AG, Psaros C, Weiser SD, Bangsberg DR, et al. Impact of HIV-related stigma on treatment adherence: systematic review and meta-synthesis. J Int AIDS Soc. 2013;16(3 Suppl 2):18640.

24 Katz IT, Ryu AE, Onuegbu AG, Psaros C, Weiser SD, Bangsberg DR, et al. Impact of HIV-related stigma on treatment adherence: systematic review and meta-synthesis. J Int AIDS Soc. 2013b;16(3 Suppl 2):18640.

25 Lipira L, Williams EC, Huh D, Kemp CG, Nevin PE, Greene P, et al. HIV-Related Stigma and Viral Suppression Among African-American Women: Exploring the Mediating Roles of Depression and ART Nonadherence. AIDS Behav. 2019;23(8):2025-36.

26 Logie CH, Ahmed U, Tharao W, Loutfy MR. A Structural Equation Model of Factors Contributing to Quality of Life Among African and Caribbean Women Living with HIV in Ontario, Canada. AIDS Res Hum Retroviruses. 2017;33(3):290-7.

27 Logie CH, Jenkinson JI, Earnshaw V, Tharao W, Loutfy MR. A Structural Equation Model of HIV-Related Stigma, Racial Discrimination, Housing Insecurity and Wellbeing among African and Caribbean Black Women Living with HIV in Ontario, Canada. PLoS One. 2016;11(9):e0162826.

28 Logie CH, Lacombe-Duncan A, Wang Y, Kaida A, Conway T, Webster K, et al. Pathways From HIV-Related Stigma to Antiretroviral Therapy Measures in the HIV Care Cascade for Women Living With HIV in Canada. J Acquir Immune Defic Syndr. 2018;77(2):144-53.

29 Logie CH, Wang Y, Lacombe-Duncan A, Wagner AC, Kaida A, Conway T, et al. HIV-related stigma, racial discrimination, and gender discrimination: Pathways to physical and mental health-related quality of life among a national cohort of women living with HIV. Prev Med. 2018;107:36-44.

30 Logie CH, Williams CC, Wang Y, Marcus N, Kazemi M, Cioppa L, et al. Adapting stigma mechanism frameworks to explore complex pathways between intersectional stigma and HIV-related health outcomes among women living with HIV in Canada. Soc Sci Med. 2019;232:129-38.

31 Mahajan AP, Sayles JN, Patel VA, Remien RH, Sawires SR, Ortiz DJ, et al. Stigma in the HIV/AIDS epidemic: A review of the literature and recommendations for the way forward. AIDS. 2008;22(Suppl2):S67-S79.

32 Meyerson B, Barnes P, Emetu R, Bailey M, Ohmit A, Gillespie A. Institutional and structural barriers to HIV testing: Elements for a theoretical framework. AIDS Patient Care and STDs. 2014;28(1):22-7.

33 Miller CT, Solomon SE, Varni SE, Hodge JJ, Knapp FA, Bunn JY. A transactional approach to relationships over time between perceived HIV stigma and the psychological and physical well-being of people with HIV. Soc Sci Med. 2016;162:97-105.

34 Mo PK, Lau JT, Yu X, Gu J. A model of associative stigma on depression and anxiety among children of HIV-infected parents in China. AIDS Behav. 2015;19(1):50-9.

35 Nyblade L, Mbuya-Brown RJ, Ezekiel MJ, Addo NA, Sabasaba AN, Atuahene K, et al. A total facility approach to reducing HIV stigma in health facilities: implementation process and lessons learned. AIDS. 2020;34 Suppl 1:S93-S102.

36 Pantelic M, Boyes M, Cluver L, Meinck F. HIV, violence, blame and shame: pathways of risk to internalized HIV stigma among South African adolescents living with HIV. J Int AIDS Soc. 2017;20(1):21771.

37 Pescosolido BA, Martin JK. The Stigma Complex. Annu Rev Sociol. 2015;41:87-116.

38 Pham HN, Protsiv M, Larsson M, Ho HT, de Vries DH, Thorson A. Stigma, an important source of dissatisfaction of health workers in HIV response in Vietnam: a qualitative study. BMC Health Serv Res. 2012;12:474.

39 Prati G, Zani B, Pietrantoni L, Scudiero D, Perone P, Cosmaro L, et al. The role of knowing someone living with HIV/AIDS and HIV disclosure in the HIV stigma framework: A Bayesian mediation analysis. Quality & Quantity: International Journal of Methodology. 2016;50(2):637-51.

40 Pulerwitz J, Oanh KT, Akinwolemiwa D, Ashburn K, Nyblade L. Improving hospital-based quality of care by reducing HIV-related stigma: evaluation results from Vietnam. AIDS Behav. 2015;19(2):246-56.

41 Rao D, Feldman BJ, Fredericksen RJ, Crane PK, Simoni JM, Kitahata MM, et al. A structural equation model of HIV-related stigma, depressive symptoms, and medication adherence. AIDS Behav. 2012;16(3):711-6.

42 Relf MV, Pan W, Edmonds A, Ramirez C, Amarasekara S, Adimora AA. Discrimination, Medical Distrust, Stigma, Depressive Symptoms, Antiretroviral Medication Adherence, Engagement in Care, and Quality of Life Among Women Living With HIV in North Carolina: A Mediated Structural Equation Model. J Acquir Immune Defic Syndr. 2019;81(3):328-35.

43 Rice WS, Crockett KB, Mugavero MJ, Raper JL, Atkins GC, Turan B. Association Between Internalized HIV-Related Stigma and HIV Care Visit Adherence. J Acquir Immune Defic Syndr. 2017;76(5):482-7.

44 Sen S, Aguilar JP, Petty M. An ecological framework for understanding HIV- and AIDS-related stigma among Asian American and Pacific Islander men who have sex with men living in the USA. Cult Health Sex. 2021;23(1):85-97.

45 Shin SS, Carpenter CL, Ekstrand ML, Yadav K, Shah SV, Ramakrishnan P, et al. Household food insecurity as mediator of the association between internalized stigma and opportunistic infections. AIDS and Behavior. 2018;22(12):3897-904.

46 Slater LZ, Moneyham L, Vance DE, Raper JL, Mugavero MJ, Childs G. The multiple stigma experience and quality of life in older gay men with HIV. J Assoc Nurses AIDS Care. 2015;26(1):24-35.

47 Stangl AL, Earnshaw VA, Logie CH, van Brakel W, L CS, Barré I, et al. The Health Stigma and Discrimination Framework: a global, crosscutting framework to inform research, intervention development, and policy on health-related stigmas. BMC Med. 2019;17(1):31.

48 Stevens ME, Parsons JA, Read SE, Nixon SA. The conceptualization of stigma within a rehabilitation framework using HIV as an example. Disabil Rehabil. 2019;41(2):235-43.

49 Steward WT, Herek GM, Ramakrishna J, Bharat S, Chandy S, Wrubel J, et al. HIV-related stigma: adapting a theoretical framework for use in India. Soc Sci Med. 2008;67(8):1225-35.

50 Thapa S, Hannes K, Cargo M, Buve A, Aro AR, Mathei C. Building a Conceptual Framework to Study the Effect of HIV Stigma-Reduction Intervention Strategies on HIV Test Uptake: A Scoping Review. J Assoc Nurses AIDS Care. 2017;28(4):545-60.

51 Thi MD, Brickley DB, Vinh DT, Colby DJ, Sohn AH, Trung NQ, et al. A qualitative study of stigma and discrimination against people living with HIV in Ho Chi Minh City, Vietnam. AIDS Behav. 2008;12(4 Suppl):S63-70.

52 Thrasher AD, Earp JA, Golin CE, Zimmer CR. Discrimination, distrust, and racial/ethnic disparities in antiretroviral therapy adherence among a national sample of HIV-infected patients. J Acquir Immune Defic Syndr. 2008;49(1):84-93.

53 Tsai AC, Bangsberg DR, Kegeles SM, Katz IT, Haberer JE, Muzoora C, et al. Internalized stigma, social distance, and disclosure of HIV seropositivity in rural Uganda. Ann Behav Med. 2013;46(3):285-94.

54 Turan B, Budhwani H, Fazeli PL, Browning WR, Raper JL, Mugavero MJ, et al. How Does Stigma Affect People Living with HIV? The Mediating Roles of Internalized and Anticipated HIV Stigma in the Effects of Perceived Community Stigma on Health and Psychosocial Outcomes. AIDS Behav. 2017;21(1):283-91.

55 Turan B, Hatcher AM, Weiser SD, Johnson MO, Rice WS, Turan JM. Framing Mechanisms Linking HIV-Related Stigma, Adherence to Treatment, and Health Outcomes. Am J Public Health. 2017;107(6):863-9.

56 Turan B, Smith W, Cohen MH, Wilson TE, Adimora AA, Merenstein D, et al. Mechanisms for the Negative Effects of Internalized HIV-Related Stigma on Antiretroviral Therapy Adherence in Women: The Mediating Roles of Social Isolation and Depression. J Acquir Immune Defic Syndr. 2016;72(2):198-205.

57 UNAIDS. Confronting discrimination: Overcoming HIV-related stigma and discrimination in healthcare settings and beyond. 2017.

58 UNAIDS. Confronting discrimination: Overcoming HIV-related stigma and discrimination in healthcare settings and beyond. Geneva, Switzerland: UNAIDS Joint United Nations Programme on HIV/AIDS; 2017b.

59 UNAIDS. Confronting discrimination: Overcoming HIV-related stigma and discrimination in healthcare settings and beyond. Geneva, Switzerland: UNAIDS Joint United Nations Programme on HIV/AIDS; 2017c.

60 UNAIDS. Confronting discrimination: Overcoming HIV-related stigma and discrimination in healthcare settings and beyond. Geneva, Switzerland: UNAIDS Joint United Nations Programme on HIV/AIDS; 2017d.

61 Vyavaharkar M, Moneyham L, Corwin S, Saunders R, Annang L, Tavakoli A. Relationships between stigma, social support, and depression in HIV-infected African American women living in the rural Southeastern United States. J Assoc Nurses AIDS Care. 2010;21(2):144-52.

62 Wardell JD, Shuper PA, Rourke SB, Hendershot CS. Stigma, coping, and alcohol use severity among people living with HIV: A prospective analysis of bidirectional and mediated associations. Ann Behav Med. 2018;52(9):762-72.

63 Watt MH, Knettel BA, Knippler ET, Kisigo G, Ngocho JS, Renju J, et al. The development of Maisha, a video-assisted counseling intervention to address HIV stigma at entry into antenatal care in Tanzania. Eval Program Plann. 2020;83:101859.

64 Williams LD. Understanding the relationships among HIV/AIDS-related stigma, health service utilization, and HIV prevalence and incidence in Sub-Saharan Africa: a multi-level theoretical perspective. Am J Community Psychol. 2014;53(1-2):146-58.

65 Williams LD, Aber JL. Using a multi-level framework to test empirical relationships among HIV/AIDS-related stigma, health service barriers, and HIV outcomes in KwaZulu‑Natal, South Africa. AIDS and Behavior. 2020;24(1):81-94.

66 Woodgate RL, Zurba M, Tennent P, Cochrane C, Payne M, Mignone J. "People try and label me as someone I'm not": The social ecology of Indigenous people living with HIV, stigma, and discrimination in Manitoba, Canada. Soc Sci Med. 2017;194:17-24.

67 Yang LH, Kleinman A. 'Face' and the embodiment of stigma in China: the cases of schizophrenia and AIDS. Soc Sci Med. 2008;67(3):398-408.

68 Yu NX, Zhang J, Chan CL. Health Care Neglect, Perceived Discrimination, and Dignity-Related Distress Among Chinese Patients With HIV. AIDS Educ Prev. 2016;28(1):90-102.

69 Zang C, Guida J, Sun Y, Liu H. Collectivism culture, HIV stigma and social network support in Anhui, China: a path analytic model. AIDS Patient Care STDS. 2014;28(8):452-8.

70 Aggarwal S, Lee DH, Minteer WB, Fenning RT, Raja SK, Bernstein ME, et al. Another Generation of Stigma? Assessing Healthcare Student Perceptions of HIV-Positive Patients in Mwanza, Tanzania. AIDS Patient Care STDS. 2017;31(2):87-95.

71 Ahmadi K, Reidpath DD, Allotey P, Hassali MAA. A latent trait approach to measuring HIV/AIDS related stigma in healthcare professionals: application of mokken scaling technique. BMC Med Educ. 2016;16:155.

72 Biemba G, Menda DM, Siame Y, Sichinga KS, Macleod W. Towards an AIDS free generation: Is stigma still an issue in Zambia? Results from a legal environment assessment of the HIV/AIDS/TB program of the Churches Health Association of Zambia. J Public Health Afr. 2019;10(2):1010.

73 Bogart LM, Landrine H, Galvan FH, Wagner GJ, Klein DJ. Perceived discrimination and physical health among HIV-positive Black and Latino men who have sex with men. AIDS Behav. 2013;17(4):1431-41.

74 Brittain K, Mellins CA, Phillips T, Zerbe A, Abrams EJ, Myer L, et al. Social Support, Stigma and Antenatal Depression Among HIV-Infected Pregnant Women in South Africa. AIDS Behav. 2017;21(1):274-82.

75 Dos Santos MM, Kruger P, Mellors SE, Wolvaardt G, van der Ryst E. An exploratory survey measuring stigma and discrimination experienced by people living with HIV/AIDS in South Africa: the People Living with HIV Stigma Index. BMC Public Health. 2014;14:80.

76 Earnshaw VA, Smith LR, Shuper PA, Fisher WA, Cornman DH, Fisher JD. HIV stigma and unprotected sex among PLWH in KwaZulu-Natal, South Africa: a longitudinal exploration of mediating mechanisms. AIDS Care. 2014;26(12):1506-13.

77 Eaton LA, Allen A, Maksut JL, Earnshaw V, Watson RJ, Kalichman SC. HIV microaggressions: a novel measure of stigma-related experiences among people living with HIV. J Behav Med. 2020;43(1):34-43.

78 Feyissa GT, Abebe L, Girma E, Woldie M. Validation of an HIV-related stigma scale among health care providers in a resource-poor Ethiopian setting. J Multidiscip Healthc. 2012;5:97-113.

79 Franke MF, Muñoz M, Finnegan K, Zeladita J, Sebastian JL, Bayona JN, et al. Validation and abbreviation of an HIV stigma scale in an adult spanish-speaking population in urban Peru. AIDS Behav. 2010;14(1):189-99.

80 Franke MF, Nelson AK, Muñoz M, Cruz JS, Atwood S, Lecca L, et al. Validation of 2 Spanish-Language Scales to Assess HIV-Related Stigma in Communities. J Int Assoc Provid AIDS Care. 2015;14(6):527-35.

81 Friedland BA, Gottert A, Hows J, Baral SD, Sprague L, Nyblade L, et al. The People Living with HIV Stigma Index 2.0: generating critical evidence for change worldwide. Aids. 2020;34 Suppl 1:S5-s18.

82 Global Network Of People Living with HIV (GNP+). The People Living with HIV Stigma Index 2008 [Available from: <https://www.stigmaindex.org/>.

83 Health Policy Project. Measuring HIV Stigma and Discrimination Among Health Facility Staff: Monitoring Tool for Global Indicators. Washington, DC: Futures Group, Health Policy Project; 2015.

84 Hernansaiz-Garrido H, Alonso-Tapia J. Internalized HIV Stigma and Disclosure Concerns: Development and Validation of Two Scales in Spanish-Speaking Populations. AIDS Behav. 2017;21(1):93-105.

85 Hojilla JC, Santiago-Rodriguez EI, Sterling S, Williams EC, Leyden W, Hare CB, et al. HIV Stigma and Its Associations with Longitudinal Health Outcomes Among Persons Living with HIV with a History of Unhealthy Alcohol Use. AIDS Behav. 2020;25(1):215-24.

86 Jimenez JC, Puig M, Ramos JC, Morales M, Asencio G, Sala AC, et al. Measuring HIV felt stigma: a culturally adapted scale targeting PLWHA in Puerto Rico. AIDS Care. 2010;22(11):1314-22.

87 Kagiura F, Fujii T, Kihana N, Maruyama E, Shimoji Y, Kakehashi M. Brief HIV stigma scale for Japanese people living with HIV: validation and restructuring using questionnaire survey data. AIDS Care. 2020;32(sup1):1-9.

88 Kalichman SC, Simbayi LC, Cloete A, Mthembu PP, Mkhonta RN, Ginindza T. Measuring AIDS stigmas in people living with HIV/AIDS: the Internalized AIDS-Related Stigma Scale. AIDS Care. 2009;21(1):87-93.

89 Kamitani E, Chen JL, Portillo C, Tokumoto J, Dawson-Rose C. Shortened and Culturally Appropriate HIV Stigma Scale for Asians Living with HIV in the United States: Psychometric Analysis. J Assoc Nurses AIDS Care. 2018;29(4):560-9.

90 Li J, Assanangkornchai S, Lu L, Jia M, McNeil EB, You J, et al. Development of internalized and personal stigma among patients with and without HIV infection and occupational stigma among health care providers in Southern China. Patient Prefer Adherence. 2016;10:2309-20.

91 Molina Y, Ramirez-Valles J. HIV/AIDS stigma: measurement and relationships to psycho-behavioral factors in Latino gay/bisexual men and transgender women. AIDS Care. 2013;25(12):1559-68.

92 Neuman M, Obermeyer CM, Group MS. Experiences of stigma, discrimination, care and support among people living with HIV: a four country study. AIDS Behav. 2013;17(5):1796-808.

93 Nyblade L, Jain A, Benkirane M, Li L, Lohiniva AL, McLean R, et al. A brief, standardized tool for measuring HIV-related stigma among health facility staff: results of field testing in China, Dominica, Egypt, Kenya, Puerto Rico and St. Christopher & Nevis. J Int AIDS Soc. 2013;16(3 Suppl 2):18718.

94 Phillips KD. Conceptual development of an instrument to measure the internalized stigma of AIDS based on the Roy adaptation model. Nurs Sci Q. 2011;24(4):306-10.

95 Phillips KD, Moneyham L, Tavakoli A. Development of an instrument to measure internalized stigma in those with HIV/AIDS. Issues Ment Health Nurs. 2011;32(6):359-66.

96 Pourmarzi D, Khoramirad A, Ahmari Tehran H, Abedini Z. Validity and Reliability of Persian Version of HIV/AIDS Related Stigma Scale for People Living With HIV/AIDS in Iran. J Family Reprod Health. 2015;9(4):164-71.

97 Rao D, Molina Y, Lambert N, Cohn SE. Assessing Stigma among African Americans Living with HIV. Stigma Health. 2016;1(3):146-55.

98 Reinius M, Wettergren L, Wiklander M, Svedhem V, Ekström AM, Eriksson LE. Development of a 12-item short version of the HIV stigma scale. Health Qual Life Outcomes. 2017;15(1):115.

99 Rutledge SE, Whyte J, Abell N, Brown KM, Cesnales NI. Measuring stigma among health care and social service providers: The HIV/AIDS Provider Stigma Inventory. AIDS Patient Care STDS. 2011;25(11):673-82.

100 Sayles JN, Hays RD, Sarkisian CA, Mahajan AP, Spritzer KL, Cunningham WE. Development and psychometric assessment of a multidimensional measure of internalized HIV stigma in a sample of HIV-positive adults. AIDS Behav. 2008;12(5):748-58.

101 See LC, Shen YM, Chen CL, Huang TM, Huang YH, Huang HC, et al. Professional attitude of health care workers toward serving HIV/AIDS patients and drug users: questionnaire design and evaluation of reliability and validity. AIDS Care. 2011;23(11):1448-55.

102 Smith EA, Miller JA, Newsome V, Sofolahan YA, Airhihenbuwa CO. Measuring HIV/AIDS-Related Stigma Across South Africa: A Versatile and Multidimensional Scale. Health Educ Behav. 2014;41(4):387-91.

103 Srithanaviboonchai K, Stockton M, Pudpong N, Chariyalertsak S, Prakongsai P, Chariyalertsak C, et al. Building the evidence base for stigma and discrimination-reduction programming in Thailand: development of tools to measure healthcare stigma and discrimination. BMC Public Health. 2017;17(1):245.

104 Stangl AL, Lilleston P, Mathema H, Pliakas T, Krishnaratne S, Sievwright K, et al. Development of parallel measures to assess HIV stigma and discrimination among people living with HIV, community members and health workers in the HPTN 071 (PopART) trial in Zambia and South Africa. J Int AIDS Soc. 2019;22(12):e25421.

105 Tyer-Viola LA, Duffy ME. The Pregnant Women with HIV Attitude Scale: Development and initial psychometric evaluation. Journal of Advanced Nursing. 2010;66(8):1852-63.

106 UNAIDS, Joint United Nations Programme on HIV/AIDS. Global AIDS Monitoring 2021: Indicators for monitoring the 2016 Political Declaration on Ending AIDS. Geneva, Switzerland; 2020.

107 Uys LR, Holzemer WL, Chirwa ML, Dlamini PS, Greeff M, Kohi TW, et al. The development and validation of the HIV/AIDS Stigma Instrument - Nurse (HASI-N). AIDS Care. 2009;21(2):150-9.

108 Varas-Díaz N, Neilands TB. Development and validation of a culturally appropriate HIV/AIDS Stigma Scale for Puerto Rican health professionals in training. AIDS Care. 2009;21(10):1259-70.

109 Visser MJ, Kershaw T, Makin JD, Forsyth BW. Development of parallel scales to measure HIV-related stigma. AIDS Behav. 2008;12(5):759-71.

110 Vreeman RC, Scanlon ML, Tu W, Slaven J, McAteer C, Aluoch J, et al. Validation of an HIV/AIDS Stigma Measure for Children Living with HIV and Their Families. J Int Assoc Provid AIDS Care. 2019;18:2325958219880570.

111 Wagner AC, Hart TA, McShane KE, Margolese S, Girard TA. Health care provider attitudes and beliefs about people living with HIV: Initial validation of the Health Care Provider HIV/AIDS Stigma Scale (HPASS). AIDS Behav. 2014;18(12):2397-408.

112 Wagner AC, MacLean R. Preliminary investigation of the STBBI Stigma Scale: Description and pilot results. Can J Public Health. 2017;108(4):e368-e73.

113 Wiklander M, Rydström LL, Ygge BM, Navér L, Wettergren L, Eriksson LE. Psychometric properties of a short version of the HIV stigma scale, adapted for children with HIV infection. Health Qual Life Outcomes. 2013;11:195.

114 Windsor LC, Benoit E, Ream GL, Forenza B. The provider perception inventory: psychometrics of a scale designed to measure provider stigma about HIV, substance abuse, and MSM behavior. AIDS Care. 2013;25(5):586-91.

115 Woldetsadik MA, Goggin K, Staggs VS, Wanyenze RK, Beyeza-Kashesya J, Mindry D, et al. Safer conception methods and counseling: Psychometric evaluation of new measures of attitudes and beliefs among HIV clients and providers. AIDS and Behavior. 2016;20(6):1370-81.

116 Wouters E, Masquillier C, Sommerland N, Engelbrecht M, Van Rensburg AJ, Kigozi G, et al. Measuring HIV- and TB-related stigma among health care workers in South Africa: a validation and reliability study. Int J Tuberc Lung Dis. 2017;21(11):19-25.

117 Xie H, Yu H, Watson R, Wen J, Xiao L, Yan M, et al. Cross-Cultural Validation of the Health Care Provider HIV/AIDS Stigma Scale (HPASS) in China. AIDS Behav. 2019;23(4):1048-56.

118 Zelaya CE, Sivaram S, Johnson SC, Srikrishnan AK, Suniti S, Celentano DD. Measurement of self, experienced, and perceived HIV/AIDS stigma using parallel scales in Chennai, India. AIDS Care. 2012;24(7):846-55.

119 Carr D, Kidd R, Fitzgerald M, Nyblade L. Acheiving a Stigma-free Health Facility and HIV Services: Resources for Administrators. Washington, DC: Futures Group, Health Policy Project; 2015.

120 Chan BT, Pradeep A, Chandrasekaran E, Prasad L, Murugesan V, Kumarasamy N, et al. Reliability, Validity, and Factor Structure of the Internalized AIDS-Related Stigma Scale in Southern India. J Int Assoc Provid AIDS Care. 2019;18:2325958219831025.

121 Chinouya M, Hildreth A, Goodall D, Aspinall P, Hudson A. Migrants and HIV stigma: findings from the Stigma Index Study (UK). Health Soc Care Community. 2017;25(1):35-42.

122 Earnshaw VA, Chaudoir SR. From conceptualizing to measuring HIV stigma: a review of HIV stigma mechanism measures. AIDS Behav. 2009;13(6):1160-77.

123 Geibel S, Gottert A, Friedland BA, Jeremiah K, McClair TL, Mallouris C, et al. Internalized stigma among people living with HIV: assessing the Internalized AIDS-Related Stigma Scale in four countries. Aids. 2020;34 Suppl 1:S33-s42.

124 Global Network Of People Living with HIV (GNP+). The People Living with HIV Stigma Index 2.0 [cited Handsearching. Available from: <https://www.stigmaindex.org/about-the-stigma-index/the-people-living-with-hiv-stigma-index-2-0/>.

125 Goodin BR, Owens MA, White DM, Strath LJ, Gonzalez C, Rainey RL, et al. Intersectional health-related stigma in persons living with HIV and chronic pain: implications for depressive symptoms. AIDS Care. 2018;30(sup2):66-73.

126 Health Policy Project. Comprehensive Package for Reducing Stigma and Discrimination in Health Facilities [cited Search on Health Policy Plus_January 27, 2021. Available from: <https://www.healthpolicyproject.com/index.cfm?id=stigmapackage>.

127 International Center for Research on Women (ICRW), STRIVE. A global HIV stigma reduction framework adapted and implemented in five settings in India: Summary report. 2013.

128 Jain A, Carr D, Nyblade L. Measuring HIV Stigma and Discrimination Among Health Facility Staff: Standardized Brief Questionnaire User Guide. Washington, DC: Futures Group, Health Policy Project; 2015.

129 Joint United Nations Programme on HIV/AIDS (UNAIDS). People Living with HIV Stigma Index: Asia Pacific Regional Analysis. 2011. Contract No.: February 4, .

130 Luz PM, Torres TS, Almeida-Brasil CC, Marins LMS, Bezerra DRB, Veloso VG, et al. Translation and validation of the Short HIV Stigma scale in Brazilian Portuguese. Health Qual Life Outcomes. 2020;18(1):322.

131 Misir P. Structuration Theory: A Conceptual Framework for HIV/AIDS Stigma. J Int Assoc Provid AIDS Care. 2015;14(4):328-34.

132 Peltzer K, Ramlagan S. Perceived stigma among patients receiving antiretroviral therapy: A prospective study in KwaZulu-Natal, South Africa. AIDS Care. 2011;23(1):60-8.

133 Rao D, Andrasik M, Acharya X, Simoni JM. Internalized stigma among African Americans living with HIV: Preliminary scale development based on qualitative data. In: Liamputtong P, editor. Stigma, discrimination and living with HIV/AIDS: A cross-cultural perspective: Springer Science + Business Media, New York, NY; 2013. p. 155-68, Chapter xxiv, 415 Pages.

134 Reinius M, Wiklander M, Wettergren L, Svedhem V, Eriksson LE. The Relationship Between Stigma and Health-Related Quality of Life in People Living with HIV Who Have Full Access to Antiretroviral Treatment: An Assessment of Earnshaw and Chaudoir's HIV Stigma Framework Using Empirical Data. AIDS Behav. 2018;22(12):3795-806.

135 Rosenburg N, Taliaferro D, Ercole P. HIV-related stigma among nursing students in Cameroon. J Assoc Nurses AIDS Care. 2012;23(2):170-6.

136 RTI International. Laura Nyblade presents at White House meeting on HIV stigma 2016 [cited Hand searching. Available from: <https://www.rti.org/news/laura-nyblade-presents-white-house-meeting-hiv-stigma>.

137 Rydström LL, Wiklander M, Ygge BM, Navér L, Eriksson LE. Legal guardians understand how children with the human immunodeficiency virus perceive quality of life and stigma. Acta Paediatr. 2015;104(9):940-7.

138 Stangl A, Barre I. STRIVE Impact Case Study: Stigma framework and measurement. Washington D.C., USA: International Center for Research on Women,; 2017.

139 Stangl A, Brady L, Fritz K. STRIVE Technical Brief: Measuring HIV stigma and discrimination. Washington, D.C.: International Center for Research on Women; 2012 (Updated in 2018).

140 Stangl A, Go V, al. e. Enabling the scale-up of efforts to reduce HIV stigma and discrimination: A new framework to inform programme implementation and measurement. XVIII International AIDS Conference; Vienna. Handsearching2010.

141 Steward WT, Chandy S, Singh G, Panicker ST, Osmand TA, Heylen E, et al. Depression is not an inevitable outcome of disclosure avoidance: HIV stigma and mental health in a cohort of HIV-infected individuals from Southern India. Psychol Health Med. 2011;16(1):74-85.

142 Tsai AC, Weiser SD, Steward WT, Mukiibi NF, Kawuma A, Kembabazi A, et al. Evidence for the reliability and validity of the internalized AIDS-related stigma scale in rural Uganda. AIDS Behav. 2013;17(1):427-33.

143 UNAIDS, World Health Organization. About the data 2018 [cited Hand searching. Available from: <http://lawsandpolicies.unaids.org/about?lan=en>.

144 Valle A, Treviño AC, Zambrano FF, Urriola KE, Sánchez LA, Elizondo JE. Perceived HIV-Associated Stigma among HIV-Seropositive Men: Psychometric Study of HIV Stigma Scale. Front Public Health. 2015;3:171.

145 Wouters E, Rau A, Engelbrecht M, Uebel K, Siegel J, Masquillier C, et al. The Development and Piloting of Parallel Scales Measuring External and Internal HIV and Tuberculosis Stigma Among Healthcare Workers in the Free State Province, South Africa. Clin Infect Dis. 2016;62 Suppl 3(Suppl 3):S244-54.

**Appendix Table 1. Evidence table for frameworks**

| **ID**  **Framework title and type**  **Domain**  **Link** | **Scope and Aim/Purpose**  **Target** | **Stigma subtype, definition**  **Discrimination subtype, definition** | **Components** | **Summary** |
| --- | --- | --- | --- | --- |
| Boyes, 2020 [1]  **Framework name:** NA  Empirical model  **Terminology:** Stigma  **Domain:** Internalized stigma  https://www.ncbi.nlm.nih.gov/pubmed/32871672 | **Scope:** Mental health, bullying, and internalized stigma among South African adolescents  **Aim/Purpose:** To test longitudinal associations between bullying victimization, internalized stigma, and mental health among adolescents living with HIV  **Framework HIV target:** South African adolescents living with HIV | **Subtype:** Internalized  **Definition:** HIV stigma = negative attitudes and prejudice directed at people living with HIV  **Framework specific to HIV:** Yes | Bullying victimization -> internalized stigma  Bullying victimization -> depression scores  Bullying victimization -> post traumatic stress scores  Bullying victimization -> anxiety scores  Internalized stigma -> depression scores  Internalized stigma- > post traumatic stress scores  Internalized stigma -> anxiety scores | Bullying victimization is associated with internalized stigma, which in turn predicts psychological symptoms over time. |
| Casale, 2019 [2]  **Framework name:** NA  Empirical model  **Terminology:** Stigma  **Domain:** Internalized stigma  https://www.ncbi.nlm.nih.gov/pubmed/30368074 | **Scope:** Social support buffering impact of stigma among South African adolescents living with HIV  **Aim/Purpose:** To test a moderated mediation model for potential effects of stigma on suicidal ideation and attempts, and stress-buffering effects of social support resources on depression and suicidal ideation and attempts  **Framework HIV target:** South African adolescents living with HIV | **Subtype:** Internalized, anticipated, and enacted  **Definition:** NA  **Framework specific to HIV:** Yes | Stigma -> depression  Stigma -> suicidal thoughts and behavior  Depression -> suicidal thoughts and behavior  MODERATORS AND INTERACTIONS:  Stigma x perceived  Stigma x support group  Depression x perceived  Depression x support group | Stigma was a risk factor for depression and for suicidal thoughts and behavior. Only perceived support availability was directly associated with less depression, however, both perceived support availability and support group participation contributed to the overall stress-buffering effects moderating the direct and indirect relationships between stigma and suicidal thoughts and behavior. |
| Chan, 2019 [3]  **Framework name:** NA  Empirical model  **Terminology:** Stigma  **Domain:** Internalized stigma  https://www.ncbi.nlm.nih.gov/pubmed/31690214 | **Scope:** HIV stigma in public and within the gay community; mental and social health of men who have sex with men living with HIV  **Aim/Purpose:** To propose and examine a mediation model to understand how HIV stigma adversely affects mental and social health  **Framework HIV target:** Men who have sex with men living with HIV | **Subtype:** NA  **Definition:** NA  **Framework specific to HIV:** Yes | HIV stigma from the public  HIV stigma within the gay community  COGNITIVE PROCESS  Negative self concept  REGULATORY PROCESS  Maladaptive coding  INTERPERSONAL PROCESS  Peer isolation  Mental health  Social health | HIV stigma within the gay community represents a significant risk factor and shows that the psychological mediation framework is a useful conceptual tool for explaining how stigma influences health outcomes in this population; findings lend support to the cognitive, regulatory, and interpersonal processes through which HIV stigma within the gay community undermines mental and social health among men who have sex with men living with HIV. |
| Christopoulos, 2020 [4]  **Framework name:** NA  Empirical model  **Terminology:** Stigma  **Domain:** Internalized stigma  https://www.ncbi.nlm.nih.gov/pubmed/32769764 | **Scope:** Internalized HIV stigma predicting viral load  **Aim/Purpose:** To examine the prospective association between internalized HIV stigma and unsuppressed viral load  **Framework HIV target:** U.S. HIV patients | **Subtype:** Internalized  **Definition:** NA  **Framework specific to HIV:** Yes | Depressive symptoms-> ART adherence  Depressive symptoms -> viral load >200 copies/ml  Internalized HIV stigma -> depressive symptoms  Internalized HIV stigma -> ART adherence  Internalized HIV stigma- > viral load >200 copies/ml  ART adherence -> viral load >200 copies/ml | Longitudinal sequential path model of the association between internalized HIV stigma and unsuppressed viral load through the mediators of depressive symptoms and antiretroviral adherence. |
| Clum, 2009 [5]  **Framework name:** NA  Empirical model  **Terminology:** Stigma  **Domain:** Internalized stigma  https://www.ncbi.nlm.nih.gov/pubmed/20024724 | **Scope:** Mediators of HIV-related stigma and risk behavior in HIV infected young women  **Aim/Purpose:** To identify pathways between HIV-related stigma and risk behavior in young HIV positive women  **Framework HIV target:** Young HIV positive women | **Subtype:** NA  **Definition:** Stigma = asocial identity that is devalued in a particular social context; a ‘‘spoiled identity"  **Framework specific to HIV:** Yes | HIV Stigma  Depression  Social support  Risk class membership | Depression was a significant mediator between HIV-related stigma and risk behavior. |
| Cluver, 2013 [6]  **Framework name:** NA  Empirical model  **Terminology:** Stigma  **Domain:** Internalized stigma  https://www.ncbi.nlm.nih.gov/pubmed/23631794 | **Scope:** Parental AIDs/AIDS orphanhood and child psychological, educational and sexual risk  **Aim/Purpose:** To develop an empirically-based theoretical model of interactive relationships between parental or primary caregiver AIDS-illness, AIDS-orphanhood and predicted intervening factors associated with children’s psychological distress, educational access and sexual health  **Framework HIV target:** Children orphaned by AIDS; children with a parental or primary caregiver that has AIDS-illness | **Subtype:** NA  **Definition:** NA  **Framework specific to HIV:** Yes | AIDS-orphan -> poverty  AIDS-orphan -> stigma  AIDS-sick parent -> poverty  AIDS-sick parent -> parental disability  AIDS-sick parent -> community violence  AIDS-sick parent -> stigma  AIDS-sick parent -> abuse  Poverty -> stigma  Poverty -> abuse  Poverty -> community violence  Poverty -> unable to afford fees or tuition  Parental disability -> stigma  Abuse -> psychological distress  Community violence -> abuse  Community violence -> sexual health risks  Community violence -> unable to afford fees or tuition  Stigma -> abuse  Psychological distress -> sexual health risks  Psychological distress -> education risks  Sexual health risks -> pregnancy  Pregnancy -> education risks  Sexual health risks -> education risks | Neither AIDS-orphanhood nor parental AIDS-illness were directly associated with psychological distress, educational access, or sexual health. Instead, significant indirect effects of AIDS orphanhood and parental AIDS-illness were obtained on all measured outcomes. Child psychological, educational and sexual health risks share a common set of intervening variables including parental disability, poverty, community violence, stigma, and child abuse that together comprise chain effects. |
| Colbert, 2010 [7]  **Framework name:** NA  Empirical model  **Terminology:** Stigma  **Domain:** Internalized stigma  https://www.ncbi.nlm.nih.gov/pubmed/20116295 | **Scope:** HIV stigma among men and women living with HIV  **Aim/Purpose:** To examine how social support and health status are related to HIV stigma, after controlling for specific socio-demographic factors, and how these relationships differ between men and women living with HIV  **Framework HIV target:** Men and women living with HIV | **Subtype:** NA  **Definition:** Stigma = attribute that is deeply discrediting  **Framework specific to HIV:** Yes | Covariates  Race  IDU history  Exposure: gay/bisexual  Exposure: IV drug use  Exposure: other/unknown  Physical HIV health  Social support  HIV stigma | The framework offers insight into understanding the relationships among gender, health status, social support, and HIV-related stigma. |
| Crockett, 2019 [8]  **Framework name:** NA  Empirical model  **Terminology:** Stigma,Discrimination  **Domain:** Internalized stigma  https://www.ncbi.nlm.nih.gov/pubmed/30773914 | **Scope:** HIV-related discrimination and the perceived impact of those experiences  **Aim/Purpose:** To investigate the role of stress associated with events of HIV-related discrimination on internalised HIV stigma, as well as the downstream effects on depressive symptoms and alcohol use severity  **Framework HIV target:** People living with HIV at a HIV clinic in the southeastern U.S. | **Subtype:** Internalized  **Definition:** HIV stigma = social devaluation and discrediting of people living with HIV  Internalized stigma = extent to which PLWH apply negative beliefs and feelings (e.g., shame, disgust) about HIV to themselves  Enacted stigma = encompasses discrimination, prejudice, and stereotypes that are experienced by people living with HIV  **Framework specific to HIV:** Yes | HIV-related discrimination -> stress related to discrimination -> internalized HIV stigma -> depressive symptoms  HIV-related discrimination -> internalized HIV stigma  HIV-related discrimination -> depressive symptoms  Stress related to discrimination - depressive symptoms | In serial mediation models, HIV-related discrimination was indirectly associated with both depressive symptoms and alcohol use severity through its associations with stress and internalized HIV stigma. |
| Deacon, 2005 [11]  **Framework name:** How Different Kinds of Stigma and Discrimination Relate to Each Other  Conceptual model  **Terminology:** Stigma,Discrimination  **Domain:** Internalized stigma  https://www.hsrcpress.ac.za/books/understanding-hiv-aids-stigma | **Scope:** Relation of stigma and discrimination  **Aim/Purpose:** To illustrate the relationship between our proposed definition of disease symptom and the current psychological literature on stigma  **Framework HIV target:** Stigmatized person or associates | **Subtype:** Expressed, enacted, internalized  **Definition:** Disease stigma = ideology that claims that people with a specific disease are different from ‘normal’ society, more than simply through their infection with a disease agent  **Framework specific to HIV:** Broader | What people BELIEVE  Stigma as ideology (attitudes and beliefs)  What people SAY  Expressed stigma  What people DO  Discrimination (enacted stigma)  Experience of stigmatization  Perception and expectation of stigmatization and discrimination  What people BELIEVE  Internalization of stigmatizing ideas  Cognitive restructuring  What people SAY  Silence and/or protest  What people DO  Withdrawal  Resistance to stigma | The 3 columns represent what people believe (stigma), what they say, and what they do (discrimination, withdrawal, activism). The rows represent what stigmatizing people believe, say or do, and what stigmatized people experience, believe, say and do. Responses to stigmatization by people living with HIV/AIDS (self-stigmatization and perceived stigma) are not included in our definition of stigma per se. Internalization of stigma may result from the same cognitive and emotional processes that lead to stigmatization, but crucially, it is imposed on the self rather than the ‘other.’ |
| Diiorio, 2009 [12]  **Framework name:** Model of Adherence  Empirical model  **Terminology:** Stigma  **Domain:** Internalized stigma  https://www.ncbi.nlm.nih.gov/pubmed/17978868 | **Scope:** Stigma and adherence to antiretroviral medication regimens  **Aim/Purpose:** To test a psychosocial model of medication adherence among people taking antiretroviral medications  **Framework HIV target:** People living with HIV | **Subtype:** Personal  **Definition:** NA  **Framework specific to HIV:** Yes | Stigma -> efficacy  Satisfaction -> efficacy  Support -> efficacy  Decision-making -> satisfaction  Support -> depression  Efficacy -> adherence  Depression -> adherence  Difficult life circumstances -> depression | These findings provide evidence to reinforce the belief that medication taking behaviors are affected by a complex set of interactions among psychosocial variables and provide direction for adherence interventions. |
| Earnshaw, 2013 [13]  **Framework name:** Stigma and HIV Disparities Model  Conceptual model  **Terminology:** Stigma,Other : Disparities  **Domain:** Internalized stigma  https://www.ncbi.nlm.nih.gov/pubmed/23688090 | **Scope:** Societal stigma related to race and ethnicity  **Aim/Purpose:** To describe how societal stigma related to race and ethnicity is associated with racial/ethnic HIV disparities via its manifestations at the structural level as well as the individual level among perceivers  **Framework HIV target:** Racial/ethnic minorities at risk of and living with HIV | **Subtype:** Societal, perceived, anticipated, internalized, multiple stigmas/intersectionality  **Definition:** Societal stigma = social devaluation and discrediting associated with a personal attribute, mark, or characteristic such as race, ethnicity, or sexual minority orientation  Perceived stigma = assessment of experiencing prejudice, stereotypes, and/or discrimination from others in the past  Anticipated stigma = involves expectations of such bias in the future  Internalized stigma = represents devaluing and discrediting oneself or one’s group based on one’s stigma  **Framework specific to HIV:** Yes | Societal stigma  Intersectional stigma  Structural manifestations  Individual-level manifestations: perceiver  Individual-level manifestations: target  Racial/ethnic HIV disparities  Moderators | Because racial/ethnic minorities at risk of and living with HIV often possess multiple stigmas we adopt an intersectionality framework and conceptualize interdependence among co-occurring stigmas. |
| Fekete, 2018 [15]  **Framework name:** NA  Empirical model  **Terminology:** Stigma  **Domain:** Internalized stigma  https://www.ncbi.nlm.nih.gov/pubmed/28749185 | **Scope:** Impact of internalized HIV stigma, loneliness, and depressive symptoms on sleep quality among people living with HIV in the U.S.  **Aim/Purpose:** To examine whether internalized HIV-stigma was indirectly related to poorer sleep quality through higher levels of loneliness and depressive symptoms  **Framework HIV target:** People living with HIV | **Subtype:** Internalized  **Definition:** HIV-related stigma = coccurs when individuals are discredited or socially devalued because they are living with HIV  **Framework specific to HIV:** Yes | Internalized stigma -> loneliness  Internalized stigma -> depressive symptoms  Internalized stigma -> global sleep quality  Internalized stigma -> daytime sleep dysfunction  Loneliness -> depressive symptoms  Loneliness -> daytime sleep dysfunction  Loneliness -> global sleep quality  Depressive symptoms -> global sleep quality  Depressive symptoms -> daytime sleep dysfunction | People living with HIV who experience HIV-related stigma may experience greater feelings of loneliness, which are related to increased depressive symptoms and poorer sleep quality. |
| Florom-Smith, 2012 [16]  **Framework name:** External, Internal, and Consequences of HIV-Related Stigma  Conceptual model  **Terminology:** Stigma  **Domain:** Internalized stigma  https://www.ncbi.nlm.nih.gov/pubmed/22861652 | **Scope:** HIV-related stigma in HIV testing, care, and prevention  **Aim/Purpose:** Concept exploration of HIV-related stigma to examine the existing knowledge base  **Framework HIV target:** People living with HIV | **Subtype:** Experienced HIV-related stigma  **Definition:** HIV-related stigma = collection of adverse attitudes, beliefs and actions of others against people living with or affected by HIV, which may result in deleterious internalized beliefs or actions taken by persons living with or affected by HIV infection that may result in negative health outcomes  External HIV-related stigma = attitudes or actions expressed toward people living with HIV, includes rejection, avoidance, intolerance, stereotyping, judgmental attitudes, discrimination, disrespect, physical violence or verbal derision, and a lack of HIV transmission knowledge leading to unfounded fears of infection  Internal HIV-related stigma = feelings, beliefs or actions within or instigated by the person living with HIV/AIDS, such as shame, self-blame, secrecy related to fear of disclosure, self-isolation, despair, and great concern over the thoughts and attitudes of others  **Framework specific to HIV:** Yes | EXTERNAL HIV-RELATED STIGMA  Attitudes or actions expressed towards people living with HIV/AIDS  Rejection  Avoidance  Intolerance  Stereotyping  Judgmental attitudes  Discrimination  Disrespect  Physical violence  Verbal derision  Lack of HIV transmission knowledge leading to unfounded fear of infection  INTERNAL HIV-RELATED STIGMA  Feelings, beliefs or actions within or instigated by the person living with HIV/AIDS  Shame  Self-blame  Secrecy related to fear of disclosure  Despair  Great concern over the thoughts and attitudes of others  CONSEQUENCES OF HIV-RELATED STIGMA  Mental health issues  Medication adherence issues  Accession of healthcare services  Employment issues  Housing issues  Risk for physical violence and/or verbal abuse | An inclusive definition of HIV-related stigma consists of two categories: external HIV-related stigma, conceived as the attitudes or actions expressed toward people living with HIV, includes rejection, avoidance, intolerance, stereotyping, judgmental attitudes, discrimination, disrespect, physical violence or verbal derision, and a lack of HIV transmission knowledge leading to unfounded fears of infection; and internal HIV-related stigma includes the feelings, beliefs or actions within or instigated by the person living with HIV/AIDS, such as shame, self-blame, secrecy related to fear of disclosure, self-isolation, despair, and great concern over the thoughts and attitudes of others; HIV-related stigma may result in a number of negative health outcomes for people living with HIV infection; negative health outcomes include mental health issues, medication adherence issues, accession of healthcare services, employment issues, housing  issues, and physical violence and verbal abuse. |
| France, 2015 [17]  **Framework name:** NA  Conceptual model  **Terminology:** Stigma  **Domain:** Internalized stigma  https://www.ncbi.nlm.nih.gov/pubmed/25768695 | **Scope:** Self-stigma among people living with HIV in Ireland  **Aim/Purpose:** To uncover core beliefs underlying self-stigma, the functions thereof, and strategies used to overcome it, among a heterogeneous group of people living with HIV in Ireland using qualitative methods  **Framework HIV target:** People living with HIV in Ireland | **Subtype:** Self-stigma  **Definition:** Stigma = attribute that is significantly discrediting which in the view of others serves to reduce the person who possesses it  Self-stigma = extent to which people endorse negative beliefs and feelings associated with their stigmatized attribute and apply them to self  **Framework specific to HIV:** Yes | SOCIAL FACTORS  Economic, cultural and political landscapes  Access to prevention, treatment and care services  Community support networks  Sources of information  Levels of stigma and discrimination  SELF FACTORS  Belief or meaning systems  Mood states  Coping skills and resilence  Levels of knowledge  Life experience and life skills  Self esteem and self awareness  Sexuality  Disclosure of HIV status  CONTEXTUAL FACTORS  Circumstances and conditions of life  Use of drugs or alcohol  Childhood sexual abuse  Power relationships  Living conditions  Time since diagnosis  Family circumstances | The framework comprises three interacting groups of factors which influence self-stigma: social factors, contextual factors and self factors; the complex interaction between these factors influences the development and perpetuation of self-stigma. |
| Health Policy Plus, 2017 [20]  Nyblade, 2016[136]  **Framework name:** Stigma Drivers and Manifestations: Relationship to the HIV Treatment Cascade  Conceptual model  **Terminology:** Stigma,Discrimination  **Domain:** Internalized stigma  http://www.healthpolicyplus.com/ns/pubs/7155-7275_StigmaReductionFactsheetversion.pdf  Health Policy Plus (Adapted from: Nyblade, L. and RTI International. 2016) | **Scope:** Stigma drivers and manifestations  **Aim/Purpose:** To addresses the drivers of stigma and discrimination  **Framework HIV target:** People living with HIV | **Subtype:** Experienced, anticipated, perceived, internalized stigma  **Definition:** NA  **Framework specific to HIV:** Yes | Immediately actionable drivers  Fear of transmission  Awareness of stigma  Attitudes  Health facility environment  Stigma manifestations  Experienced, anticipated, perceived, internalized, etc  Avoidance, harassment, refusal to treat, etc  HIV treatment cascade  Testing  Linkage to care and retention in care  Adherence  Viral suppression | Stigma and discrimination are firmly established as key barriers that impede scaleup of HIV care and treatment, impacting all stages of the treatment cascade. |
| Katz, 2013 [24]  **Framework name:** NA  Empirical model  **Terminology:** Stigma  **Domain:** Internalized stigma  https://www.ncbi.nlm.nih.gov/pubmed/24242258 | **Scope:** Stigma and antiretroviral therapy adherence  **Aim/Purpose:** To assess the relationship between HIV-related stigma and adherence to HIV antiretroviral therapy  **Framework HIV target:** HIV-positive persons | **Subtype:** Internalized, enacted  **Definition:** NA  **Framework specific to HIV:** Yes | Enacted stigma -> group-specific: internalized stigma and concealment -> adherence  Enacted stigma -> general: adaptive coping and social support -> adherence  Group-specific: internalized stigma and concealment <-> general: adaptive coping and social support  Poverty <-> enacted stigma  Poverty -> adherence  Moderators: health systems and social norms -> enacted stigma and group-specific: internalized stigma and concealment | The stigma of HIV compromises adherence through general as well as group-specific psychological processes; adaptive coping and social support were critical determinants of participants’ ability to overcome structural and economic barriers associated with poverty to successfully adhere to antiretroviral therapy. |
| Lipira, 2019 [25]  **Framework name:** NA  Empirical model  **Terminology:** Stigma  **Domain:** Internalized stigma  https://www.ncbi.nlm.nih.gov/pubmed/30343422 | **Scope:** HIV-related stigma and viral supression among African-American women living with HIV in Chicago and Birmingham  **Aim/Purpose:** To evaluate the relationship between HIV-related stigma and viral suppression, and to assess the role of depression and nonadherence to antiretroviral therapy as mediators  **Framework HIV target:** African-American women living with HIV | **Subtype:** NA  **Definition:** HIV-related stigma = prejudice, discounting, discrediting and discrimination that is directed at people perceived as having HIV  **Framework specific to HIV:** Yes | HIV related stigma  Depressive symptoms  ART nonadherence  Viral suppression | HIV-related stigma is common among African-American women living with HIV, and those who experience higher levels of stigma are less likely to be virally suppressed. |
| Logie, 2016 [27]  **Framework name:** NA  Empirical model  **Terminology:** Stigma,Discrimination  **Domain:** Internalized stigma  https://www.ncbi.nlm.nih.gov/pubmed/27669510 | **Scope:** Stigma, racial discrimination, housing insecurity and wellbeing  **Aim/Purpose:** To test a conceptual model of the pathways linking HIV-related stigma, racial discrimination, housing insecurity, and wellbeing  **Framework HIV target:** African and Caribbean Black Women Living with HIV | **Subtype:** Personalized  **Definition:** Internalized stigma = acceptance of negative views about people living with HIV  Enacted stigma = negative treatment from others  Perceived stigma = awareness of negative social norms and attitudes towards people living with HIV  **Framework specific to HIV:** Yes | Racial discrimination -> HIV related stigma  Racial discrimination -> self-rated health  Racial discrimination -> social support  Racial discrimination -> depression  HIV related stigma -> self-rated health  HIV related stigma -> social support  HIV related stigma -> depression  Housing insecurity -> depression  Housing insecurity -> social support | Racial discrimination had significant direct effects on: HIV-related stigma, depression and social support, and an indirect effect on self-rated health via HIV-related stigma. HIV-related stigma and housing insecurity had direct effects on depression and social support, and HIV-related stigma had a direct effect on self-rated health. |
| Logie, 2017 [26]  **Framework name:** Factors Contributing to Quality of Life Among African and Caribbean Women Living with HIV in Ontario  Empirical model  **Terminology:** Stigma  **Domain:** Internalized stigma  https://www.ncbi.nlm.nih.gov/pubmed/27750027 | **Scope:** Stigma, racial discrimination, social support, and quality of life  **Aim/Purpose:** To test a conceptual model of pathways between HIV related stigma, racial discrimination, depression, social support, and quality of life  **Framework HIV target:** Women living with HIV | **Subtype:** Personalized stigma  **Definition:** HIV-related stigma = includes processes of devaluing, labeling, and stereotyping that result in loss of status, unjust and unfair treatment, and social isolation of people living with HIV  **Framework specific to HIV:** Yes | Racism -> HIV-related stigma  HIV-related stigma -> depression  Racism -> depression  Racism -> social support  Racism -> quality of life  Depression -> quality of life  Social support -> quality of life | Racial discrimination was associated with increased HIV-related stigma, and HIV-related stigma and racial discrimination compromised quality of life. |
| Logie, 2018 [29]  **Framework name:** HIV-Related Stigma, Racial Discrimination, and Gender Discrimination on Health-Related Quality of Life (Mental and Physical Health) Among Women Living with HIV in Canada (2013-2015)  Empirical model  **Terminology:** Stigma,Discrimination  **Domain:** Internalized stigma  https://www.ncbi.nlm.nih.gov/pubmed/29277410 | **Scope:** Stigma, discrimination on quality of life among women living with HIV  **Aim/Purpose:** To test pathways from multiple types of stigma to physical and mental health-related quality of life  **Framework HIV target:** Women living with HIV | **Subtype:** NA  **Definition:** Stigma = processes include labeling, loss of status, and discrimination in contexts of unequal power distribution  **Framework specific to HIV:** Yes | HIV related stigma -> racial discrimination  HIV related stigma -> gender discrimination  HIV related stigma -> economic insecurity  HIV related stigma -> social support  HIV related stigma -> quality of life mental health  HIV related stigma -> quality of life physical health  Racial discrimination -> HIV related stigma  Racial discrimination -> gender discrimination  Racial discrimination -> economic insecurity  Racial discrimination -> quality of life physical health  Gender discrimination -> HIV related stigma  Gender discrimination -> racial discrimination  Gender discrimination -> social support  Gender discrimination -> quality of life mental health  Economic insecurity -> quality of life mental health  Economic insecurity -> quality of life physical health  Social support -> quality of life mental health  Social support -> quality of life physical health  Quality of life mental health -> quality of life physical health  Quality of life physical health -> quality of life mental health | HIV-related stigma and gender discrimination had significant direct effects on mental health-related quality of life.. Social support mediated the relationship between HIV-related stigma and mental health-related quality of life. Findings reveal complex relationships between intersecting stigma and health-related quality of life. |
| Logie, 2018 [28]  **Framework name:** NA  Empirical model  **Terminology:** Stigma  **Domain:** Internalized stigma  https://www.ncbi.nlm.nih.gov/pubmed/29135650 | **Scope:** Women living with HIV in Canada  **Aim/Purpose:** To examine pathways from HIV-related stigma to antiretroviral therapy initiation, current use, and adherence among women living with HIV in Canada  **Framework HIV target:** Women living with HIV in Canada | **Subtype:** Personalized, negative self-image, and public attitudes  **Definition:** NA  **Framework specific to HIV:** Yes | Personalized (stigma)  Negative self image (stigma)  Public attitudes (stigma)  Depression  HIV disclosure concerns  Antiretroviral therapy initiation  Current antiretroviral therapy use  Antiretroviral therapy adherence | HIV-related stigma is associated with reduced likelihood of antiretroviral therapy initiation and current antiretroviral therapy use, and suboptimal antiretroviral therapy adherence. |
| Miller, 2016 [33]  **Framework name:** NA  Empirical model  **Terminology:** Stigma,Discrimination  **Domain:** Internalized stigma  https://www.ncbi.nlm.nih.gov/pubmed/27348608 | **Scope:** HIV stigma and the psychological and physical well-being of people with HIV  **Aim/Purpose:** To examine the transactional relationships between HIV stigma with psychological and physical well-being of people with HIV  **Framework HIV target:** New England residents with HIV | **Subtype:** Perceived  **Definition:** NA  **Framework specific to HIV:** Yes | Well-being -> stigma  Race/ethnicity  Age  Gender  Sexual orientation  Years since diagnosis | Three aspects of HIV stigma and well-being indices within and across the three time points. |
| Mo, 2015 [34]  **Framework name:** NA  Empirical model  **Terminology:** Stigma  **Domain:** Internalized stigma  https://www.ncbi.nlm.nih.gov/pubmed/24879629 | **Scope:** Stigma on depression and anxiety among children of HIV-infected parents in China  **Aim/Purpose:** Examined the relationship between associative stigma, self esteem, optimism, anxiety and depression among children of HIV-infected parents in rural China  **Framework HIV target:** Children of HIV-infected parents in rural China | **Subtype:** Associative  **Definition:** Stigma = attribute linking a person to a set of undesirable characteristics that may lead to prejudice and discrimination  **Framework specific to HIV:** Yes | Associative stigma -> depression  Associative stigma -> anxiety  Associative stigma -> self-esteem  Associative stigma -> optimism  Optimism -> anxiety  Optimism -> depression  Self-esteem -> anxiety  Self-esteem -> depression | Associative stigma had a significant negative relationship on self-esteem and optimism, which were associated with higher levels of depression and anxiety. The indirect effects of associative stigma on depression and anxiety were significant. Findings suggest that associative stigma has a significant negative impact on mental health of children affected by HIV. |
| Pantelic, 2017 [36]  **Framework name:** NA  Empirical model  **Terminology:** Stigma  **Domain:** Internalized stigma  https://www.ncbi.nlm.nih.gov/pubmed/28853517 | **Scope:** Stigma and risk pathways  **Aim/Purpose:** To test a theoretical model of multi-level risk pathways to internalized HIV stigma  **Framework HIV target:** Adolescents living with HIV | **Subtype:** Internalized, enacted, anticipated  **Definition:** Internalized HIV stigma = person living with HIV internalizes perceived negative public attitudes towards people living with HIV and accepts them as applicable to themself  **Framework specific to HIV:** Yes | HIV related disability -> enacted HIV stigma  HIV related disability -> abuse victimization  Enacted HIV stigma -> depressive symptoms  Abuse victimization -> anticipated HIV stigma  Enacted HIV stigma -> anticipated HIV stigma  Abuse victimization -> depressive symptoms  Anticipated HIV stigma -> internalized HIV stigma  Depressive symptoms -> internalized HIV stigma  internalized HIV stigma -> anticipated HIV stigma  internalized HIV stigma -> depressive symptoms | Indirect pathways suggest multi-level mechanisms leading to internalized HIV stigma. Findings suggest that protection from violence within homes, communities and schools may interrupt risk pathways from HIV-related health problems to psychological distress and internalized HIV stigma. |
| Prati, 2016 [39]  **Framework name:** Conceptual Models for HIV Uninfected Individuals and People Living with HIV/AIDS  Empirical model  **Terminology:** Stigma  **Domain:** Internalized stigma  https://link.springer.com/article/10.1007/s11135-015-0168-2 | **Scope:** Knowing someone with HIV and HIV disclosure  **Aim/Purpose:** To evaluate the role of knowing someone living with HIV/AIDS and disclosure of one’s HIV-positive status in the HIV Stigma Framework using a Bayesian mediation analysis  **Framework HIV target:** People living with HIV/AIDS | **Subtype:** Internalized, enacted stigma  **Definition:** NA  **Framework specific to HIV:** Yes | Knowledge of someone living with HIV/AIDS  Enacted HIV stigma  Disclosure of HIV-positive status  Internalized stigma  Undetectable viral load  Engagement in HIV care | Knowing someone living with HIV/AIDS was negatively related to internalized stigma among PLWHA. Internalized stigma mediated the relationship between personal knowledge of someone living with HIV/AIDS and disclosure of one’s HIV-positive status. In turn, disclosure of one’s HIV-positive status mediated the relationship between internalized stigma and engagement in HIV care. Undetectable viral load was predicted by internalized stigma but not by disclosure of one’s HIV-positive status and enacted stigma. |
| Rao, 2012 [41]  **Framework name:** NA  Empirical model  **Terminology:** Stigma  **Domain:** Internalized stigma  https://www.ncbi.nlm.nih.gov/pubmed/21380495 | **Scope:** Stigma, depression and medication adherence  **Aim/Purpose:** We investigated whether depressive symptoms mediate the relationship between stigma and medication adherence  **Framework HIV target:** People living with HIV | **Subtype:** Enacted, internalized  **Definition:** Stigma = negative attitudes held by members of the public  HIV-related stigma = occurs in the social environment, where it may be sensed or experienced by a person living with HIV (PLWH), who may subsequently internalize this stigma  **Framework specific to HIV:** Yes | Stigma -> depressive symptoms  Depressive symptoms -> adherence  Stigma -> adherence | Stigma is associated with depressive symptoms, which in turn is associated with adherence. |
| Relf, 2019 [42]  **Framework name:** NA  Empirical model  **Terminology:** Stigma,Discrimination  **Domain:** Internalized stigma  https://www.ncbi.nlm.nih.gov/pubmed/30893124 | **Scope:** Age, stigma, discrimination, medical distrust, adherence, quality of life, and depression among women living with HIV  **Aim/Purpose:** To explore the moderating effect of age on everyday discrimination; group-based medical distrust; enacted, anticipated, internalized HIV stigma; depressive symptoms; HIV disclosure; engagement in care; antiretroviral medication adherence; and quality of life among women living with HIV  **Framework HIV target:** Women living with HIV | **Subtype:** Internalized, enacted, anticipated  **Definition:** Anticipated HIV stigma = reflected in the person’s concerns about discrimination or adverse events that might happen should one’s HIV status become known by others – whether a consequence of intentional or planned disclosure, or inadvertent disclosure through breaches in confidentiality  Perceived or community stigma = relates to how much a PLWH believes that the public stigmatizes someone with HIV  Internalized HIV stigma = also referred to as self-stigma, occurs when the negative attitudes, beliefs, and feelings associated with HIV become integrated into self – threatening self-concept and self-esteem  **Framework specific to HIV:** Yes | Everyday discrimination -> group based medical distrust  Everyday discrimination -> depressive symptoms  Everyday discrimination -> anticipated HIV stigma  Group based medical distrust -> everyday discrimination  Group based medical distrust -> internalized HIV stigma  Group based medical distrust -> engagement in care  Enacted HIV stigma -> anticipated HIV stigma  Enacted HIV stigma -> internalized HIV stigma  Internalized HIV stigma -> HIV disclosure  Internalized HIV stigma -> anticipated HIV stigma  Internalized HIV stigma -> depressive symptoms  Depressive symptoms -> engagement in care  Depressive symptoms -> ART adherence  Depressive symptoms -> quality of life  Age -> internalized HIV stigma  Age -> quality of life  Engagement in care -> quality of life | Everyday discrimination, group-based medical distrust, and internalized stigma adversely affect depressive symptoms, ART medication adherence, and engagement in care, which collectively influence the quality of life of women living with HIV. |
| Rice, 2017 [43]  **Framework name:** NA  Empirical model  **Terminology:** Stigma  **Domain:** Internalized stigma  https://www.ncbi.nlm.nih.gov/pubmed/28885270 | **Scope:** HIV clinic patients and HIV care visit adherence in Birmingham, Alabama  **Aim/Purpose:** To investigated the association between internalized stigma and HIV visit adherence  **Framework HIV target:** HIV clinic patients | **Subtype:** Internalized  **Definition:** Internalized HIV-related stigma = acceptance and adoption of negative beliefs existing in society about being HIV-positive and applying them to oneself  **Framework specific to HIV:** Yes | Internalized stigma -> visit adherence  Internalized stigma -> medication adherence  Visit adherence -> medication adherence | Results highlight the importance of internalized HIV stigma to multiple and sequential HIV care continuum outcomes. |
| Shin, 2018 [45]  **Framework name:** NA  Empirical model  **Terminology:** Stigma  **Domain:** Internalized stigma  https://www.ncbi.nlm.nih.gov/pubmed/29934793 | **Scope:** Mediation analysis of food insecurity, internalized stigma, and opportunistic infections  **Aim/Purpose:** To investigate the potential pathways for the association between internalized stigma and opportunistic infections among women living with HIV in rural India  **Framework HIV target:** Women living with HIV | **Subtype:** Internalized  **Definition:** Stigma = set of negative beliefs that society has about something or someone  **Framework specific to HIV:** Yes | Internalized stigma  Food insecurity  Adherence  Opportunistic infections  Confounders | Food insecurity was a strong mediator of the association between internalized stigma and the number of opportunistic infections, while the indirect effect of stigma through adherence was minimal for both outcomes. |
| Slater, 2015 [46]  **Framework name:** NA  Empirical model  **Terminology:** Stigma  **Domain:** Internalized stigma  https://www.ncbi.nlm.nih.gov/pubmed/25249266 | **Scope:** Multiple stigmas affecting quality of life among older gay men with HIV  **Aim/Purpose:** To determine predictors of homonegativity, internalized HIV stigma, and ageism, and stigma experiences that were predictive of quality of life  **Framework HIV target:** Older gay men with HIV | **Subtype:** Internalized  **Definition:** Stigma = someone’s unacceptable or undesirable characteristics that are deeply discrediting and that impact his/ her place in society  **Framework specific to HIV:** Broader Multiple | Internalized HIV stigma  Availability of support  Total medication  HIV support group participation  Age  Emotion-focused coping  Quality of life  Ageism  Homo-negativity  Problem focused coping | Older gay men with HIV may experience stigma related to their sexual orientation, HIV status and age, which together may have a greater detrimental effect on quality of life than when independently considering individual stigmas. |
| Steward, 2008 [49]  Steward, 2011 [141]  **Framework name:** Theoretical Framework Linking Facets of Stigma, Avoidance of HIV Serostatus Disclosure, and Psychological Distress  Empirical model  **Terminology:** Stigma,Discrimination  **Domain:** Internalized stigma  https://www.ncbi.nlm.nih.gov/pubmed/18599171 | **Scope:** HIV-related stigma in Southern India  **Aim/Purpose:** To examine whether a multi-component framework, initially consisting of enacted, felt normative, and internalized forms of individual stigma experiences, could be used to understand HIV-related stigma in Southern India  **Framework HIV target:** People living with HIV in southern India | **Subtype:** Enacted, felt normative, vicarious and internalized  **Definition:** Enacted stigma = overt acts of discrimination and hostility directed at a person because of his or her perceived stigmatized status  Felt normative stigma = subjective awareness of stigma  Internalize stigma = extent to which an individual accepts stigma as valid  **Framework specific to HIV:** Yes | Enacted stigma  Vicarious stigma  Felt normative stigma  Internalized stigma  Avoiding disclosure of HIV status  Psychological distress | Results suggest that enacted and vicarious stigma influenced felt normative stigma; that enacted, felt normative, and internalized stigma were associated with higher levels of depression; and that the associations of depression with felt normative and internalized forms of stigma were mediated by the use of coping strategies designed to avoid disclosure of one's HIV serostatus. |
| Tsai. 2013 [53]  **Framework name:** NA  Empirical model  **Terminology:** Stigma  **Domain:** Internalized stigma  https://www.ncbi.nlm.nih.gov/pubmed/23690283 | **Scope:** Conceptual model of internalized stigma, social distance, and disclosure of HIV seropositivity  **Aim/Purpose:** To study the relationship between internalized HIV-related stigma and serostatus disclosure and to determine the extent to which this association varies with the degree of social distance  **Framework HIV target:** People living with HIV | **Subtype:** Internalized  **Definition:** Internalized HIV-related stigma = extent to which persons with HIV accept their discredited status as valid and develop self-defacing internal representations of themselves  **Framework specific to HIV:** Yes | Internalized stigma -> disclosure of seropositivity  Health status -> disclosure of seropositivity  Social distance -> between internalized stigma and disclosure of seropositivity | Inhibiting effects of stigma on disclosure vary across different types of social ties |
| Turan, 2016 [56]  **Framework name:** NA  Empirical model  **Terminology:** Stigma  **Domain:** Internalized stigma  https://www.ncbi.nlm.nih.gov/pubmed/26885803 | **Scope:** Stigma, social isolation and depression on antiretroviral therapy adherence in women  **Aim/Purpose:** To test whether social isolation (loneliness and lack of social support) and depressive symptoms mediate the relationship between internalized stigma and HIV medication non-adherence in a large sample of racially diverse women living with HIV across the USA  **Framework HIV target:** Women living with HIV | **Subtype:** Internalized  **Definition:** NA  **Framework specific to HIV:** Yes | Internalized HIV stigma  Adherence  Social support  Depressive symptoms | Interconnected psychosocial mechanisms affect ART adherence, and improvements in adherence may require multifaceted interventions addressing both mental health and interpersonal factors, especially for minority women. |
| Turan, 2017 [54]  **Framework name:** Modified Health Stigma Framework  Empirical model  **Terminology:** Stigma  **Domain:** Internalized stigma  https://www.ncbi.nlm.nih.gov/pubmed/27272742 | **Scope:** Effect of stigma on health and psychosocial outcomes  **Aim/Purpose:** To examine how the effects of perceived HIV stigma in the community on health outcomes for people living with HIV are mediated by internalized stigma and anticipated stigma  **Framework HIV target:** People living with HIV | **Subtype:** Internalized, anticipated, enacted, perceived  **Definition:** NA  **Framework specific to HIV:** Yes | Perceived community stigma -> internalized stigma -> affective outcomes  Perceived community stigma -> internalized stigma -> anticipated stigma (community) -> medication adherence  Perceived community stigma -> anticipated stigma (community) -> medication adherence  Perceived community stigma -> internalized stigma -> anticipated stigma (friends/family) -> social support  Perceived community stigma -> internalized stigma -> social support  Perceived community stigma -> anticipated stigma (friends/family) -> social support  Perceived community stigma -> internalized stigma -> anticipated stigma (community) -> social support  Perceived community stigma -> internalized stigma -> social support  Perceived community stigma -> anticipated stigma (community)> social support  Perceived community stigma -> internalized stigma -> anticipated stigma (healthcare) -> trust in physicians | Experienced stigma is hypothesized to predict physical health; internalized stigma is hypothesized to predict affective, cognitive, and mental health outcomes as well as health care behaviors, and anticipated stigma is hypothesized to predict health care behaviors and interpersonal outcomes. |
| Vyavaharkar, 2010 [61]  **Framework name:** NA  Empirical model  **Terminology:** Stigma  **Domain:** Internalized stigma  https://www.ncbi.nlm.nih.gov/pubmed/19879778 | **Scope:** Stigma, social support, and depression in HIV-infected African American women  **Aim/Purpose:** To explore the relationships between HIV-related stigma, social support, and depression in a sample of African American women with HIV disease living in the rural Southeastern United States  **Framework HIV target:** HIV-infected African American women living in the rural Southeastern United States | **Subtype:** Perceived, internalized  **Definition:** NA  **Framework specific to HIV:** Yes | HIV-related stigma (perceived stigma, internalized stigma)  Sources of available support  Depression | HIV-related stigma (perceived stigma and internalized stigma) were found to mediate the relationship between sources of available social support and depression. |
| Williams, 2014 [64]  **Framework name:** NA  Conceptual model  **Terminology:** Stigma  **Domain:** Internalized stigma  https://www.ncbi.nlm.nih.gov/pubmed/24477769 | **Scope:** HIV/AIDS related stigma in Sub-Saharan Africa  **Aim/Purpose:** To summarize the major relationships discussed in the preceding theoretical description of the ecological processes through which HIV/AIDS related stigma is related to HIV/AIDS-related health services utilization and to HIV prevalence and HIV incidence in the context of Sub-Saharan Africa  **Framework HIV target:** People with HIV living in Sub-Saharan Africa | **Subtype:** NA  **Definition:** Stigma = attribute that is deeply discrediting,’’ or an attribute that makes [a person] different from others in the category of persons available for him to be, and of a less desirable kind—in the extreme, a person who is quite thoroughly bad, or dangerous, or weak  **Framework specific to HIV:** Yes | Community-level stigma  Normalization of behavior and attitudes about HIV  Community-level HIV prevalence  Community-level HIV incidence  Individual-level perception or fear of stigma  Sexual and other risk behaviors  Individual-level health service utilization  Longevity of HIV positive persons  Individual-level HIV transmission | Draws from an empirical literature review as well as from well-known theoretical frameworks from multiple disciplines, to propose a theoretical framework for the ecological and multilevel relationships among HIV/AIDS related stigma, health service utilization, and HIV outcomes in this context. |
| Zang, 2014 [69]  **Framework name:** NA  Empirical model  **Terminology:** Stigma  **Domain:** Internalized stigma  https://www.ncbi.nlm.nih.gov/pubmed/24853730 | **Scope:** HIV stigma, social network support, and individualism–collectivism  **Aim/Purpose:** To examine the interrelationships among individualism–collectivism, HIV stigma, and social network support  **Framework HIV target:** People living with HIV/ AIDS in China | **Subtype:** Self  **Definition:** Public stigma = negative attitudes and judgments toward a person on the basis of a specific trait endorsed by society  Self-stigma = fear of societal attitudes and discrimination perceived by individuals with stigmatizing attributes  **Framework specific to HIV:** Yes | Family Individualism–Collectivism Interpersonal Assessment Inventory  Friends Individualism–Collectivism Interpersonal Assessment Inventory  Neighbor Individualism–Collectivism Interpersonal Assessment Inventory  Social network support (family)  Social network support (friends)  Social network support (neighbor)  Public stigma  Self stigma | HIV stigma may mediate the relationship between collectivist culture and social network support |
| Darlington, 2017 [9]  **Framework name:** Current State-of-Science of HIV-Related Stigma Among HIV + Women in the Southern US  Empirical model  **Terminology:** Stigma  **Domain:** Internalized stigma,Stigma or discrimination in healthcare settings  https://www.ncbi.nlm.nih.gov/pubmed/27492025 | **Scope:** HIV-related stigma among women living in the South  **Aim/Purpose:** Understanding HIV-related stigma among women in the Southern United States  **Framework HIV target:** HIV positive women | **Subtype:** Perceived, experienced, internalized  **Definition:** HIV-related stigma = shame or disgrace attached to this disease and expressed through negative social reactions towards people infected with the virus  Perceived stigma = expectation of negative perceptions before they are experienced, often leading to limited disclosure for fear of rejection  Experienced stigma = what actually happens, in the form of social discrimination and rejection  Internalized stigma = affects a person’s own psychological self-image and value  **Framework specific to HIV:** Yes | Cultural Perceptions  Healthcare provider perceptions  Stigma among HIV positive women  Identity of HIV positive women  Redefined identity  Depression  Self-isolation  Reproductive decision-making  Limited disclosure  Medication no-adherence and healthcare access  Sexual decision-making  Structural level  Interpersonal level  Intrapersonal level  Stigma-reduction interventions | A description of the current state-of-science of HIV-related stigma among HIV and women in the Southern US |
| Earnshaw, 2013 [14]  Earnshaw, 2009 [122]; Misir, 2015 [131]; Goodin, 2018 [125]; Reinius, 2018 [134]  **Framework name:** Hypothesized associations between HIV stigma mechanisms and health and well-being among people living with HIV  Empirical model  **Terminology:** Stigma  **Domain:** Internalized stigma,Stigma or discrimination in healthcare settings  https://www.ncbi.nlm.nih.gov/pubmed/23456594 | **Scope:** HIV stigma mechanism and health and well-being  **Aim/Purpose:** To test the HIV stigma framework evaluating HIV stigma mechanisms and well being  **Framework HIV target:** Community clinic providing integrated HIV care | **Subtype:** Internalized, anticipated, enacted  **Definition:** HIV stigma = social or structural level phenomenon that exists when labeling, stereotyping, status loss, and discrimination occur within a power structure  Internalized HIV stigma = endorsing negative feelings and beliefs associated with HIV and applying them to the self  Anticipated HIV stigma = expectations of discrimination, stereotyping, and/or prejudice from others in the future due to one’s HIV  Enacted HIV stigma = experiences of discrimination, stereotyping, and/or prejudice from others in the past or present due to one’s HIV  **Framework specific to HIV:** Yes | HIV stigma mechanism: internalized HIV stigma, anticipated HIV stigma, enacted HIV stigma  Health and well-being: affective (helplessness, acceptance of HIV, perceived benefits of HIV), behavioral (antiretroviral adherence medical care visits), physical (CD4 count, chronic illnesses) | Internalized stigma associates significantly with indicators of affective (i.e., helplessness regarding, acceptance of, and perceived benefits of HIV) and behavioral (i.e., days in medical care gaps and ARV nonadherence) health and well-being. Enacted and anticipated stigma associate with indicators of physical health and wellbeing (i.e., CD4 count less than 200 and chronic illness comorbidity respectively). |
| Logie, 2019 [30]  **Framework name:** NA  Empirical model  **Terminology:** Stigma,Discrimination  **Domain:** Internalized stigma,Stigma or discrimination in healthcare settings  https://www.ncbi.nlm.nih.gov/pubmed/31079013 | **Scope:** Women living with HIV in Canada  **Aim/Purpose:** To integrate concepts from multiple frameworks and test pathways from intersectional stigma to HIV-related health outcomes via interpersonal, psychological, mental health, and stress mechanisms among women living with HIV in Canada  **Framework HIV target:** Women living with HIV | **Subtype:** Intersectional  **Definition:** NA  **Framework specific to HIV:** Yes | HIV-related stigma  Racial discrimination  Gender discrimination  Social support  Women-centered healthcare  Presence of depressive symptoms  Presence of severe depressive symptoms  Resilience  History of injection drug use  ART adherence  CD4 count  Undetectable viral load | Integrating concepts from multiple frameworks to examine pathways from intersectional stigma to mental health via interpersonal and institutional support, and from mental health to care engagement and HIV-related health via coping strategies among women living with HIV. |
| Thi, 2008 [51]  **Framework name:** NA  Empirical model  **Terminology:** Stigma,Discrimination  **Domain:** Internalized stigma,Stigma or discrimination in healthcare settings  https://www.ncbi.nlm.nih.gov/pubmed/18360743 | **Scope:** Causes of HIV stigma and discrimination in Vietnam  **Aim/Purpose:** To provide a schematic diagram of stigma and discrimination against people living with HIV addressing causes, effects, and relationships  **Framework HIV target:** People living with HIV/AIDS | **Subtype:** Internalized  **Definition:** NA  **Framework specific to HIV:** Yes | Misperceptions about HIV transmission risk -> fear of HIV transmission  Misperceptions about HIV transmission risk -> STIGMA against people living with HIV in the community  Fear of HIV transmission -> STIGMA against people living with HIV in the community  Negative representations of people living with HIV in the media and public health messages -> fear of HIV transmission  Negative representations of people living with HIV in the media and public health messages -> association of HIV with illegal & immoral behavior  Negative representations of people living with HIV in the media and public health messages -> STIGMA against people living with HIV in the community  Association of HIV with illegal & immoral behavior -> STIGMA against people living with HIV in the community  Association of HIV with illegal & immoral behavior -> negative attitudes about people living with HIV  Negative attitudes about people living with HIV -> STIGMA against people living with HIV in the community  STIGMA against PLHIV in the community -> DISCRIMINATION  DISCRIMINATION family/community -> isolation  DISCRIMINATION family/community -> self stigmatization  DISCRIMINATION family/community -> suicidal thoughts  DISCRIMINATION family/community -> depression  DISCRIMINATION health care sector/workplace-> fewer job opportunities  DISCRIMINATION health care sector/workplace-> decreased access to health care  DISCRIMINATION health care sector/workplace-> loss of employment and income  DISCRIMINATION health care sector/workplace-> loss of social and family support | Three main themes relating to stigma and discrimination emerged: (1) attitudes, misperceptions, and negative media representations led to stigmatization of people living with HIV; (2) acts of discrimination occurred within various sectors of Vietnamese society, including the family, the community, the healthcare sector, and the workplace; and (3) stigma and discrimination resulted in negative effects on people living with HIV. |
| Wardell, 2018 [62]  **Framework name:** NA  Empirical model  **Terminology:** Stigma  **Domain:** Internalized stigma,Stigma or discrimination in healthcare settings  https://www.ncbi.nlm.nih.gov/pubmed/30124756 | **Scope:** Associations among HIV-related stigma, coping, and problem drinking  **Aim/Purpose:** Examining prospective bidirectional and mediated associations among HIV-related stigma, maladaptive coping, and alcohol use severity in patients enrolled in the Ontario HIV Treatment Network Cohort study  **Framework HIV target:** Patients receiving medical care for HIV | **Subtype:** Enacted, internalized  **Definition:** HIV-related stigma = devaluation of individuals based on their HIV status , and includes “prejudice, discounting, discrediting, and discrimination directed at people perceived to have AIDS or HIV  **Framework specific to HIV:** Yes | HIV stigma ->maladaptive coping  Maladaptive coping -> HIV stigma  Maladaptive coping -> alcohol use severity  Alcohol use severity -> maladaptive coping | Cross-lagged panel model of the prospective associations among HIV-related stigma, maladaptive coping strategies, and alcohol use severity. |
| Watt, 2020[63]  **Framework name:** NA  Conceptual model  **Terminology:** Stigma  **Domain:** Internalized stigma,Stigma or discrimination in healthcare settings  https://www.ncbi.nlm.nih.gov/pubmed/32795711 | **Scope:** Women and their partners attending a first antenatal care appointment in Tanzania  **Aim/Purpose:** Stigma visual used to discuss how issues of internalized, anticipated and enacted stigma might relate to their situation of living with HIV, and to try and reduce these as barriers of HIV care engagement  **Framework HIV target:** Women living with HIV and HIV-negative women and their partners | **Subtype:** Internalized, anticipated, enacted  **Definition:** NA  **Framework specific to HIV:** Yes | Internalized stigma  CBT component: negative thoughts/feelings about oneself  Enacted stigma  CBT component: negative thoughts/feelings about others  Anticipated stigma  CBT component: negative thoughts/feelings about the future | Addressing HIV stigma at the first antenatal care visit can help individuals living with HIV to overcome stigma related barriers to the initiation and maintenance of HIV care, and can reduce stigmatizing attitudes among those who test negative for HIV. |
| Gilbert, 2016 [18]  **Framework name:** NA  Conceptual model  **Terminology:** Stigma  **Domain:** Internalized stigma,Stigma or discrimination in healthcare settings,Stigma or discrimination in law  https://www.ncbi.nlm.nih.gov/pubmed/26781444 | **Scope:** HIV-related stigma evidenced in South Africa  **Aim/Purpose:** To examine individual and social/structural components of HIV-related stigma in South Africa  **Framework HIV target:** HIV-related stigma in South Africa | **Subtype:** Internalized, enacted discrimination, by association, instrumental, symbolic  **Definition:** Stigma = co-occurrence of labelling, stereotyping, separating, status loss and discrimination  **Framework specific to HIV:** Yes | Stigma process  Stigma triggers  Stigmatizing behaviors  Types of stigma  Stigma outcomes  Forces impacting on/shaping stigma  Wider social context  Healthcare system  Agents | To provide a comprehensive framework that offers insights into the individual as well as the social/structural components of HIV-related stigma in a particular context. |
| Pescosolido, 2015 [37]  **Framework name:** Framework Integrating Normative Influences on Stigma (FINIS)  Empirical model  **Terminology:** Stigma,Discrimination  **Domain:** Internalized stigma,Stigma or discrimination in healthcare settings,Stigma or discrimination in law  https://www.ncbi.nlm.nih.gov/pubmed/26855471 | **Scope:** A multilevel approach that can be tailored to stigmatized statuses  **Aim/Purpose:** To provide a theoretical architecture of concepts, proposing a stigma complex, a system of interrelated, heterogeneous parts bringing together insights across disciplines to provide a more realistic and complicated sense of the challenge facing research and change efforts  **Framework HIV target:** General stigma model | **Subtype:** Perceived, endorsed, anticipated, received, enacted, self-stigma, courtesy stigma, public stigma, prover-based stigma, structural stigma  **Definition:** Stigma = deeply discrediting attribute; “mark of shame”; “mark of oppression”; devalued social identity  Discrimination = behaviors that act to endorse and reinforce stereotypes, and disadvantage those labeled  Self- stigma = internalized acceptance of stereotypes and prejudice  Courtesy stigma = stereotypes, prejudice, and discrimination by association with marked groups  Public stigma = stereotypes, prejudice, and discrimination endorsed by the general population  Provider-based stigma = prejudice and discrimination voiced or exercised, consciously or unconsciously, by occupational groups designated to provide assistance to stigmatized groups  Structural stigma = prejudice and discrimination by policies, laws, and constitutional practice; also called institutionalized stigma  Perceived stigma = represents agreement with a statement that prejudice and discrimination exist toward a labeled group  Anticipated stigma = refers to individuals’ or groups’ expectations that others will devalue and discriminate against them  Endorsed stigma = expressed agreement with existing stereotypes  Received stigma = focuses on the stigmatized, asking whether they have personally experienced prejudice and discrimination  Enacted stigma = occurs when individuals behave in a way that prejudices or discriminates against the stigmatized (i.e., differential treatment)  **Framework specific to HIV:** No | THE INDIVIDUAL  Social psychological context: the stigmatizer (explicit, implicit, intention and cognitive resources); the stigmatized: awareness, stereotype threat, attributional ambiguity  Disease characteristics: concealability, contagion risk, course of disease, control of disease, culpability, comprehension  Social characteristics: age, race/ethnicity, class, social distance  Behaviors: positive/negative, severity, visibility, frequency  Mark  THE COMMUNITY  Cognitive responses  Affective responses  Media context (exposure, genre, production characteristics)  Social network characteristics (size, strength, endurance, valence)  National context: economic development, welfare state ideology, health care system, globalization, cultural values  Label  Stigma (attitudes, behavioral predisposition, behavior)  Response (self: psychological, external attribution, disengagement, social comparison; social: self-stigmatization, protest/righteous anger, education/advocacy; others: distance, caretaking, and advocacy  Life course  Illness career  Culture and climate, client/provider networks, treatment modalities, institutional policies  TREATMENT SYSTEM | In essence, the FINIS is a systems science approach. The rationale for FINIS lies in evidence, reviewed here, that stigma emanates from many societal and individual systems whose interconnections cannot be divorced from one another. They coexist in a dynamic relationship in which there is an interplay across, for example, the media, the community, and the individual. |
| Sen, 2021[44]  **Framework name:** Bronfenbrenner’s Ecological Systems Theory  Conceptual model  **Terminology:** Stigma  **Domain:** Internalized stigma,Stigma or discrimination in healthcare settings,Stigma or discrimination in law  https://www.ncbi.nlm.nih.gov/pubmed/32031498 | **Scope:** Asian American and Pacific Islander men who have sex with men in the USA  **Aim/Purpose:** To explore the manifestation of HIV stigma at the micro, meso and macro levels and how these might impact on HIV testing and HIV service utilisation  **Framework HIV target:** Asian American and Pacific Islander men who have sex with men | **Subtype:** Multilevel ecological framework of stigma  **Definition:** Stigma = an “attribute that is deeply discrediting” that reduces the stigmatised individual “from a whole and usual person to a tainted, discounted one" (Goffman)  HIV-related stigma = manifests itself through mechanisms that differ for HIV negative and HIV positive individuals (prejudice, stereotyping and discrimination; manifest as anticipated stigma, enacted stigma and internalised stigma)  **Framework specific to HIV:** Yes | Macro/structural level factors  Meso level factors  Micro/individual level factors  HIV Testing | A model which is culturally grounded and bridges the individual, interpersonal and societal conceptualisations of stigma. |
| Stangl, 2019 [47]  Stangl, 2018 [139] ; ICRW, 2013 [127]; Stangl, 2017 [138]; Stangl, 2010 [140]  **Framework name:** The Health Stigma and Discrimination Framework  Conceptual model  **Terminology:** Stigma,Discrimination  **Domain:** Internalized stigma,Stigma or discrimination in healthcare settings,Stigma or discrimination in law  https://www.ncbi.nlm.nih.gov/pubmed/30764826  International Center for Research on Women | **Scope:** Health-related stigmas  **Aim/Purpose:** To amplify our collective ability to respond effectively and at-scale to a major driver of poor health outcomes globally  **Framework HIV target:** People living with HIV | **Subtype:** Internalized, perceived stigma, associated stigma, experienced discrimination  **Definition:** Internalized or self-stigma = stigmatized group member’s own adoption of negative societal beliefs and feelings, as well as the social devaluation, associated with their stigmatized status  **Framework specific to HIV:** No | Drivers of stigma  Facilitators of stigma  Intersecting stigmas  Manifestations  Outcomes  Health and social impacts | The Health Stigma and Discrimination Framework is a global, crosscutting framework based on theory, research, and practice, which demonstrates its application to a range of health conditions, and discusses how stigma related to race, gender, sexual orientation, class, and occupation intersects with health-related stigmas, and how the framework can be used to enhance research, programming, and policy efforts. |
| Stevens, 2019 [48]  **Framework name:** Rehabilitation Framework  Conceptual model  **Terminology:** Stigma  **Domain:** Internalized stigma,Stigma or discrimination in healthcare settings,Stigma or discrimination in law  https://www.ncbi.nlm.nih.gov/pubmed/28978242 | **Scope:** Conceptualizing HIV in a rehabilitation framework  **Aim/Purpose:** To help rehabilitation professionals better understand the dynamic and nuanced forms of stigma and how they relate to rehabilitation  **Framework HIV target:** People living with HIV | **Subtype:** Enacted, self, and structural stigma  **Definition:** Enacted stigma = extent to which an individual encounters stigma from others during the course of his/her social relations with other individuals or groups  Self-stigma = arises from the internalization of negative attitudes experienced in the broader environment  Structural stigma = influenced by social institutions within the environment in which the individual lives  **Framework specific to HIV:** Broader Applying it to HIV but framework itself is very broad | Health condition  Body functions/structures (impairments)  Activity (limitations)  Participation (restriction)  Environmental factors  Personal factors  Contextual factors | Three broad spheres of stigma are described: enacted, self, and structural stigma. These three forms of stigma are then aligned in unique ways with three particular constructs of the International Classification of Functioning, Disability and Health: participation restrictions, environmental, and personal contextual factors. |
| Thapa, 2017 [50]  **Framework name:** NA  Empirical model  **Terminology:** Stigma  **Domain:** Internalized stigma,Stigma or discrimination in healthcare settings,Stigma or discrimination in law  https://www.ncbi.nlm.nih.gov/pubmed/28473183 | **Scope:** Effect of stigma-reduction intervention strategies on HIV test uptake  **Aim/Purpose:** A conceptual framework to illustrate mechanisms involved in reducing HIV stigma and increasing HIV test uptake  **Framework HIV target:** NA | **Subtype:** NA  **Definition:** Stigma = social process, experienced or anticipated, characterized by exclusion, rejection, blame, or devaluation that results from experience, perception, or reasonable anticipation of an adverse social judgment about a person or group  HIV stigma = process of devaluation of people either living with or associated with HIV infection  **Framework specific to HIV:** Yes | Awareness creation -> improve knowledge-> change attitude -> behavior change  Influencing normative behavior -> change attitude -> behavior change  Influencing normative behavior -> behavior change  Providing support -> behavior change -> reduced HIV stigma -> HIV test-uptake  Developing regulatory flaws -> behavior change -> reduced HIV stigma -> HIV test-uptake  Social-contextual factors: availability of HIV-related health services, health worker related factors, social trust, community decision, social support -> reduced HIV stigma  Social-contextual factors: availability of HIV-related health services, health worker related factors, social trust, community decision, social support -> individual factors: risk perception, interaction with PLWH, fear to test for HIV, income  Individual factors: risk perception, interaction with PLWH, fear to test for HIV, income -> HIV test up-take  Reduced HIV stigma -> individual factors: risk perception, interaction with PLWH, fear to test for HIV, income | A conceptual framework to illustrate the mechanisms of the effect of stigma-reduction interventions strategies on HIV test uptake. |
| UNAIDS, 2017 [57]  **Framework name:** Effects of Stigma and Discrimination on Health Care Access for Prevention, Testing and Treatment  Conceptual model  **Terminology:** Stigma,Discrimination  **Domain:** Internalized stigma,Stigma or discrimination in healthcare settings,Stigma or discrimination in law  https://www.unaids.org/sites/default/files/media_asset/confronting-discrimination_en.pdf  UNAIDS | **Scope:** Stigma and discrimination as health care service barriers  **Aim/Purpose:** NA  **Framework HIV target:** People living with HIV | **Subtype:** NA  **Definition:** NA  **Framework specific to HIV:** Yes | DRIVERS OF DISCRIMINATION  Societal stigma and discrimination against key populations and marginalized groups  General HIV stigma and discrimination  Age of consent laws  Criminalization of key populations  HIV criminalization  Misconceptions about HIV  MANIFESTATIONS OF DISCRIMINATION  Arrest, violence or blackmailing of key populations  Forced sterilization or abortion  Lack of appropriate and accessible  information and services  Discrimination in health care against key populations and marginalized groups  HIV discrimination in health care  Internalized stigma  Breaches of privacy  EFFECT ON HEALTH CARE  Nondisclosure of HIV status or risk behavior  Avoidance of health services  Unmet prevention needs  Reluctance to test or treat  Lack of counseling  Substandard or no care  Denial of treatment or involuntary treatment | Criminal laws, community attitudes, misinformation, prejudice and fear are all drivers of stigma and actual manifestations of discrimination affecting access to health care by people living with HIV and key populations. |
| Woodgate, 2017 [66]  **Framework name:** Social Ecological Framework  Conceptual model  **Terminology:** Stigma,Discrimination  **Domain:** Internalized stigma,Stigma or discrimination in healthcare settings,Stigma or discrimination in law  https://www.ncbi.nlm.nih.gov/pubmed/29055805 | **Scope:** Stigma and discrimination on the lives of Indigenous people who contracted HIV in their youth (i.e. 15–29 years of age) who are HIV positive within their various settings in Manitoba, Canada  **Aim/Purpose:** Developing a better structural understanding of the impacts of stigma and discrimination on the lives of Indigenous people who are HIV positive  **Framework HIV target:** Indigenous people living with HIV in Manitoba, Canada | **Subtype:** NA  **Definition:** Stigma = socially constructed negative stereotype, attitude, or belief used to produce or justify social difference and when that stigma is acted upon, it can result in discrimination, or the unfair treatment resulting from prejudice  **Framework specific to HIV:** Yes | Macrosystem (child welfare services)  Exosystem (health services)  Mesosystem (community)  Microsystem (family, home, and peer group)  Indigenous people living with HIV | Stigma and discrimination caused barriers for Indigenous people living with HIV through inhibiting their ease of access to supports including family, peers, community, and long- and short-term health services. |
| Turan, 2017 [55]  **Framework name:** Conceptual Framework for HIV-Related Stigma, Engagement in Care, and Health Outcomes  Conceptual model  **Terminology:** Stigma  **Domain:** Internalized stigma,Stigma or discrimination in law  https://www.ncbi.nlm.nih.gov/pubmed/28426316 | **Scope:** Adherence to treatment, health outcomes  **Aim/Purpose:** A conceptual framework for individual-level dimensions of stigma and potential individual and interpersonal mechanisms explaining how stigma affects HIV-related health  **Framework HIV target:** People living with HIV | **Subtype:** Perceived community stigma, experienced stigma, internalized stigma, anticipated stigma, structural stigma, intersectional stigmas  **Definition:** Stigma = pervasive social process: individuals with socially undesirable attributes or identities are seen as having lower social value than are others and as a consequence face prejudice and discrimination; the attribute that is the target of stigmatization may be a particular racial or ethnic background, poverty, a chronic disease, sexual orientation, or any other characteristic that is interpreted in society as a sign of the flawed or inferior character of the person  Structural stigma = attitudes in societies, practices, structures, services, and laws that work to the disadvantage of minority groups  Intersectional stigma = tendency for people living with HIV to simultaneously experience stigma and discrimination because of HIV and other aspects of their identities, such as their race, economic situation, or sexual orientation  **Framework specific to HIV:** Yes | Structural stigma  Intersectional stigmas  Enacted  Community  Anticipated  Internalized  Interpersonal factors  Psychological resources  Mental health  Stress processes  ART adherence  Retention in care  CD4 count  Viral load | In the context of intersectional and structural stigmas, individual-level dimensions of HIV-related stigma operate through interpersonal factors, mental health, psychological resources, and biological stress pathways. |
| Davtyan, 2017 [10]  **Framework name:** Model of HIV-Related Stigma in Health Care Settings  Empirical model  **Terminology:** Stigma  **Domain:** Stigma or discrimination in healthcare settings  https://www.ncbi.nlm.nih.gov/pubmed/28830704 | **Scope:** Stigma in health care settings  **Aim/Purpose:** Used Grounded Theory to understand how health care workers conceptualized HIV-related stigma and to develop a model to project a purposive view of stigma in health care settings  **Framework HIV target:** People living with HIV | **Subtype:** NA  **Definition:** NA  **Framework specific to HIV:** Yes | Origin of HIV-related stigma: historically negative depiction of HIV  Unequal provider-patient power dynamics  Triggers of HIV-related stigma: fear, education and training, inadvertent behaviors, contact with patients with HIV  Resulting stigmatizing feeling and behaviors: care refusal, nervousness, anxiety, misconceptions, assumptions about mode of transmission, patient neglect, detachment, referrals  Impact of HIV-related stigma on patients: treatment engagement, psychological function | Stigma may be rooted in historically derogatory representations of HIV and intensified by power inequalities; stigma may be triggered by fear, inadequate clinical education and training, unintentional behaviors, and limited contact with people living with HIV. |
| Ikeda, 2019 [21]  **Framework name:** NA  Conceptual model  **Terminology:** Stigma,Discrimination  **Domain:** Stigma or discrimination in healthcare settings  https://www.ncbi.nlm.nih.gov/pubmed/31297246 | **Scope:** Quality improvement and stigma and discrimination  **Aim/Purpose:** Linking quality improvement capacity building to reduction of stigma and discrimination  **Framework HIV target:** People living with HIV | **Subtype:** Stigma and discrimination  **Definition:** NA  **Framework specific to HIV:** Yes | IMPACT  Sustained achievement of 95-95-95 targets through improvements in testing, linkage, care engagement, adherence  LONG-TERM OUTCOMES  Reduction in HIV-related stigma and discrimination in healthcare settings; development of enabling clinic environments  INTERMEDIATE OUTCOMES  Regional exchange to generate strategies and policy innovations to spur national adoption  Increased capacity of national cadres to monitor stigma and discrimination; scale effective interventions  Increased capacity of facility cadres to use data and QI methods to address stigma and discrimination  OUTPUTS  Accumulation of effective interventions  Peer learning and exchange  Increased knowledge of QI concepts, tools  Increased knowledge of stigma and discrimination and its drivers  Ongoing support of key stakeholders  ACTIVITIES  Engagement/design meetings  Facility sensitization  Exchange meetings  Coaching visits  Routine data collection  Knowledge management  Small-scale tests of change  INPUTS  UCSF- Healthqual  Ministries of Health  PLWH groups  Implementing partners  Funding | Sustained achievement of desired outcomes is achieved by building the capacity of national-level, district-level and facility-level cadres to continuously monitor and improve the quality of healthcare service delivery. |
| Jain, 2012 [22]  **Framework name:** Framework for Addressing and Measuring Stigma & Discrimination in Healthcare Facilities  **Terminology:** Stigma,Discrimination  **Domain:** Stigma or discrimination in healthcare settings  https://www.healthpolicyproject.com/pubs/66_WorkingPaperStigmaScaleUpMeasurementJuly.pdf  Health Policy Project | **Scope:** Stigma and discrimination in healthcare facilities  **Aim/Purpose:** To help policymakers and programmers understand the pathways through which stigma and discrimination contribute to outcomes and impacts and specific entry points for programmatic intervention and measurement  **Framework HIV target:** Healthcare staff, healthcare facilities | **Subtype:** Anticipated, experienced, secondary; discrimination  **Definition:** Stigma = social process of devaluing persons, beginning with marking or labeling of differences, attributing negative connotations or values to those differences, leading to distancing and separation of the person and culminating in discrimination  Anticipated stigma = real or imagined fears of societal (e.g., family, community, healthcare professionals) attitudes and behaviors if HIV or other stigmatized behavior (e.g., drug use) is disclosed  Experienced stigma = forms of stigmatizing behaviors or discrimination that are not typically actionable under law and experienced by people living with HIV or individuals associated with HIV, such as family members or healthcare providers  Secondary stigma = stigma experienced by individuals who are associated with people living with HIV (e.g., family, partners, friends, healthcare professionals)  Internalized stigma = acceptance by the self that the external stigma is true and justified—of society’s judgment of oneself as being of a “lesser status.”  Compound/Layered stigma = experience of multiple stigmas (e.g., stigma toward men who have sex with men, transgenders, migrants, poor women, people who inject drugs plus HIV stigma)  Observed stigma = forms of stigma witnessed by an individual (e.g., nurse gossiping about a client’s HIV status as seen by a lab technician)  Discrimination = unfair and unjust treatment of an individual on the basis of a real or perceived status or attribute (e.g., HIV status or association with HIV positive individuals) Discrimination = typically actionable under law  **Framework specific to HIV:** Yes | Actionable drivers to be addressed in healthcare facilities (stigma and discrimination toward clients and healthcare staff): individual health facility staff level; institutional level  Reduced stigma manifestations  Improvement outcomes  Improved impacts | To capture the importance of programmatic interventions at both the individual staff level and institutional or environmental level, the actionable drivers for reducing stigma and discrimination are divided into two sections: measurement of the key drivers at both these levels is essential to supporting effective programming. The first section focuses on key drivers to address at the individual level. Example interventions to address these drivers include creating awareness and understanding of how stigma and discrimination manifests in healthcare facilities and the impact; deepening knowledge of HIV transmission and prevention to address unwarranted fears of transmission; and addressing attitudes and social judgments about clients living with or affected by HIV. |
| Meyerson, 2014 [32]  **Framework name:** NA  Empirical model  **Terminology:** Stigma,Discrimination  **Domain:** Stigma or discrimination in healthcare settings  https://www.ncbi.nlm.nih.gov/pubmed/24313812 | **Scope:** Institutional and structural expressions of stigma in HIV testing  **Aim/Purpose:** To examine evidence of institutional and structural stigma in the HIV testing process  **Framework HIV target:** People seeking an HIV test | **Subtype:** NA  **Definition:** Institutional discrimination = similar to structural discrimination, but is defined by intentionality: disparity is intended for particular populations, whereas structural discrimination creates disparity unintentionally  **Framework specific to HIV:** Yes | Labeling  Stereotyping  STIGMA  Prejudice  Discrimination | Examples of structural stigma included social geography, organization, and staff behavior at first encounter and reception, and staff behavior when experiencing the actual HIV test. Institutional stigma was socially expressed through staff behavior at entry/reception and when experiencing the HIV test. The emerging elements demonstrate the potential compounding of stigma experiences with deleterious effect. |
| Nyblade, 2020 [35]  Health Policy Project [126]  **Framework name:** Three-Stage ‘Total Facility’ Stigma-Reduction Approach  Conceptual model  **Terminology:** Stigma  **Domain:** Stigma or discrimination in healthcare settings  https://www.ncbi.nlm.nih.gov/pubmed/32881798  RTI International | **Scope:** Health facility HIV stigma in Ghana and Tanzania  **Aim/Purpose:** Three-stage ‘total facility’ approach to reduce health facility HIV stigma  **Framework HIV target:** People living with HIV | **Subtype:** NA  **Definition:** Stigma = social process through which an individual’s worth and social standing are eroded on the basis of a perceived association with socially unacceptable behaviors or marginalized groups, resulting in a loss of status and discrimination  **Framework specific to HIV:** No | Sociological levels addressed: Individual, Interpersonal, Facility/institutional  Formative research  Capacity building  Integration into facility structures and processes | The total facility approach adapts a socioecological model to the individual health facility environment, working with the health facility to develop stigma reduction intervention activities that address stigma at multiple socioecological levels: individual health workers, with a focus on client interactions; the interpersonal level of how staff work together and interact with each other across departments and staff cadres; and the institutional/ health facility level. |
| Pham, 2012 [38]  **Framework name:** Adjusted Spector’s Job Satisfaction Model  Empirical model  **Terminology:** Stigma  **Domain:** Stigma or discrimination in healthcare settings  https://www.ncbi.nlm.nih.gov/pubmed/23259923 | **Scope:** Stigma as a source of dissatisfaction among healthcare workers in Vietnam  **Aim/Purpose:** To suggest an adjusted Spector’s model of the job satisfaction of HIV service health workers in Vietnam  **Framework HIV target:** People living with HIV | **Subtype:** NA  **Definition:** NA  **Framework specific to HIV:** Yes | JOB SATISFACTION  Stigma  Pay  Promotion  Supervision  Fringe benefits  Contingent rewards  Operating procedures  Co-workers  Nature of work  Communication | This study confirmed the relationship between stigmatization of PLHIV and stigma experienced by staff because of association with PLHIV from families, colleagues, and society. The experiencing stigma results in additional work-related stress, low self-esteem, poor views of their profession, and lower income. |
| Pulerwitz, 2015 [40]  **Framework name:** Conceptual Framework for HIV Stigma  Conceptual model  **Terminology:** Stigma  **Domain:** Stigma or discrimination in healthcare settings  https://www.ncbi.nlm.nih.gov/pubmed/25382350 | **Scope:** Stigma in Vietnamese society  **Aim/Purpose:** A conceptual framework to understand the relationship among the types of stigma, and factors resulting in stigma  **Framework HIV target:** People living with HIV | **Subtype:** Fear-based, value-based, enacted  **Definition:** Stigma = attribute that is deeply discrediting within a particular social interaction  HIV stigma = pattern of prejudice, discounting, discrediting and discrimination directed at people perceived to have HIV, their significant others and close associates, and their social groups and communities  **Framework specific to HIV:** Yes | Precursors  Fear-based stigma  Value-based stigma  Enacted stigma | There are multiple types of stigma and several underlying sources. |
| Thrasher, 2008 [52]  **Framework name:** NA  Empirical model  **Terminology:** Stigma,Discrimination  **Domain:** Stigma or discrimination in healthcare settings  https://www.ncbi.nlm.nih.gov/pubmed/18667919 | **Scope:** Healthcare discrimination/distrust, and racial/ethnic disparities on antiretroviral therapy adherence  **Aim/Purpose:** To assess the extent to which discriminatory health care experiences and health care provider distrust influence treatment related attitudes, beliefs, and self-reported adherence in a national sample of HIV-infected patients  **Framework HIV target:** HIV-infected patients | **Subtype:** NA  **Definition:** NA  **Framework specific to HIV:** Yes | Racial/ethnic minority status  Discriminatory healthcare experiences  Healthcare provider distrust  Psychological burden of medication  Weak medication efficacy beliefs  Difficulty accessing medication  Difficulty scheduling medication  Antiretroviral therapy adherence | More discrimination predicted greater distrust, weaker treatment benefit beliefs, and, in turn, poorer adherence. Distrust affected adherence by increasing treatment-related psychological distress and weakening treatment benefit beliefs. |
| UNAIDS, 2017 [58]  **Framework name:** Examples of Stigma and Discrimination that Create Gaps Across the HIV Testing and Treatment Cascade  Conceptual model  **Terminology:** Stigma,Discrimination  **Domain:** Stigma or discrimination in healthcare settings  https://www.unaids.org/sites/default/files/media_asset/confronting-discrimination_en.pdf  UNAIDS | **Scope:** Healthcare stigma that creates gaps in HIV testing and treatment cascade  **Aim/Purpose:** NA  **Framework HIV target:** People living with HIV | **Subtype:** NA  **Definition:** NA  **Framework specific to HIV:** Yes | EXAMPLES OF BARRIERS SPECIFIC TO ONE STEP OF THE CASCADE  All people with HIV: Fear of lack of confidentiality among health-care workers undermined HIV testing uptake in sub-Saharan African countries; men who have sex with men and transgender women in New York City who feared HIV stigma were less likely to have had an HIV test in the previous six months  People living with HIV who know their HIV status: People living with HIV who experienced high HIV stigma were 2.4 times more likely to present late for HIV care; HIV-related stigma inhibits uptake of services to prevent mother-to-child transmission; fear of disclosing HIV-positive status to their male partner and stigma identified as key barriers to initiating lifelong treatment among pregnant women living with HIV in three districts in Uganda  People living with HIV who are accessing antiretroviral therapy; People living with HIV who are virally suppressed: Stigma or fear of stigma leading to non-disclosure undermine treatment adherence by compromising social support mechanisms  BARRIERS THAT EXIST ACROSS THE CASCADE  Societal fear of HIV infection  Negative attitudes towards key populations  Stigmatizing attitudes and practices among health-care workers towards people living with HIV, people at high risk of HIV infection and caregivers  Denial of health services to people living with HIV and marginalized groups  Lack of family and community support | When health systems or health-care workers adopt stigmatizing attitudes and practices or discriminate against people living with HIV or people at high risk of HIV infection, those people are less likely to access services, hampering efforts to reach the 90–90–90 treatment targets. |
| Williams, 2020 [65]  **Framework name:** NA  Empirical model  **Terminology:** Stigma  **Domain:** Stigma or discrimination in healthcare settings  https://www.ncbi.nlm.nih.gov/pubmed/30798458 | **Scope:** Multi-level framework of stigma in Sub-Saharan Africa  **Aim/Purpose:** To examine multilevel relationships of HIV/AIDS-related stigma to health service barriers and HIV outcomes in KwaZulu-Natal, South Africa  **Framework HIV target:** People living with HIV | **Subtype:** NA  **Definition:** NA  **Framework specific to HIV:** Yes | Individual level: stigmatizing attitudes towards people with HIV, perceptions of comm. normative HIV-related stigma  Household level: HSBs, household HIV ration  Near-neighbor cluster level: stigmatizing attitudes towards people with HIV/AIDS, HSB, HIV prevalence, comm. normative HIVAIDS-related stigma  Community level: stigmatizing attitudes towards people with HIV/AIDS, comm. normative HIV/AIDs-related stigma | Diferential patterns of prediction suggest that HIV/AIDS-related (HAR) stigma stigma is a multi-level construct with multiple dimensions that relate to important outcomes diferently within and across multiple ecological levels. |
| Yang, 2008 [67]  **Framework name:** Stigma Model for China  Conceptual model  **Terminology:** Stigma  **Domain:** Stigma or discrimination in healthcare settings  https://www.ncbi.nlm.nih.gov/pubmed/18420325 | **Scope:** Changes in moral status to describe how stigma operate in China  **Aim/Purpose:** To propose a three-layered model to conceptualize how societal features of stigma and changes in moral status result in adverse local outcomes in China  **Framework HIV target:** People living with HIV | **Subtype:** Subjective, collective, interpersonal  **Definition:** NA  **Framework specific to HIV:** Broader Schizophrenia and AIDS | SOCIETAL FACTORS INFLUENCING STIGMA  Public conceptions of stigmatized conditions  Institutional forms of stigma- field or structures of capital  CHANGE IN MORAL ASPECT OF STIGMA  Loss of face ('symbolic capital')  CHANGES IN SUBJECTIVE, COLLECTIVE, AND INTERPERSONAL ASPECTS OF STIGMA  Subjective/individual aspects: moral-emotional (i.e emotional reactions), moral- somatic (i.e physical manifestations)  Collective aspects: between family members, between social network members  Interpersonal aspects: physician stigma, family member status, loss of guanxi (or access to 'social capital' | NA |
| Yu, 2016 [68]  **Framework name:** Conceptual Framework of Factors Predicting Dignity-Related Distress in Patients with HIV  Empirical model  **Terminology:** Stigma,Discrimination  **Domain:** Stigma or discrimination in healthcare settings  https://www.ncbi.nlm.nih.gov/pubmed/26829259 | **Scope:** Health care neglect and perceived discrimination in dignity-related distress among Chinese people living with HIV  **Aim/Purpose:** To examine the roles of health care neglect and perceived discrimination in dignity-related distress  **Framework HIV target:** Chinese people living with HIV | **Subtype:** Perceived discrimination  **Definition:** NA  **Framework specific to HIV:** Yes | VARIABLES IN THE CLINIC SETTING -> DIGNITY RELATED DISTRESS  Psychological symptoms  Physical symptoms  Healthcare neglect  VARIABLES FROM THE GENERAL BACKGROUND -> DIGNITY RELATED DISTRESS  Perceived discrimination | The results showed that psychological symptoms, physical symptoms, and health care neglect not only independently predicted dignity-related distress, but also moderated with each other to show a three-way interaction. Perceived discrimination also explained the variance in dignity-related distress. |
| UNAIDS, 2017 [59]  **Framework name:** Examples of Stigma and Discrimination that Create Gaps Across the HIV Prevention Cascade  Conceptual model  **Terminology:** Stigma,Discrimination  **Domain:** Stigma or discrimination in healthcare settings,Stigma or discrimination in law  https://www.unaids.org/sites/default/files/media_asset/confronting-discrimination_en.pdf  UNAIDS | **Scope:** Healthcare stigma and discrimination that create gaps across the HIV prevention cascade  **Aim/Purpose:** NA  **Framework HIV target:** People living with HIV | **Subtype:** NA  **Definition:** NA  **Framework specific to HIV:** Yes | BARRIERS SPECIFIC TO ONE STEP OF THE CASCADE  All people at risk of acquiring HIV: Transgender women in Argentina were three times more likely to avoid health care if previously discriminated by healthcare workers; female sex workers in Kenya who feared stigma from health-care workers were twice as likely to avoid non-HIV care; fear of stigma has been identified as a barrier to pre-exposure prophylaxis initiation  People at risk of acquiring HIV who know of and have an intention to use prevention mechanisms: Criminalization of drug use has a negative effect on HIV prevention and treatment; Signing into law of the SameSex Marriage Prohibition Act criminalizing same-sex sexual relationships in Nigeria led to increased fear of accessing health care among men who have sex with men; Criminalization of same-sex sexual acts in Senegal led to decreased participation in HIV prevention by men who have sex with men; denial of prevention services to specific populations (e.g. undocumented migrants); denial of sexual and reproductive health rights  Availability of prevention mechanisms to people at risk of acquiring HIV  People at risk of acquiring HIV consistently using available prevention mechanisms and remaining HIV negative  BARRIERS THAT EXIST ACROSS THE CASCADE  Societal fear of HIV infection  Negative attitudes towards key populations  Criminalization of key populations  Criminalization of HIV transmission  Violence and the threat of violence | The framework describes examples of stigma and discrimination that create gaps across the HIV prevention cascade |
| UNAIDS, 2017 [60]  **Framework name:** Removing Stigma and Discrimination Improves Health Care Access for Prevention, Testing and Treatment  Conceptual model  **Terminology:** Stigma,Discrimination  **Domain:** Stigma or discrimination in healthcare settings,Stigma or discrimination in law  https://www.unaids.org/sites/default/files/media_asset/confronting-discrimination_en.pdf  UNAIDS | **Scope:** Confronting stigma to remove healthcare stigma barriers  **Aim/Purpose:** NA  **Framework HIV target:** People living with HIV | **Subtype:** NA  **Definition:** NA  **Framework specific to HIV:** Yes | REDUCING DISCRIMINATION  Standards for health professionals  Educate health-service providers  Rights education for people living with HIV and key populations  Monitoring health services  Decriminalization of key populations  Protective laws  Accountability in courts and tribunals  Legal advice and representation  MANIFESTATIONS OF REDUCED DISCRIMINATION  Reduction in internalized stigma  Legal protection against discrimination and violence  Confidentiality  Nondiscriminatory health care  Respectful health services  Accessible and appropriate information and services  EFFECT ON HEALTHCARE  Quality health care  Increased use of health services  Disclosure of risk behaviors and HIV status to health services  Increased uptake of testing and treatment | By addressing drivers, removal of harmful laws, introduction of protective laws, education of rights holders and service providers and legal empowerment of communities to defend their rights, the gap between those who can access services and those who cannot can be closed, leading to better health for all. |
| Hagopian, 2017 [19]  **Framework name:** NA  Conceptual model  **Terminology:** Stigma,Discrimination  **Domain:** Stigma or discrimination in law  https://www.ncbi.nlm.nih.gov/pubmed/28580879 | **Scope:** Pathways in which anti-homosexuality laws lead to increased incidence of HIV in a population  **Aim/Purpose:** To explore how the US President's Emergency Plan for AIDS Relief may have used its influence to reduce the criminalization of homosexuality in the countries where it operated  **Framework HIV target:** Gay men and other men who have sex with men in African nations | **Subtype:** NA  **Definition:** NA  **Framework specific to HIV:** Yes | Homonegativity  Anti-homosexuality laws  HIV stigma  Avoidance of VCT  Poor treatment compliance  Failure to disclose  Increased HIV | The punitive legal and social environments have served to fuel the HIV epidemic through the mechanisms illustrated in the framework. |
| Katz, 2013 [23]  **Framework name:** Reciprocal Relationships Between Poverty and Stigma  Empirical model  **Terminology:** Stigma  **Domain:** Stigma or discrimination in law  https://www.ncbi.nlm.nih.gov/pubmed/24242258 | **Scope:** Poverty and stigma  **Aim/Purpose:** NA  **Framework HIV target:** HIV-positive persons | **Subtype:** NA  **Definition:** NA  **Framework specific to HIV:** Yes | Illness -> economic inadequacy -> exclusion  Stigma -> exclusion -> food and livelihood insecurity | HIV-associated illness reinforces the perceived economic inadequacy of HIV-positive persons, who are excluded from networks of mutual aid; stigmatized persons are excluded from the community, undermining their social support and worsening economic insecurity. |
| Mahajan, 2008 [31]  **Framework name:** Conceptual Framework for HIV/AIDS Related Stigma  Conceptual model  **Terminology:** Stigma,Discrimination  **Domain:** Stigma or discrimination in law  https://www.ncbi.nlm.nih.gov/pubmed/18641472 | **Scope:** Inequalities and stigma  **Aim/Purpose:** S schematic that illustrates a starting point for a conceptual framework for HIV/AIDS related stigma, derived from this review of the literature  **Framework HIV target:** People living with HIV | **Subtype:** NA  **Definition:** NA  **Framework specific to HIV:** Yes | POWER  Structural violence  Pre-existing stigma  LABELING  STEREOTYPING  SEPARATING AND STATUS LOSS  DISCRIMINATION  Self-imposed  Individual discrimination  Structural/institutional discrimination | Inequalities in social, political, and economic power are the foundation on which stigmatization is promulgated. For HIV/AIDS related stigma, structural violence and pre-existing stigmas potentiate the power of stigmatizers and enable even more intense stigmatization and discrimination. Stigma exists when labeling, stereotyping, separation/status loss, and discrimination in the setting of power imbalance simultaneously converge. |

**Appendix Table 2. Critical appraisal frameworks**

| **Author, year** | **Framework name** | **Source** | **Stakeholder involvement** | **Evidence based** | **Defined population** | **Validity tested** |
| --- | --- | --- | --- | --- | --- | --- |
| Boyes, 2020 [1] | NA | Unclear | Unclear | Low risk of bias | Low risk of bias | Low risk of bias |
| Casale, 2019 [2] | NA | Unclear | Unclear | Low risk of bias | Low risk of bias | Unclear |
| Chan, 2019 [3] | NA | Unclear | Unclear | Low risk of bias | Unclear | Low risk of bias |
| Christopoulos, 2020 [4] | NA | Unclear | Unclear | Low risk of bias | High risk of bias | Low risk of bias |
| Clum, 2009 [5] | NA | Unclear | Unclear | Low risk of bias | Low risk of bias | Low risk of bias |
| Cluver, 2013 [6] | NA | Unclear | Unclear | Low risk of bias | Low risk of bias | Low risk of bias |
| Colbert, 2010 [7] | NA | Unclear | Unclear | Low risk of bias | High risk of bias | Low risk of bias |
| Crockett, 2019 [8] | NA | Unclear | Unclear | Low risk of bias | Unclear | Unclear |
| Darlington, 2017 [9] | Current State-of-Science of HIV-Related Stigma Among HIV + Women in the Southern US | Unclear | Unclear | Low risk of bias | Low risk of bias | Unclear |
| Davtyan, 2017 [10] | Model of HIV-Related Stigma in Health Care Settings | Unclear | Unclear | Low risk of bias | Low risk of bias | Low risk of bias |
| Deacon, 2005 [11] | How Different Kinds of Stigma and Discrimination Relate to Each Other | Unclear | Unclear | Unclear | High risk of bias | Unclear |
| Diiorio, 2009 [12] | Model of Adherence | Unclear | Unclear | Low risk of bias | Unclear | Low risk of bias |
| Earnshaw, 2013 [14] | Hypothesized associations between HIV stigma mechanisms and health and well-being among people living with HIV | Low risk of bias | Unclear | Low risk of bias | Unclear | Low risk of bias |
| Earnshaw, 2013 [13] | Stigma and HIV Disparities Model | Unclear | Unclear | Low risk of bias | High risk of bias | Low risk of bias |
| Fekete, 2018 [15] | NA | Unclear | Unclear | Low risk of bias | High risk of bias | Unclear |
| Florom-Smith, 2012 [16] | External, Internal, and Consequences of HIV-Related Stigma | Unclear | Unclear | Low risk of bias | High risk of bias | Unclear |
| France, 2015 [17] | NA | Unclear | Unclear | Low risk of bias | Unclear | Unclear |
| Gilbert, 2016 [18] | NA | High risk of bias | High risk of bias | Unclear | High risk of bias | High risk of bias |
| Hagopian, 2017 [19] | NA | Unclear | Unclear | Unclear | High risk of bias | Unclear |
| Health Policy Plus, 2017 [20] | Stigma Drivers and Manifestations: Relationship to the HIV Treatment Cascade | Low risk of bias | Unclear | Unclear | High risk of bias | Unclear |
| Ikeda, 2019 [21] | NA | Unclear | Low risk of bias | Unclear | Low risk of bias | Unclear |
| Jain, 2012 [22] | Framework for Addressing and Measuring Stigma & Discrimination in Healthcare Facilities | Low risk of bias | Low risk of bias | Low risk of bias | Low risk of bias | Unclear |
| Katz, 2013 [24] | NA | Unclear | Unclear | Low risk of bias | High risk of bias | Unclear |
| Katz, 2013 [23] | Reciprocal Relationships Between Poverty and Stigma | Unclear | Unclear | Low risk of bias | High risk of bias | Low risk of bias |
| Lipira, 2019 [25] | NA | Unclear | Unclear | Low risk of bias | Low risk of bias | Unclear |
| Logie, 2016 [27] | NA | Unclear | Unclear | Low risk of bias | Low risk of bias | Low risk of bias |
| Logie, 2017 [26] | Factors Contributing to Quality of Life Among African and Caribbean Women Living with HIV in Ontario | Unclear | Unclear | Low risk of bias | Low risk of bias | Low risk of bias |
| Logie, 2018 [29] | HIV-Related Stigma, Racial Discrimination, and Gender Discrimination on Health-Related Quality of Life (Mental and Physical Health) Among Women Living with HIV in Canada (2013-2015) | Unclear | Unclear | Low risk of bias | Low risk of bias | Low risk of bias |
| Logie, 2018 [28] | NA | Unclear | Unclear | Low risk of bias | Low risk of bias | Low risk of bias |
| Logie, 2019 [30] | NA | Unclear | Unclear | Low risk of bias | Low risk of bias | Low risk of bias |
| Mahajan, 2008 [31] | Conceptual Framework for HIV/AIDS Related Stigma | Unclear | Unclear | Low risk of bias | High risk of bias | Unclear |
| Meyerson, 2014 [32] | NA | Unclear | Unclear | Unclear | High risk of bias | Unclear |
| Miller, 2016 [33] | NA | Unclear | Unclear | Low risk of bias | Unclear | Low risk of bias |
| Mo, 2015 [34] | NA | Unclear | Unclear | Low risk of bias | Low risk of bias | Low risk of bias |
| Nyblade, 2020 [35] | Three-Stage ‘Total Facility’ Stigma-Reduction Approach | Low risk of bias | Unclear | Low risk of bias | Unclear | Unclear |
| Pantelic, 2017 [36] | NA | Unclear | Unclear | Low risk of bias | Low risk of bias | Low risk of bias |
| Pescosolido, 2015 [37] | Framework Integrating Normative Influences on Stigma (FINIS) | High risk of bias | High risk of bias | Unclear | High risk of bias | High risk of bias |
| Pham, 2012 [38] | Adjusted Spector’s Job Satisfaction Model | Unclear | Unclear | Unclear | Low risk of bias | Unclear |
| Prati, 2016 [39] | Conceptual Models for HIV Uninfected Individuals and People Living with HIV/AIDS | Unclear | Unclear | Low risk of bias | Unclear | Unclear |
| Pulerwitz, 2015 [40] | Conceptual Framework for HIV Stigma | Unclear | Unclear | Unclear | Unclear | Unclear |
| Rao, 2012 [41] | NA | Unclear | Unclear | Low risk of bias | Unclear | Low risk of bias |
| Relf, 2019 [42] | NA | Unclear | Unclear | Low risk of bias | Low risk of bias | Low risk of bias |
| Rice, 2017 [43] | NA | Unclear | Unclear | Low risk of bias | Unclear | Unclear |
| Sen, 2021[44] | Bronfenbrenner’s Ecological Systems Theory | Unclear | High risk of bias | Unclear | High risk of bias | Unclear |
| Shin, 2018 [45] | NA | Unclear | Unclear | Low risk of bias | Unclear | Unclear |
| Slater, 2015 [46] | NA | Unclear | Unclear | Low risk of bias | Low risk of bias | Unclear |
| Stangl, 2019 [47] | The Health Stigma and Discrimination Framework | Low risk of bias | Unclear | Low risk of bias | Unclear | Low risk of bias |
| Stevens, 2019 [48] | Rehabilitation Framework | Unclear | Unclear | Unclear | High risk of bias | Unclear |
| Steward, 2008 [49] | Theoretical Framework Linking Facets of Stigma, Avoidance of HIV Serostatus Disclosure, and Psychological Distress | Unclear | Unclear | Low risk of bias | Unclear | Unclear |
| Thapa, 2017 [50] | NA | Unclear | Unclear | Low risk of bias | Unclear | Low risk of bias |
| Thi, 2008 [51] | NA | Unclear | Unclear | Low risk of bias | Unclear | Unclear |
| Thrasher, 2008 [52] | NA | Unclear | Unclear | Low risk of bias | Unclear | Low risk of bias |
| Tsai. 2013 [53] | NA | Unclear | Unclear | Unclear | High risk of bias | Unclear |
| Turan, 2016 [56] | NA | Unclear | Unclear | Low risk of bias | Unclear | Unclear |
| Turan, 2017 [55] | Conceptual Framework for HIV-Related Stigma, Engagement in Care, and Health Outcomes | Unclear | High risk of bias | Unclear | High risk of bias | High risk of bias |
| Turan, 2017 [54] | Modified Health Stigma Framework | Unclear | Unclear | Low risk of bias | Unclear | Unclear |
| UNAIDS, 2017 [58] | Examples of Stigma and Discrimination that Create Gaps Across the HIV Testing and Treatment Cascade | Low risk of bias | Unclear | Unclear | Low risk of bias | Unclear |
| UNAIDS, 2017 [57] | Effects of Stigma and Discrimination on Health Care Access for Prevention, Testing and Treatment | Low risk of bias | Unclear | Unclear | High risk of bias | Unclear |
| UNAIDS, 2017 [59] | Examples of Stigma and Discrimination that Create Gaps Across the HIV Prevention Cascade | Low risk of bias | Unclear | Unclear | Low risk of bias | Unclear |
| UNAIDS, 2017 [60] | Removing Stigma and Discrimination Improves Health Care Access for Prevention, Testing and Treatment | Low risk of bias | Unclear | Unclear | High risk of bias | Unclear |
| Vyavaharkar, 2010 [61] | NA | Unclear | Unclear | Low risk of bias | Low risk of bias | Unclear |
| Wardell, 2018 [62] | NA | Unclear | Unclear | Low risk of bias | Unclear | Low risk of bias |
| Watt, 2020 [63] | NA | Unclear | Low risk of bias | Low risk of bias | Low risk of bias | Low risk of bias |
| Williams, 2014 [64] | NA | Unclear | Unclear | Low risk of bias | Unclear | Unclear |
| Williams, 2020 [65] | NA | Unclear | Unclear | Unclear | Unclear | Unclear |
| Woodgate, 2017 [66] | Social Ecological Framework | Unclear | Low risk of bias | Unclear | Low risk of bias | Unclear |
| Yang, 2008 [67] | Stigma Model for China | Unclear | High risk of bias | Unclear | High risk of bias | Unclear |
| Yu, 2016 [68] | Conceptual Framework of Factors Predicting Dignity-Related Distress in Patients with HIV | Unclear | Unclear | Unclear | Unclear | Unclear |
| Zang, 2014 [69] | NA | Unclear | Unclear | Low risk of bias | Unclear | Low risk of bias |

**Framework Compendium**

Boyes ME, Pantelic M, Casale M, Toska E, Newnham E, Cluver LD. Prospective associations between bullying victimisation, internalised stigma, and mental health in South African adolescents living with HIV. *J Affect Disord.* 2020;276:418-423. <https://www.ncbi.nlm.nih.gov/pubmed/32871672>


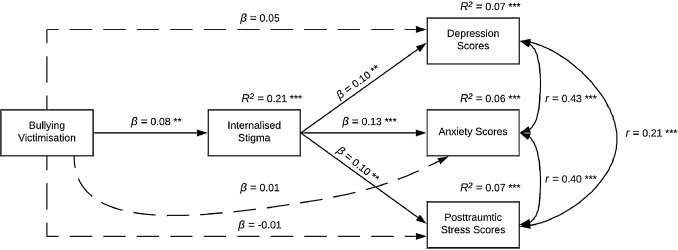


Associations between bullying victimisation, internalised stigma, and symptoms of depression, anxiety, and posttraumatic stress (Note: Bullying victimisation and internalised stigma measured at baseline assessment. Depression, anxiety, and posttraumatic stress scores measured at follow up assessment. *** p < 0.001 ** p < 0.01. Solid lines = significant associations. Dashed lines = non-significant associations).

Casale M, Boyes M, Pantelic M, Toska E, Cluver L. Suicidal thoughts and behaviour among South African adolescents living with HIV: Can social support buffer the impact of stigma? J Affect Disord. 2019;245:82-90. https://www.ncbi.nlm.nih.gov/pubmed/30368074
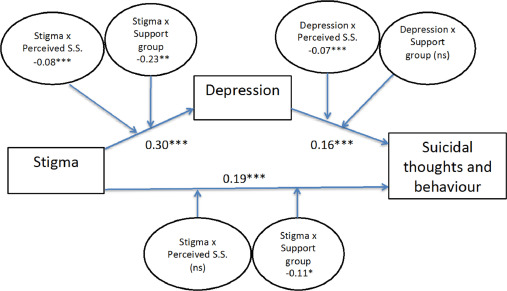


Results of moderated mediation analysis (n = 1053). The figure displays unstandardized coefficients and significance levels for individual associations and interaction terms, generated from the moderated mediation analysis. The ovalz shapes contain results of interaction terms or moderators tested. The conditional direct and indirect pathway effects of stigma on suicidal thoughts and behaviour at different values of the moderators are displayed in [Table 5](https://www-sciencedirect-com.libproxy2.usc.edu/science/article/pii/S0165032717324898#tbl0005). Lower (LLCI) and upper (ULCI) confidence intervals that do not cross zero denote significant effects at the specific indicated values of the moderators. *** p < .001; ** p < .01; * p < .05.

Chan RCH, Mak WWS. Cognitive, Regulatory, and Interpersonal Mechanisms of HIV Stigma on the Mental and Social Health of Men Who Have Sex With Men Living With HIV. *Am J Mens Health.* 2019;13(5):1557988319873778. <https://www.ncbi.nlm.nih.gov/pubmed/31690214>


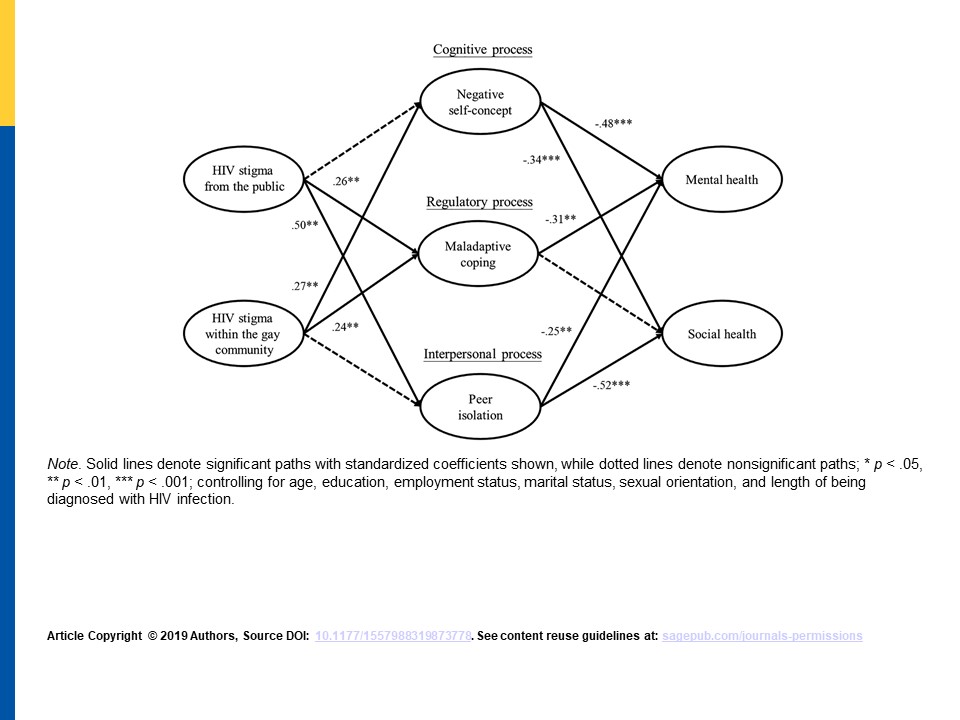


Christopoulos KA, Neilands TB, Dilworth S, et al. Internalized HIV stigma predicts subsequent viremia in US HIV patients through depressive symptoms and ART adherence. *Aids.* 2020;34(11):1665-1671. <https://www.ncbi.nlm.nih.gov/pubmed/32769764>

Measurement time points for internalized HIV stigma, depressive symptoms, antiretroviral adherence and viral load.


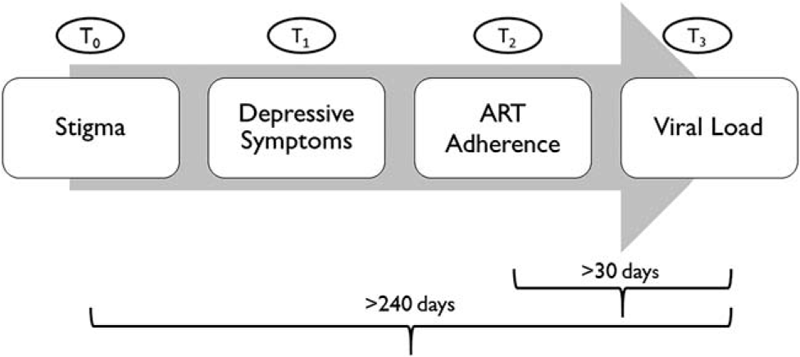


Longitudinal sequential path model of the association between internalized HIV stigma and unsuppressed viral load through the mediators of depressive symptoms and antiretroviral adherence.


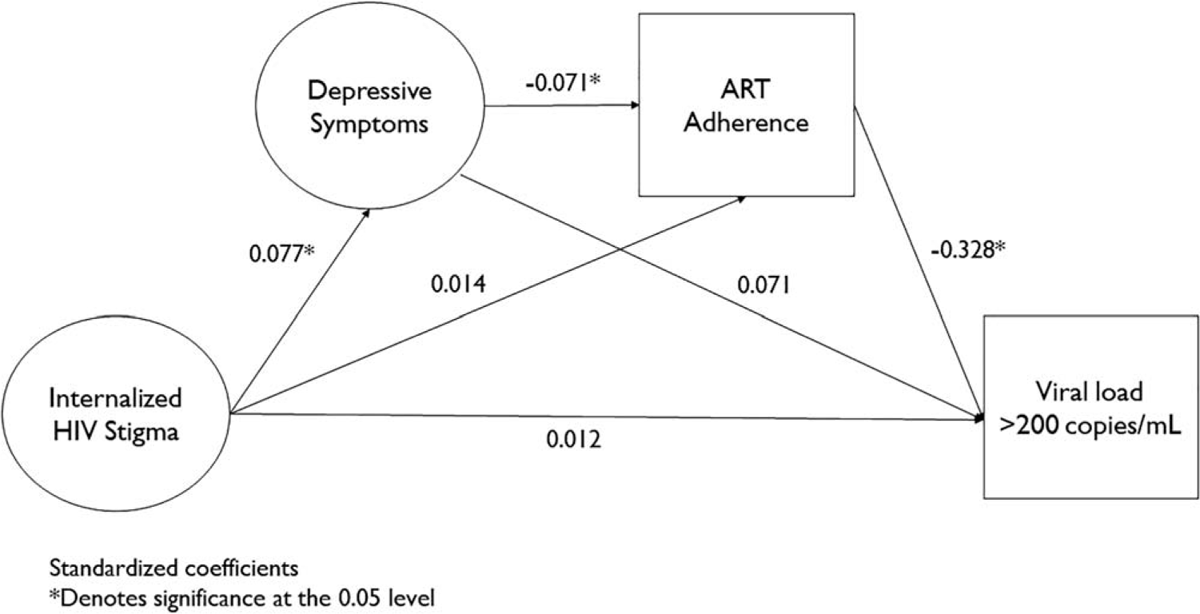


Clum G, Chung SE, Ellen JM, Adolescent Medicine Trials Network for HIVAI. Mediators of HIV-related stigma and risk behavior in HIV infected young women. *AIDS Care.* 2009;21(11):1455-1462. <https://www.ncbi.nlm.nih.gov/pubmed/20024724>

Final path model showing the indirect effect of HIV stigma on risk class membership through social support and depression. Standardized path coefficients were included in path diagram. *p<0.05.


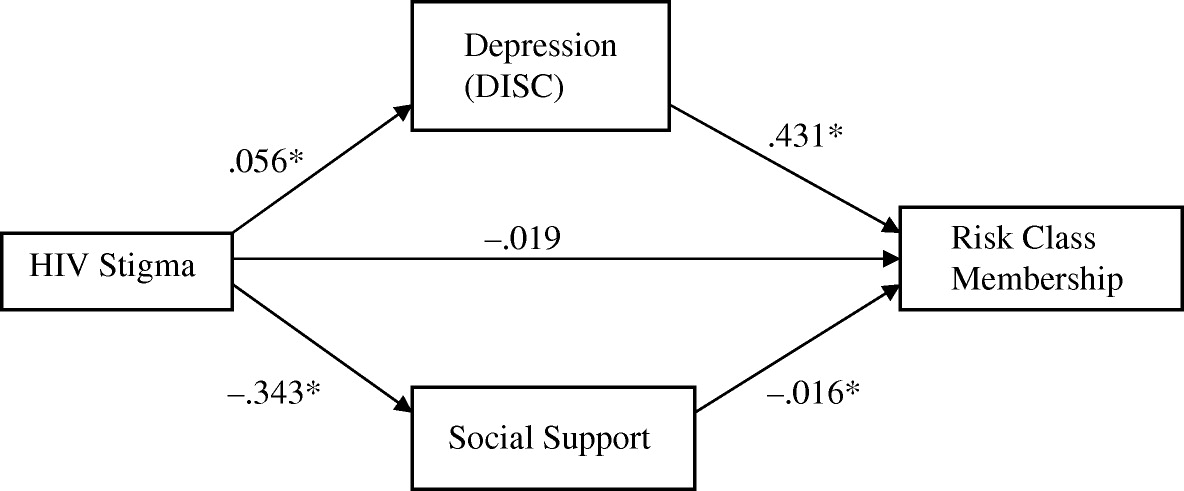


Cluver L, Orkin M, Boyes ME, Sherr L, Makasi D, Nikelo J. Pathways from parental AIDS to child psychological, educational and sexual risk: developing an empirically-based interactive theoretical model. *Soc Sci Med.* 2013;87:185-193. <https://www.ncbi.nlm.nih.gov/pubmed/23631794>

Final estimates (and fit statistics) for the outcomes when modelled independently.


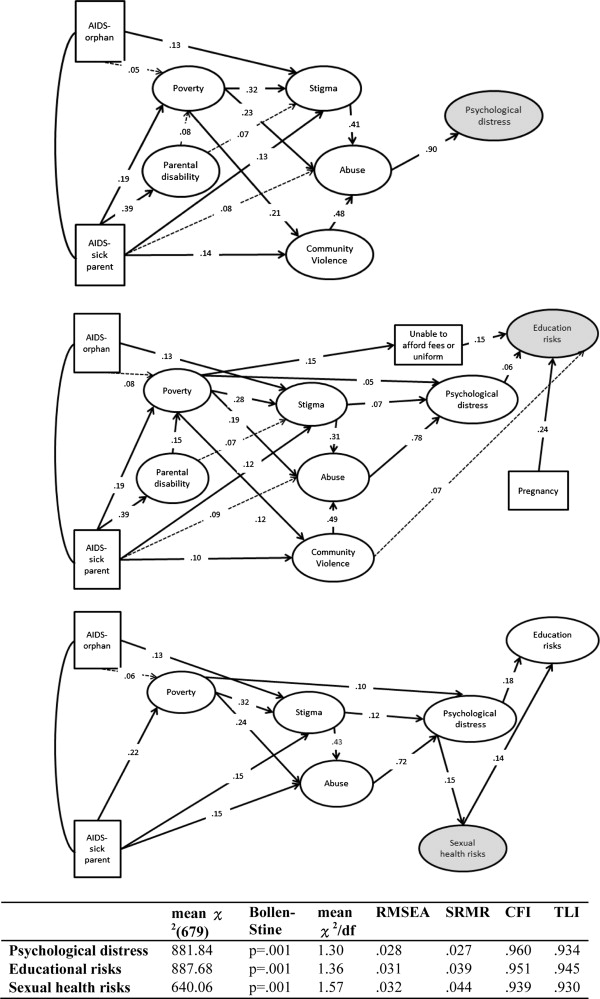


Final estimates for the outcomes when modelled simultaneously.


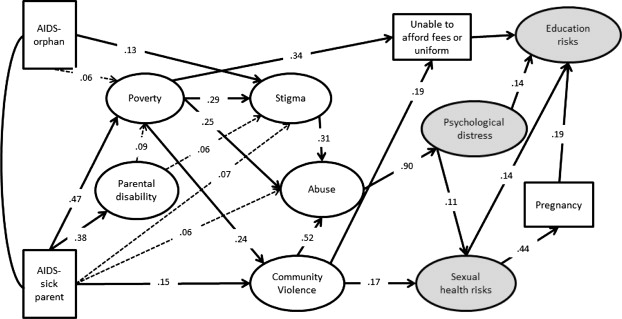


Colbert AM, Kim KH, Sereika SM, Erlen JA. An examination of the relationships among gender, health status, social support, and HIV-related stigma. *JANAC: Journal of the Association of Nurses in AIDS Care.* 2010;21(4):302-313. <https://www.ncbi.nlm.nih.gov/pubmed/20116295>


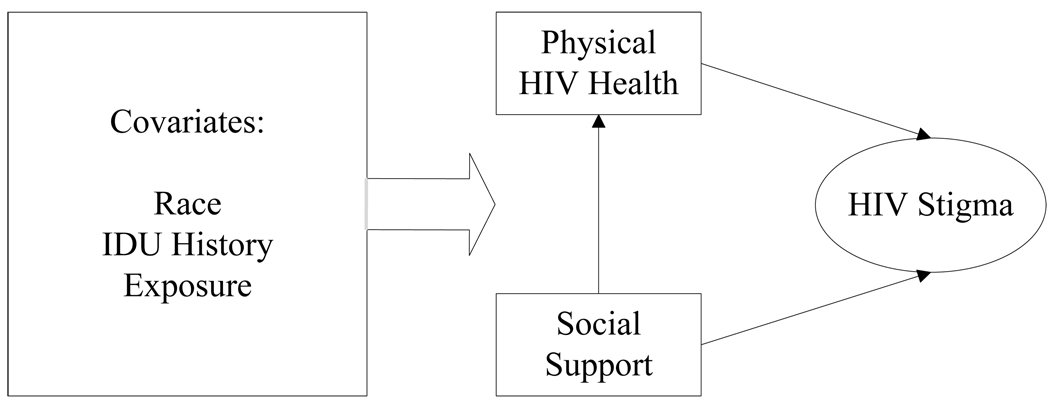


Conceptual model.

Crockett KB, Kalichman SC, Kalichman MO, Cruess DG, Katner HP. Experiences of HIV-related discrimination and consequences for internalised stigma, depression and alcohol use. *Psychol Health.* 2019;34(7):796-810. <https://www.ncbi.nlm.nih.gov/pubmed/30773914>

Serial mediation model depicting pathways between HIV-related discrimination in the past year, stress related to experiences of discrimination, internalised HIV stigma, and depressive symptoms. The following covariates were included: age, years since HIV diagnosis, race, gender, sexual orientation, employment status. Pathways shown are unstandardised beta coefficients. aDenotes when mediators are included in model (direct effect). Statistical significance is denoted as follows: †p < .10; *p < .05; **p < .001.


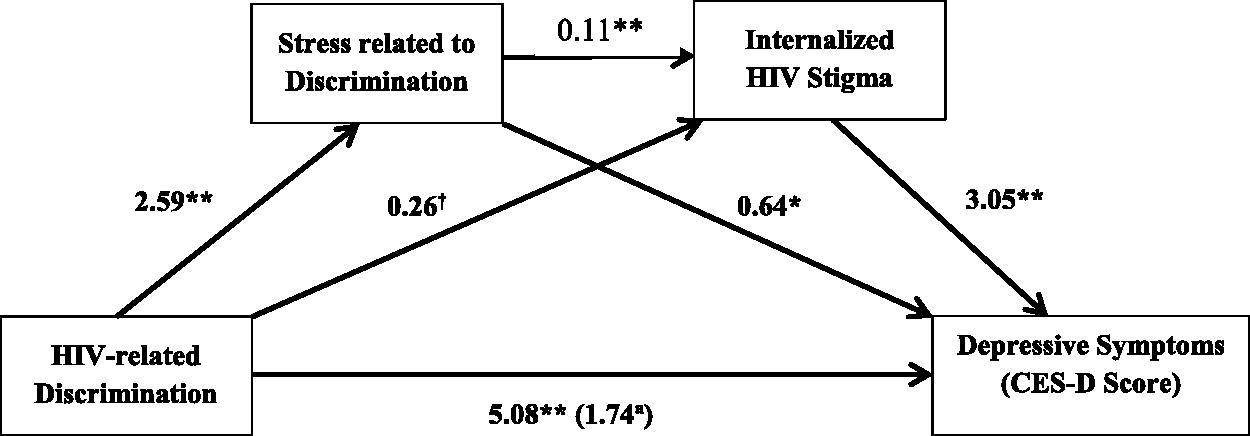


Serial mediation model depicting pathways between HIV-related discrimination in the past year, stress related to experiences of discrimination, internalised HIV stigma, and alcohol use severity. The following covariates were included: age, years since HIV diagnosis, race, gender, sexual orientation, employment status. Pathways shown are unstandardised beta coefficients. aDenotes when mediators are included in model (direct effect). Statistical significance is denoted as follows: †p < .10; *p < .05; **p < .001.


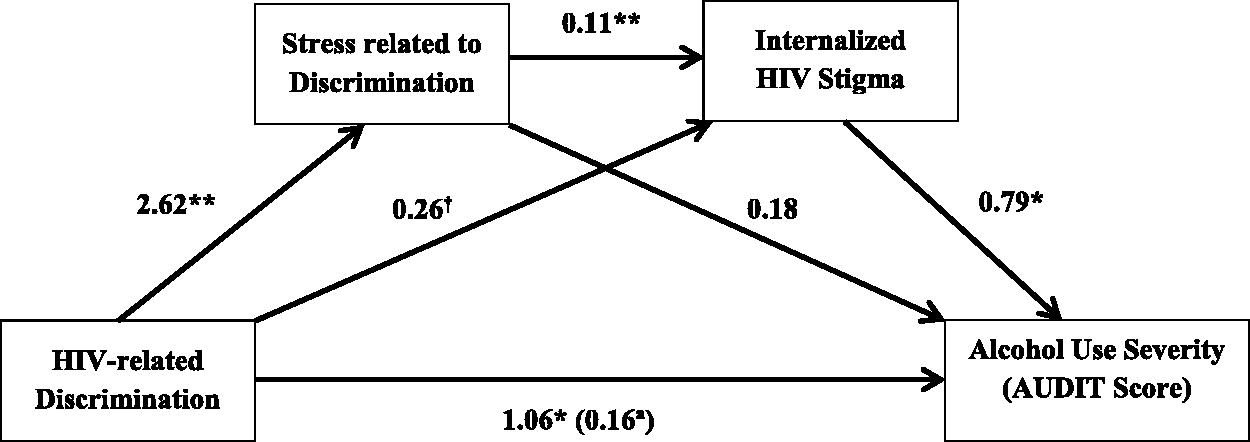


Darlington CK, Hutson SP. Understanding HIV-related stigma among women in the Southern United States: A literature review. *AIDS and Behavior.* 2017;21(1):12-26. <https://www.ncbi.nlm.nih.gov/pubmed/27492025>


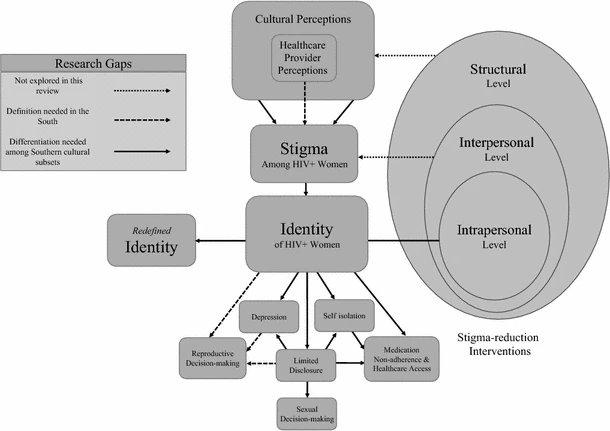


Current state-of-science of HIV-related stigma among HIV + women in the Southern US

Davtyan M, Olshansky EF, Brown B, Lakon C. A Grounded Theory Study of HIV-Related Stigma in U.S.-Based Health Care Settings. *J Assoc Nurses AIDS Care.* 2017;28(6):907-922. <https://www.ncbi.nlm.nih.gov/pubmed/28830704>


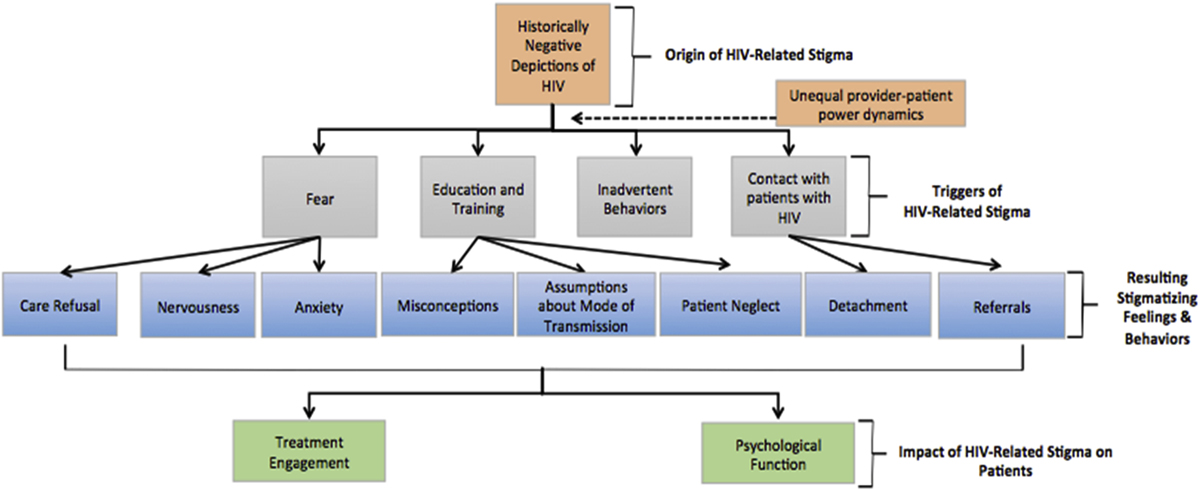


Model of HIV-related stigma in health care settings.

Deacon H. *Understanding HIV/AIDS Stigma. A Theoretical and Methodological Analysis.* Cape Town: HSRC Press;2005. <https://www.hsrcpress.ac.za/books/understanding-hiv-aids-stigma>


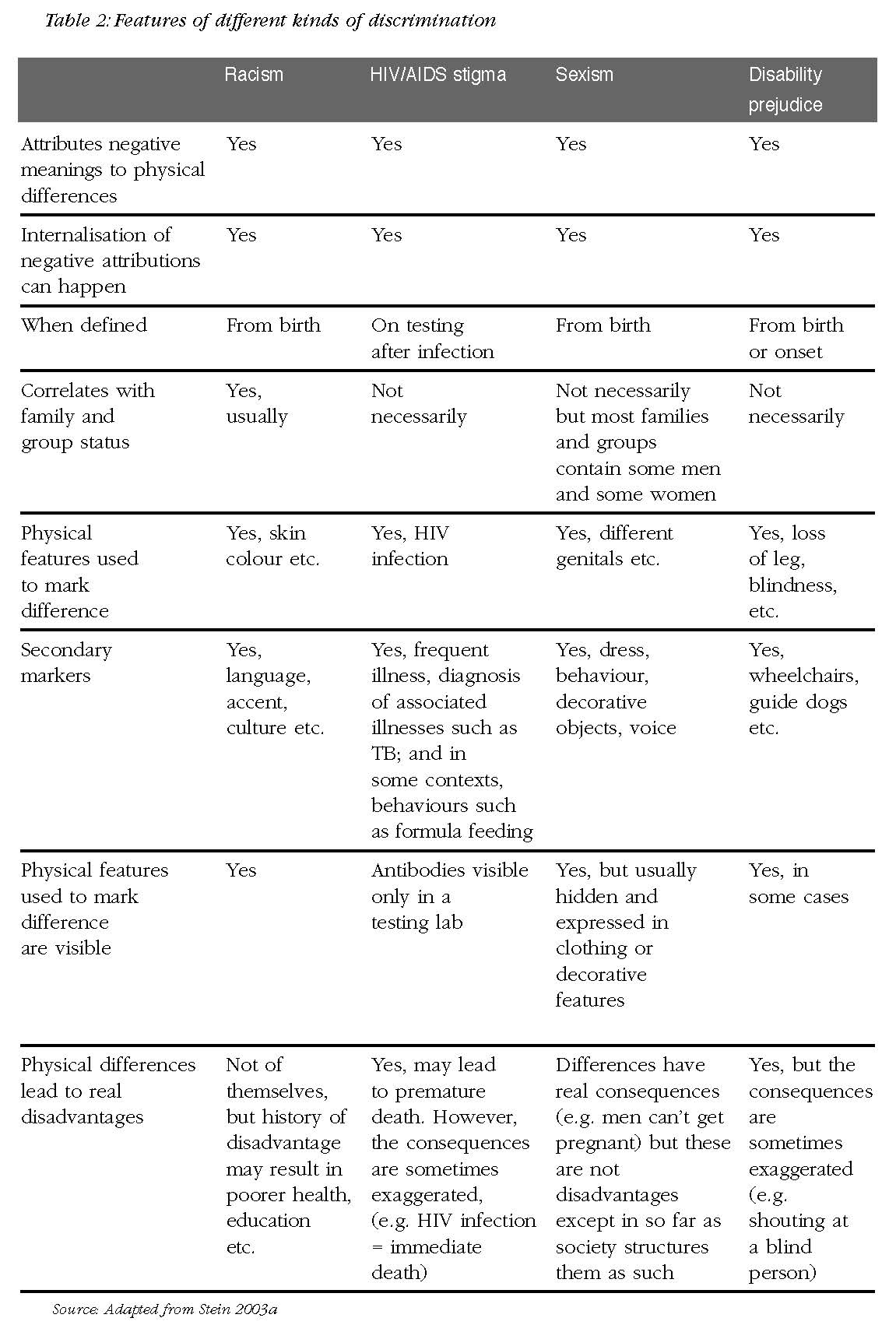


Diiorio C, McCarty F, Depadilla L, et al. Adherence to antiretroviral medication regimens: a test of a psychosocial model. *AIDS Behav.* 2009;13(1):10-22. <https://www.ncbi.nlm.nih.gov/pubmed/17978868>

# Full proposed model of adherence


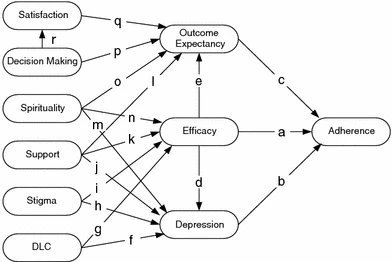


# Model of adherence (model B)


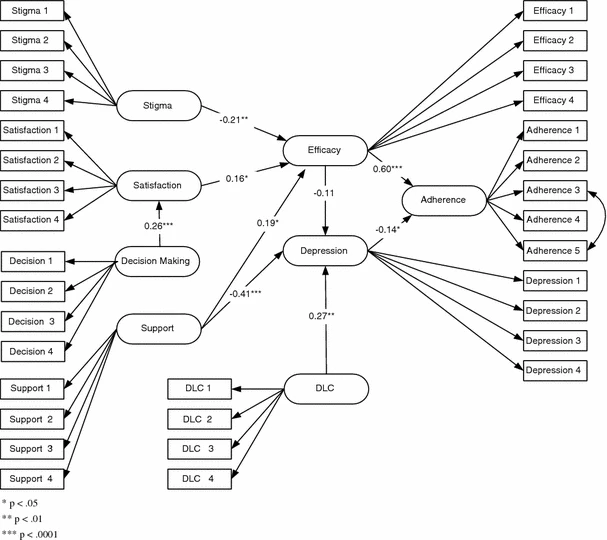


# Reduced model of adherence


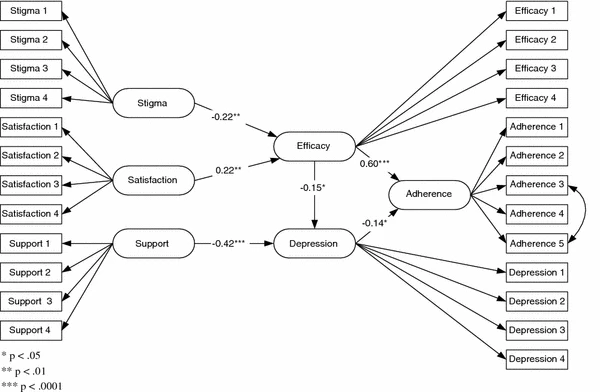


Earnshaw VA, Bogart LM, Dovidio JF, Williams DR. Stigma and racial/ethnic HIV disparities: moving toward resilience. *Am Psychol.* 2013;68(4):225-236. <https://www.ncbi.nlm.nih.gov/pubmed/23688090>

### Stigma and HIV Disparities Model


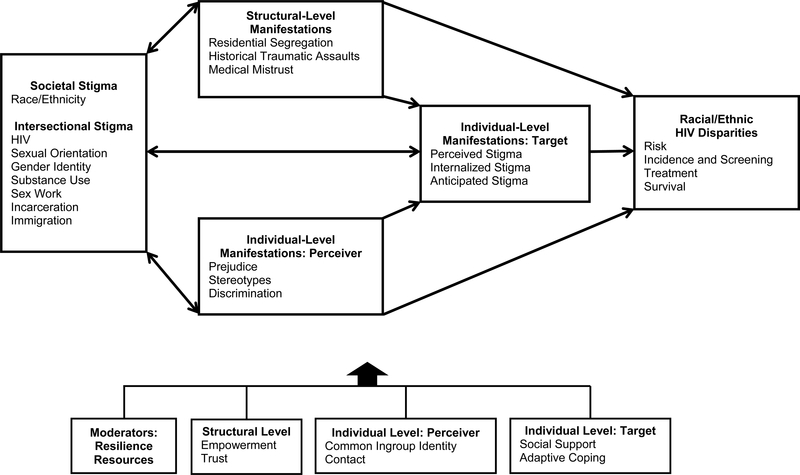


Earnshaw VA, Smith LR, Chaudoir SR, Amico KR, Copenhaver MM. HIV stigma mechanisms and well-being among PLWH: a test of the HIV stigma framework. AIDS Behav. 2013;17(5):1785-1795. <https://www.ncbi.nlm.nih.gov/pubmed/23456594>


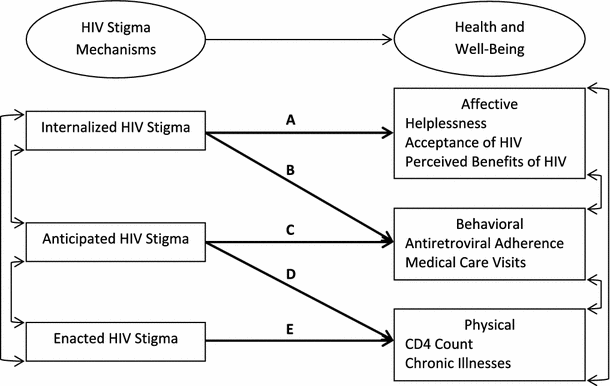


Hypothesized associations between HIV stigma mechanisms and health and well-being among PLWH. Paths in bold are evaluated within current paper

Fekete EM, Williams SL, Skinta MD. Internalised HIV-stigma, loneliness, depressive symptoms and sleep quality in people living with HIV. *Psychol Health.* 2018;33(3):398-415. <https://www.ncbi.nlm.nih.gov/pubmed/28749185>

The indirect association of internalised stigma on global sleep quality through increased loneliness and depressive symptoms.


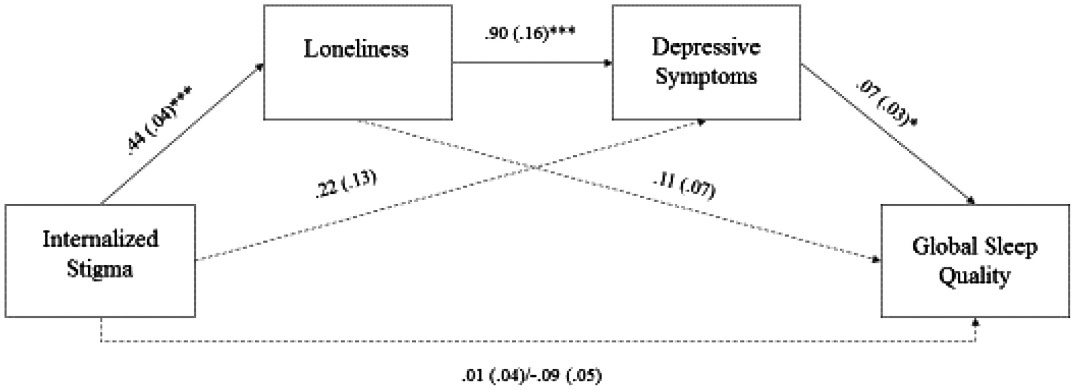


The indirect association of internalised stigma on daytime sleep dysfunction through increased loneliness and depressive symptoms.


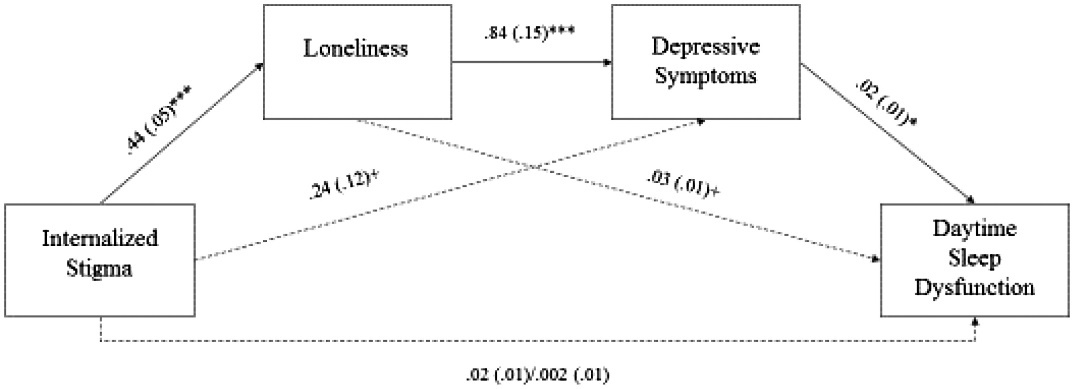


Florom-Smith AL, De Santis JP. Exploring the concept of HIV-related stigma. *Nurs Forum.* 2012;47(3):153-165. <https://www.ncbi.nlm.nih.gov/pubmed/22861652>


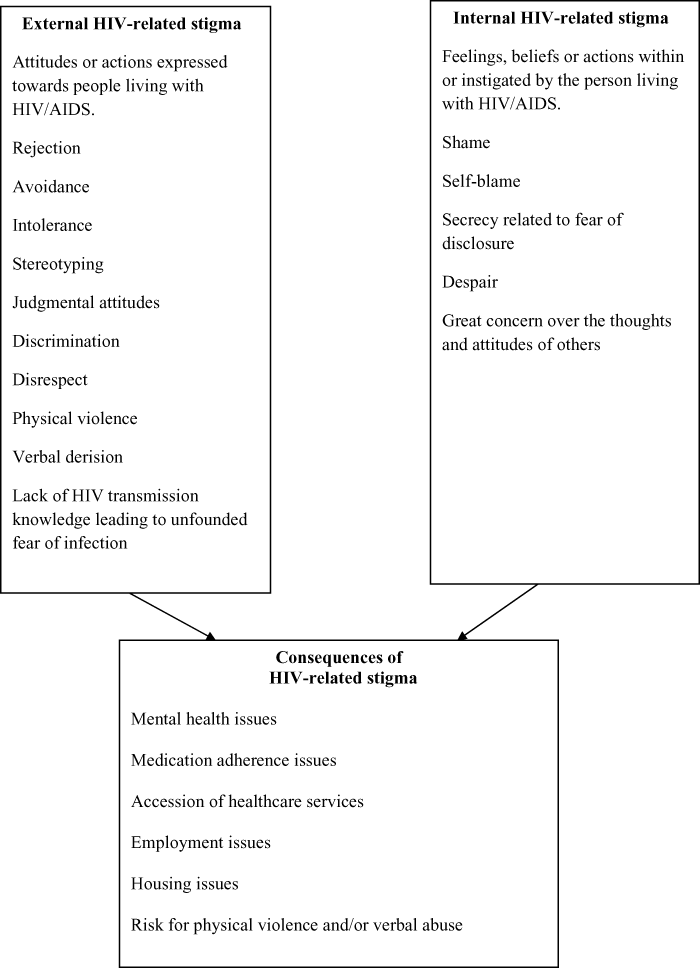


France NF, McDonald SH, Conroy RR, et al. "An unspoken world of unspoken things": a study identifying and exploring core beliefs underlying self-stigma among people living with HIV and AIDS in Ireland. *Swiss Med Wkly.* 2015;145:w14113. <https://www.ncbi.nlm.nih.gov/pubmed/25768695>

Framework for qualitative analysis of interview data.

Three main areas of analysis are shown: Categories of core beliefs (A, red), Functions of self-stigma (B, magenta) and Coping strategies (C, blue). Emergent themes were organised into categories (solid boxes), and subcategories where required (open boxes).


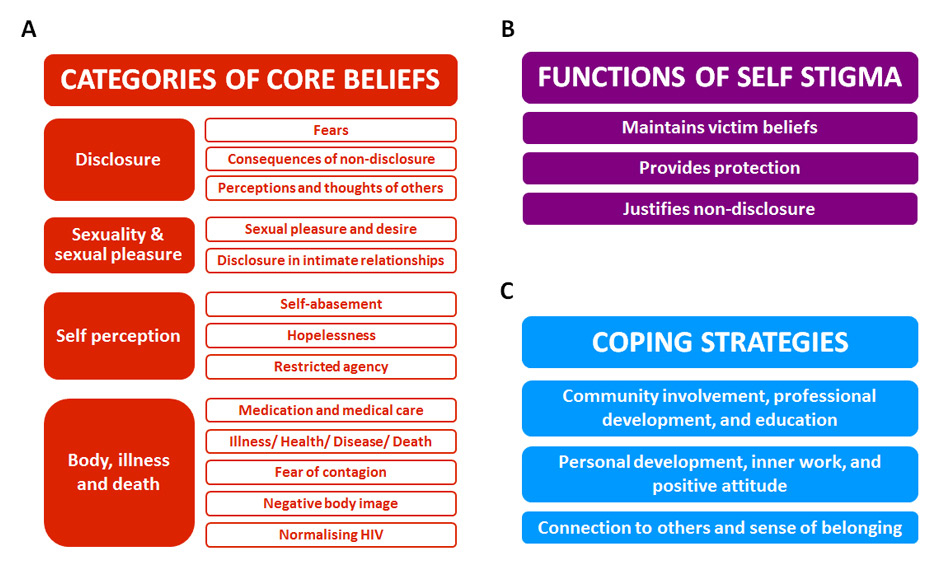


Conceptual framework of self-stigma.

Designed using concepts from Morrison (2006) [52], the framework comprises three interacting groups of factors (factors listed are non-exhaustive) which influence self-stigma: social factors, contextual factors and self factors. The complex interaction between these factors influences the development and perpetuation of self-stigma.


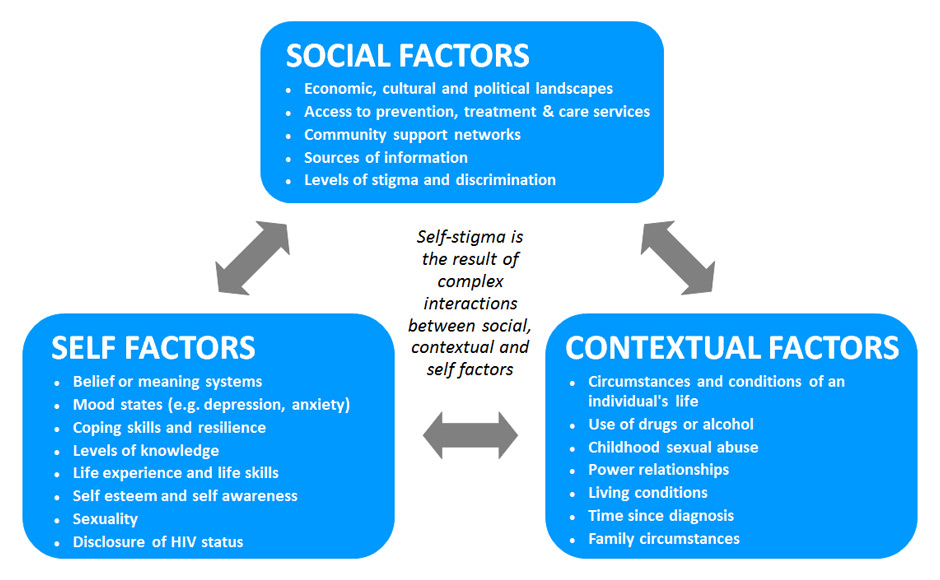


Gilbert L. 'The mercurial piece of the puzzle': Understanding stigma and HIV/AIDS in South Africa. *Sahara j.* 2016;13(1):8-16. <https://www.ncbi.nlm.nih.gov/pubmed/26781444>

The stigma process. (b) Forces impacting on/or shaping stigma.

**
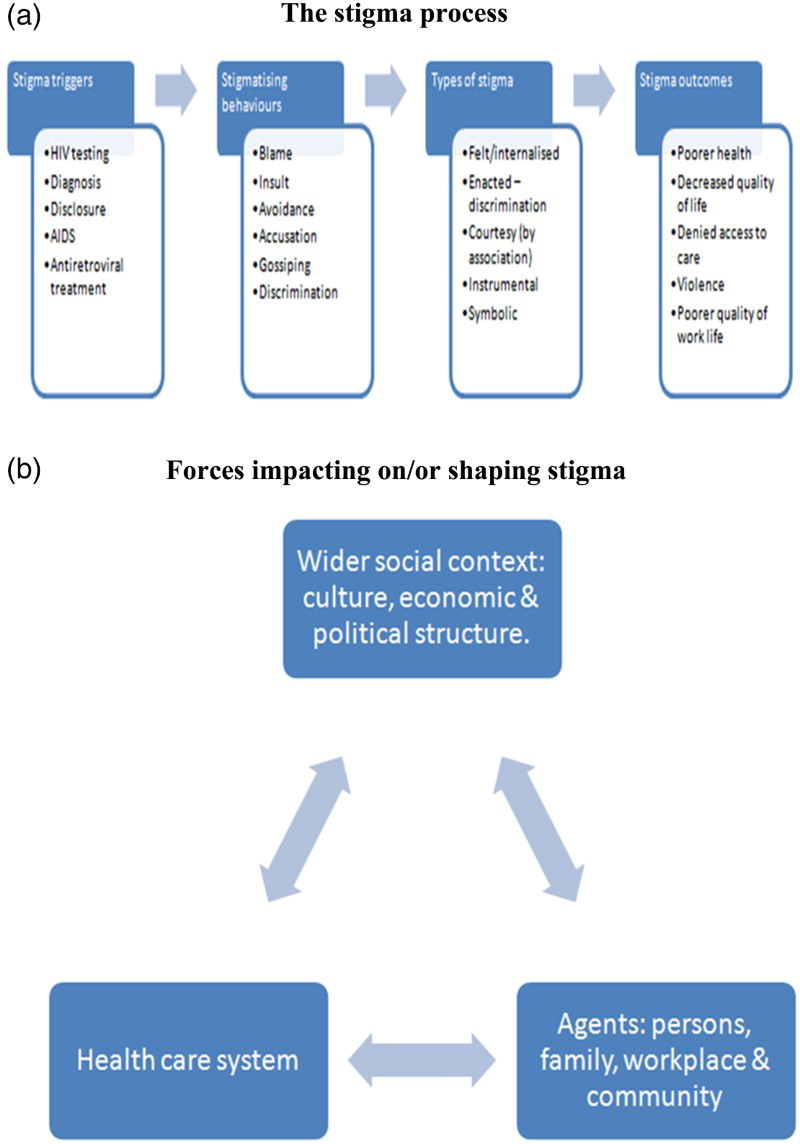
**

Hagopian A, Rao D, Katz A, Sanford S, Barnhart S. Anti-homosexual legislation and HIV-related stigma in African nations: what has been the role of PEPFAR? *Glob Health Action.* 2017;10(1):1306391. <https://www.ncbi.nlm.nih.gov/pubmed/28580879>

Authors’ conceptual framework of the pathways in which anti-homosexuality laws lead to increased incidence of HIV in a population.

**
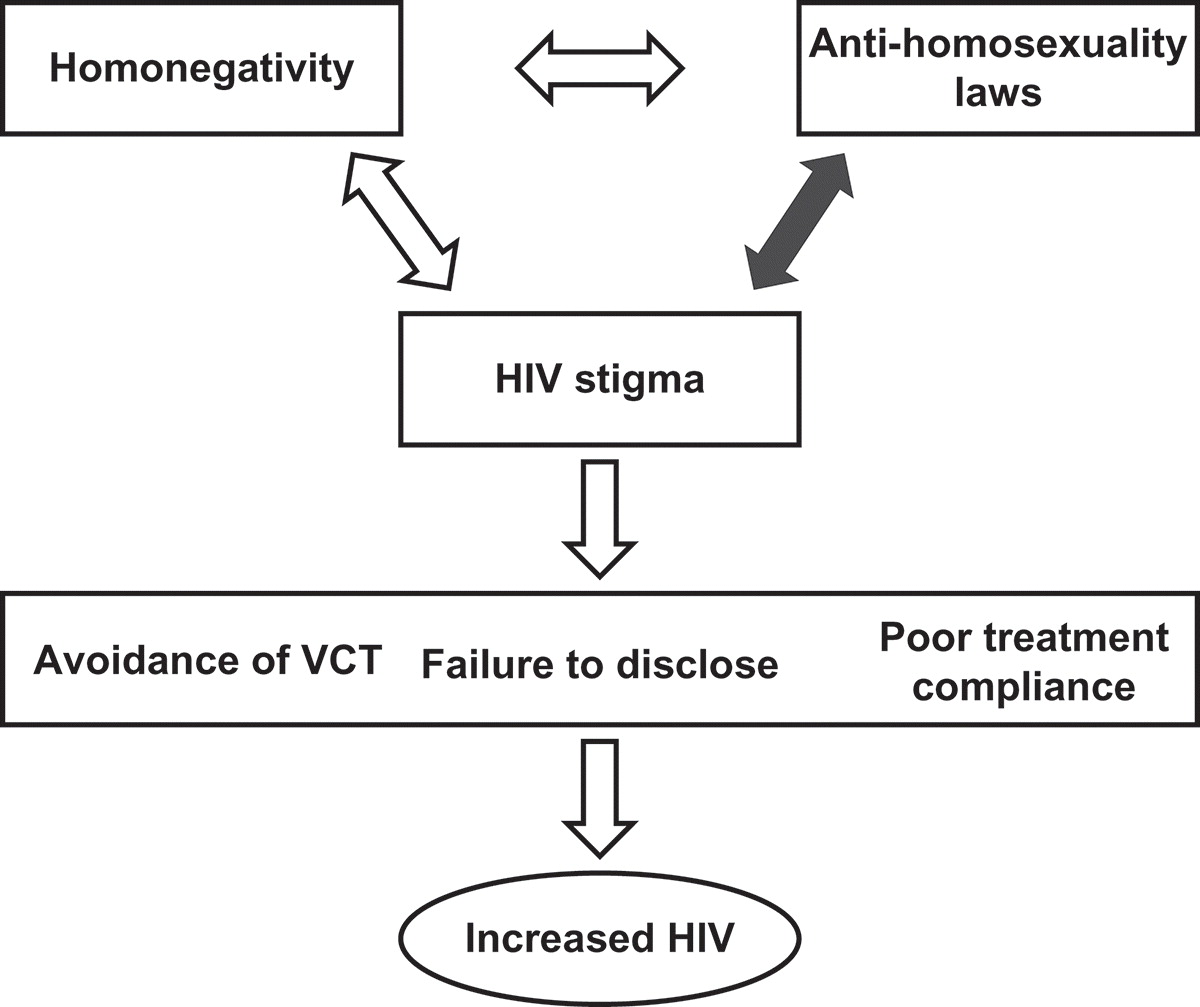
**

Health Policy Plus. Groundbreaking Research and Initiatives to Measure and Reduce Stigma and *Discrimination.* Washington, DC2017. <http://www.healthpolicyplus.com/ns/pubs/7155-7275_StigmaReductionFactsheetversion.pdf>
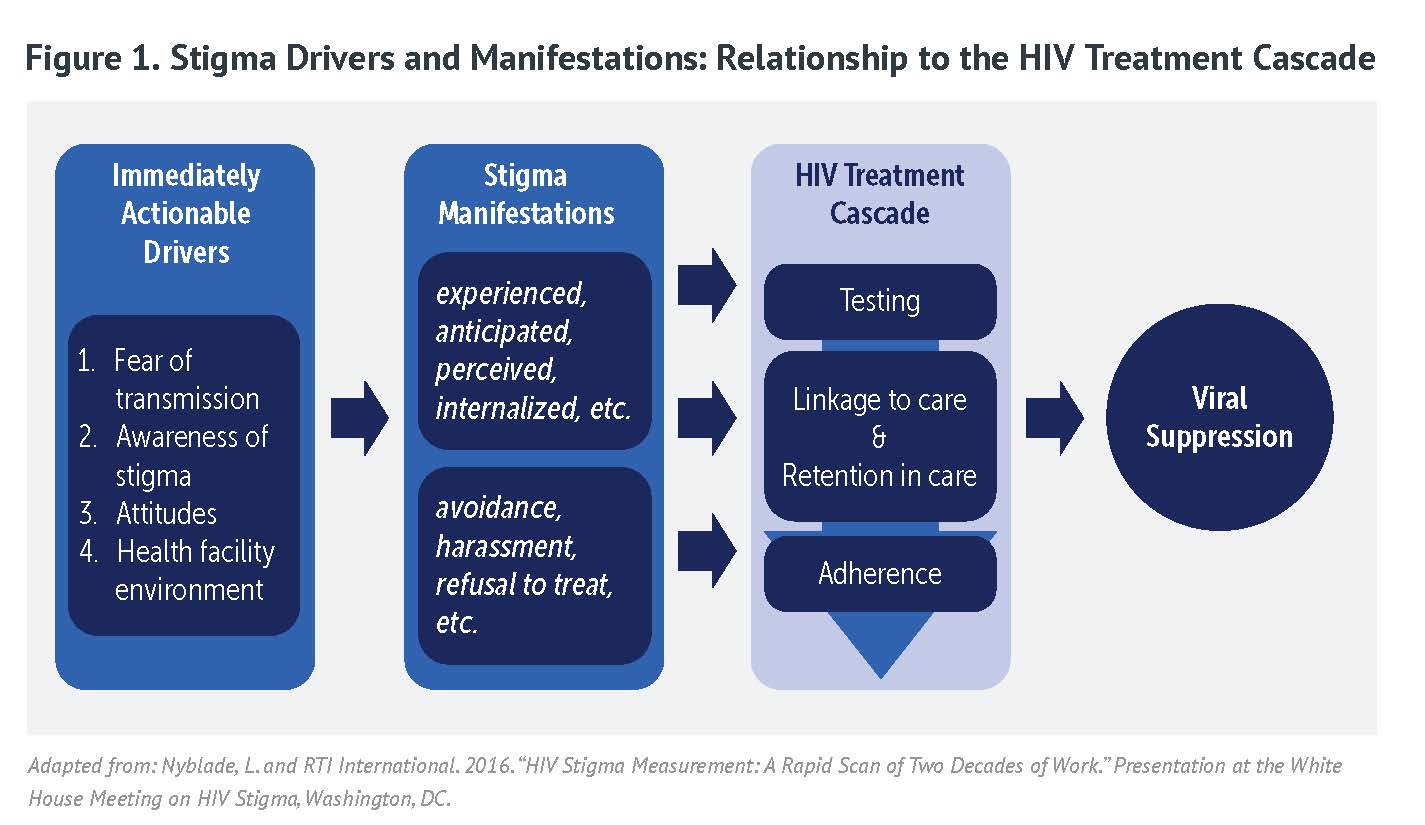


Ikeda DJ, Nyblade L, Srithanaviboonchai K, Agins BD. A quality improvement approach to the reduction of HIV-related stigma and discrimination in healthcare settings. *BMJ Glob Health.* 2019;4(3):e001587. <https://www.ncbi.nlm.nih.gov/pubmed/31297246>


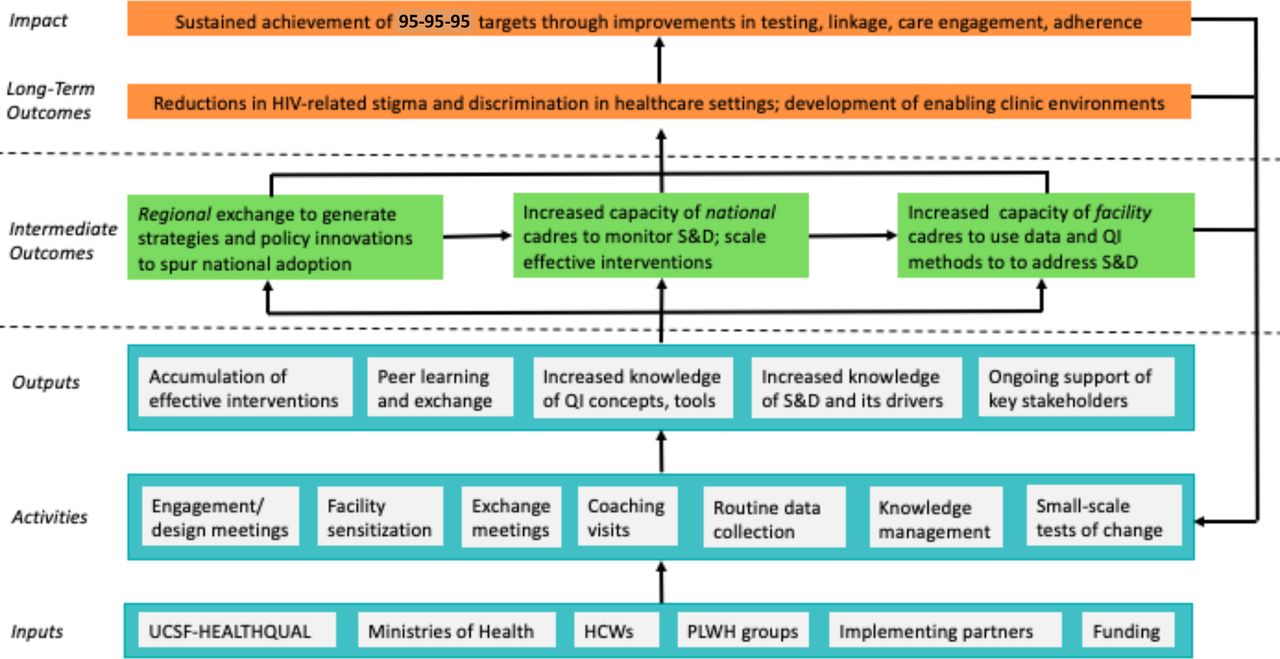


**Theory of action—linking QI capacity building to reduction of S&D. HCWs, healthcare workers; PLWH, people living with HIV; QI, quality improvement; S&D, stigma and discrimination.**

**
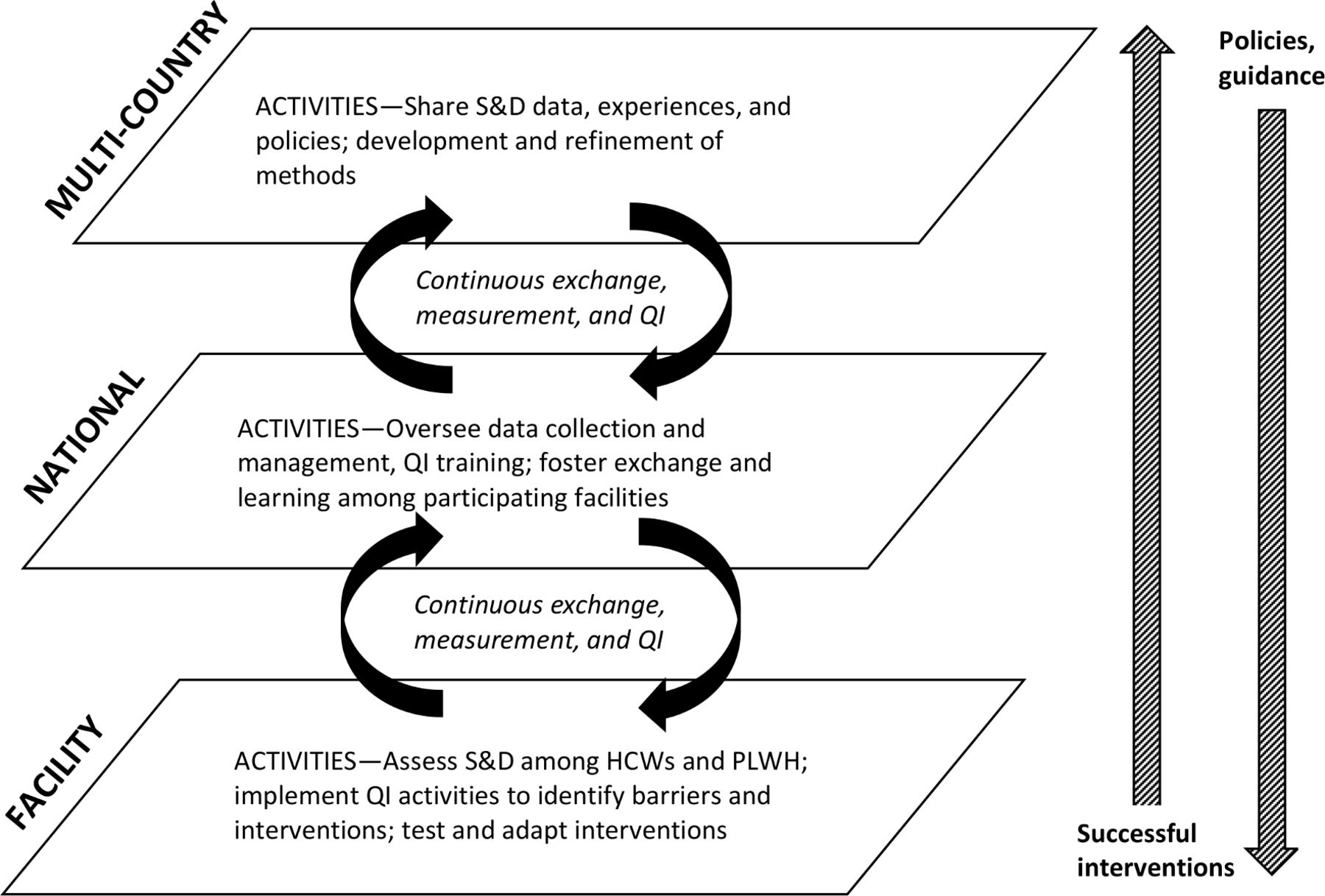
**

**Network design—fostering multiple layers of intervention and exchange. HCWs, healthcare workers; PLWH, people living with HIV; QI, quality improvement; S&D, stigma and discrimination.**

Jain A, Nyblade L. *Scaling Up Policies, Interventions, and Measurement for Stigma-Free HIV Prevention, Care, and Treatment Services.* Washington, DC: Futures Group, Health Policy Project;2012. <https://www.healthpolicyproject.com/pubs/66_WorkingPaperStigmaScaleUpMeasurementJuly.pdf>

Katz IT, Ryu AE, Onuegbu AG, et al. Impact of HIV-related stigma on treatment adherence: systematic review and meta-synthesis. *J Int AIDS Soc.* 2013;16(3 Suppl 2):18640. <https://www.ncbi.nlm.nih.gov/pubmed/24242258>


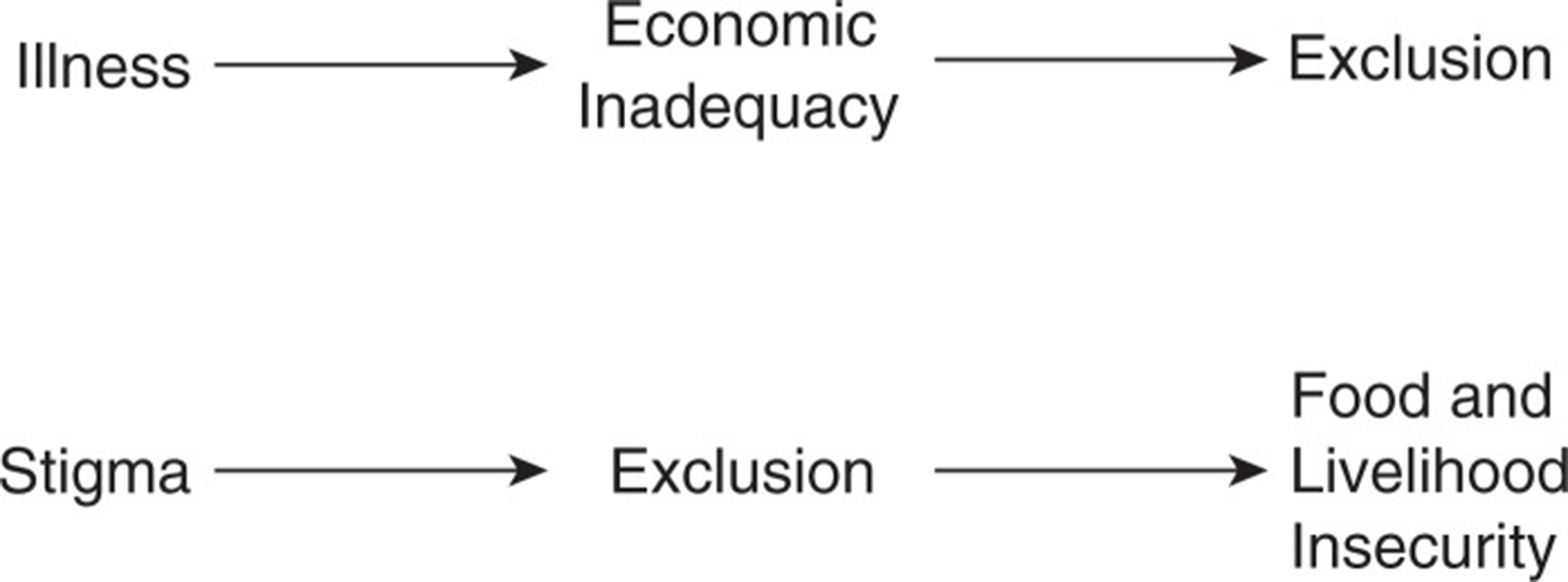


Reciprocal relationships between poverty and stigma. HIV-associated illness reinforces the perceived economic inadequacy of HIV-positive persons, who are excluded from networks of mutual aid. Stigmatized persons are excluded from the community, undermining their social support and worsening economic insecurity.


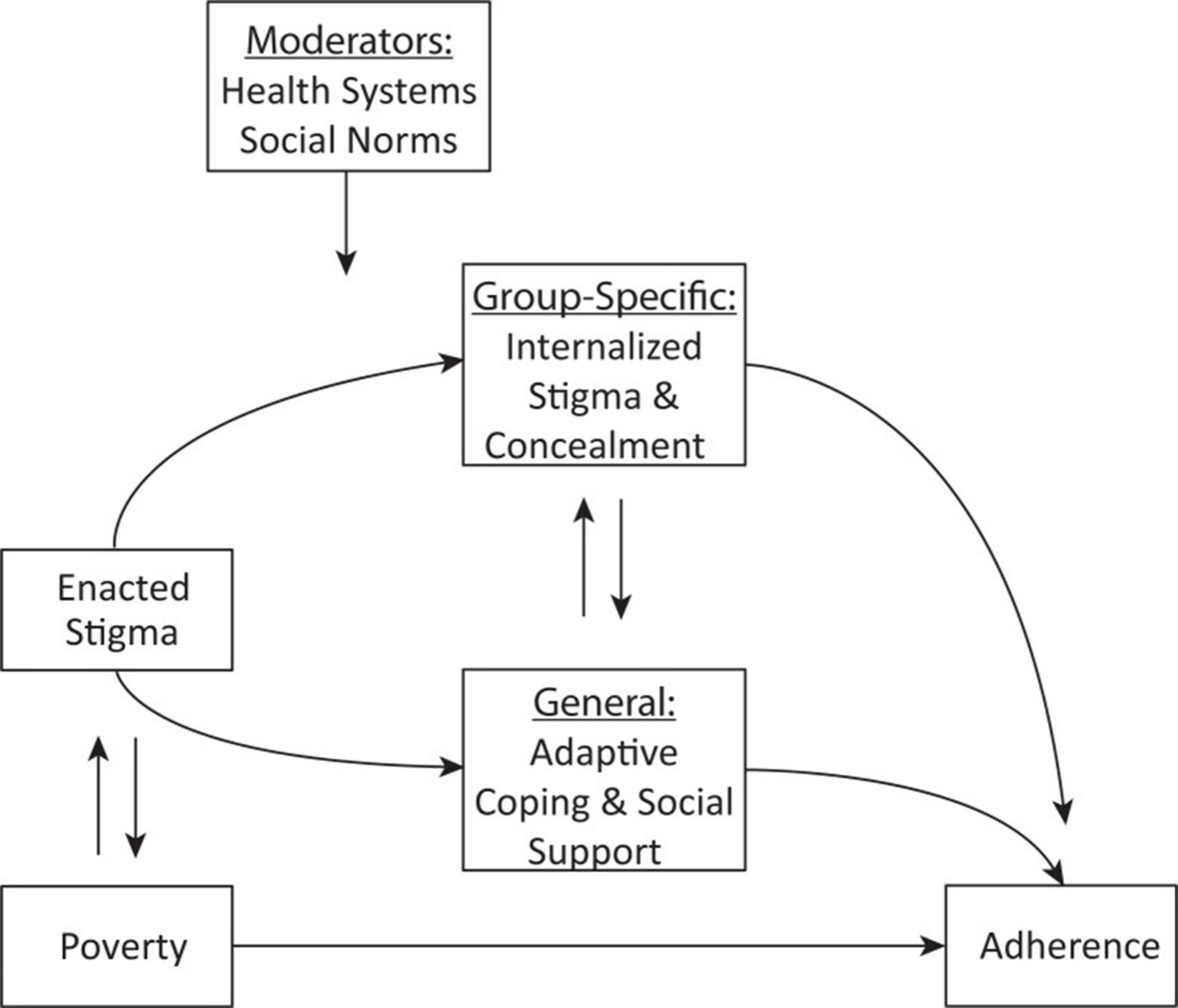


Conceptual model. This figure summarizes the findings of our meta-synthesis of 34 qualitative studies and analysis of 41 quantitative studies. The stigma of HIV was found to compromise ART adherence through general as well as group-specific psychological processes. Adaptive coping and social support were critical determinants of participants’ ability to overcome structural and economic barriers associated with poverty to successfully adhere to ART.

Katz IT, Ryu AE, Onuegbu AG, et al. Impact of HIV-related stigma on treatment adherence: systematic review and meta-synthesis. *J Int AIDS Soc.* 2013b;16(3 Suppl 2):18640. <https://www.ncbi.nlm.nih.gov/pubmed/24242258>

Reciprocal relationships between poverty and stigma. HIV-associated illness reinforces the perceived economic inadequacy of HIV-positive persons, who are excluded from networks of mutual aid. Stigmatized persons are excluded from the community, undermining their social support and worsening economic insecurity.


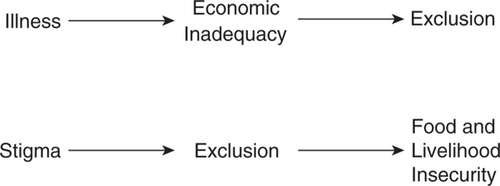


Conceptual model. This figure summarizes the findings of our meta-synthesis of 34 qualitative studies and analysis of 41 quantitative studies. The stigma of HIV was found to compromise ART adherence through general as well as group-specific psychological processes. Adaptive coping and social support were critical determinants of participants’ ability to overcome structural and economic barriers associated with poverty to successfully adhere to ART.


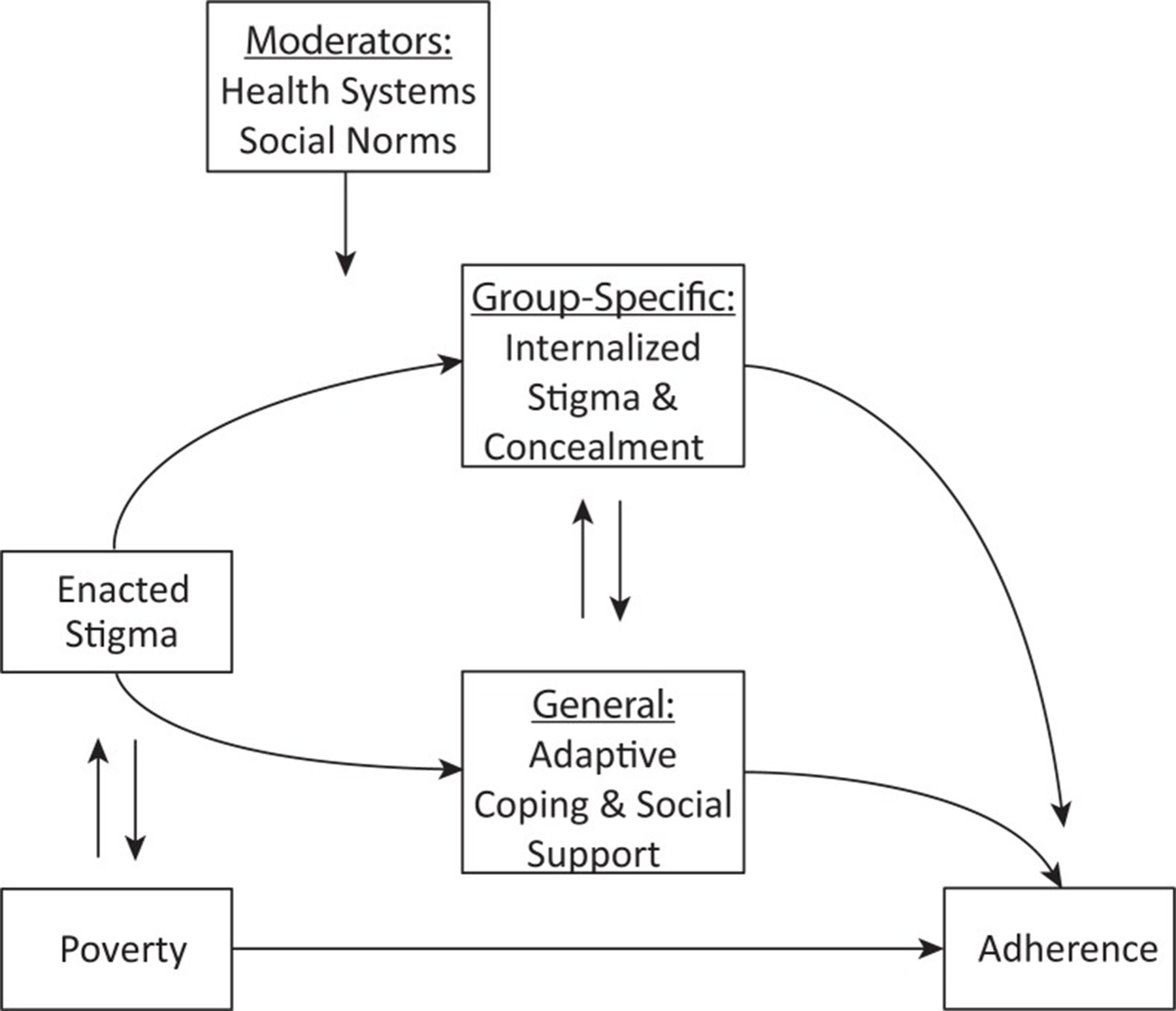


Lipira L, Williams EC, Huh D, et al. HIV-Related Stigma and Viral Suppression Among African-American Women: Exploring the Mediating Roles of Depression and ART Nonadherence. *AIDS Behav.* 2019;23(8):2025-2036. <https://www.ncbi.nlm.nih.gov/pubmed/30343422>


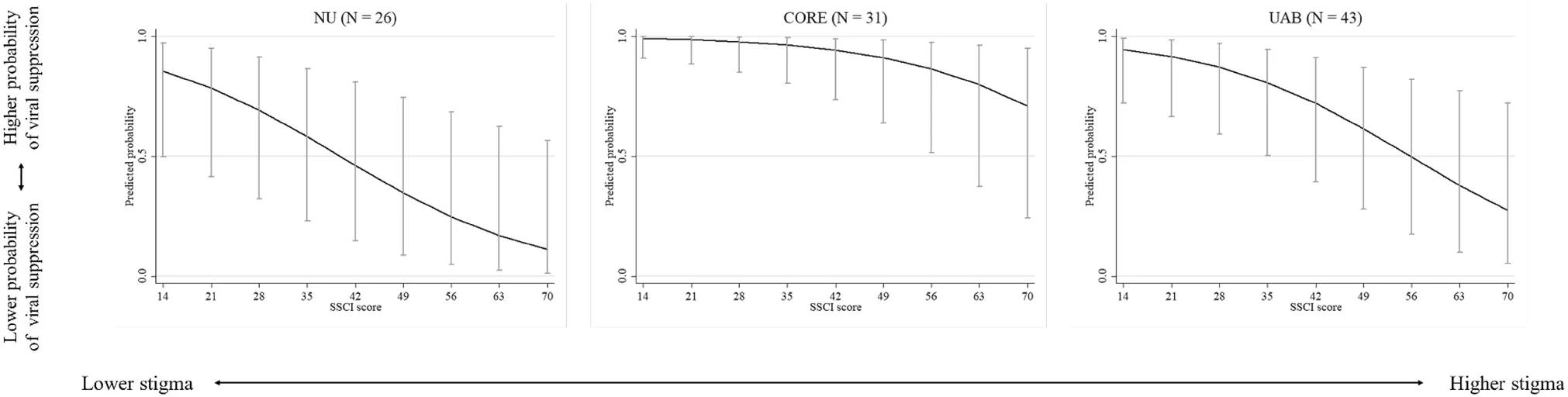


Predicted probability of viral suppression and 95% confidence intervals for different levels of HIV-related stigma (SSCI), by study site, among a sample of African-American women living with HIV enrolled in the Unity Study (N = 100). *SSCI* 14-item Stigma Scale for Chronic Illness, *NU* Northwestern University, *CORE* Ruth M. Rothstein CORE Center, *UAB* University of Alabama, Birmingham


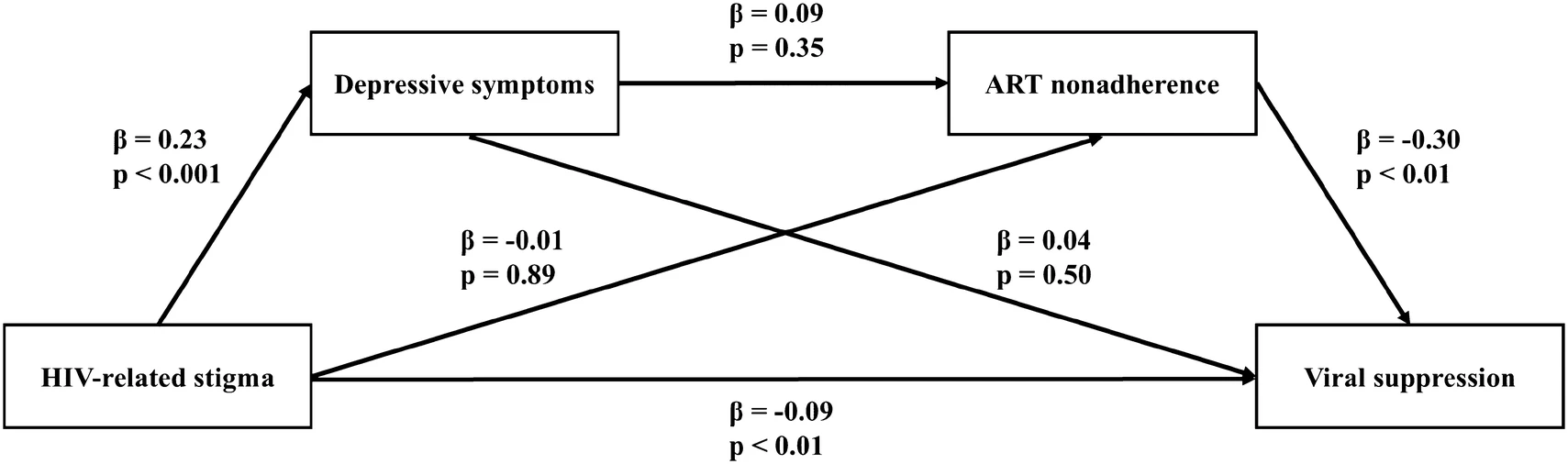


Primary serial mediation model with unstandardized path coefficients estimating associations between HIV-related stigma, depressive symptoms, ART nonadherence and viral suppression among a sample of African-American women living with HIV enrolled in the Unity Study (N = 100). *ART* antiretroviral therapy

Logie CH, Jenkinson JI, Earnshaw V, Tharao W, Loutfy MR. A Structural Equation Model of HIV-Related Stigma, Racial Discrimination, Housing Insecurity and Wellbeing among African and Caribbean Black Women Living with HIV in Ontario, Canada. *PLoS One.* 2016;11(9):e0162826. <https://www.ncbi.nlm.nih.gov/pubmed/27669510>

Tested conceptual model of the relationship between HIV-related stigma, racial discrimination, housing insecurity and wellbeing among African and Caribbean women living with HIV in Ontario.

Fig 1 depicts hypothesized relationships between variables analyzed in the model. Solid lines represent hypothesized direct effects. Ovals represent latent variables, and rectangles represent observed variables


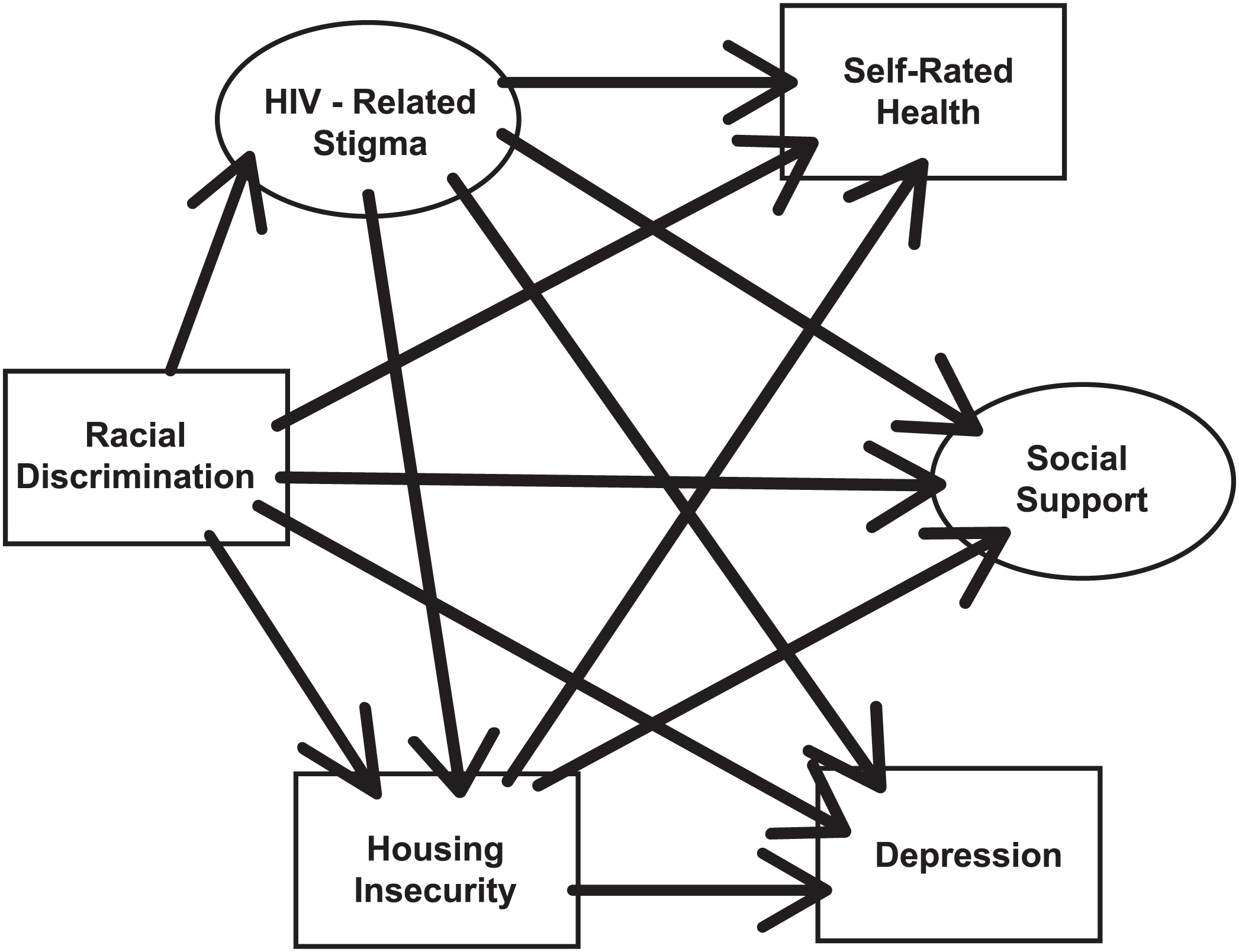


Final model of the relationship between HIV-related stigma, racial discrimination, housing insecurity and wellbeing among African and Caribbean women living with HIV in Ontario (n = 157).

Fig 2 depicts the relationships between the latent variables (depicted as ovals) and observed variables (depicted as rectangles). Solid lines represent statistically significant direct effects and dotted lines represent statistically significant indirect effects. The standardized path coefficients next to each arrow reflect the strength and direction of the effect between variables, and the coefficient is similar to standardized beta weights in regression modeling.


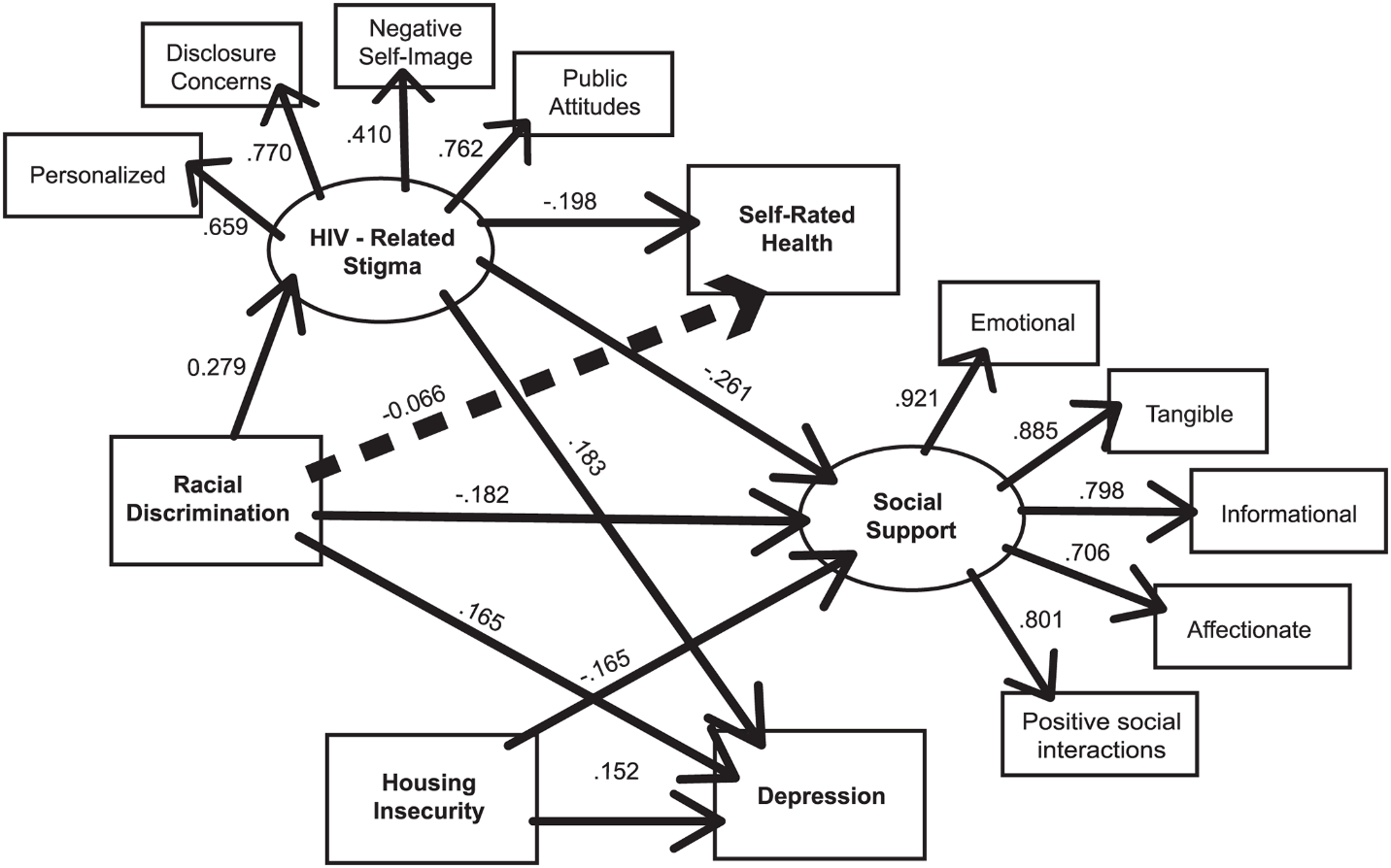


Logie CH, Wang Y, Lacombe-Duncan A, et al. HIV-related stigma, racial discrimination, and gender discrimination: Pathways to physical and mental health-related quality of life among a national cohort of women living with HIV. *Prev Med.* 2018;107:36-44. <https://www.ncbi.nlm.nih.gov/pubmed/29277410>

Tested model for HIV-related stigma, racial discrimination, and gender discrimination on mental and physical health-related quality of life for women living with [HIV](https://www-sciencedirect-com.libproxy1.usc.edu/topics/medicine-and-dentistry/human-immunodeficiency-virus) in Canada (2013–2015).


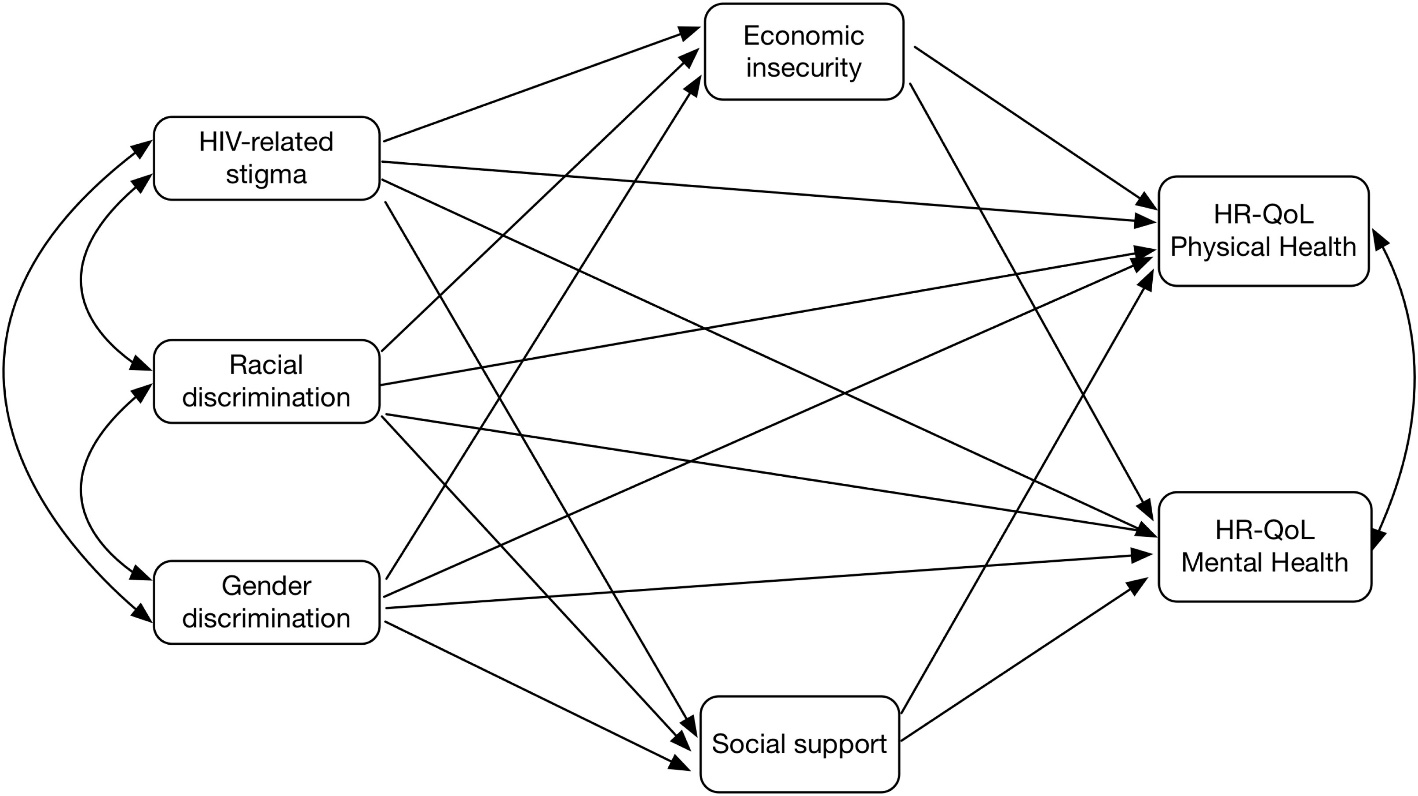


Final model for HIV-related stigma, racial discrimination, and gender discrimination on health-related quality of life (mental and physical health) among women living with [HIV](https://www-sciencedirect-com.libproxy1.usc.edu/topics/medicine-and-dentistry/human-immunodeficiency-virus) in Canada (2013–2015).

Note. Standard coefficients are reported with the standard errors in parentheses.

Statistical significance is noted with the following notations: *p < 0.05, **p < 0.01, ***p < 0.001.

Covariates include: age, ethnicity, immigration status, relationship status, and education level.


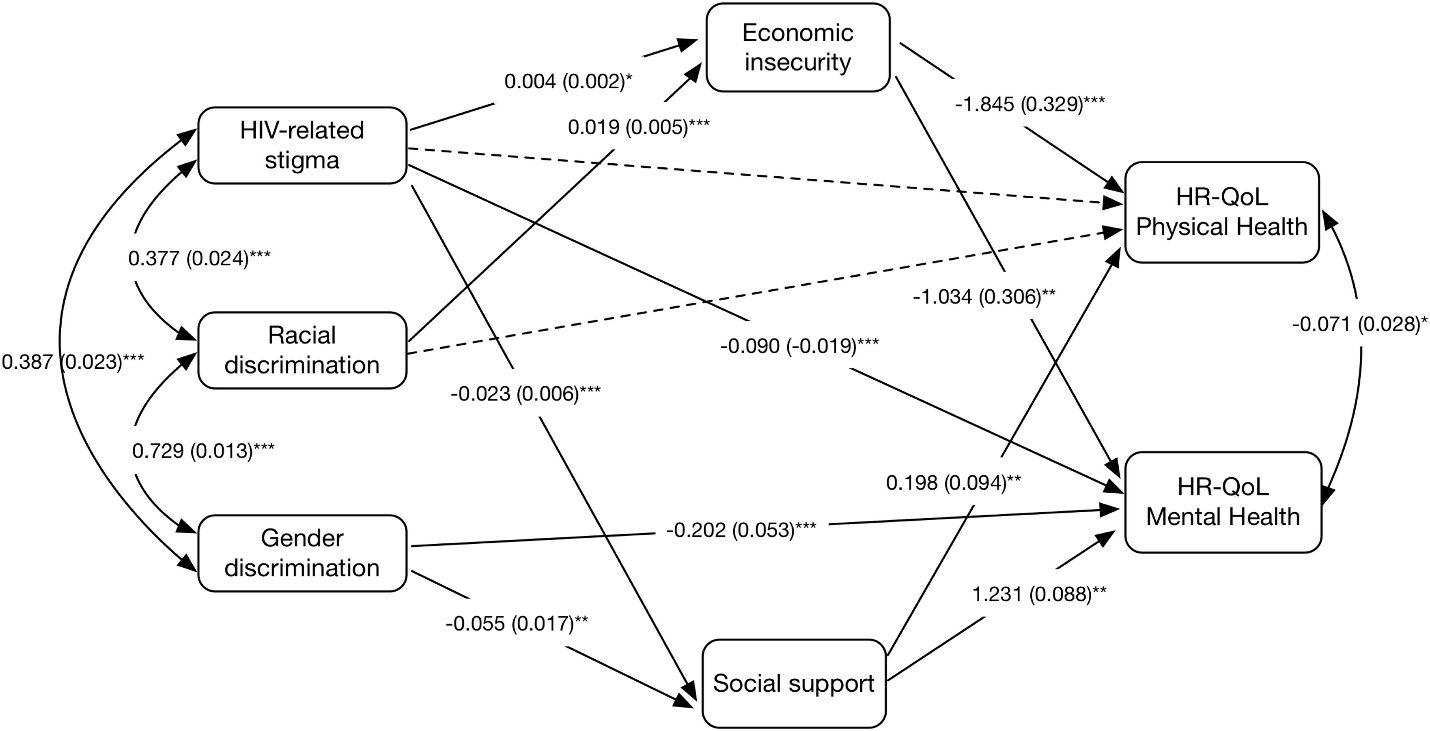


Logie CH, Lacombe-Duncan A, Wang Y, et al. Pathways From HIV-Related Stigma to Antiretroviral Therapy Measures in the HIV Care Cascade for Women Living With HIV in Canada. *J Acquir Immune Defic Syndr.* 2018;77(2):144-153. <https://www.ncbi.nlm.nih.gov/pubmed/29135650>

Conceptualized pathways from HIV-related stigma to ART initiation, current ART use, and >90% ART adherence.


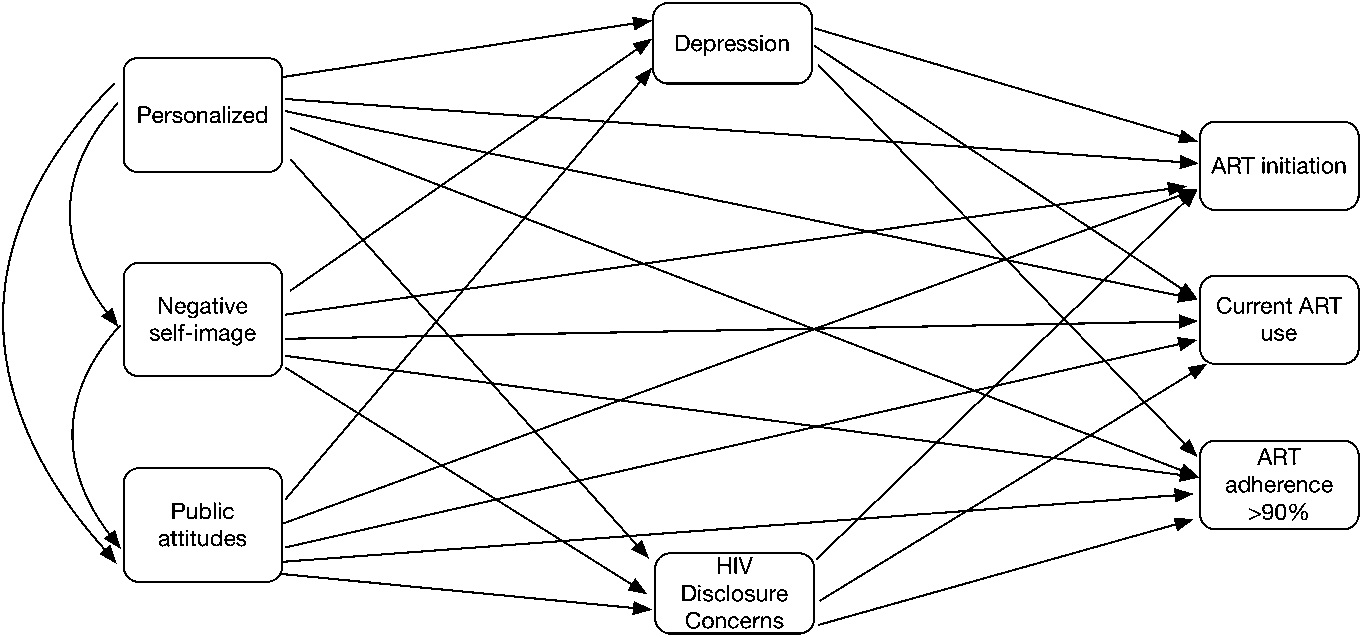


Final path analysis results for HIV-related stigma on ART initiation, current ART use, and ART adherence >90%. Standard coefficients are reported with the standard errors in parentheses. Statistical significance is noted with the following notations; *P < 0.05, **P < 0.01, ***P < 0.001. Covariates include age, ethnicity, immigration status, relationship status, education level, and years with HIV.


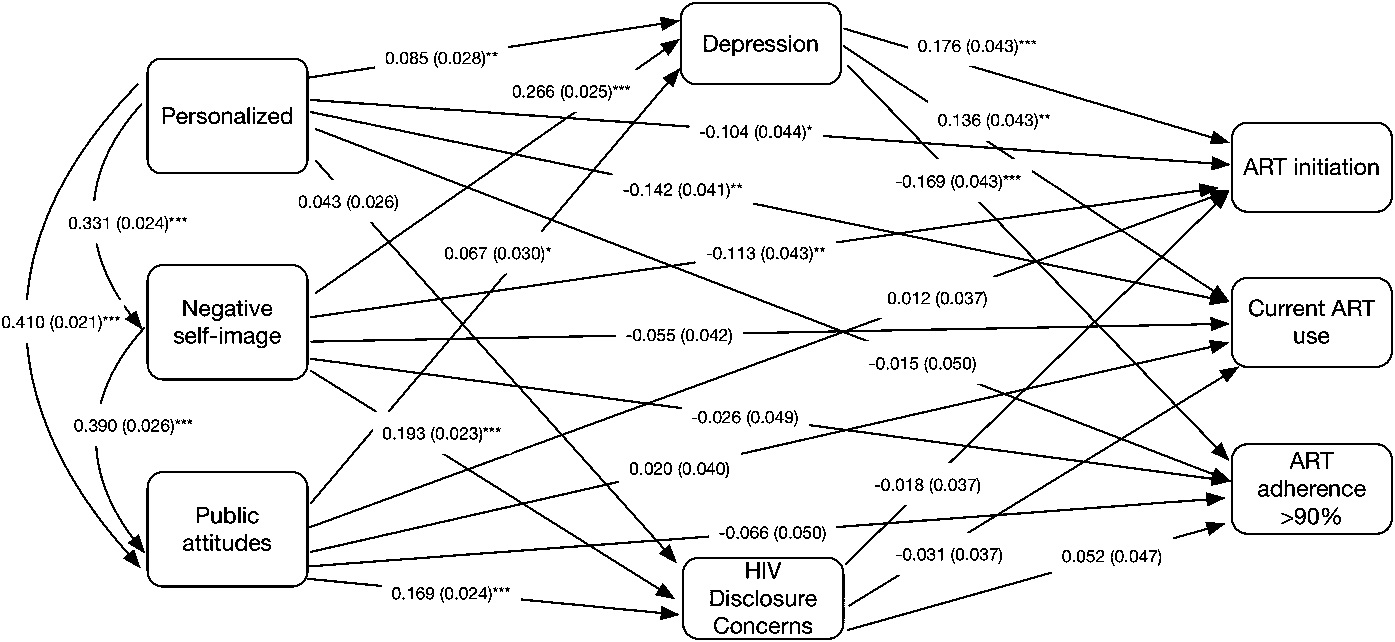


Logie CH, Williams CC, Wang Y, et al. Adapting stigma mechanism frameworks to explore complex pathways between intersectional stigma and HIV-related health outcomes among women living with HIV in Canada. *Soc Sci Med.* 2019;232:129-138. <https://www.ncbi.nlm.nih.gov/pubmed/31079013>


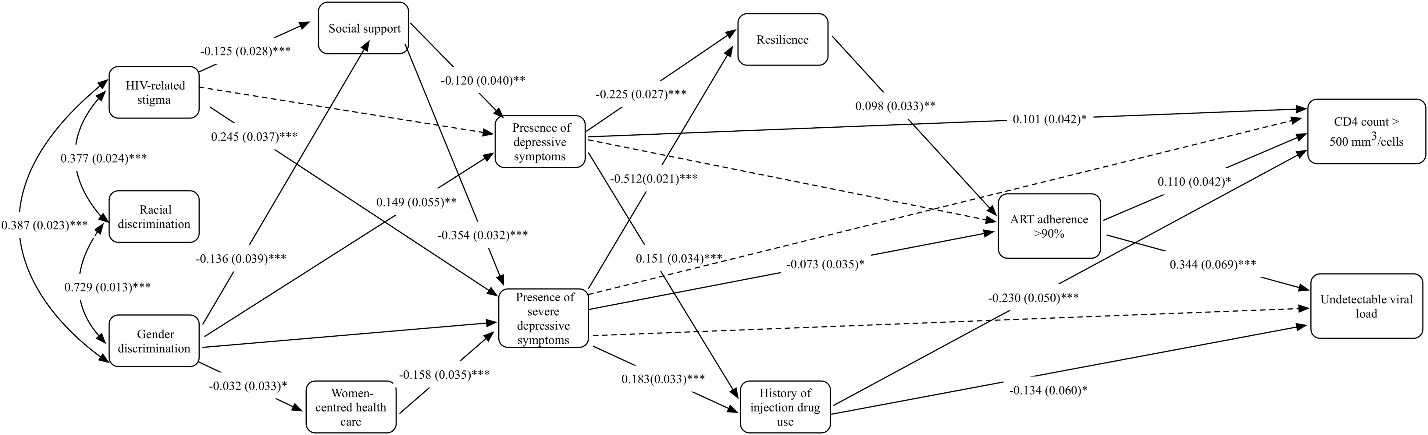


Final path analysis for intersectional stigma types, depression and HIV-related clinical outcomes for an integrated model of intersectional stigma mechanisms among women living with HIV in Canada (N = 1367).

Note. Standard coefficients are reported with standard errors in parentheses. Covariates include age, ethnicity, immigration status, relationship status, education level and years living with HIV. *p < 0.05, **p < 0.01, ***p < 0.001.


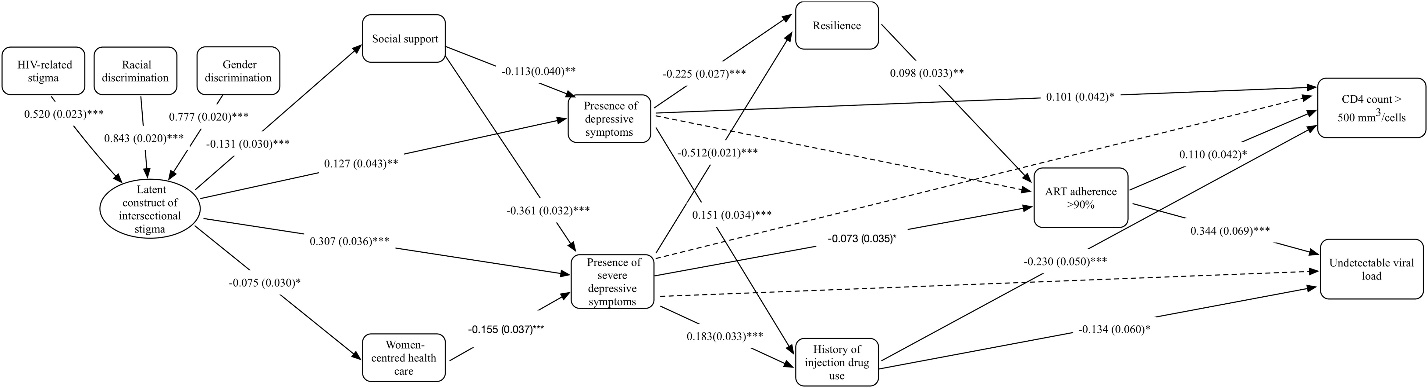


Final structural equation model for a latent construct of intersectional stigma, depression and HIV-related clinical outcomes for an integrated model of intersectional stigma mechanisms among women living with HIV in Canada (N = 1367).

Note. Standard coefficients are reported with standard errors in parentheses. Covariates include age, ethnicity, immigration status, relationship status, education level and years living with HIV. *p < 0.05, **p < 0.01, ***p < 0.001.

Logie CH, Ahmed U, Tharao W, Loutfy MR. A Structural Equation Model of Factors Contributing to Quality of Life Among African and Caribbean Women Living with HIV in Ontario, Canada. *AIDS Res Hum Retroviruses.* 2017;33(3):290-297. <https://www.ncbi.nlm.nih.gov/pubmed/27750027>

Tested conceptual model of factors contributing to quality of life among African and Caribbean women living with HIV.


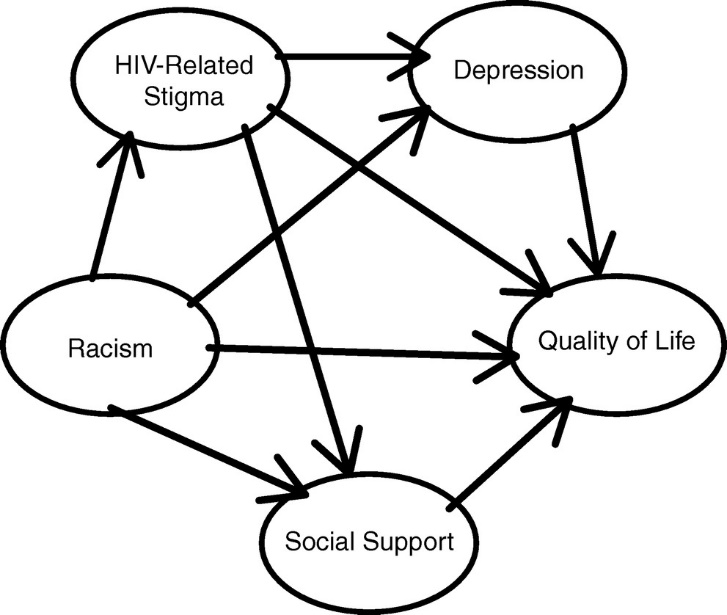


Final model of factors contributing to quality of life among African and Caribbean women living with HIV in Ontario (n = 166).


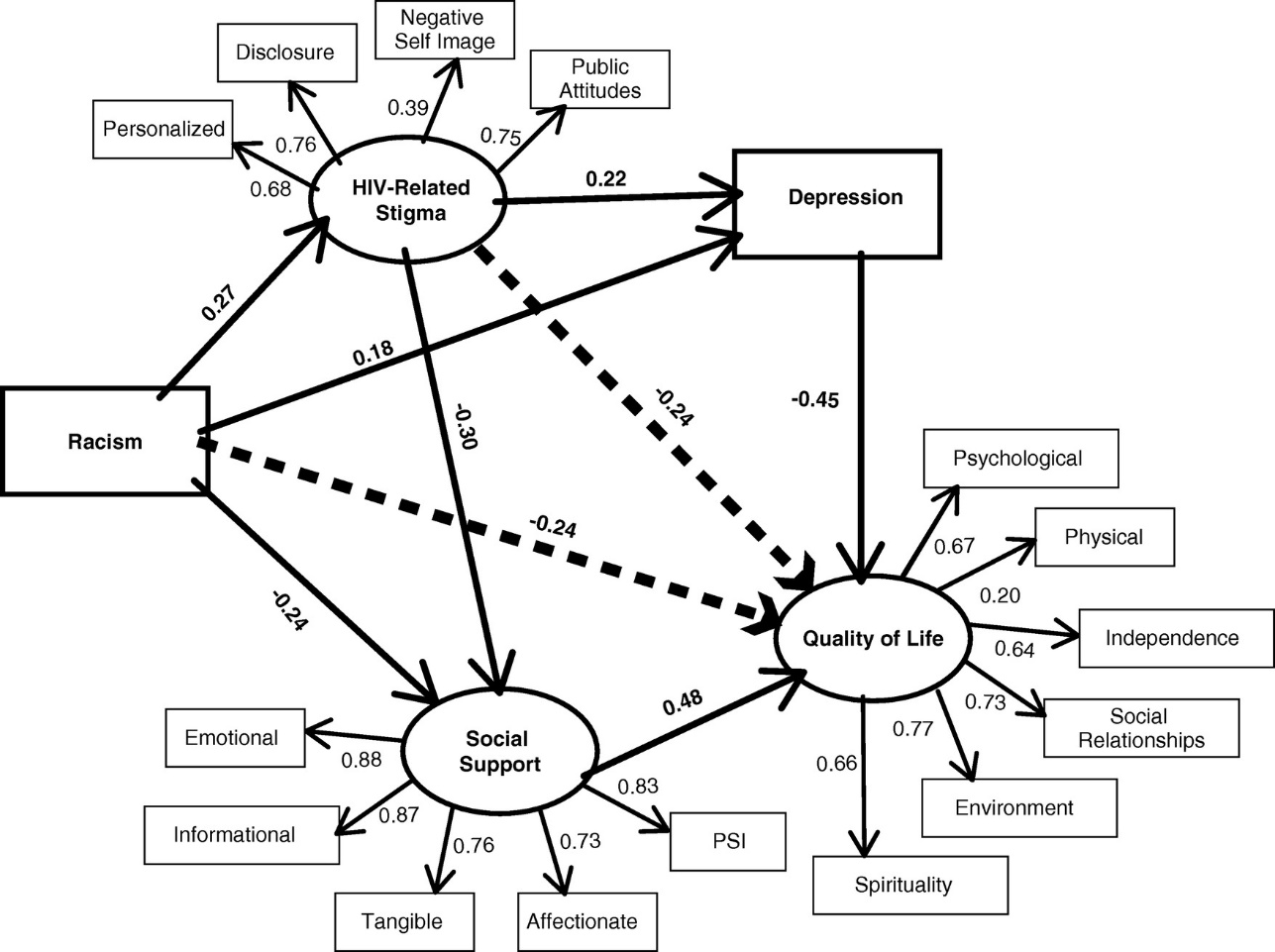


Mahajan AP, Sayles JN, Patel VA, et al. Stigma in the HIV/AIDS epidemic: A review of the literature and recommendations for the way forward. *AIDS.* 2008;22(Suppl2):S67-S79. <https://www.ncbi.nlm.nih.gov/pubmed/18641472>

| In [Fig. 2](https://ovidsp-dc2-ovid-com.libproxy1.usc.edu/ovid-b/ovidweb.cgi?QS2=434f4e1a73d37e8ce3703c84bb996c0dae34b80a178e4349b07a85a42861bd0de1f6544bc0b33d6ab9560e6ae818dfa5eadaf7572a8fce9c8236b79bfc015acf38f7cdd89643719e15710b3a8d9c6efd01cc34b653f3065872f93fbb0bf7736987a9b41a2b9be22bc683d928379266dc7177b17e58ced2235a3e505b2f53bbaa7c014e4a14f1877da3893fa7f0cd35108a3fa435c2cafdae6e1ca0d449d44d58f6308448a4dd4a2278dd4aee2215ed698b877aef4634397a6c8ee77b4cd48378455028ae4c14d11bbd071326fa002f174497906a2819c3589564804f12c8e3d9ee30339718fea30ace9404f4899fbc8082b117d97026eca0cd15876cac159a0a08c1ba452ac2f7d74788a2537c7cae03992f4c33f6a803c8e4721e265701bb316b27ace8a646025ba73ed023ffe9ce4403c5641ce0e4c359385314358b7ab908#FF2), we offer a schematic that illustrates a starting point for a conceptual framework for H/A stigma, derived from this review of the literature. |  |
| --- | --- |


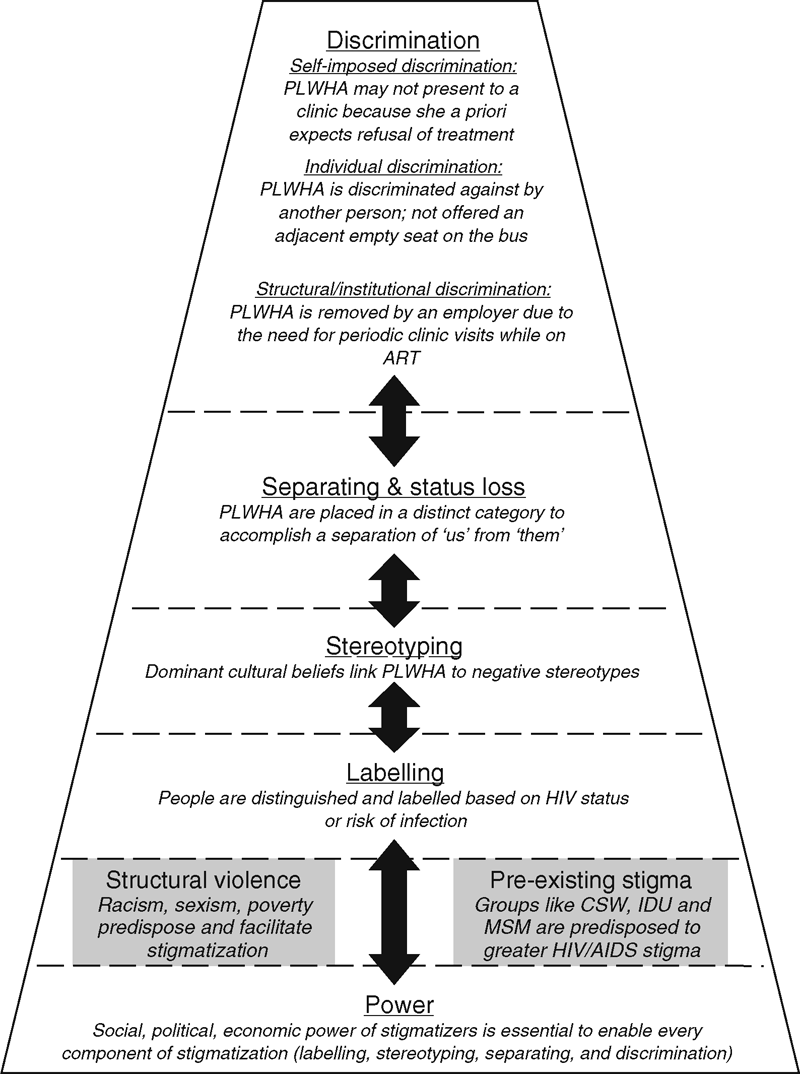


Meyerson B, Barnes P, Emetu R, Bailey M, Ohmit A, Gillespie A. Institutional and structural barriers to HIV testing: Elements for a theoretical framework. *AIDS Patient Care and STDs.* 2014;28(1):22-27. <https://www.ncbi.nlm.nih.gov/pubmed/24313812>


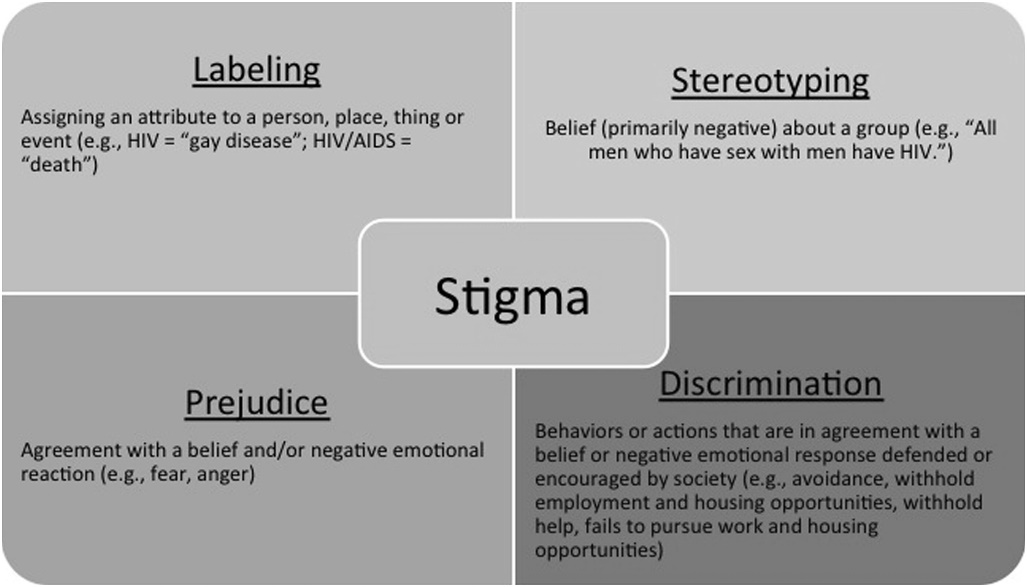


 Conceptual framework elements of stigma.

Miller CT, Solomon SE, Varni SE, Hodge JJ, Knapp FA, Bunn JY. A transactional approach to relationships over time between perceived HIV stigma and the psychological and physical well-being of people with HIV. *Soc Sci Med.* 2016;162:97-105. <https://www.ncbi.nlm.nih.gov/pubmed/27348608>


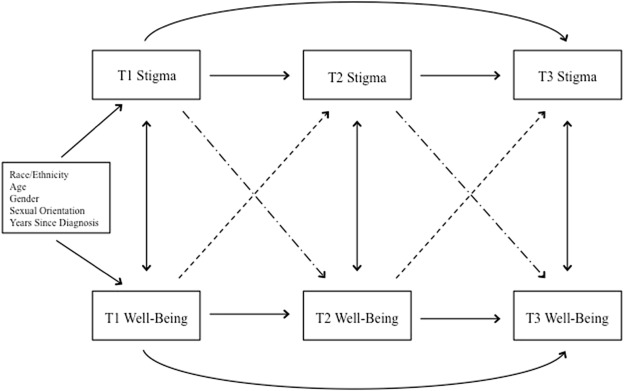


Path analysis model used to examine the relationships of stigma and well-being across time.

Mo PK, Lau JT, Yu X, Gu J. A model of associative stigma on depression and anxiety among children of HIV-infected parents in China. *AIDS Behav.* 2015;19(1):50-59. <https://www.ncbi.nlm.nih.gov/pubmed/24879629>


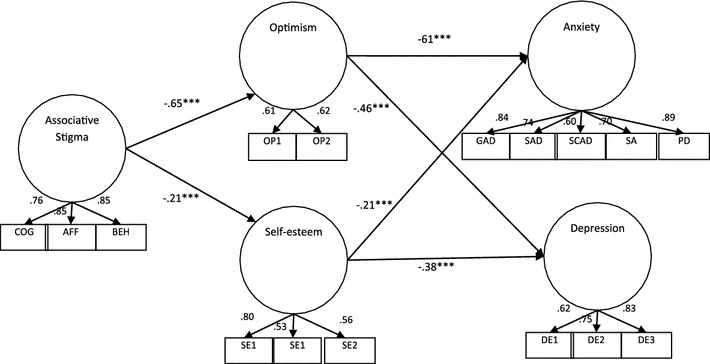


Measurement and structural model for associative stigma, self-esteem, optimism, anxiety and depression among children of parents with HIV/AIDS. All factor loadings were significant at the *p* < .001 level. ****p* < .001. *COG* cognitive component of associative stigma, *AFF* affective component of associative stigma, *BEH* behavioral component of associative stigma, *OP1* optimism parcel 1, *OP2* optimism parcel 2, *SE1* self-esteem parcel 1, *SE2* self-esteem parcel 2, *SE3* self-esteem parcel 3, *DE1* depression factor 1, *DE2* depression factor 2, *DE3* depression factor3, *GAD* generalised anxiety disorder, *SAD* separation anxiety disorder, *SCAD* social anxiety disorder, *SA* school avoidance disorder, *PD* panic disorder


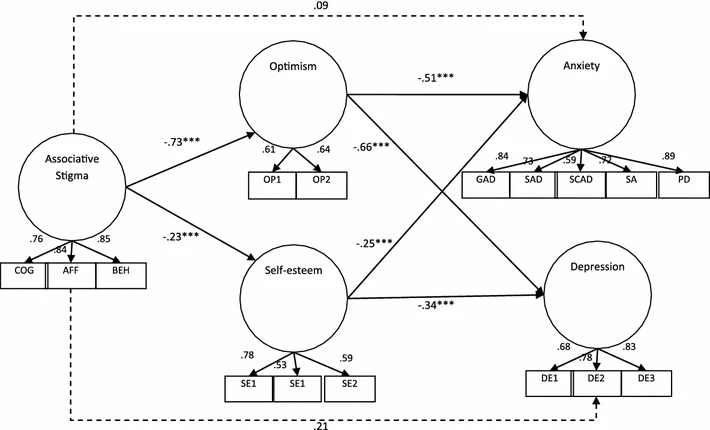


Testing of indirect effects of associative stigma on depression and anxiety among children of parents with HIV/AIDS. All path coefficients shown were averages from 1,000 bootstrap samples and were standardised. Paths shown in dashed lines were added to test direct effects of associative stigma on anxiety and depression. All factor loadings were significant at the *p* < .001 level. ****p* < .001. *COG* cognitive component of associative stigma, *AFF* affective component of associative stigma, *BEH* behavioral component of associative stigma, *OP1* optimism parcel 1, *OP2* optimism parcel 2, *SE1* self-esteem parcel 1, *SE2* self-esteem parcel 2, *SE3* self-esteem parcel 3, *DE1* depression factor 1, *DE2* depression factor 2, *DE3* depression factor3, *GAD* generalised anxiety disorder, *SAD* separation anxiety disorder, *SCAD* social anxiety disorder, *SA* school avoidance disorder, *PD* panic disorder

Nyblade L, Mbuya-Brown RJ, Ezekiel MJ, et al. A total facility approach to reducing HIV stigma in health facilities: implementation process and lessons learned. *AIDS.* 2020;34 Suppl 1:S93-S102. <https://www.ncbi.nlm.nih.gov/pubmed/32881798>

Overview of three-stage ‘total facility’ stigma-reduction approach.


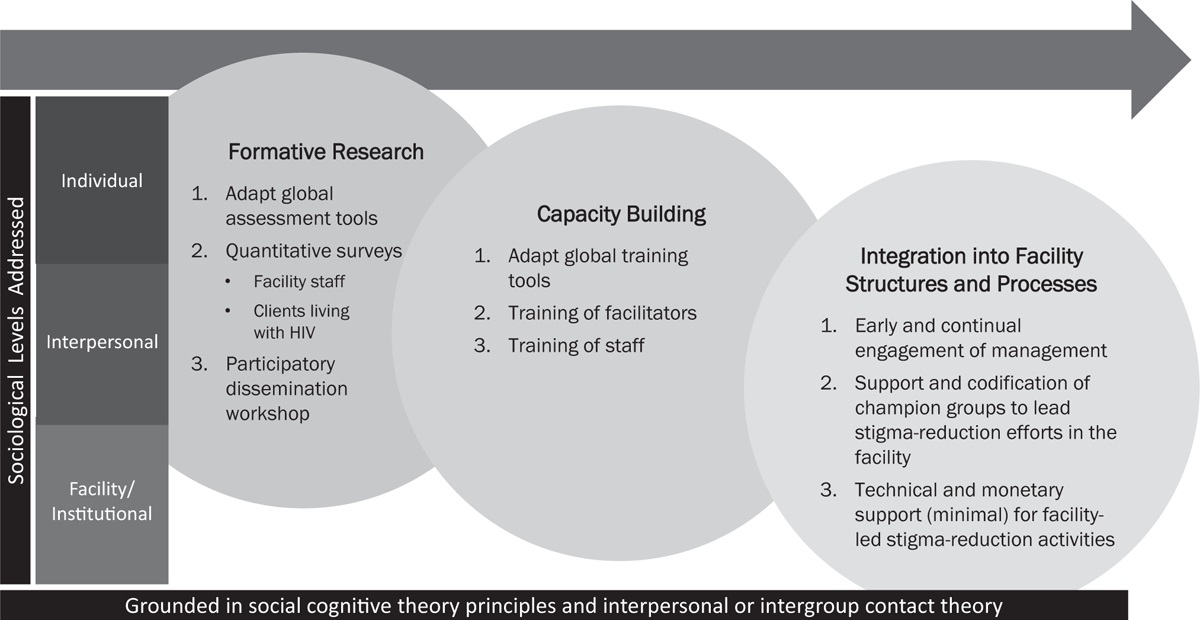


Pantelic M, Boyes M, Cluver L, Meinck F. HIV, violence, blame and shame: pathways of risk to internalized HIV stigma among South African adolescents living with HIV. *J Int AIDS Soc.* 2017;20(1):21771. <https://www.ncbi.nlm.nih.gov/pubmed/28853517>

Hypothesized risk pathways from HIV-related disability to internalized HIV stigma.


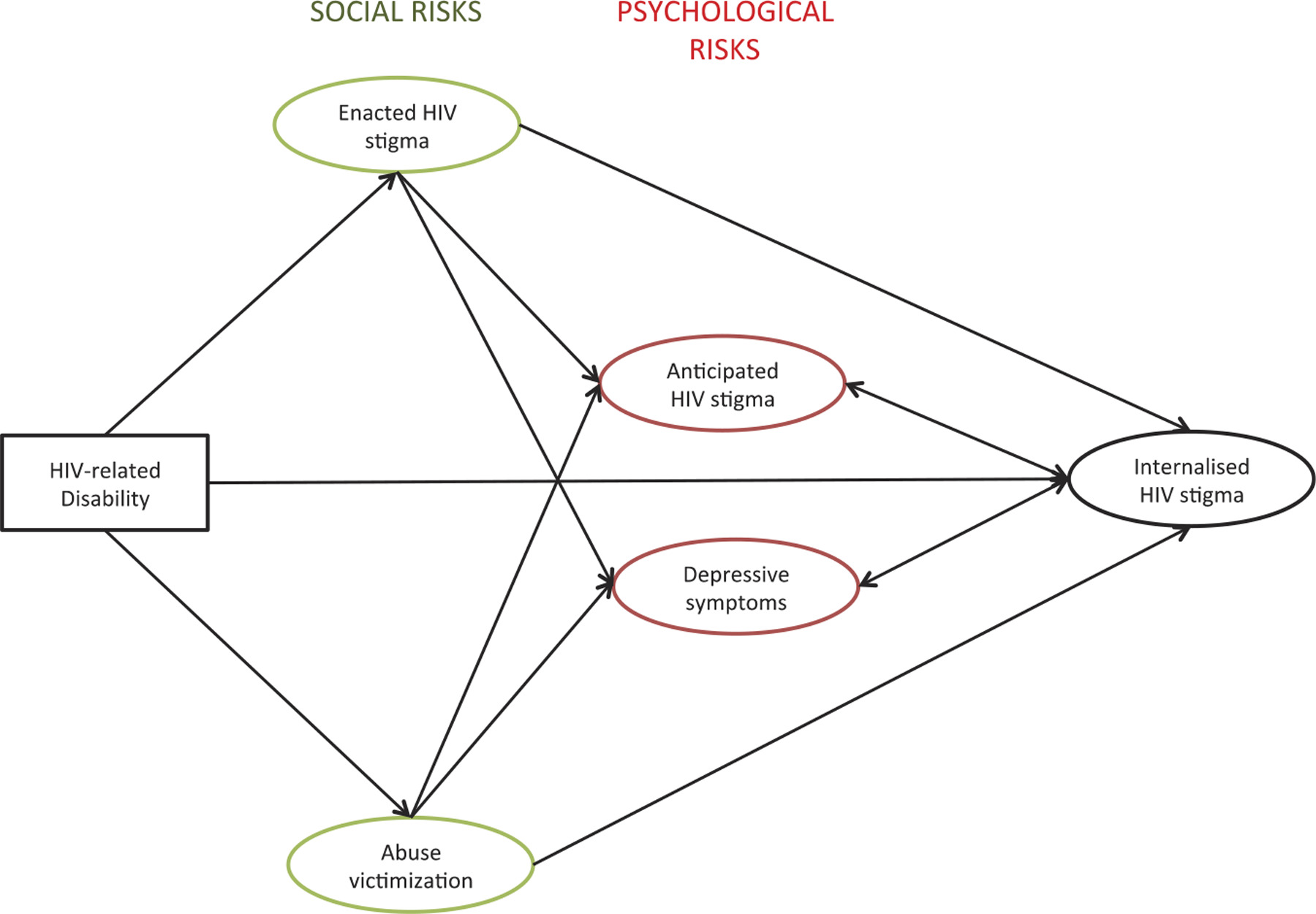


Final structural equation model results. Rectangular shape signifies an observed variable whereas ovals mark latent variables. Values indicate standardized *β* weights. Dotted lines indicate hypothesized pathways that were non-significant. Full lines indicate pathways that were significant. *** indicates *p* < .001; ** indicates *p* < .005; * indicates *p* < .05. Model fit: RMSEA: .023; CFI: .94; TLI: .95; WRMR: 1.070. Model controlled for age, gender, rural household location.


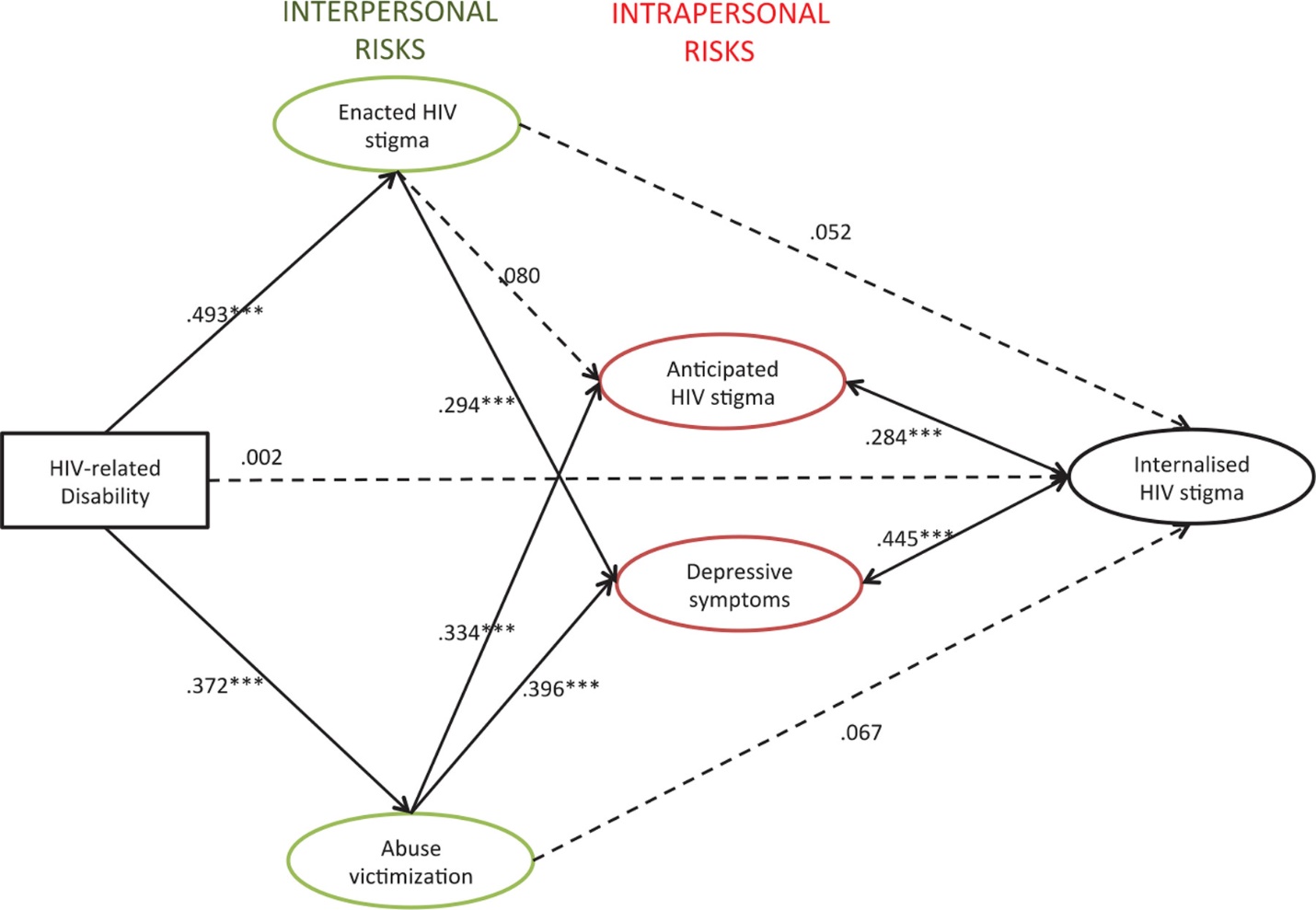


Pescosolido BA, Martin JK. The Stigma Complex. *Annu Rev Sociol.* 2015;41:87-116. <https://www.ncbi.nlm.nih.gov/pubmed/26855471>


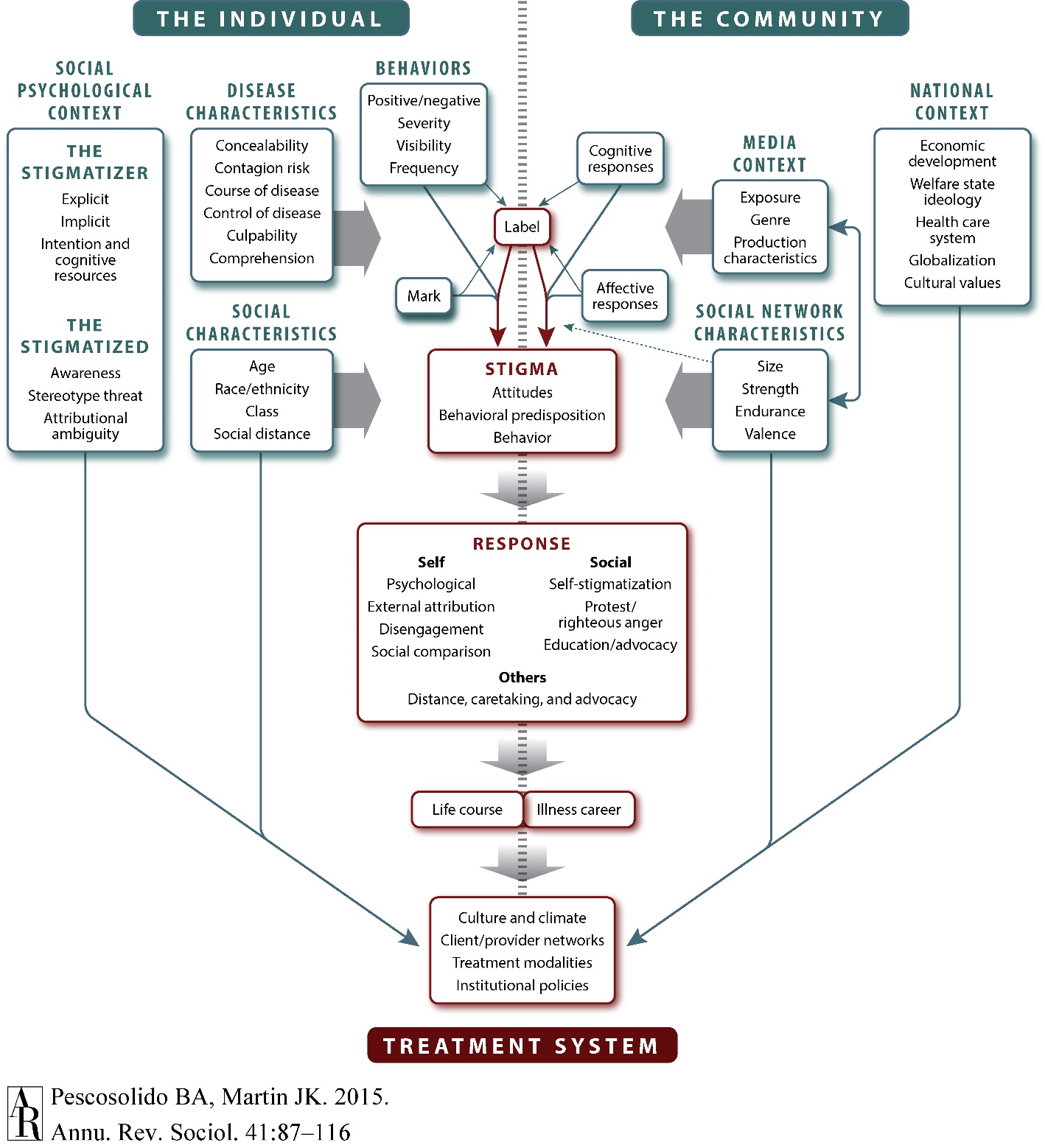


Framework Integrating Normative Influence on Stigma (FINIS). Adapted and reprinted with permission from Pescosolido et al. 2008b.

Pham HN, Protsiv M, Larsson M, Ho HT, de Vries DH, Thorson A. Stigma, an important source of dissatisfaction of health workers in HIV response in Vietnam: a qualitative study. *BMC Health Serv Res.* 2012;12:474. <https://www.ncbi.nlm.nih.gov/pubmed/23259923>


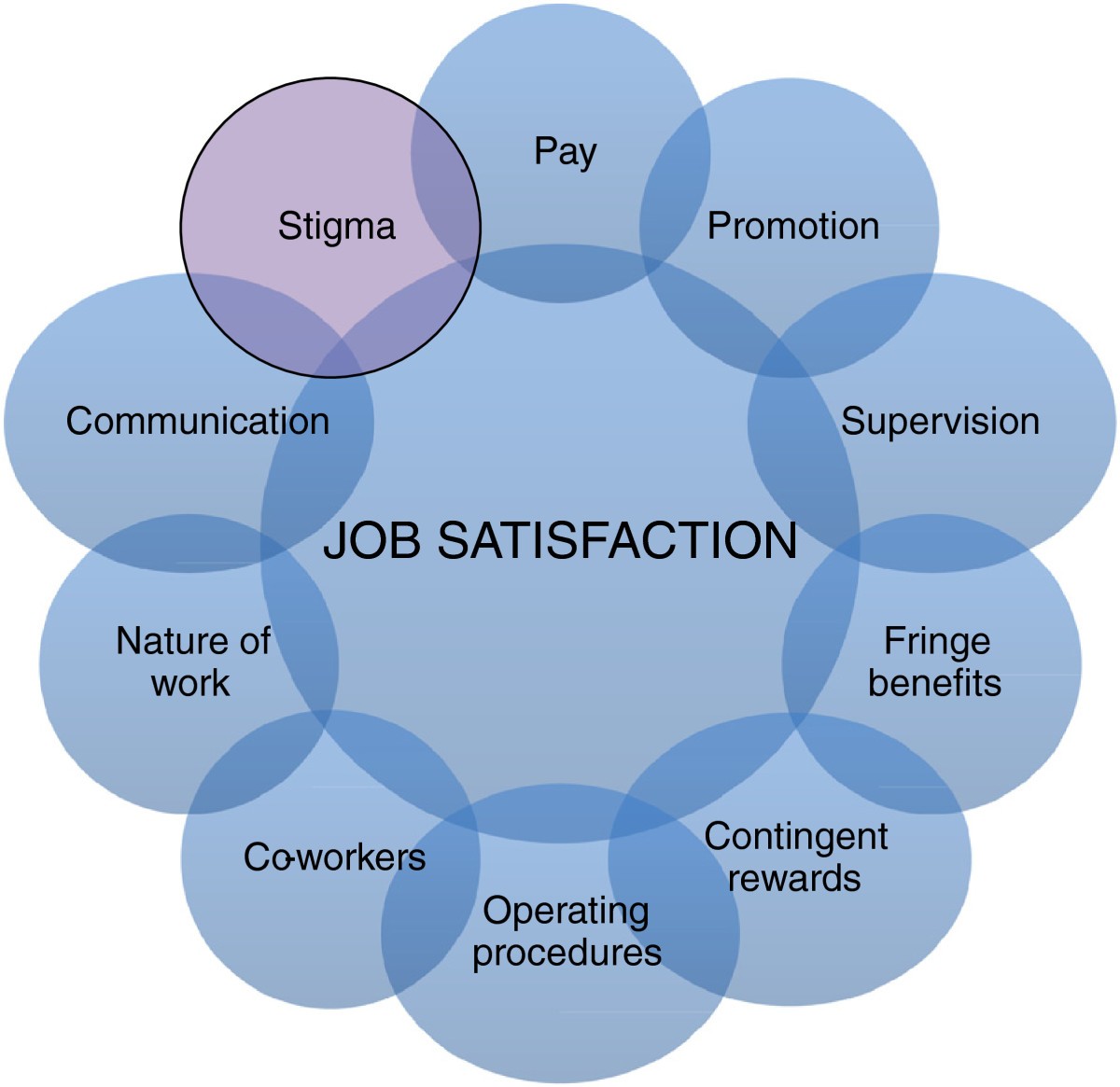


Adjusted Spector’s job satisfaction model of health workers in HIV service organizations.

Prati G, Zani B, Pietrantoni L, et al. The role of knowing someone living with HIV/AIDS and HIV disclosure in the HIV stigma framework: A Bayesian mediation analysis. *Quality & Quantity: International Journal of Methodology.* 2016;50(2):637-651. <https://link.springer.com/article/10.1007/s11135-015-0168-2>


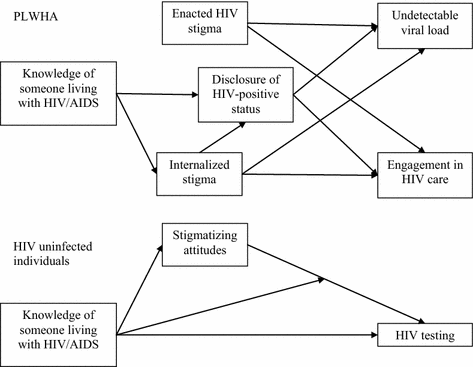


Conceptual models for HIV uninfected individuals and PLWHA


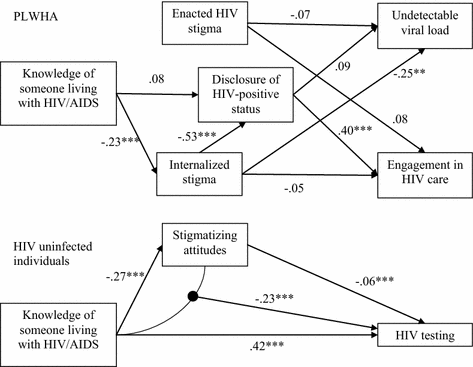


Standardized coefficients for path models. ∗p<0.05;∗∗p<0.01;∗∗∗p<0.001

Pulerwitz J, Oanh KT, Akinwolemiwa D, Ashburn K, Nyblade L. Improving hospital-based quality of care by reducing HIV-related stigma: evaluation results from Vietnam. *AIDS Behav.* 2015;19(2):246-256. <https://www.ncbi.nlm.nih.gov/pubmed/25382350>


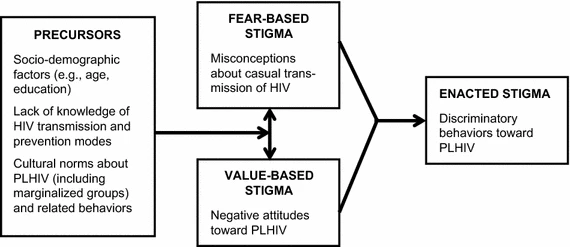


Rao D, Feldman BJ, Fredericksen RJ, et al. A structural equation model of HIV-related stigma, depressive symptoms, and medication adherence. *AIDS Behav.* 2012;16(3):711-716. <https://www.ncbi.nlm.nih.gov/pubmed/21380495>

# Depicts the relationships between latent variables analyzed in the model. The latent variables are depicted as *circles* and the observed variables (i.e. items) as *squares* (questionnaire items labeled depressive symptoms 1–9 and stigma 1–4). Stigma is associated with depressive symptoms, which in turn is associated with adherence. The path coefficients, which can be interpreted as standardized beta weights in a regression model, are shown next to each *arrow*. The *arrow* with the *dotted line* represents the effect between stigma and adherence that is diminished when depressive symptoms are included in the model, indicating that depressive symptoms partially mediate the relationship between stigma and adherence. Models were adjusted for age, sex, and race


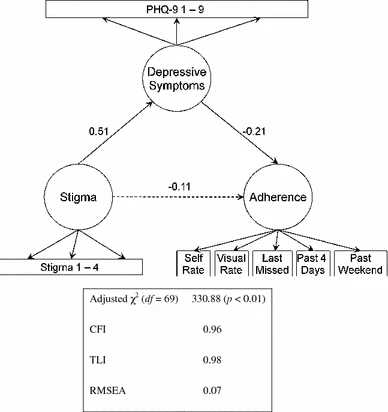


Relf MV, Pan W, Edmonds A, Ramirez C, Amarasekara S, Adimora AA. Discrimination, Medical Distrust, Stigma, Depressive Symptoms, Antiretroviral Medication Adherence, Engagement in Care, and Quality of Life Among Women Living With HIV in North Carolina: A Mediated Structural Equation Model. *J Acquir Immune Defic Syndr.* 2019;81(3):328-335. <https://www.ncbi.nlm.nih.gov/pubmed/30893124>

The hypothesized structural equation model.


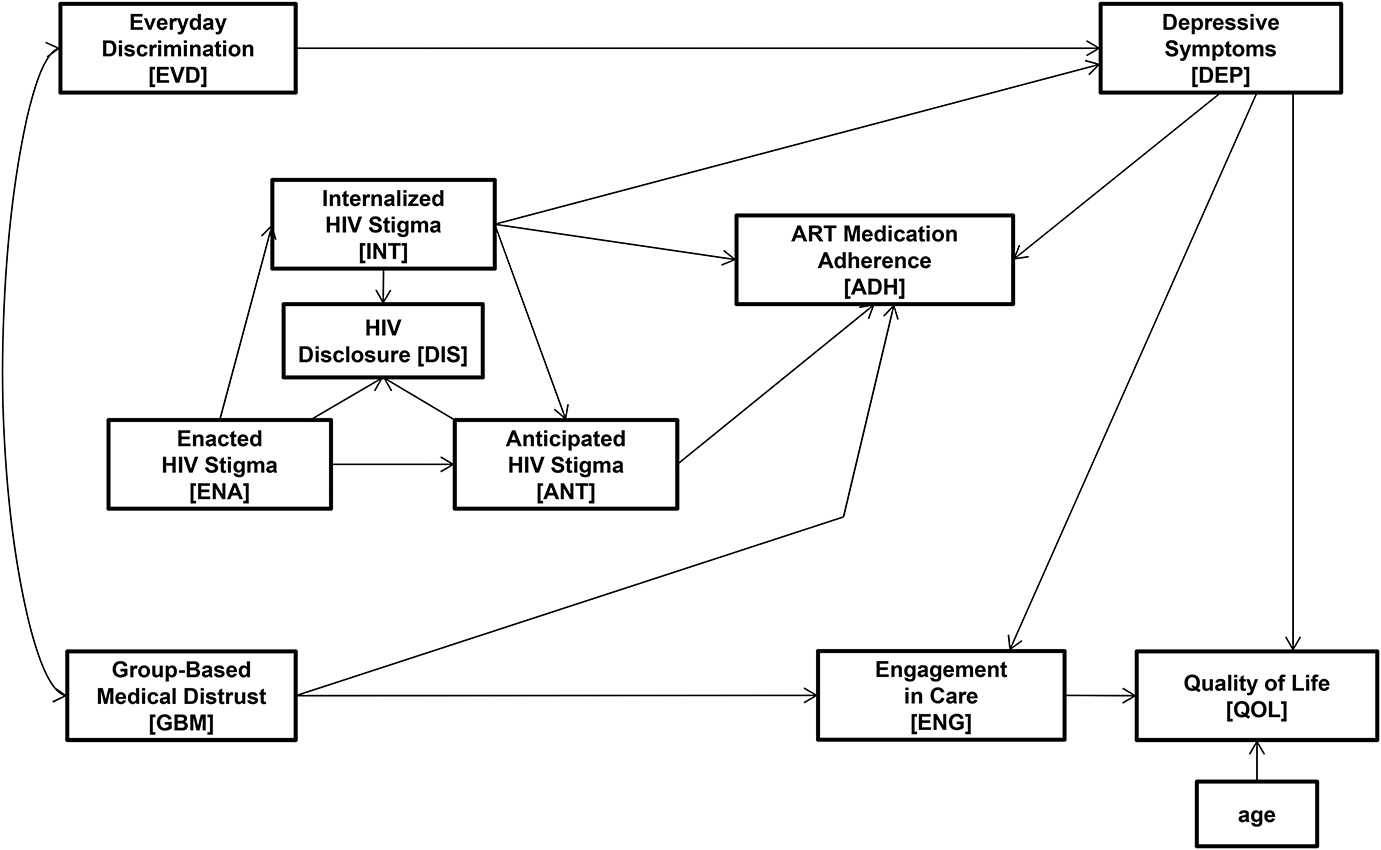


The final fitted structural equation model with stigma as a multidimensional construct. Fit indices for model: χ^2^ = 35.004, df = 39, P = 0.653; χ^2^/df = 0.898, GFI = 0.951; NFI = 0.889; IFI = 1.014; RFI = 0.843; CFI = 1.000; RMSEA = 0.000 (90% CI = 0.000–0.53). Dotted pathways were newly identified during stepwise model generation. CFI, comparative fit index; CI, confidence interval; GFI, goodness of fit index; IFI, incremental fit index; NFI, normed fit index; RFI, relative fit index; RMSEA; root mean square error of approximation.


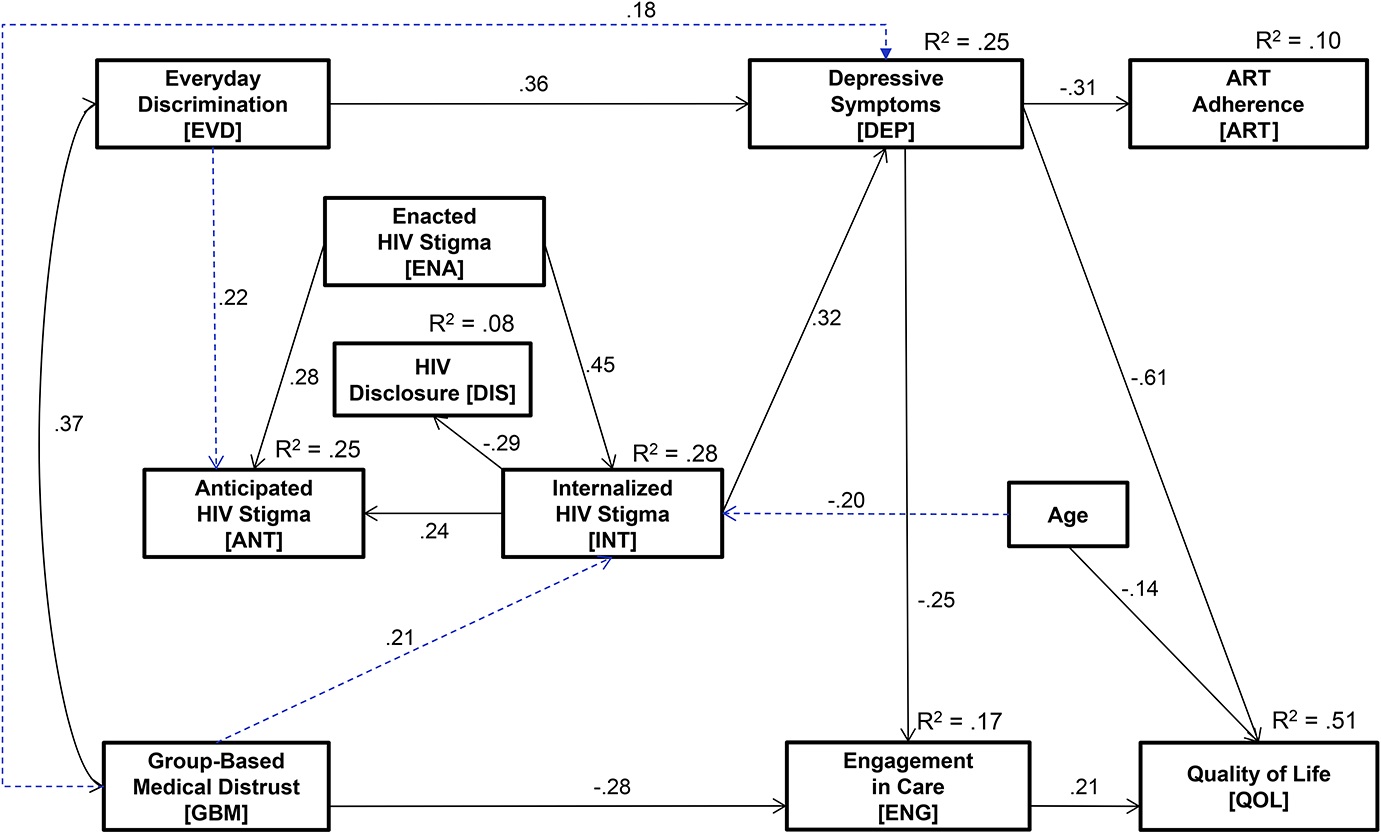


Rice WS, Crockett KB, Mugavero MJ, Raper JL, Atkins GC, Turan B. Association Between Internalized HIV-Related Stigma and HIV Care Visit Adherence. *J Acquir Immune Defic Syndr.* 2017;76(5):482-487. <https://www.ncbi.nlm.nih.gov/pubmed/28885270>

Visit adherence mediates the effect of internalized stigma on medication adherence.


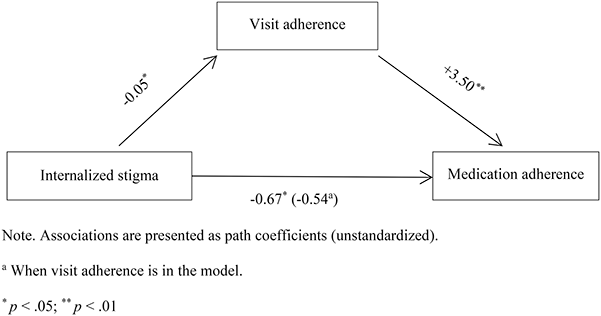


Sen S, Aguilar JP, Petty M. An ecological framework for understanding HIV- and AIDS-related stigma among Asian American and Pacific Islander men who have sex with men living in the USA. *Cult Health Sex.* 2021;23(1):85-97. <https://www.ncbi.nlm.nih.gov/pubmed/32031498>

Thematic representation of the relational model between HIV testing and related help seeking behaviours and HIV-related Stigma (micro, meso and macro levels) guided by Bronfenbrenner’s ([1979](https://www-tandfonline-com.libproxy2.usc.edu/doi/figure/10.1080/13691058.2019.1690164?scroll=top&needAccess=true)) ecological systems model.


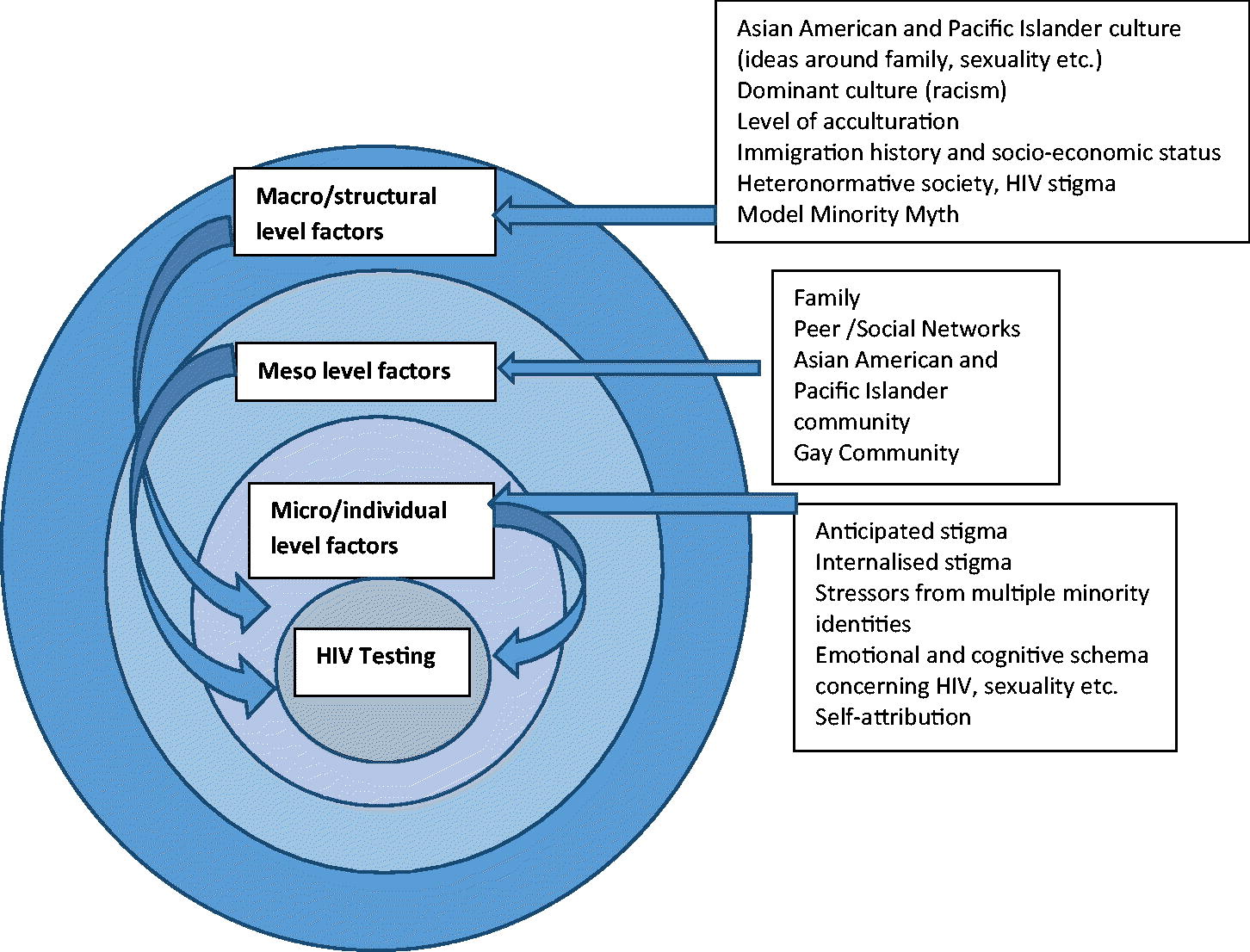


Shin SS, Carpenter CL, Ekstrand ML, et al. Household food insecurity as mediator of the association between internalized stigma and opportunistic infections. *AIDS and Behavior.* 2018;22(12):3897-3904. <https://www.ncbi.nlm.nih.gov/pubmed/29934793>


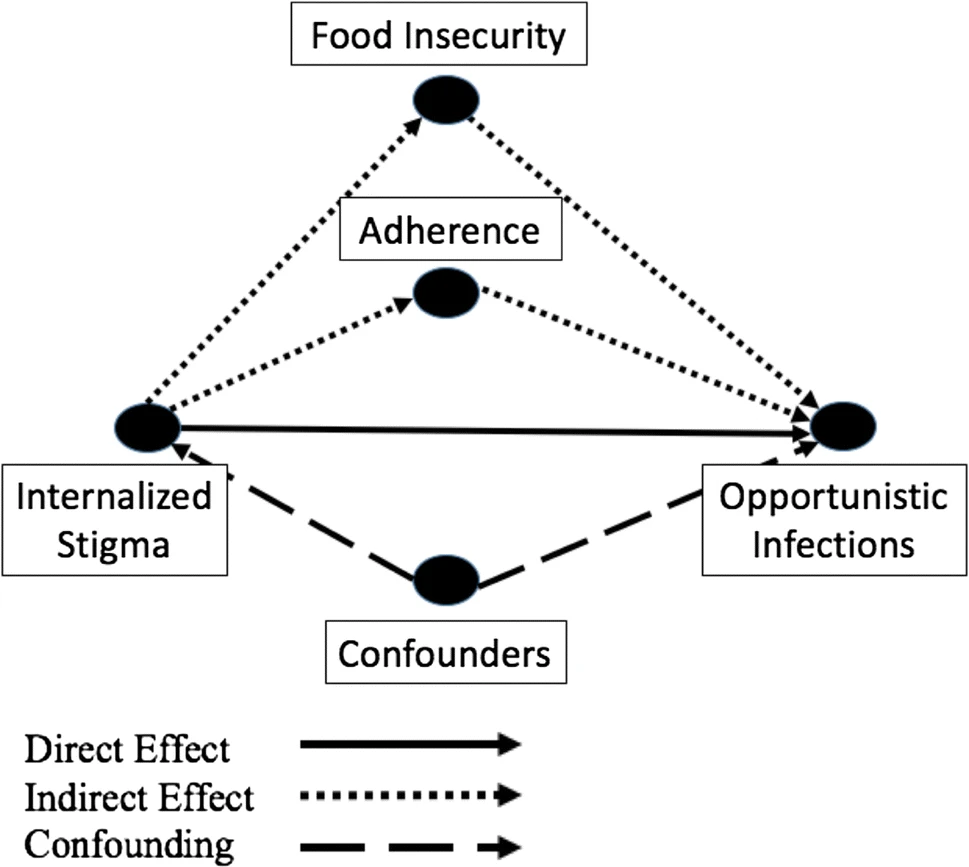


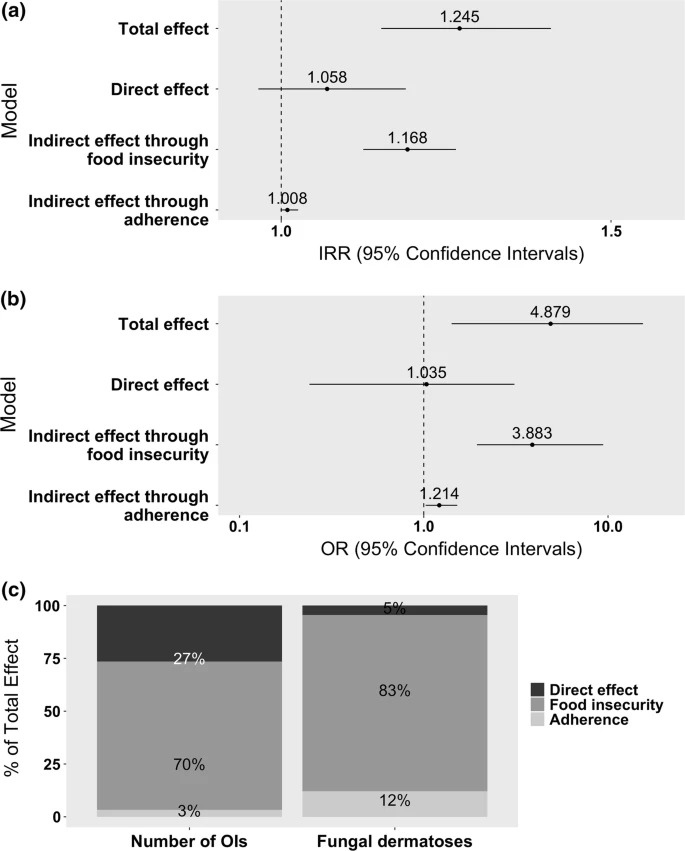


Slater LZ, Moneyham L, Vance DE, Raper JL, Mugavero MJ, Childs G. The multiple stigma experience and quality of life in older gay men with HIV. *J Assoc Nurses AIDS Care.* 2015;26(1):24-35. <https://www.ncbi.nlm.nih.gov/pubmed/25249266>

Visual representation of the relationships that emerged from regression analysis using standardized betas.


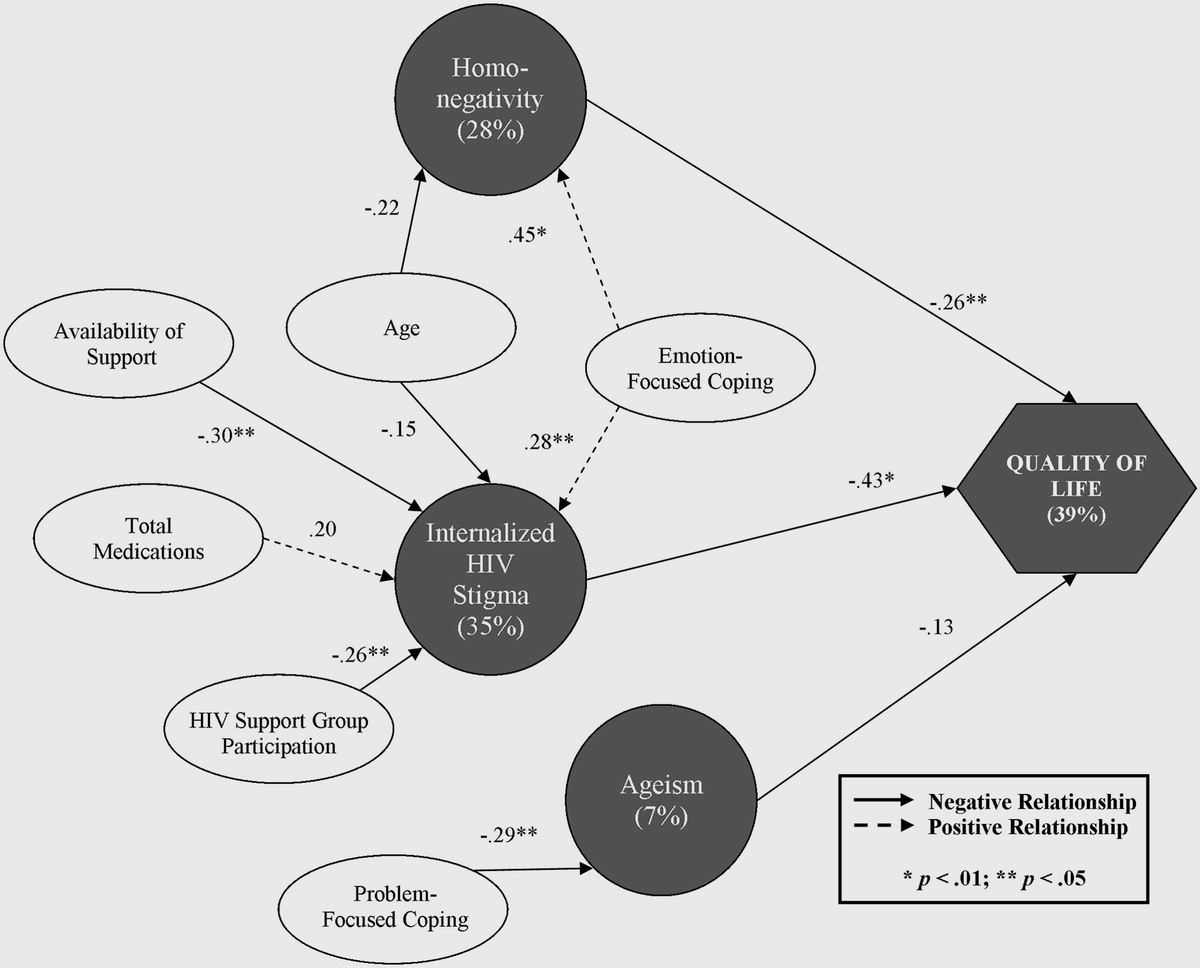


Stangl AL, Earnshaw VA, Logie CH, et al. The Health Stigma and Discrimination Framework: a global, crosscutting framework to inform research, intervention development, and policy on health-related stigmas. *BMC Med.* 2019;17(1):31. <https://www.ncbi.nlm.nih.gov/pubmed/30764826>


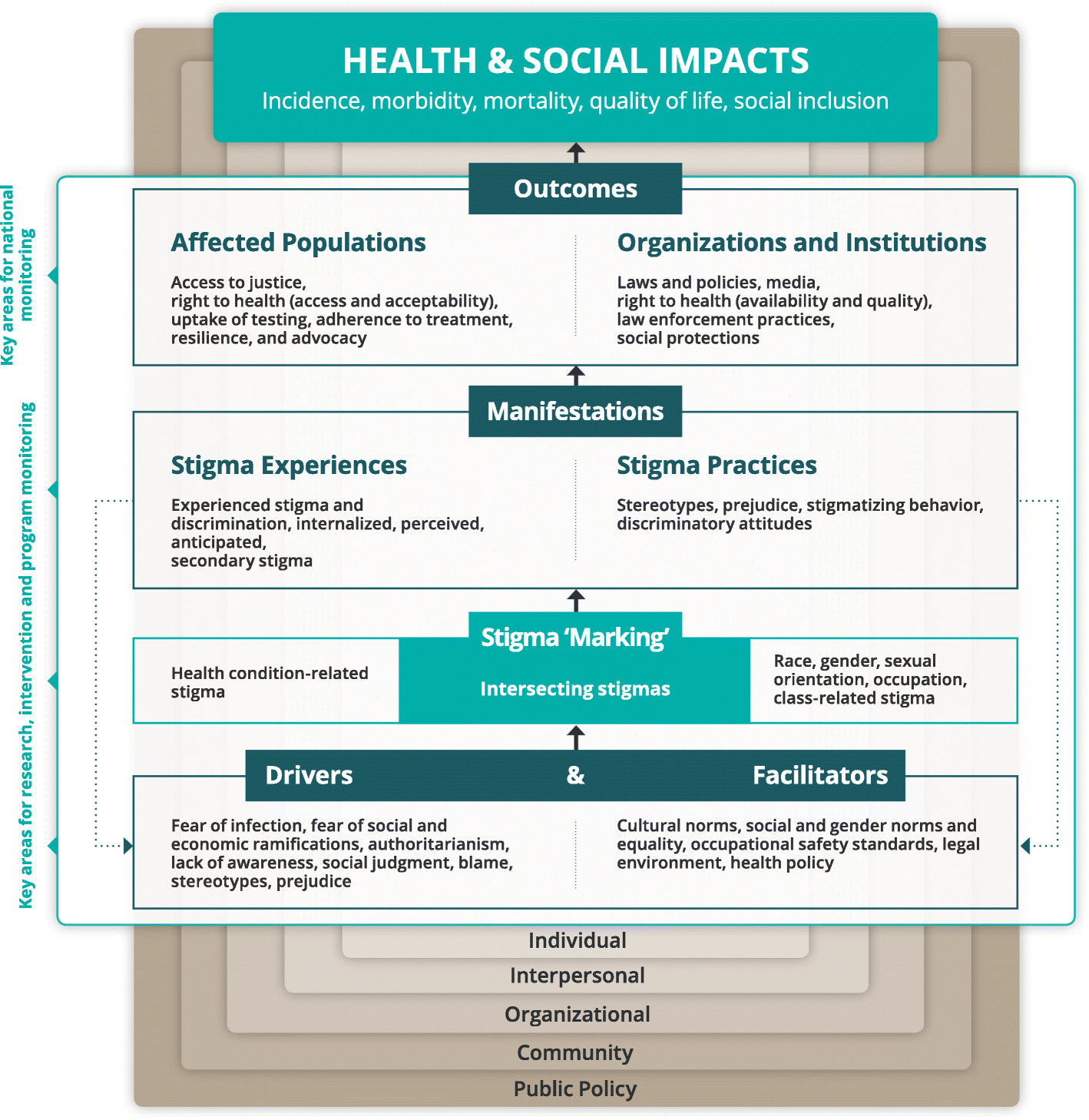


Stevens ME, Parsons JA, Read SE, Nixon SA. The conceptualization of stigma within a rehabilitation framework using HIV as an example. *Disabil Rehabil.* 2019;41(2):235-243. <https://www.ncbi.nlm.nih.gov/pubmed/28978242>

Enacted stigma and its relationship with the International Classification of Functioning, Disability and Health.

**
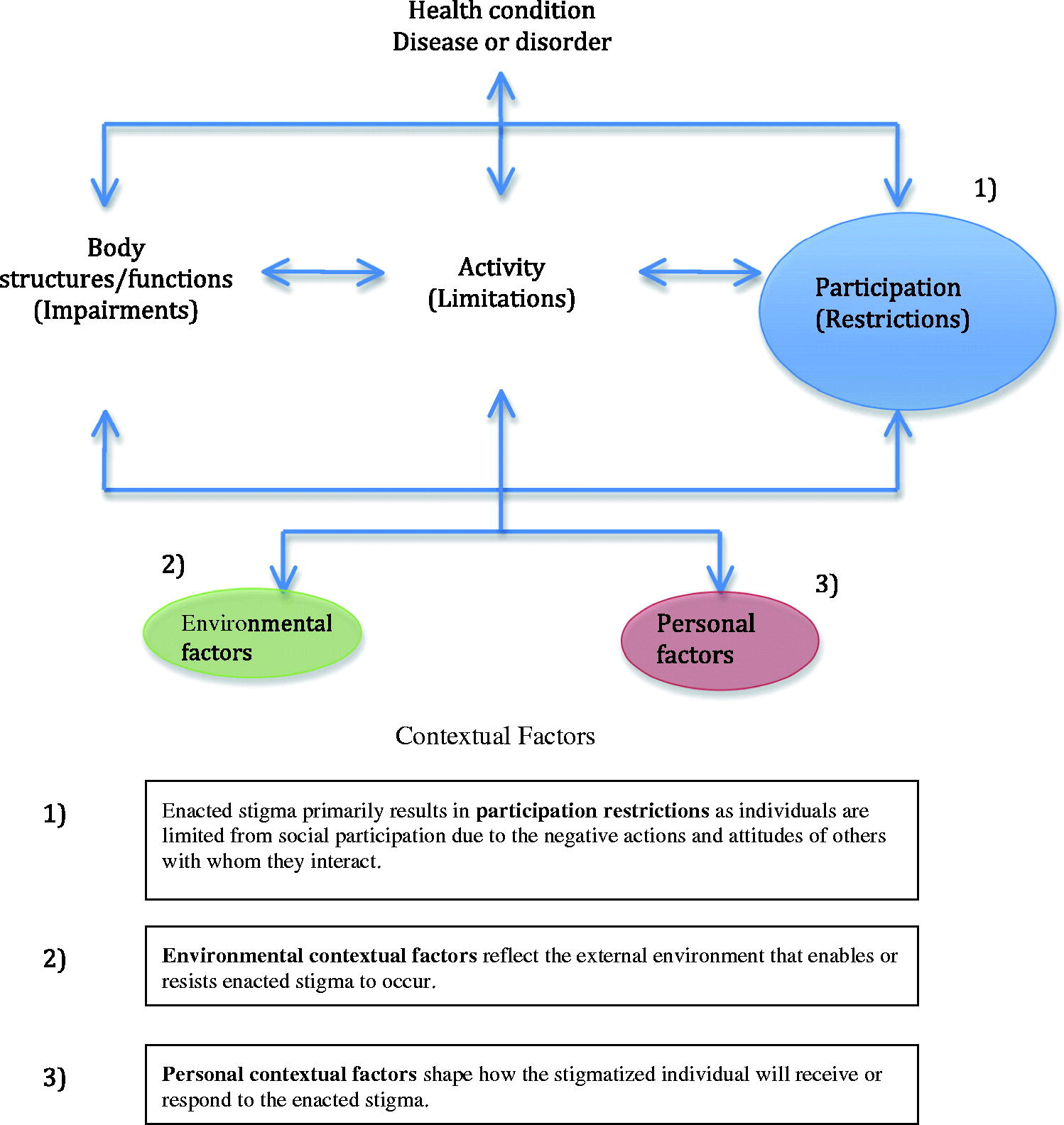
**

Steward WT, Herek GM, Ramakrishna J, et al. HIV-related stigma: adapting a theoretical framework for use in India. *Soc Sci Med.* 2008;67(8):1225-1235. <https://www.ncbi.nlm.nih.gov/pubmed/18599171>


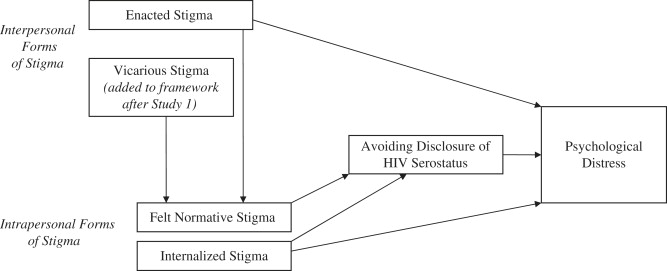


Theoretical framework linking facets of stigma, avoidance of HIV serostatus disclosure, and psychological distress.


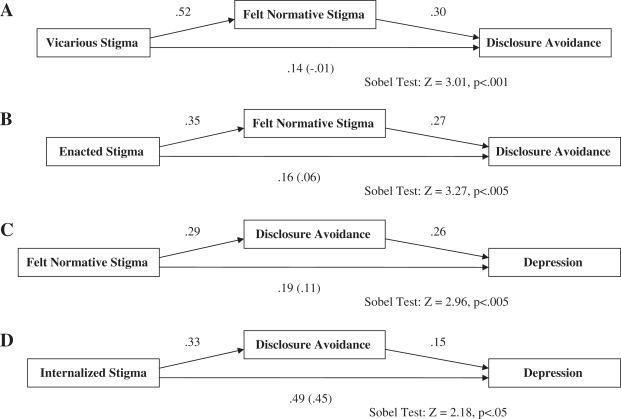


Mediation analyses examining associations among stigma, disclosure avoidance, and depression.

Thapa S, Hannes K, Cargo M, Buve A, Aro AR, Mathei C. Building a Conceptual Framework to Study the Effect of HIV Stigma-Reduction Intervention Strategies on HIV Test Uptake: A Scoping Review. *J Assoc Nurses AIDS Care.* 2017;28(4):545-560. <https://www.ncbi.nlm.nih.gov/pubmed/28473183>

## **Purposeful sampling strategies.**


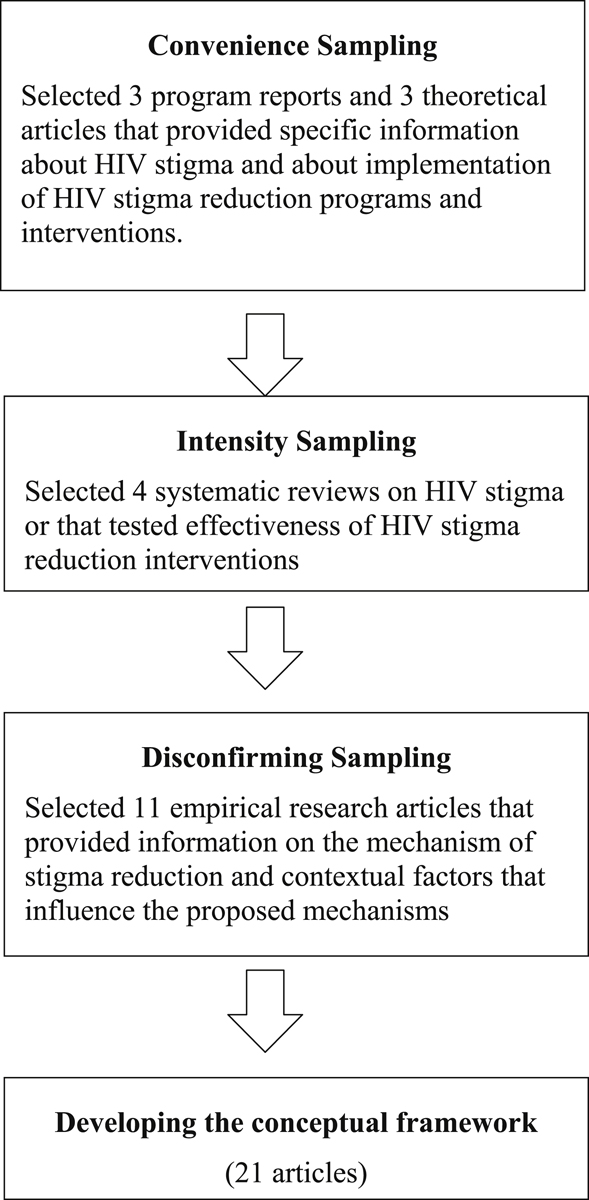


## **Conceptual framework explaining the effect of stigma-reduction intervention strategies on HIV test uptake.**

*Note.*PLWH = people living with HIV infection.
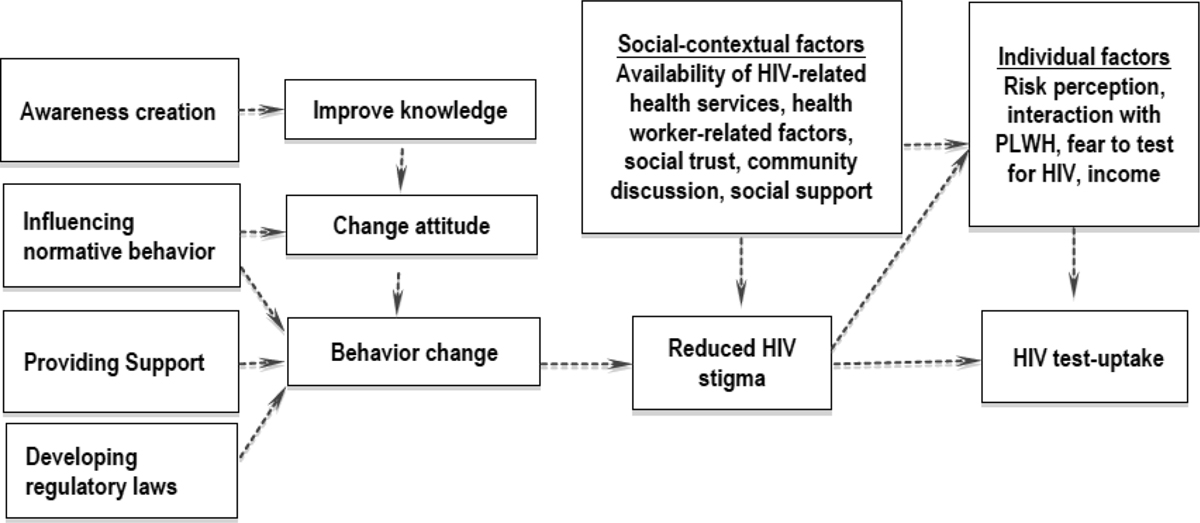


Thi MD, Brickley DB, Vinh DT, et al. A qualitative study of stigma and discrimination against people living with HIV in Ho Chi Minh City, Vietnam. *AIDS Behav.* 2008;12(4 Suppl):S63-70. <https://www.ncbi.nlm.nih.gov/pubmed/18360743>

# Schematic diagram of stigma and discrimination against PLHIV in Ho Chi Minh City: causes, effects and relationships


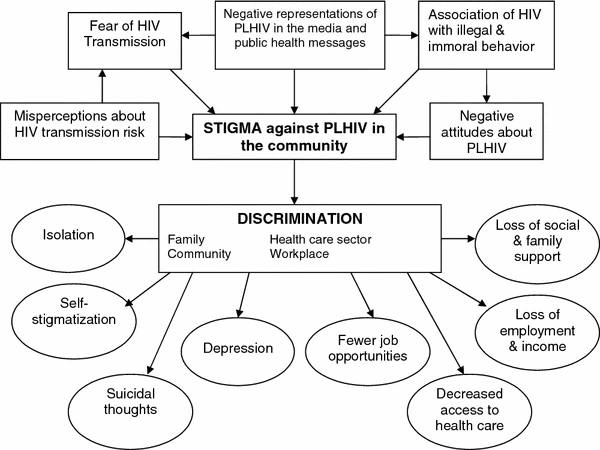


Thrasher AD, Earp JA, Golin CE, Zimmer CR. Discrimination, distrust, and racial/ethnic disparities in antiretroviral therapy adherence among a national sample of HIV-infected patients. *J Acquir Immune Defic Syndr.* 2008;49(1):84-93. <https://www.ncbi.nlm.nih.gov/pubmed/18667919>


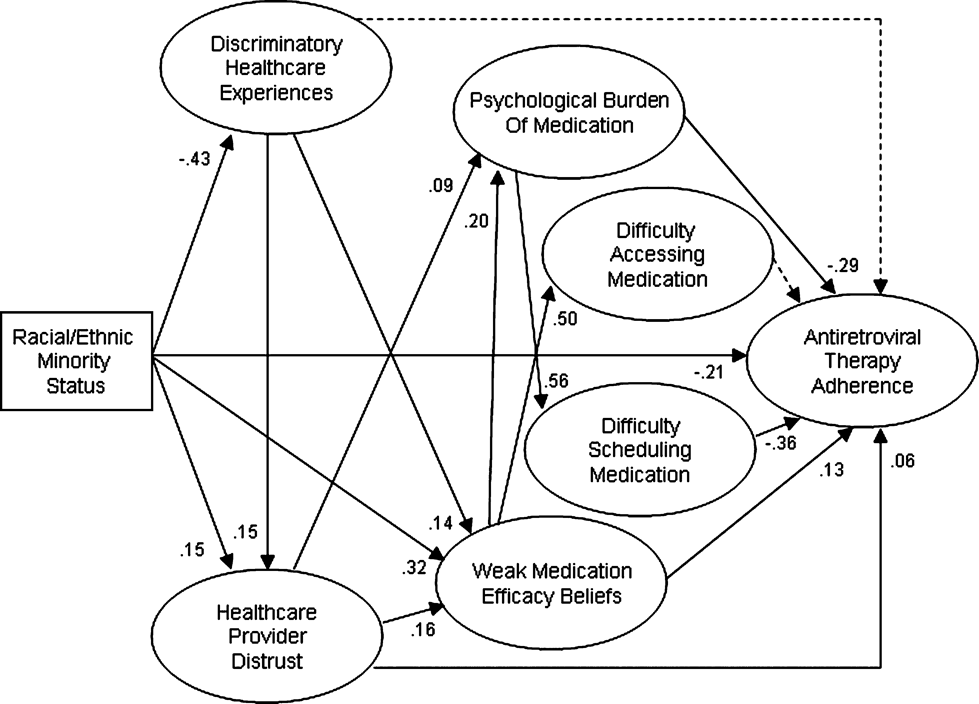


Tsai AC, Bangsberg DR, Kegeles SM, et al. Internalized stigma, social distance, and disclosure of HIV seropositivity in rural Uganda. *Ann Behav Med.* 2013;46(3):285-294. <https://www.ncbi.nlm.nih.gov/pubmed/23690283>

Conceptual model of internalized stigma, social distance, and disclosure of HIV seropositivity


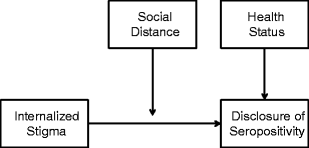


Turan B, Budhwani H, Fazeli PL, et al. How Does Stigma Affect People Living with HIV? The Mediating Roles of Internalized and Anticipated HIV Stigma in the Effects of Perceived Community Stigma on Health and Psychosocial Outcomes. *AIDS Behav.* 2017;21(1):283-291. <https://www.ncbi.nlm.nih.gov/pubmed/27272742>

# The health stigma framework (HSF) originally proposed by Earnshaw, modified by adding perceived community stigma as an additional stigma mechanism


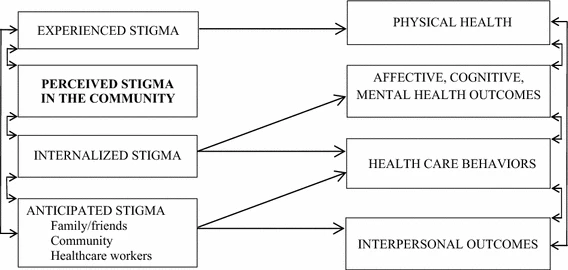


# Internalized stigma mediates the effect of perceived community stigma on self-esteem. Path coefficients are unstandardized (B). Sex, race, age, socio-economic status, and time on ART are also controlled. ^a^When internalized stigma is in the model. ^*^ *p* < 0.05; ^**^ *p* < 0.01


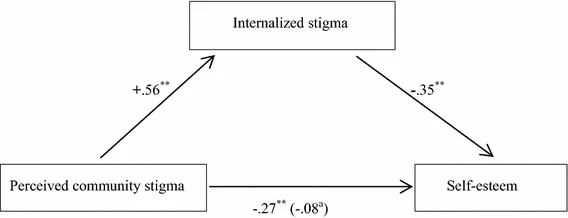


# Internalized stigma and anticipated community stigma mediate the effect of perceived community stigma on medication adherence (serial mediation). Path coefficients are unstandardized (B). Sex, race, age, socio-economic status, and time on ART are also controlled. ^a^When internalized stigma and anticipated community stigma are in the model. ^*^ *p* < 0.05; ^**^ *p* < 0.01


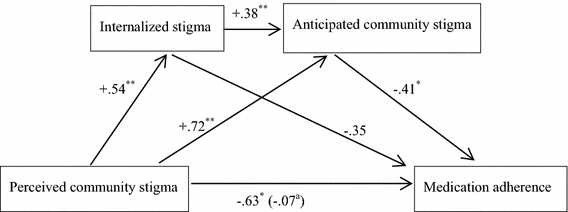


# Internalized stigma and anticipated stigma from friends and family mediate the effect of perceived community stigma on social support (serial mediation). Path coefficients are unstandardized (B). Sex, race, age, socio-economic status, and time on ART are also controlled. ^a^When internalized stigma and anticipated stigma from friends and family are in the model. ^*^ *p* < 0.05; ^**^ *p* < 0.01


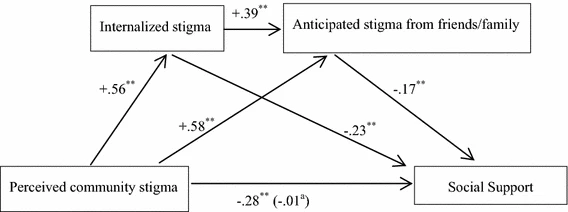


# Internalized stigma and anticipated stigma from healthcare workers mediate the effect of perceived community stigma on trust in physicians (serial mediation). Path coefficients are unstandardized (B). Sex, race, age, socio-economic status, and time on ART are also controlled. When internalized stigma and anticipated stigma from healthcare workers are in the model. ^*^ *p* < 0.05; ^**^ *p* < 0.01


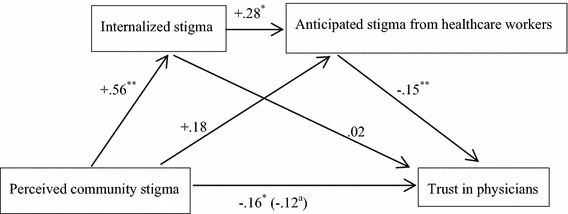


Turan B, Hatcher AM, Weiser SD, Johnson MO, Rice WS, Turan JM. Framing Mechanisms Linking HIV-Related Stigma, Adherence to Treatment, and Health Outcomes. *Am J Public Health.* 2017;107(6):863-869. <https://www.ncbi.nlm.nih.gov/pubmed/28426316>

Conceptual Framework for HIV-Related Stigma, Engagement in Care, and Health Outcome


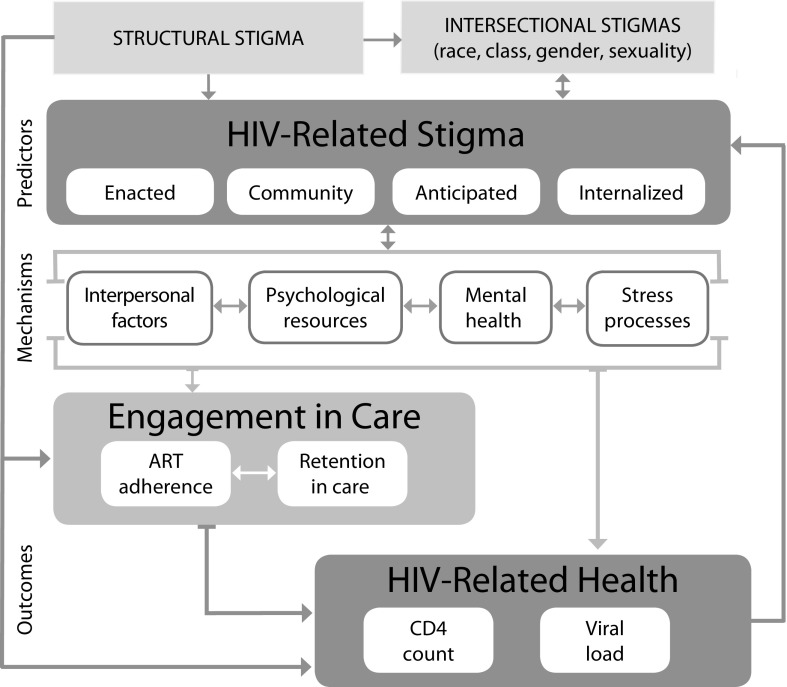


Turan B, Smith W, Cohen MH, et al. Mechanisms for the Negative Effects of Internalized HIV-Related Stigma on Antiretroviral Therapy Adherence in Women: The Mediating Roles of Social Isolation and Depression. *J Acquir Immune Defic Syndr.* 2016;72(2):198-205. <https://www.ncbi.nlm.nih.gov/pubmed/26885803>

Depression mediates the effect of internalized HIV-related stigma on suboptimal medication adherence for racial/ethnic minority groups (ie, non-whites). n = 1029.


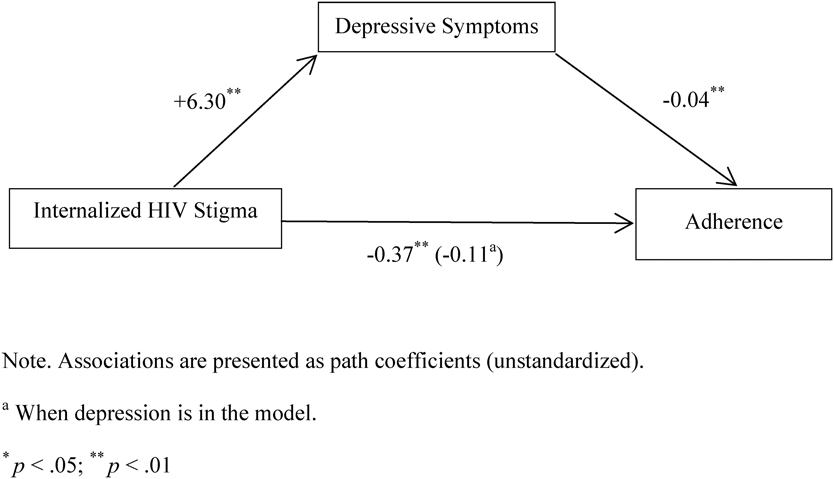


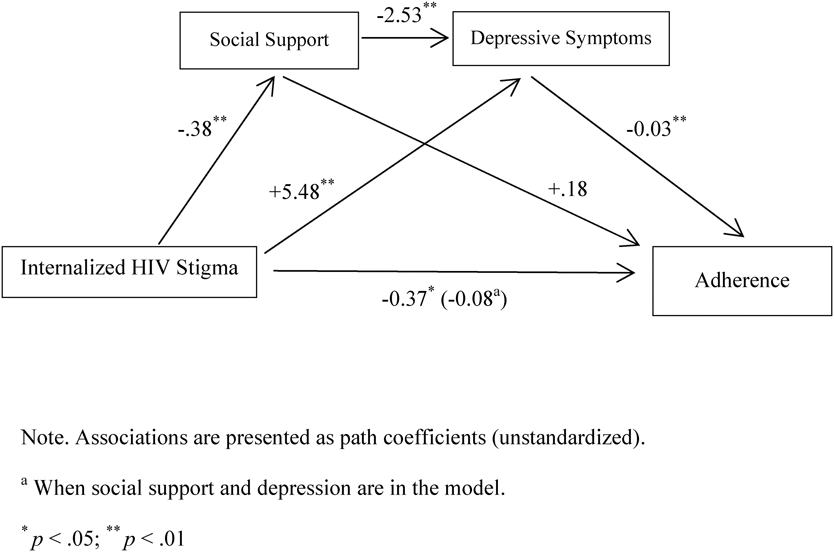


UNAIDS. *Confronting discrimination: Overcoming HIV-related stigma and discrimination in healthcare settings and beyond.* 2017. <https://www.unaids.org/sites/default/files/media_asset/confronting-discrimination_en.pdf>


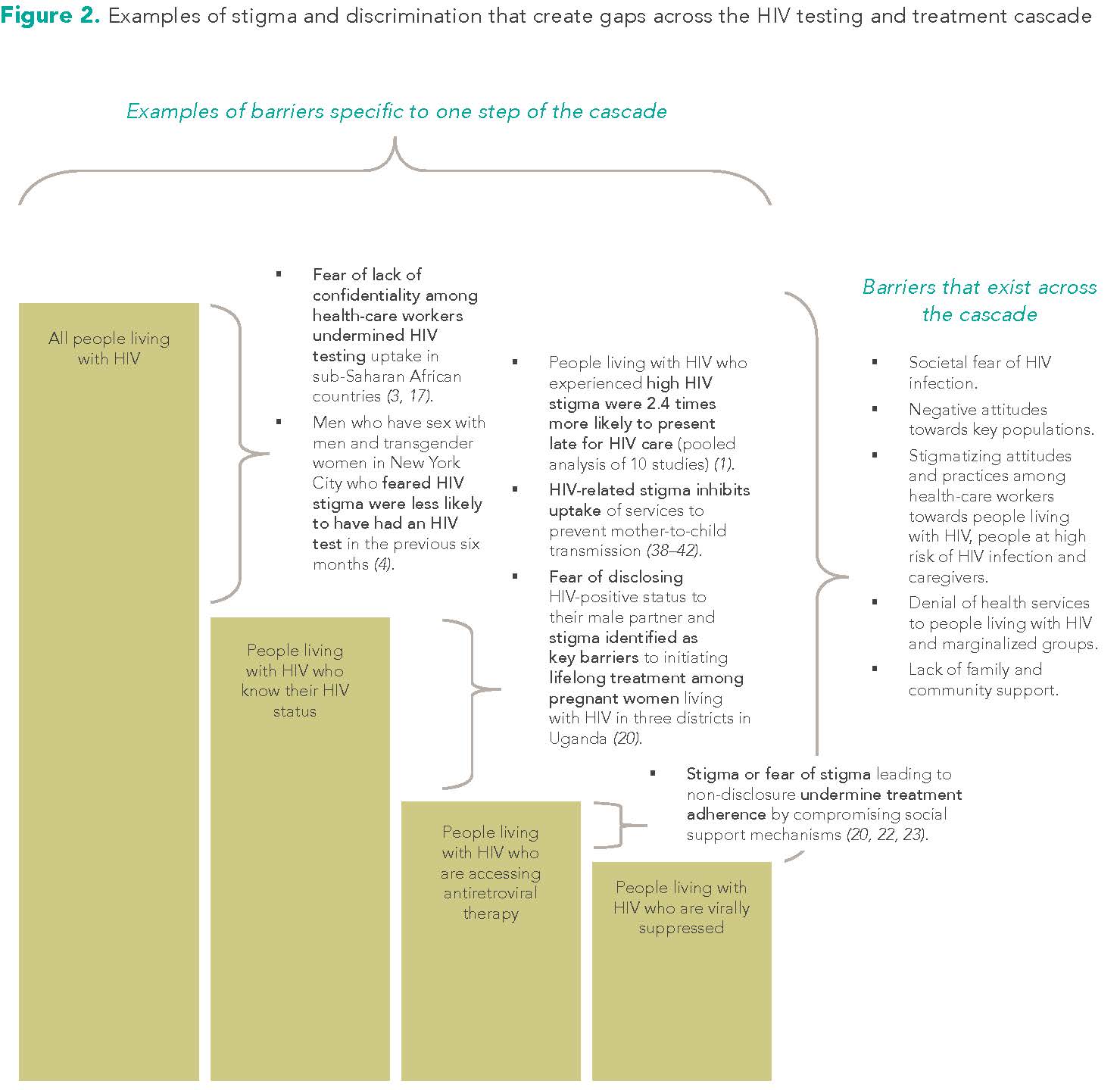


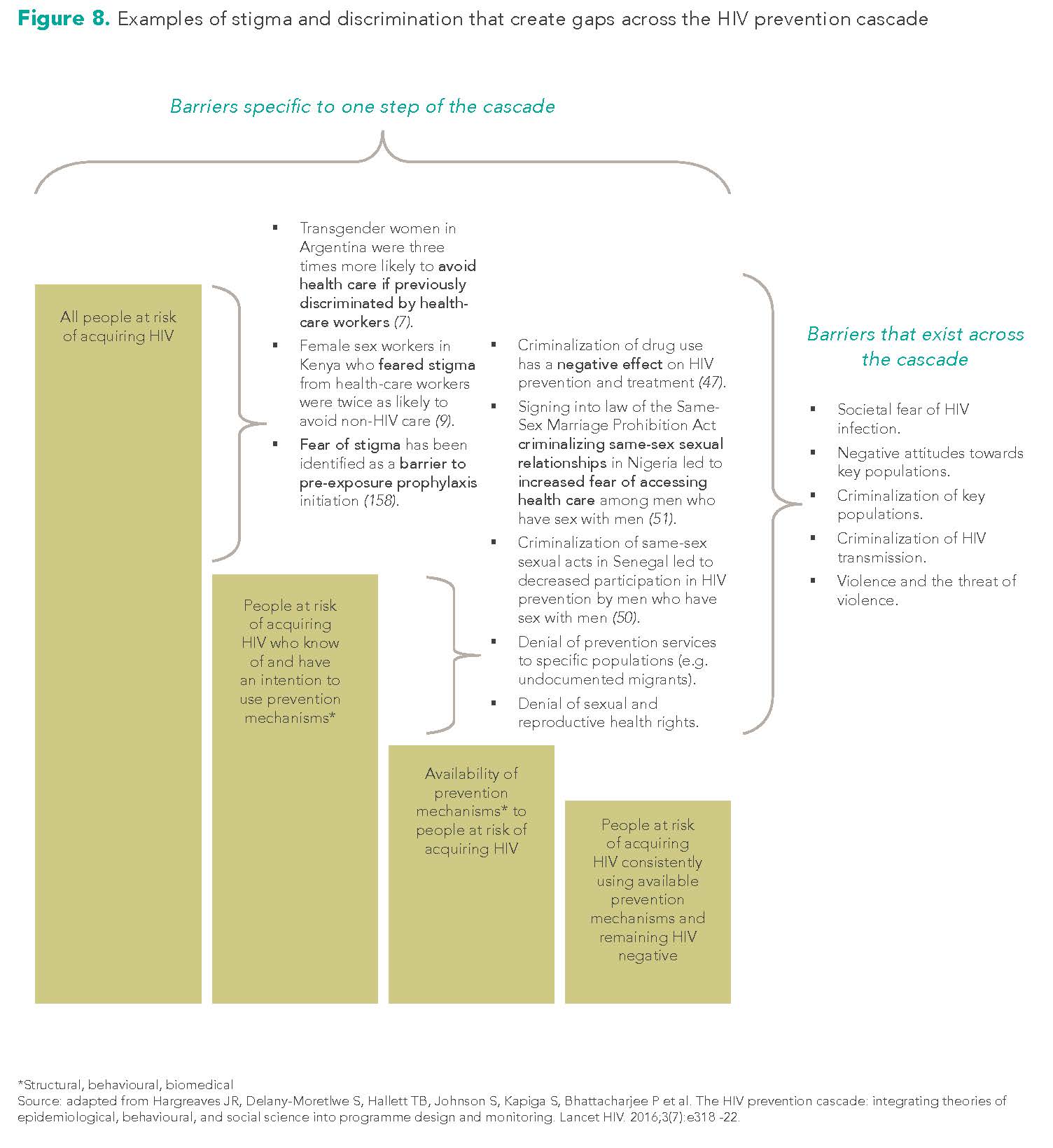


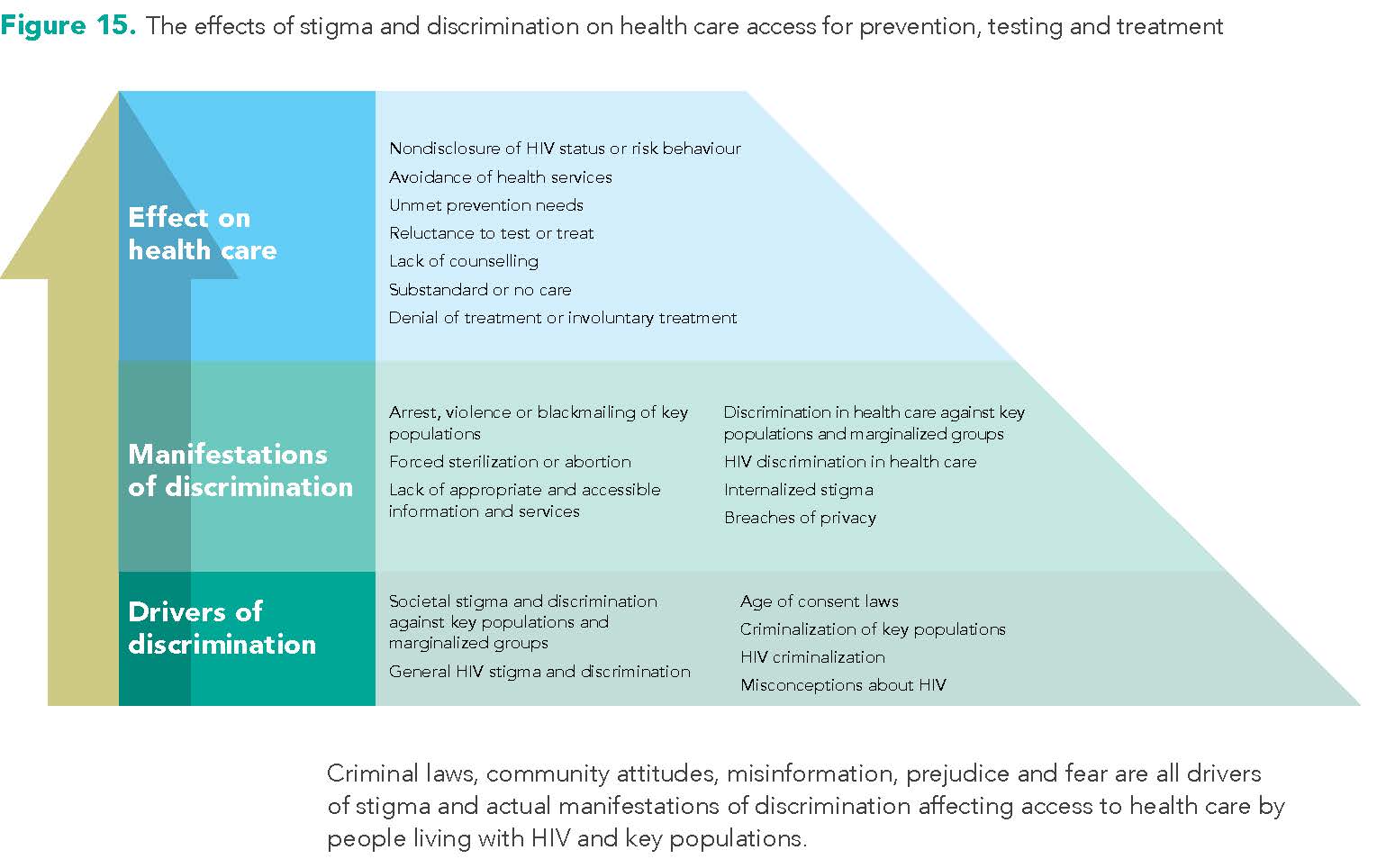


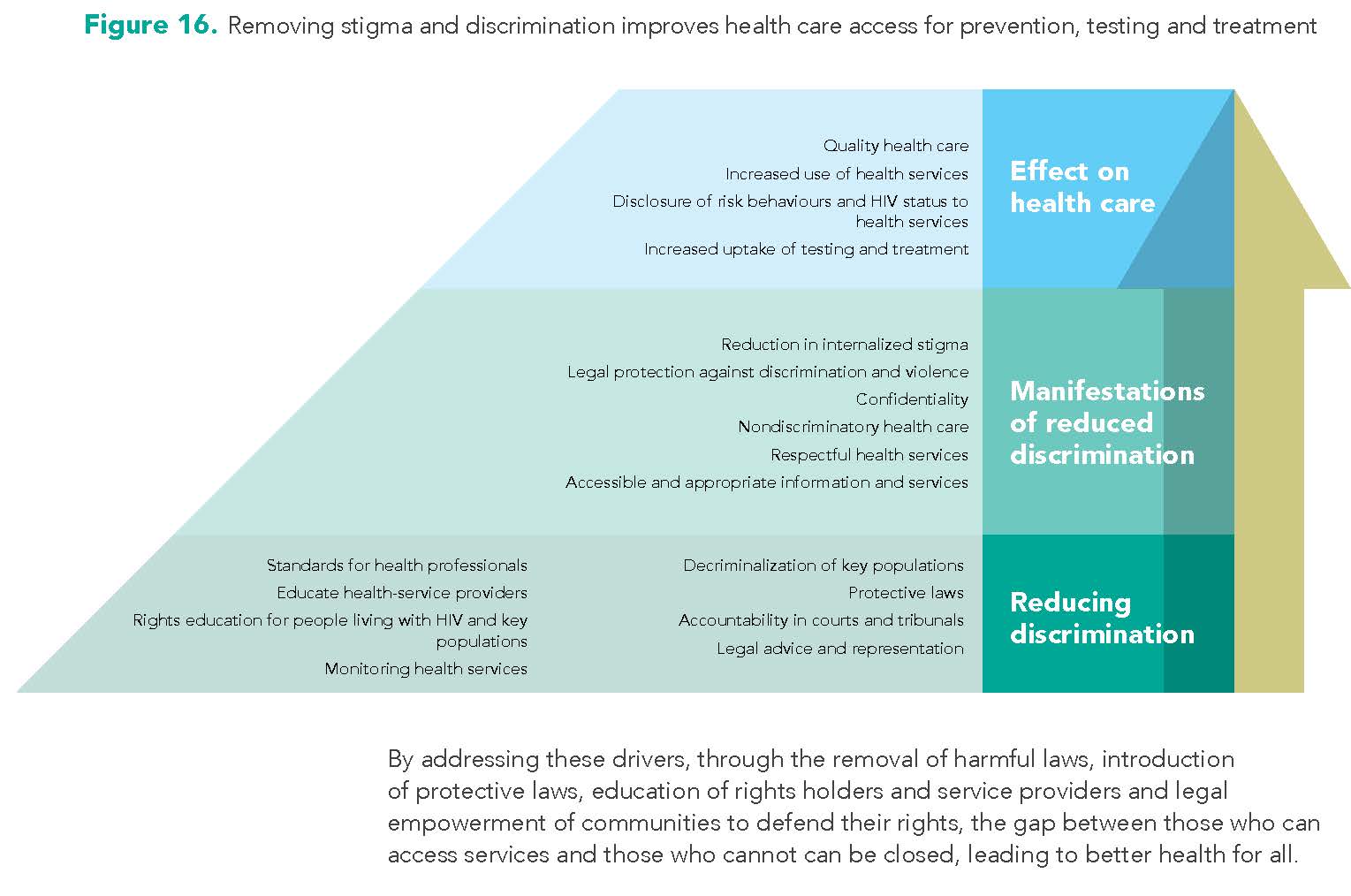


UNAIDS. *Confronting discrimination: Overcoming HIV-related stigma and discrimination in healthcare settings and beyond.* Geneva, Switzerland: UNAIDS Joint United Nations Programme on HIV/AIDS;2017b. <https://www.unaids.org/sites/default/files/media_asset/confronting-discrimination_en.pdf>

UNAIDS. *Confronting discrimination: Overcoming HIV-related stigma and discrimination in healthcare settings and beyond.* Geneva, Switzerland: UNAIDS Joint United Nations Programme on HIV/AIDS;2017c. <https://www.unaids.org/sites/default/files/media_asset/confronting-discrimination_en.pdf>

UNAIDS. *Confronting discrimination: Overcoming HIV-related stigma and discrimination in healthcare settings and beyond.* Geneva, Switzerland: UNAIDS Joint United Nations Programme on HIV/AIDS;2017d. <https://www.unaids.org/sites/default/files/media_asset/confronting-discrimination_en.pdf>

Vyavaharkar M, Moneyham L, Corwin S, Saunders R, Annang L, Tavakoli A. Relationships between stigma, social support, and depression in HIV-infected African American women living in the rural Southeastern United States. *J Assoc Nurses AIDS Care.* 2010;21(2):144-152. <https://www.ncbi.nlm.nih.gov/pubmed/19879778>

HIV related stigma as a mediator between sources of available support and depression.


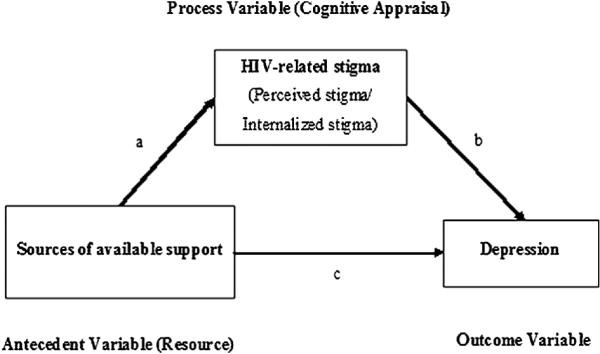


Wardell JD, Shuper PA, Rourke SB, Hendershot CS. Stigma, coping, and alcohol use severity among people living with HIV: A prospective analysis of bidirectional and mediated associations. *Ann Behav Med.* 2018;52(9):762-772. <https://www.ncbi.nlm.nih.gov/pubmed/30124756>

Cross-lagged panel model of the prospective associations among HIV-related stigma, maladaptive coping strategies, and alcohol use severity. Assessments were spaced approximately 12 months apart. All paths from each variable at one wave to each variable at the next wave were included in the model; however, no direct paths between HIV-related stigma and alcohol use severity were statistically significant and so these paths were omitted from the figure for clarity. Standardized parameter estimates are shown with standard errors in parentheses. Moreover, only statistically significant covariance estimates are shown in the figure although all covariances were freely estimated among all variables within each wave. Also, all variables at Waves 2, 3, and 4 were regressed on Wave 1 age and time since HIV diagnosis, and all covariances among age, time since HIV diagnosis, and all Wave 1 variables were estimated in the model, but these covariates are not shown in the model for simplicity. Dashed arrows represent paths that were hypothesized but were not statistically significant. *p < .050; **p < .010.


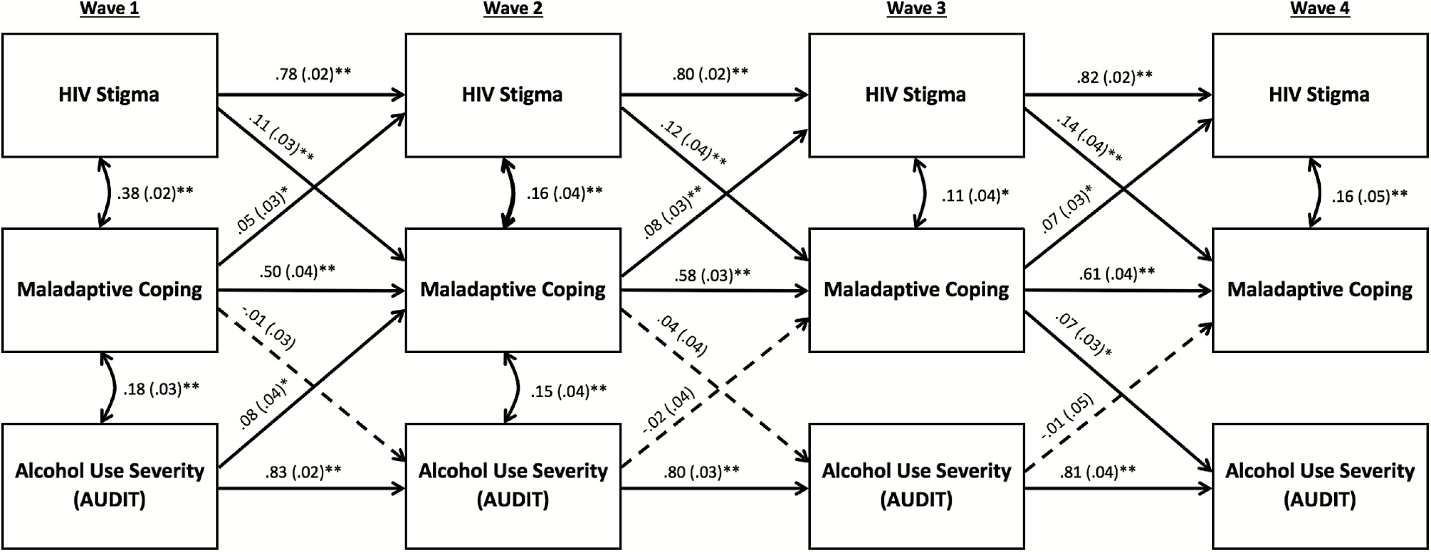


Watt MH, Knettel BA, Knippler ET, et al. The development of Maisha, a video-assisted counseling intervention to address HIV stigma at entry into antenatal care in Tanzania. *Eval Program Plann.* 2020;83:101859. <https://www.ncbi.nlm.nih.gov/pubmed/32795711>

Visual model representing the stigma framework.


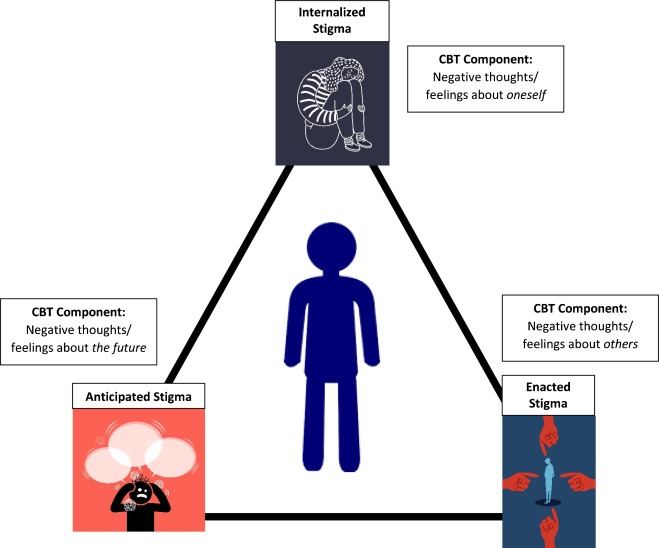


Williams LD. Understanding the relationships among HIV/AIDS-related stigma, health service utilization, and HIV prevalence and incidence in Sub-Saharan Africa: a multi-level theoretical perspective. *Am J Community Psychol.* 2014;53(1-2):146-158. <https://www.ncbi.nlm.nih.gov/pubmed/24477769>

Conceptual model of relationships among constructs of interest in Sub-Saharan African context


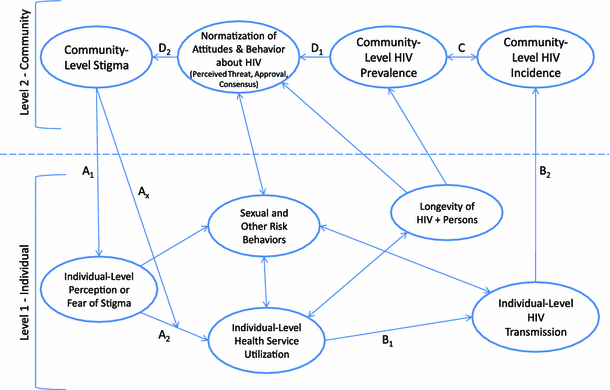


Williams LD, Aber JL. Using a multi-level framework to test empirical relationships among HIV/AIDS-related stigma, health service barriers, and HIV outcomes in KwaZulu‑Natal, South Africa. *AIDS and Behavior.* 2020;24(1):81-94. <https://www.ncbi.nlm.nih.gov/pubmed/30798458>


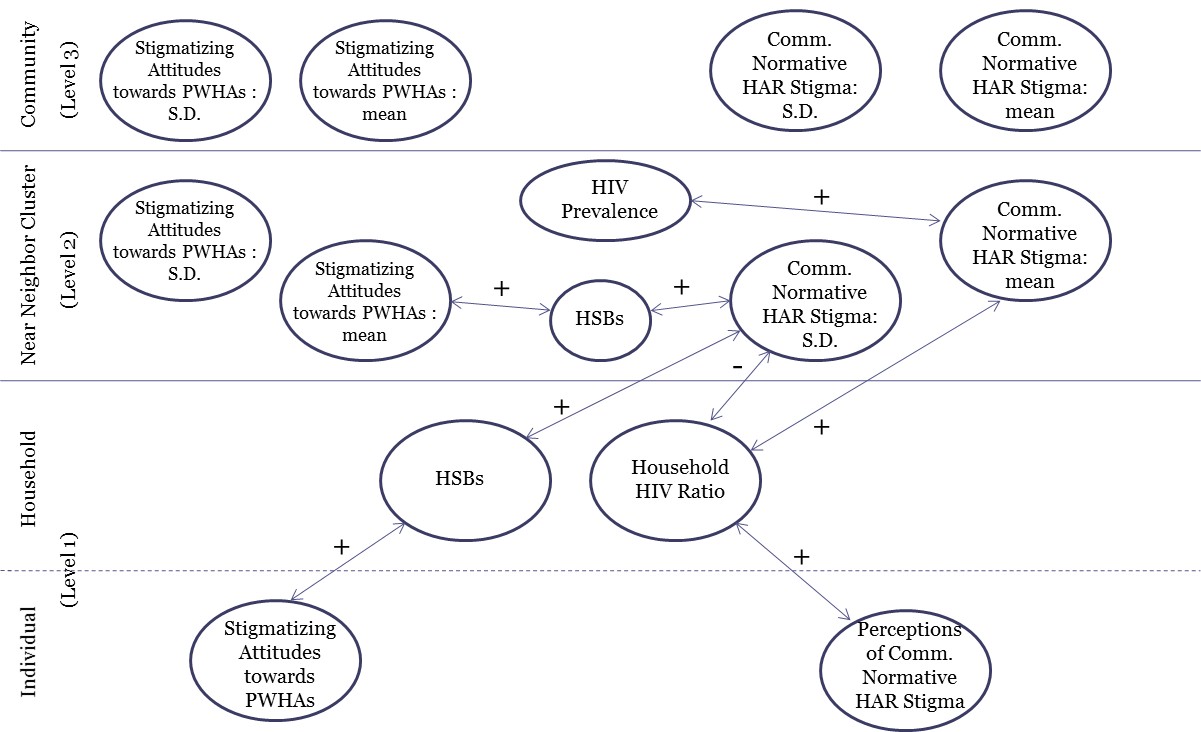


*Model Summarizing all Tested and Significant Relationships among Constructs of*

*Interest from Paper 2.* (Significant relationships are connoted by the presence of a ‘+’ or ‘-’ indicating the direction of the significant relationship.)

Woodgate RL, Zurba M, Tennent P, Cochrane C, Payne M, Mignone J. "People try and label me as someone I'm not": The social ecology of Indigenous people living with HIV, stigma, and discrimination in Manitoba, Canada. *Soc Sci Med.* 2017;194:17-24. <https://www.ncbi.nlm.nih.gov/pubmed/29055805>

The social ecology of stigma and discrimination [S] for Indigenous people living with HIV in Manitoba, Canada.
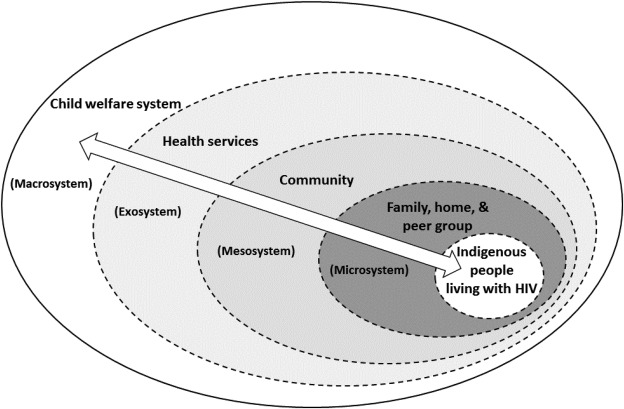


Ecomaps depicting the quality of social-ecological relationships in the lives of Indigenous people who contracted HIV in their youth.


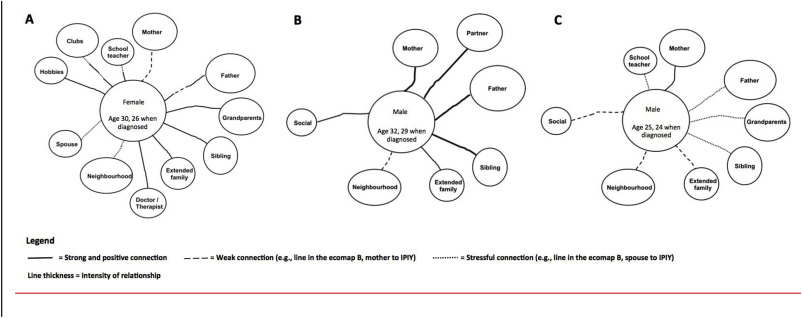


Yang LH, Kleinman A. 'Face' and the embodiment of stigma in China: the cases of schizophrenia and AIDS. *Soc Sci Med.* 2008;67(3):398-408. <https://www.ncbi.nlm.nih.gov/pubmed/18420325>

A stigma model for China.


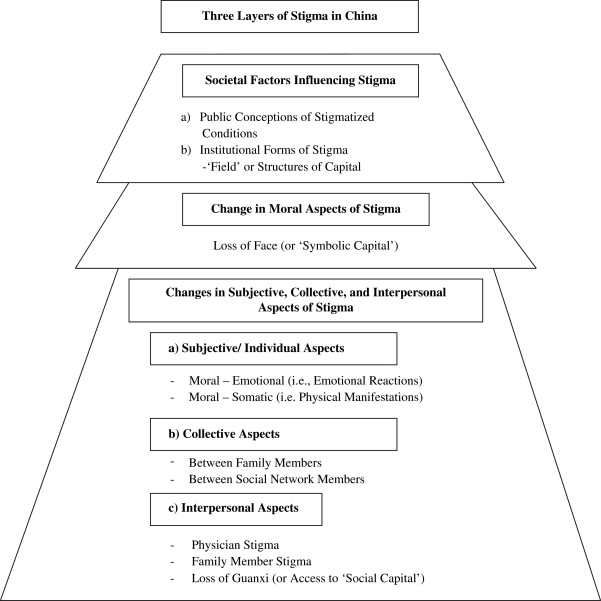


Yu NX, Zhang J, Chan CL. Health Care Neglect, Perceived Discrimination, and Dignity-Related Distress Among Chinese Patients With HIV. *AIDS Educ Prev.* 2016;28(1):90-102. <https://www.ncbi.nlm.nih.gov/pubmed/26829259>


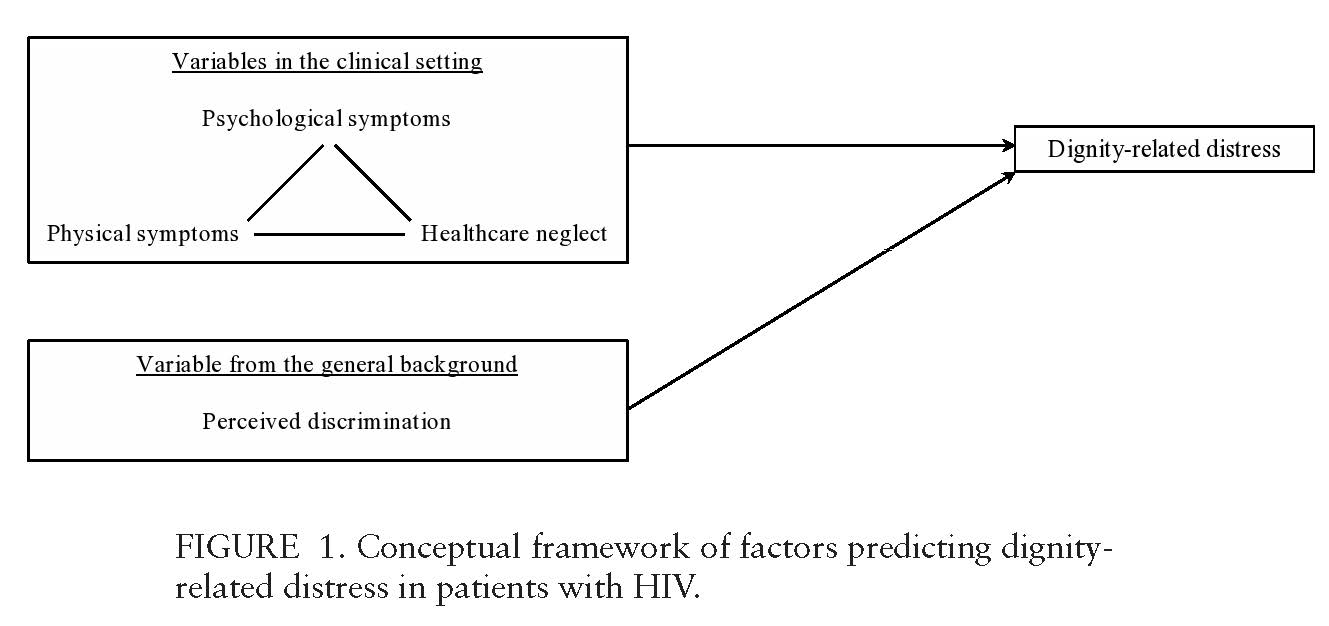


Zang C, Guida J, Sun Y, Liu H. Collectivism culture, HIV stigma and social network support in Anhui, China: a path analytic model. *AIDS Patient Care STDS.* 2014;28(8):452-458. <https://www.ncbi.nlm.nih.gov/pubmed/24853730>


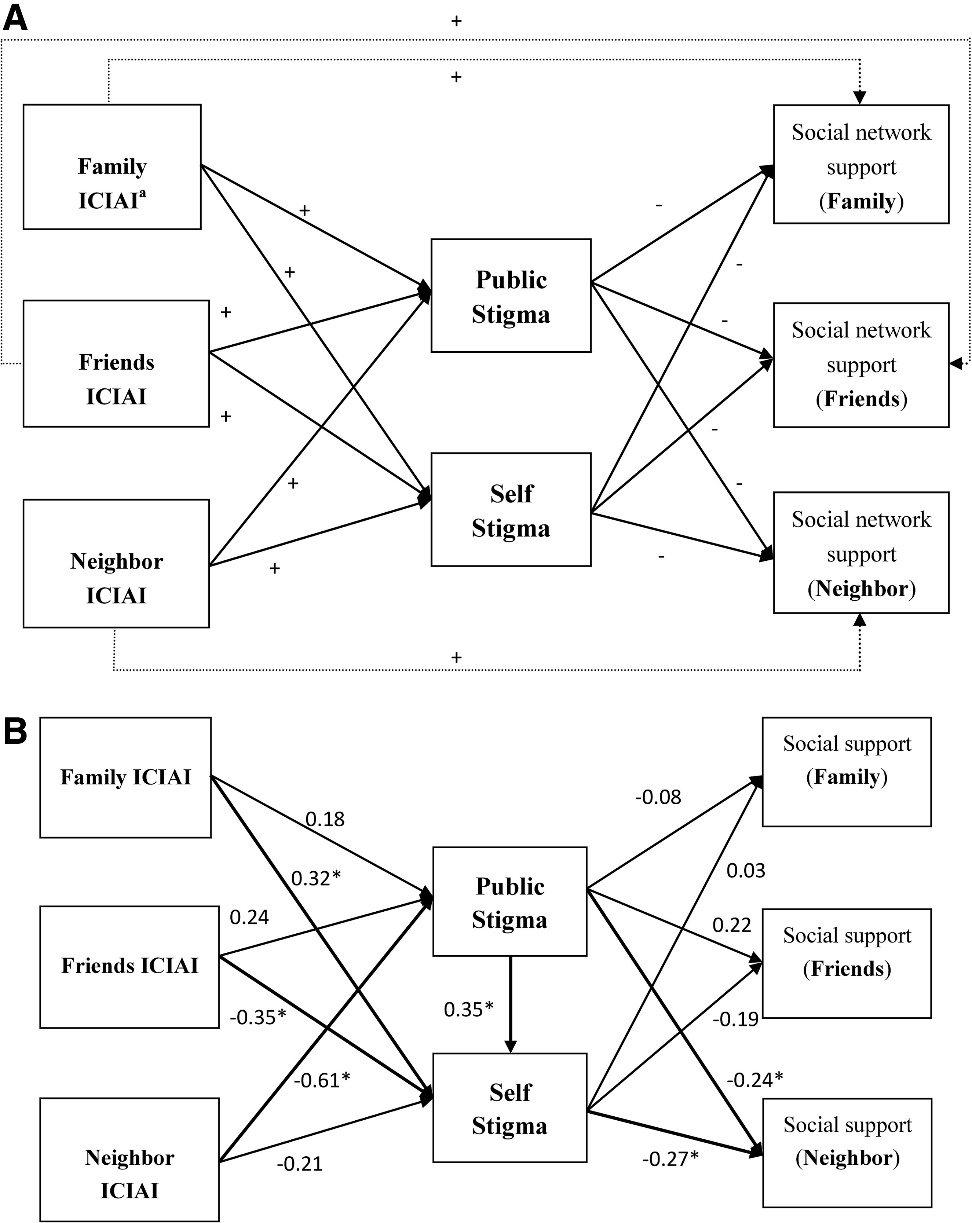


Hypothesized interrelationships among culture, HIV stigma, and social network support. **(B)** Path analysis of interrelationships among culture, HIV stigma, and social network support. **p*≤0.05; ^a^Individualism–Collectivism Interpersonal Assessment Inventory.

**Appendix Table 3. Critical appraisal for measures**

| **Author, year** | **Measure name** | **Internal consistency** | **Reliability (other)** | **Content validity** | **Structural validity** | **Criterion validity** | **Crosscultural validity** | **Responsiveness** | **Interpretability** |
| --- | --- | --- | --- | --- | --- | --- | --- | --- | --- |
| Aggarwal, 2017 [70] | NA | Unclear | Unclear | Unclear | Unclear | Unclear | Low risk of bias | Unclear | Unclear |
| Ahmadi, 2016 [71] | N/A | Low risk of bias | Unclear | Low risk of bias | Unclear | Low risk of bias | Unclear | Unclear | Low risk of bias |
| Biemba, 2019 [72] | NA | Unclear | Unclear | Unclear | Unclear | Unclear | Unclear | Unclear | Unclear |
| Bogart, 2013 [73] | Multiple Discrimination Scale | Low risk of bias | Low risk of bias | Unclear | Unclear | Low risk of bias | Unclear | Unclear | Unclear |
| Brittain, 2017 [74] | Adapted Social Impact Scale | Low risk of bias | Unclear | Unclear | Unclear | Unclear | Low risk of bias | Unclear | Unclear |
| Dos Santos, 2014 [75] | People Living with HIV Stigma Index (adaptation) | Unclear | Unclear | Unclear | Unclear | Unclear | Unclear | Unclear | Unclear |
| Earnshaw, 2013 [14] | HIV Stigma Mechanism Measure | Low risk of bias | Unclear | Low risk of bias | Low risk of bias | Low risk of bias | Low risk of bias | Unclear | Unclear |
| Earnshaw, 2014 [76] | NA | Low risk of bias | Unclear | Unclear | Unclear | Unclear | Unclear | Unclear | Unclear |
| Eaton, 2020 [77] | HIV Microaggressions | Unclear | Unclear | Unclear | Low risk of bias | Unclear | Unclear | Unclear | Low risk of bias |
| Feyissa, 2012 [78] | NA | Low risk of bias | Unclear | Unclear | Unclear | Low risk of bias | Unclear | Unclear | Unclear |
| Franke, 2010 [79] | NA | Low risk of bias | Unclear | Unclear | Low risk of bias | Low risk of bias | Low risk of bias | Unclear | Low risk of bias |
| Franke, 2015 [80] | Opinions about HIV Scale | Low risk of bias | Unclear | Unclear | Unclear | Low risk of bias | Low risk of bias | Unclear | Unclear |
| Friedland, 2020 [81] | People Living with HIV Stigma Index 2.0 | Low risk of bias | Unclear | Low risk of bias | Unclear | Unclear | Low risk of bias | Unclear | Low risk of bias |
| Health Policy Project, 2013 [83] | Measuring HIV Stigma and Discrimination Among Health Facility Staff: Standardized Brief Questionnaire | Unclear | Unclear | Unclear | Unclear | Unclear | Unclear | Unclear | Unclear |
| Hernansaiz-Garrido, 2017 [84] | HIV-Internalized Stigma Scale and the HIV-Disclosure Concerns Scale | Low risk of bias | Low risk of bias | Unclear | Low risk of bias | Low risk of bias | Low risk of bias | Unclear | Low risk of bias |
| Hojilla, 2020 [85] | Abbreviated 10-item Berger HIV stigma scale | Unclear | Unclear | Unclear | Unclear | Unclear | Unclear | Unclear | Unclear |
| Jimenez, 2010 [86] | HIV Felt-Stigma Scale (HFSS) | Low risk of bias | Low risk of bias | Unclear | Low risk of bias | Low risk of bias | Low risk of bias | Low risk of bias | Low risk of bias |
| Kagiura, 2020 [87] | Brief HIV stigma scale for Japanese people living with HIV | Unclear | Unclear | Unclear | Low risk of bias | Low risk of bias | Low risk of bias | Unclear | Low risk of bias |
| Kalichman, 2009 [88] | Internalized AIDS-Related Stigma Scale | Low risk of bias | Unclear | Unclear | Low risk of bias | Low risk of bias | Low risk of bias | Unclear | Unclear |
| Kamitani, 2018 [89] | HIV Stigma Scale for Asians Living with HIV in the United States | Low risk of bias | Unclear | Unclear | Low risk of bias | Low risk of bias | Low risk of bias | Unclear | Unclear |
| Li, 2016 [90] | NA | Low risk of bias | Unclear | Unclear | Low risk of bias | Unclear | Unclear | Unclear | Low risk of bias |
| Molina, 2013 [91] | NA | Low risk of bias | Unclear | Unclear | Low risk of bias | Unclear | Unclear | Unclear | Low risk of bias |
| Neuman, 2013 [92] | NA | Low risk of bias | Unclear | Unclear | Unclear | Unclear | Unclear | Unclear | Unclear |
| Nyblade, 2013 [93] | Health facility questionnaire; Attitudes towards PLHIV | Unclear | Unclear | Unclear | Unclear | Unclear | Unclear | Unclear | Unclear |
| Phillips, 2011 [95] | Internalized Stigma of AIDS Tool (ISAT) | Low risk of bias | Unclear | Low risk of bias | Unclear | Low risk of bias | Unclear | Unclear | Unclear |
| Phillips, 2011 [94] | The Internalized Stigma of AIDS Tool | Unclear | Unclear | Unclear | Unclear | Unclear | Unclear | Unclear | Unclear |
| Pourmarzi, 2015 [96] | Persian Version of HIV/AIDS Related Related Stigma Scale for People Living With HIV/AIDS in Iran | Low risk of bias | Low risk of bias | Low risk of bias | Low risk of bias | Low risk of bias | Low risk of bias | Unclear | Unclear |
| Rao, 2016 [97] | Stigma Scale for Chronic Illness (SSCI) | Low risk of bias | Unclear | Unclear | Low risk of bias | Low risk of bias | Unclear | Unclear | Unclear |
| Reinius, 2017 [98] | Short Version of the HIV Stigma Scale | Low risk of bias | Unclear | Low risk of bias | Low risk of bias | Unclear | Unclear | Unclear | Low risk of bias |
| Rutledge, 2011[99] | HIV/AIDS Provider Stigma Inventory (HAPSI) | Low risk of bias | Unclear | Low risk of bias | Low risk of bias | Low risk of bias | Unclear | Unclear | Unclear |
| Sayles, 2008 [100] | NA | Low risk of bias | Unclear | Unclear | Low risk of bias | Low risk of bias | Unclear | Unclear | Low risk of bias |
| See, 2011 [101] | Professional attitude of health care workers toward serving HIV/AIDS patients and drug users | Low risk of bias | Unclear | Low risk of bias | Low risk of bias | Low risk of bias | Unclear | Unclear | Low risk of bias |
| Smith, 2014 [102] | HIV/AIDS Stigma Scale | Low risk of bias | Unclear | Unclear | Low risk of bias | Unclear | Low risk of bias | Unclear | Unclear |
| Srithanaviboonchai, 2017 [103] | Brief health staff questionnaire | Unclear | Unclear | Unclear | Unclear | Unclear | Unclear | Unclear | Unclear |
| Stangl, 2019 [104] | NA | Low risk of bias | Unclear | Low risk of bias | Low risk of bias | Unclear | Unclear | Unclear | Low risk of bias |
| Stigma Index, 2008 [82] | Stigma Index | Unclear | Unclear | Unclear | Unclear | Unclear | Unclear | Unclear | Unclear |
| Tyer-Viola, 2010 [105] | Pregnant Women with HIV Attitude Scale (PWHAS) | Low risk of bias | Unclear | Low risk of bias | Low risk of bias | Unclear | Unclear | Unclear | Unclear |
| UNAIDS, 2020 [106] | National Commitments and Policy Instrument | Unclear | Unclear | Unclear | Unclear | Unclear | Low risk of bias | Unclear | Unclear |
| Uys, 2009 [107] | HIV/AIDS Stigma Instrument – Nurse (HASI-N) | Low risk of bias | Unclear | Unclear | Unclear | Low risk of bias | Unclear | Unclear | Low risk of bias |
| Varas-Diaz, 2009 [108] | Spanish HIV/AIDS Stigma Scale | Low risk of bias | Unclear | Unclear | Unclear | Low risk of bias | Low risk of bias | Unclear | Unclear |
| Visser, 2008 [109] | Internalized stigma of HIV positive women | Low risk of bias | Unclear | Unclear | Unclear | Low risk of bias | Unclear | Unclear | Low risk of bias |
| Vreeman, 2019 [110] | SAFI (Stigma in AIDS Family Inventory) | Low risk of bias | Low risk of bias | Unclear | Unclear | Unclear | Low risk of bias | Unclear | Unclear |
| Wagner, 2014 [111] | Health Care Provider HIV/AIDS Stigma Scale (HPASS) | Low risk of bias | Low risk of bias | Low risk of bias | Low risk of bias | Low risk of bias | Unclear | Unclear | Unclear |
| Wagner, 2017 [112] | Sexually Transmitted and Blood-Borne Infections Stigma Scale (STBBI) | Low risk of bias | Unclear | Unclear | Low risk of bias | Low risk of bias | Unclear | Unclear | Low risk of bias |
| Wiklander, 2013 [113] | HIV Stigma Scale for Children (HSSC-8) | Low risk of bias | Unclear | Unclear | Low risk of bias | Low risk of bias | Low risk of bias | Unclear | Low risk of bias |
| Windsor, 2013 [114] | Provider Perception Inventory (PPI) | Low risk of bias | Unclear | Unclear | Low risk of bias | Low risk of bias | Unclear | Unclear | Low risk of bias |
| Woldetsadik, 2016 [115] | NA | Low risk of bias | Unclear | Low risk of bias | Unclear | Low risk of bias | Unclear | Unclear | Unclear |
| Wouters, 2017 [116] | Respondents' Stigma Towards HIV (HIVRES); Colleagues’ Stigmatizing Attitudes, Perceptions and Behaviors towards HIV (HIVOES) | Low risk of bias | Unclear | Unclear | Low risk of bias | Low risk of bias | Unclear | Unclear | Unclear |
| Xie, 2019 [117] | Health Care Provider HIV/AIDS Stigma Scale (HPASS) Adaptation | Low risk of bias | Low risk of bias | Low risk of bias | Low risk of bias | Unclear | Low risk of bias | Unclear | Unclear |
| Zelaya, 2012 [118] | NA | Low risk of bias | Unclear | Unclear | Unclear | Low risk of bias | Low risk of bias | Unclear | Low risk of bias |

**Appendix Table 4. Evidence table for measures**

| **ID**  **Framework title and type**  **Domain**  **Link** | **Stigma / discrimination subtype**  **Aim**  **Definition**  **Framework** | **Target population**  **Surveyed participants** | **Scale structure**  **Number of items**  **Answer mode** | **Reliability**  **Validity** |
| --- | --- | --- | --- | --- |
| Sayles, 2008 [100]  **Year published:** 2008  **Domain:** Internalized stigma  **Measure name:** NA  https://www.ncbi.nlm.nih.gov/pubmed/18389363 | **Aim:** To develop and evaluate a multi-dimensional measure of internalized HIV stigma that captures stigma related to treatment and other aspects of the disease among socio-demographically diverse persons living with HIV/AIDS  **Definition:** Internalized stigma = occurs as an individual internalizes cultural norms and narratives that identify him/her as a member of a deviant group, and assumes a ‘‘spoiled identity’’ as described in the work of Goffman  **Stigma subtype addressed:** Internalized  **Underlying framework:** NA | **Target population:** HIV-positive adults  **Surveyed participants:** HIV-positive adults in which women, racial/ethnic minorities, and people with limited income and education were represented  **Framework specific to HIV:** Yes | **Scale structure:** 4 scales: stereotypes, disclosure concerns, social relationships, self-acceptance  **Number of items:** 28 items  **Answer mode:** Rating scale | **Reliability:** Internal consistency reliability was 0.93 for the overall measure, and exceeded 0.85 for three of the four stigma scales  **Validity**: Items discriminated well across scales, and correlations of the scales with shame, social support, and mental health supported construct validity |
| Visser, 2008 [109]  **Year published:** 2008  **Domain:** Internalized stigma  **Measure name:** Internalized stigma of HIV positive women  https://www.ncbi.nlm.nih.gov/pubmed/18266101 | **Aim:** To develop three equivalent stigma scales that could assess the different perspectives of HIV-related stigma (personal attitudes held by individuals; stigma attributed to others and the internalized stigma felt by those living with HIV) and to compare the level of stigma  **Definition:** Stigma = social construction of deviation from an ideal or expectation, contributing to a powerful discrediting social label that reduces the way individuals see themselves and are viewed by others  Internalized stigma = stigma felt by someone living with HIV as a response to stigma in the community  **Stigma subtype addressed:** Personal views of stigma, stigma attributed to others, internalised stigma  **Underlying framework:** NA | **Target population:** HIV positive women  **Surveyed participants:** HIV-infected pregnant women recruited at clinics in Tshwane (South Africa)  **Framework specific to HIV:** Yes | **Scale structure:** 2 factors: blame and judgement, and interpersonal distancing  **Number of items:** 17 items  **Answer mode:** Rating scale | **Reliability:** Internal consistency for Blame and judgement ranged from 0.61 to 0.80 across samples and for Interpersonal distancing from 0.61 to 0.81  **Validity**: For the HIV positive women, those who were more knowledgeable had lower levels of internalised stigma (r = -0.12, P <0.05), but there was no significant relationship between knowledge and the level of stigma they attributed to others; higher levels of internalised stigma were significantly associated with increased levels of depression (r = 0.20, P<0.001), decreased self-esteem (r = -0.16, P<0.01), and decreased perceived social support (r = -0.18, P<0.01) |
| Kalichman, 2009 [88]  Peltzer, 2011 [132]; Geibel, 2020 [123]; Chan, 2019 [120]; Tsai, 2013 [142]  **Year published:** 2009  **Domain:** Internalized stigma  **Measure name:** Internalized AIDS-Related Stigma Scale  https://www.ncbi.nlm.nih.gov/pubmed/19085224 | **Aim:** To adapt 6 items from the community-held AIDS-Related Stigma Scale to reflect internal representations of AIDS-related stigma  **Definition:** NA  **Stigma subtype addressed:** Internalized  **Underlying framework:** Goffman’s (1963) model of social stigma; items were adapted from a theoretically based and psychometrically sound measure of community-held AIDS-related stigmas (Kalichman et al., 2005) | **Target population:** HIV-positive men and women  **Surveyed participants:** HIV-positive men and women in Swaziland, South Africa, and Atlanta (Georgia), India, Uganda  **Framework specific to HIV:** Yes | **Scale structure:** 1 scale  **Number of items:** 6 items  **Answer mode:** Dichotomous scale | **Reliability:** Internally consistent (overall alpha 0.75) and time stable (r 0.53)  **Validity**: Evidence in support of the scale’s convergent, discriminant, and criterion-related validity; Internalized stigma was positively correlated with depression scores in all three countries, inversely associated with social support in all three countries and internalized stigma was correlated with HIV symptoms in Swaziland and not in Cape Town or Atlanta; the 4 items related to self-hatred or shame loaded positively on one of two factors in a factor analysis (Southern India dataset), while the 2 items related to fears of disclosure loaded positively on the second factor; another exploratory factor analysis indicated the presence of a single factor (Uganda dataset) |
| Franke, 2010 [79]  Valle, 2015 [144]  **Year published:** 2010  **Domain:** Internalized stigma  **Measure name:** NA  https://www.ncbi.nlm.nih.gov/pubmed/18841460 | **Aim:** To validate the Spanish version of the Berger HIV Stigma Scale and create a valid and reliable abridged version  **Definition:** NA  **Stigma subtype addressed:** Personalized  **Underlying framework:** Adapted from the Berger HIV Stigma Scale | **Target population:** HIV-infected adults  **Surveyed participants:** Peruvian patients initiating antiretroviral therapy  **Framework specific to HIV:** Yes | **Scale structure:** 4 sub-scales: personalized stigma, disclosure concerns, negative self-image, and concern with public attitudes toward people with HIV  **Number of items:** 21 items  **Answer mode:** Rating scale | **Reliability:** Overall internal reliability of the abridged scale was good (0.84), and subscale Cronbach’s values were comparable to those of the full scale  **Validity**: The abridged version demonstrated moderate negative correlations with quality of life (MOS-HIV summary score) and mental health (MOS-HIV mental health subscore) and positive correlations with depression (HSCL score); the Negative Self-Image subscore from both scales also moderately correlated with depression, quality of life, and mental health scores |
| Jimenez, 2010 [86]  **Year published:** 2010  **Domain:** Internalized stigma  **Measure name:** HIV Felt-Stigma Scale (HFSS)  https://pubmed.ncbi.nlm.nih.gov/20665283/ | **Aim:** To culturally adapt and validate a scale to measure HIV-related felt stigma in a group of people living with HIV/AIDS (PLWHA) in Puerto Rico  **Definition:** NA  **Stigma subtype addressed:** Personalized, felt  **Underlying framework:** Cultural adaptation of Berger’s HSS scale | **Target population:** People living with HIV/AIDS (PLWHA) in Puerto Rico  **Surveyed participants:** People living with HIV/AIDS (PLWHA) in Puerto Rico  **Framework specific to HIV:** Yes | **Scale structure:** 4 dimensions: personalized stigma, disclosure concerns, negative self-image, and concern with public attitudes  **Number of items:** 17 items  **Answer mode:** Rating scale | **Reliability:** Alpha coefficient of the 17-item total-scale score 0.91; test-retest correlations demonstrated that 3 of the 4 dimensions were stable over time, and total-scale scores in the test-retest correlations were similar; the concern with public attitudes dimension did not show a significant correlation  **Validity**: Alpha coefficient of the 17-item total-scale score (0.91) indicates that the scale measures both a single construct and a multidimensional one; the results showed significant correlations between the 4 dimensions and the total scale, even with independent items in every dimension, suggesting it measures a general felt-stigma concept; in general, the HFSS showed stronger correlations with all compared instruments than did the 48-item scale; the HFSS correlated more strongly to instruments that measure the same or associated constructs |
| Phillips, 2011 [95]  **Year published:** 2011  **Domain:** Internalized stigma  **Measure name:** Internalized Stigma of AIDS Tool (ISAT)  https://www.ncbi.nlm.nih.gov/pubmed/21692574 | **Aim:** To develop an instrument to measure internalized stigma in those with HIV/AIDS  **Definition:** Stigma = attribute that is deeply discrediting and a mark that reduces the bearer from “from a whole and usual person to a tainted, discounted one  Internalization of stigma = process in which stigmatized persons accept the negative views that others in society hold about them and incorporates those views into their self-concept  **Stigma subtype addressed:** Internalized  **Underlying framework:** Items were generated from interviews, a panel of experts reviewed, modified, and came to consensus that the items were tapping the domain of internalized stigma of HIV/AIDS | **Target population:** Persons with HIV/AIDS  **Surveyed participants:** HIV-infected women living in the Southeastern United States who were recruited from community-based HIV/AIDS service organizations  **Framework specific to HIV:** Yes | **Scale structure:** 1 scale  **Number of items:** 10 items  **Answer mode:** Rating scale | **Reliability:** Cronbach’s alpha 0.91 (Time 1), 0.92 (Time 2), and 0.92 (Time 3)  **Validity**: Convergent validity was supported with significant positive correlations with the Centers for Epidemiological Studies Depression Scale (CES-D) (rho = 0.33, p < 0.0001) and the Perceived Stigma Scale (PSS) (rho = 0.56, < 0.0001) |
| Phillips, 2011 [94]  **Year published:** 2011  **Domain:** Internalized stigma  **Measure name:** The Internalized Stigma of AIDS Tool  https://www.ncbi.nlm.nih.gov/pubmed/21975476 | **Aim:** To describe the development of an instrument to measure internalized stigma of HIV/ AIDS  **Definition:** Stigma = social construct in which an individual (the stigmatized person who is marked) is devalued or even rejected by others (the stigmatizers who mark) based on some departure from the social norm; an attribute that is deeply discrediting  **Stigma subtype addressed:** NA  **Underlying framework:** Based on the self-concept adaptive mode of the Roy adaptation model | **Target population:** Persons living with HIV/AIDS  **Surveyed participants:** NA  **Framework specific to HIV:** Yes | **Scale structure:** Physical and personal self-components  **Number of items:** 10 items  **Answer mode:** Rating scale | **Reliability:** NA  **Validity**: NA |
| Zelaya, 2012 [118]  **Year published:** 2012  **Domain:** Internalized stigma  **Measure name:** NA  https://www.ncbi.nlm.nih.gov/pubmed/22272891 | **Aim:** To develop and psychometrically test three parallel scales measuring self, experienced, and perceived stigma  **Definition:** Stigma = social process, discrediting and devaluing individuals or groups with an attribute that is either feared or sanctioned by society as immoral or deviant  **Stigma subtype addressed:** Self, experienced, perceived  **Underlying framework:** Three main theoretical domains of HIV/AIDS stigma: self-imposed, perceived, and/or experienced by people living with HIV/AIDS | **Target population:** People living with HIV/AIDS  **Surveyed participants:** People living with HIV/AIDS in India  **Framework specific to HIV:** Yes | **Scale structure:** 3 scales: self, experienced, and perceived stigma  **Number of items:** 22 items  **Answer mode:** Rating scale | **Reliability:** Cronbach’s alpha 0.84 for self stigma, 0.86 for experienced stigma, 0.83 for perceived stigma  **Validity**: External validity was ascertained by confirming a significant positive association between the measure of each type of stigma and depression (measured using CES-D, structural equation modeling) |
| Molina, 2013 [91]  **Year published:** 2013  **Domain:** Internalized stigma  **Measure name:** NA  https://www.ncbi.nlm.nih.gov/pubmed/23668809 | **Aim:** To develop a multidimensional assessment of HIV/AIDS stigma for Latino GBT living with HIV/AIDS, and to test whether such stigma is related to self-esteem, safe sex self-efficacy, social support, and alcohol, and drug use  **Definition:** Stigma = enduring condition, status or attribute that is negatively valued by a society and whose possession consequently discredits and disadvantages an individual  Enacted stigma = may take the form of experiences such as being denied housing because of one’s HIV-positive status  Perceived stigma = stigmatized individual’s awareness of society’s negative views toward his/her group  Internalized stigma = adoption of society’s negative views into the self-concept  **Stigma subtype addressed:** Internalized, perceived, and enacted  **Underlying framework:** Developed from qualitative data and existing research | **Target population:** Latino gay/ bisexual men and transgender women living with HIV/AIDS  **Surveyed participants:** Latino gay/ bisexual men and transgender women living with HIV/AIDS  **Framework specific to HIV:** Yes | **Scale structure:** 4 domains: Internalized, perceived, generalized enacted, romantic and sexual enacted  **Number of items:** 36 items  **Answer mode:** Rating scale | **Reliability:** Internalized alpha 0.88, perceived 0.96, generalized enacted 0.90, romantic and sexual enacted stigma 0.91; all items had item-total correlations equal to 0.42 or higher  **Validity**: The HIV/AIDS stigma dimensions showed adequate convergent validity; parallel dimensions of different stigmata were particularly correlated with one another |
| Wiklander, 2013 [113]  Rydstrom, 2015 [137]  **Year published:** 2013  **Domain:** Internalized stigma  **Measure name:** HIV Stigma Scale for Children (HSSC-8)  https://www.ncbi.nlm.nih.gov/pubmed/24225077 | **Aim:** To test a short version of the 40-item HIV Stigma Scale, adapted for children with HIV infection  **Definition:** Stigma = attribute that is deeply discrediting that reduces the individual from a whole and usual person to a tainted, discounted one  **Stigma subtype addressed:** Disclosure concerns, negative self-image, and concerns with public attitudes  **Underlying framework:** Adapted from Berger HIV Stigma Scale | **Target population:** Children with HIV infection  **Surveyed participants:** Children 8–18 years old with HIV infection in Sweden  **Framework specific to HIV:** Yes | **Scale structure:** 3 dimensions: disclosure concerns, negative self-image, and concerns with public attitudes  **Number of items:** 8 items  **Answer mode:** Rating scale | **Reliability:** Acceptable internal consistency (0.78-0.81) with exception for the disclosure concerns subscale (0.55)  **Validity**: Evidence for internal validity was supported by a principal component analysis suggesting a 3-factor solution with all items loading on the same subscales as in the original HSS-40; evidence for external validity was supported in correlational analyses with measures of HRQoL, where higher levels of stigma correlated with poorer HRQoL |
| Earnshaw, 2014 [76]  **Year published:** 2014  **Domain:** Internalized stigma  **Measure name:** NA  https://www.ncbi.nlm.nih.gov/pubmed/25040218 | **Aim:** This study tests whether HIV self-stigma, or experiences of stigma at the individual level, is associated with engagement in unprotected sex among people living with HIV in KwaZulu-Natal, South Africa  **Definition:** HIV public stigma = social devaluation and discrediting associated with HIV  **Stigma subtype addressed:** Self  **Underlying framework:** Informed by the AIDS-Related Stigma Scale (Kalichman et al., 2005) | **Target population:** People living with HIV  **Surveyed participants:** People living with HIV in KwaZulu-Natal, South Africa  **Framework specific to HIV:** Yes | **Scale structure:** 1 scale  **Number of items:** 7 items  **Answer mode:** Rating scale | **Reliability:** Strong reliability at each assessment (Cronbach’s alpha 0.88-0.91)  **Validity**: NA |
| Pourmarzi, 2015 [96]  **Year published:** 2015  **Domain:** Internalized stigma  **Measure name:** Persian Version of HIV/AIDS Related Related Stigma Scale for People Living With HIV/AIDS in Iran  https://www.ncbi.nlm.nih.gov/pmc/articles/PMC4818378/ | **Aim:** Aimed to assess validity and reliability of the Persian version of HIV/AIDS related stigma scale which was developed by Kang et al for people living with HIV/AIDS in Iran  **Definition:** Stigma = a mark of disgrace and shame associated with specific conditions.  **Stigma subtype addressed:** Perceived  **Underlying framework:** NA | **Target population:** People living with HIV/AIDS in Iran  **Surveyed participants:** People living with HIV/AIDS in Iran  **Framework specific to HIV:** Yes | **Scale structure:** Five subdomains: social rejection, negative self-worth, perceived interpersonal insecurity, financial insecurity, discretionary disclosure 0.83  **Number of items:** 20 items  **Answer mode:** Rating scale | **Reliability:** Cronbach’s alpha for overall scale 0.85, for social rejection 0.84, negative self-worth 0.70, perceived interpersonal insecurity 0.57, financial insecurity, 0.70 discretionary disclosure 0.83; test–retest reliability was ICC = 0.78  **Validity**: Correlation between items and their hypothesized subscale > 0.5; correlation between an item and its own subscale was significantly higher than its correlation with other subscales |
| Rao, 2016 [97]  Rao, 2013 [133]  **Year published:** 2016  **Domain:** Internalized stigma  **Measure name:** Stigma Scale for Chronic Illness (SSCI)  https://pubmed.ncbi.nlm.nih.gov/27761520/ | **Aim:** To describe the psychometric properties of the 14-item SSCI used with a population of African Americans living with HIV.  **Definition:** Public stigma = stigmas held by members of the public, such as healthcare professionals, clergy, or employers, about people with devalued characteristics that result in stereotypes, prejudice and discrimination  Internalized stigma = once public stigmas are enacted (i.e., personally experienced), they can be internalized by the stigmatized individual  **Stigma subtype addressed:** Enacted, internalized  **Underlying framework:** Earnshaw and colleagues (2013) framework of HIV-related stigma | **Target population:** African Americans Living with HIV  **Surveyed participants:** African Americans Living with HIV  **Framework specific to HIV:** Broader | **Scale structure:** 2 scales: internalized and enacted stigma  **Number of items:** 14 items  **Answer mode:** Rating scale | **Reliability:** Overall scale Cronbach’s alpha 0.93 and item-total correlations were equal or greater than 0.42; Cronbach’s alphas for the SSCIEnacted and SSCI-Internalized Stigma scales were 0.93 and 0.84  **Validity**: Excellent concurrent validity, strong positive relationships between the HSS and the SSCI overall (r = .76, df = 62, p <.0001), SSCI-Enacted (r = .69, df = 62, p <.0001), and SSCI-Internalized summary scores (r = .71, df = 62, p <.0001) |
| Brittain, 2017 [74]  **Year published:** 2017  **Domain:** Internalized stigma  **Measure name:** Adapted Social Impact Scale  https://pubmed.ncbi.nlm.nih.gov/27052843/ | **Aim:** To examine factors associated with social support and stigma among pregnant women initiating antiretroviral therapy in the Western Cape, South Africa; and explored associations with depressive symptoms  **Definition:** NA  **Stigma subtype addressed:** Internalized  **Underlying framework:** NA | **Target population:** HIV-infected pregnant women  **Surveyed participants:** HIV-infected pregnant women  **Framework specific to HIV:** Yes | **Scale structure:** Five items included from the original scale and 2 items added assessing HIV-related stigma in the context of motherhood specifically; two domains-social rejection and internalized shame  **Number of items:** 7 items  **Answer mode:** Rating scale | **Reliability:** Social rejection Cronbach's alpha 0.75 and internalized shame Cronbach's alpha 0.80  **Validity**: NA |
| Hernansaiz-Garrido, 2017 [84]  **Year published:** 2017  **Domain:** Internalized stigma  **Measure name:** HIV-Internalized Stigma Scale and the HIV-Disclosure Concerns Scale  https://www.ncbi.nlm.nih.gov/pubmed/26837625 | **Aim:** To develop instruments to measure internalized stigma and disclosure concerns in the Spanish population, and to analyze their reliability and validity and to provide a short version.  **Definition:** HIV stigma = socially constructed and shared knowledge about the devalued status of people living with HIV, who as a result are subject to prejudice, discounting, discrediting, and discrimination  Internalized stigma = represents the devaluation and discredit of oneself based on one’s stigma. It is regarded as a process ‘‘in which stigmatized persons accept the negative views that others in society hold about them and incorporate those views into their self-concept’  **Stigma subtype addressed:** Internalized  **Underlying framework:** NA | **Target population:** People living with HIV in Spanish-speaking countries  **Surveyed participants:** Adults living with HIV in Spanish-speaking countries  **Framework specific to HIV:** Yes | **Scale structure:** 2 scales  **Number of items:** 20 items (10 items each)  **Answer mode:** Rating scale | **Reliability:** Cronbach’s alphas for the HIV-ISS and the HIV-DCS were 0.94 and 0.93; test–retest correlation was ICC .79 (p<.001; 95 % CI .71–.85) for the HIV-ISS, and .86 (p\.001; 95 % CI  .81–.90) for the HIV-DCS  **Validity**: All of the HIV-ISS items loaded on Factor 1 and all items from the HIV-DCS scale loaded on Factor 2, which suggests that Factor 1 represents Internalized Stigma due to HIV condition and Factor 2 refers to Disclosure Concerns; with respect to criterion validity, results showed that the whole scales and the short forms are significantly related to depression, anxiety and self-esteem |
| Reinius, 2017 [98]  Luz, 2020 [130]  **Year published:** 2017  **Domain:** Internalized stigma  **Measure name:** Short Version of the HIV Stigma Scale  https://www.ncbi.nlm.nih.gov/pubmed/28558805 | **Aim:** To develop a substantially shorter, but still valid, version of the HIV Stigma Scale  **Definition:** NA  **Stigma subtype addressed:** Personalized  **Underlying framework:** Adapted from Berger's HIV Stigma Scale | **Target population:** People living with HIV in Sweden  **Surveyed participants:** People living with HIV in Sweden  **Framework specific to HIV:** Yes | **Scale structure:** 4 sub-scales: personalized stigma, disclosure concerns, concerns with public attitudes and negative self-image  **Number of items:** 12 items  **Answer mode:** Rating scale | **Reliability:** Cronbach’s alpha for the subscales were all >0.7  **Validity**: The hypothesized factor structure was replicated in exploratory factor analysis without cross loadings and CFA supported construct validity with high standardised effects (>0.7) of items on the intended scales; the chi square test was statistically significant (p<0.001) but alternate fit measures indicated acceptable fit (comparative fit index: 0.963, Tucker-Lewis index: 0.950 and root mean square error of approximation: 0.071); corrected item-total correlation coefficients were >0.4 for all items, with a variation indicating that the broadness of the concept of stigma had been captured; all but two aspects of HIV-related stigma that the instrument is intended to cover were captured by the selected items in the short version |
| Kamitani, 2018 [89]  **Year published:** 2018  **Domain:** Internalized stigma  **Measure name:** HIV Stigma Scale for Asians Living with HIV in the United States  https://pubmed.ncbi.nlm.nih.gov/29544965/ | **Aim:** To adapt the shortened Berger Stigma Scale to be culturally appropriate for Asians living with HIV in the United States  **Definition:** NA  **Stigma subtype addressed:** Personalized stigma/disclosure, negative self-image, public attitude  **Underlying framework:** Adapted shortened Berger Stigma Scale | **Target population:** Asians Living with HIV in the United States  **Surveyed participants:** Asians Living with HIV in the United States  **Framework specific to HIV:** Yes | **Scale structure:** 3 subscales: personalized stigma/disclosure, negative self-image, public attitude  **Number of items:** 13 items  **Answer mode:** Rating scale | **Reliability:** Overall Cronbach's alpha 0.92  **Validity**: Factor loadings ranged from 0.57 to 0.85 for the Stigma Scale for ALWH; The Stigma Scale for ALWH was negatively correlated with health status and acculturation, but not with education |
| Vreeman, 2019 [110]  **Year published:** 2019  **Domain:** Internalized stigma  **Measure name:** SAFI (Stigma in AIDS Family Inventory)  https://pubmed.ncbi.nlm.nih.gov/31581890/ | **Aim:** To evaluate a culturally adapted, developmentally appropriate measure to assess HIV stigma experienced by children living with HIV and their caregivers in western Kenya  **Definition:** NA  **Stigma subtype addressed:** Perceived, enacted, internalized, courtesy  **Underlying framework:** Conceptual framework using Earnshaw & Chaudoir, 2009 and Stewart et al., 2008 | **Target population:** Children living with HIV and their families  **Surveyed participants:** Child–caregiver dyads  **Framework specific to HIV:** Yes | **Scale structure:** 4 dimensions using subtype of stigma (perceived, enacted, internalized, courtesy), and broken into child stigma items and caregiver items  **Number of items:** 23 items (11 items child stigma, 13 items caregiver stigma)  **Answer mode:** Dichotomous scale,Answer mode varies by item | **Reliability:** Test–retest reliability was high; responses by both children and caregivers on individual stigma items were highly consistent  **Validity**: Construct validity was high on child- and caregiver-reported emotional and behavioral health, with higher levels of reported HIV stigma associated with poorer mental health |
| Eaton, 2020 [77]  **Year published:** 2020  **Domain:** Internalized stigma  **Measure name:** HIV Microaggressions  https://pubmed.ncbi.nlm.nih.gov/31177373/ | **Aim:** To develop items for a measure of HIV-based microaggressions and to explore relationships between the HIV-based microaggression and HIV-related health outcomes  **Definition:** NA  **Stigma subtype addressed:** NA  **Underlying framework:** NA | **Target population:** People living with HIV  **Surveyed participants:** Primarily black men living with HIV  **Framework specific to HIV:** Yes | **Scale structure:** 3 sub-scales: direct microaggressions - negative treatment towards individual living with HIV; self-protection from microaggressions - avoiding circumstances where stigmatizing experiences are assumed to occur; indirect microaggressions - negative treatment towards all people living with HIV  **Number of items:** 13 items  **Answer mode:** Rating scale | **Reliability:** NA  **Validity**: The microaggressions scale demonstrated convergent validity (with internalized, enacted, and anticipated stigmas) and discriminant validity (with social support) |
| Hojilla, 2020 [85]  **Year published:** 2020  **Domain:** Internalized stigma  **Measure name:** Abbreviated 10-item Berger HIV stigma scale  https://www.ncbi.nlm.nih.gov/pubmed/32632497 | **Aim:** To examine the demographic and clinical correlates of HIV stigma and evaluated how HIV stigma was associated with physical and mental health outcomes one year later in a primary-care based cohort of persons living with HIV  **Definition:** HIV stigma = construct that explains negative social attitudes toward persons living with HIV as well as internalization of negative feelings and beliefs experienced by persons living with HIV  **Stigma subtype addressed:** Personalized stigma  **Underlying framework:** NA | **Target population:** Persons living with HIV (PLHIV)  **Surveyed participants:** Persons living with HIV  **Framework specific to HIV:** Yes | **Scale structure:** 4 subscales: personalized stigma, disclosure concerns, negative self-image, and concerns around public attitudes towards persons living with HIV  **Number of items:** 10 items  **Answer mode:** Rating scale | **Reliability:** NA  **Validity**: NA |
| Kagiura, 2020 [87]  **Year published:** 2020  **Domain:** Internalized stigma  **Measure name:** Brief HIV stigma scale for Japanese people living with HIV  https://pubmed.ncbi.nlm.nih.gov/31658827/ | **Aim:** To validate a 10-item HIV stigma scale that was originally developed in the U.S.A. and adapting it for Japanese adults with HIV  **Definition:** NA  **Stigma subtype addressed:** NA  **Underlying framework:** Adapted from Wright’s HIV stigma scale | **Target population:** Japanese adults with HIV  **Surveyed participants:** Japanese adults with HIV  **Framework specific to HIV:** Yes | **Scale structure:** 4 subcategories- personalized stigma, disclosure, negative self-image and public attitudes  **Number of items:** 10 items  **Answer mode:** Rating scale | **Reliability:** Omega values in the total HSSj, and the subcategories of “concern with public stigma” and “negative self-image” were 0.89, 0.83 and 0.87  **Validity**: Correlations with other psychological scales were between 0.34 and 0.51 |
| Bogart, 2013 [73]  **Year published:** 2013  **Domain:** Internalized stigma,Stigma or discrimination in healthcare settings  **Measure name:** Multiple Discrimination Scale  https://www.ncbi.nlm.nih.gov/pubmed/23297084 | **Aim:** To develop a Multiple Discrimination Scale to assess perceived interpersonal, institutional, and violent forms of discrimination due to HIV-serostatus, race/ethnicity, and sexual orientation and to explore health correlates of perceived discrimination due to HIV-status, race/ ethnicity, and sexual orientation  **Definition:** NA  **Stigma subtype addressed:** Interpersonal discrimination, institutional discrimination, violent discrimination  **Underlying framework:** NA | **Target population:** HIV-Positive Black and Latino Men Who Have Sex with Men  **Surveyed participants:** HIV-Positive Black and Latino Men Who Have Sex with Men  **Framework specific to HIV:** Yes | **Scale structure:** 3 subdomains: discrimination events in the past year due to race/ethnicity (MDS-Race), sexual orientation (MDS-Gay), and HIV-serostatus (MDS-HIV)  **Number of items:** 30 items total, 10 per subdomain  **Answer mode:** Dichotomous scale | **Reliability:** Cronbach's alpha >0.80 for all 3 subscales; follow-up scores were used to assess test–retest reliability (> 0.60 for all subscales); the 3 MDS subscales were significantly correlated  **Validity**: All 3 MDS subscales were significantly associated with validated stigma constructs from prior research, showing high convergent validity |
| Earnshaw, 2013 [14]  Earnshaw, 2009 [122]; Misir, 2015 [131]; Goodin, 2018 [125]; Reinius, 2018 [134]  **Year published:** 2013  **Domain:** Internalized stigma,Stigma or discrimination in healthcare settings  **Measure name:** HIV Stigma Mechanism Measure  https://www.ncbi.nlm.nih.gov/pubmed/23456594 | **Aim:** To examine whether HIV stigma mechanisms differentially relate to indicators of affective, behavioral, and physical health and well-being among people living with HIV  **Definition:** HIV stigma = social or structural level phenomenon that exists when labeling, stereotyping, status loss, and discrimination occur within a power structure  Internalized HIV stigma = endorsing negative feelings and beliefs associated with HIV and applying them to the self  Anticipated HIV stigma = expectations of discrimination, stereotyping, and/or prejudice from others in the future due to one’s HIV  Enacted HIV stigma = experiences of discrimination, stereotyping, and/or prejudice from others in the past or present due to one’s HIV  **Stigma subtype addressed:** Internalized, anticipated, enacted  **Underlying framework:** HIV Stigma Framework (Earnshaw & Chaudoir) | **Target population:** People living with HIV  **Surveyed participants:** People living with HIV recruited from an inner-city clinic in the Bronx, NY  **Framework specific to HIV:** Yes | **Scale structure:** 3 subdomains: internalized, anticipated, enacted  **Number of items:** 24 items total, 6 internalized, 9 anticipated, 9 enacted  **Answer mode:** Rating scale | **Reliability:** Cronbach's alpha internalized HIV stigma 0.89, anticipated HIV stigma 0.87, enacted HIV stigma 0.87  **Validity**: The 3 scales were considered to be distinct with the majority of variability in each scale non-overlapping; internalized HIV stigma was uniquely associated with indicators of poorer affective health and wellbeing, including greater helplessness, lower acceptance, and lower perceived benefits of having HIV |
| Neuman, 2013 [92]  **Year published:** 2013  **Domain:** Internalized stigma,Stigma or discrimination in healthcare settings  **Measure name:** NA  https://www.ncbi.nlm.nih.gov/pubmed/23479002 | **Aim:** This study examined experiences of interpersonal discrimination, internalized stigma, and discrimination at health care facilities among HIV-positive adults aged 18 years and older utilizing health facilities in four countries in SubSaharan Africa.  **Definition:** NA  **Stigma subtype addressed:** Internalized stigma, interpersonal discrimination, discrimination experienced in health care facilities  **Underlying framework:** NA | **Target population:** HIV-positive adults  **Surveyed participants:** HIV-positive adults  **Framework specific to HIV:** Yes | **Scale structure:** 3 scales- interpersonal discrimination; discrimination experienced in health care facilities; internalized stigma  **Number of items:** 19 total  **Answer mode:** Dichotomous scale | **Reliability:** Cronbach's alpha scores for both the interpersonal discrimination and health care discrimination measures were >0.8, the score for the internalized stigma score was 0.68  **Validity**: NA |
| Li, 2016[90]  **Year published:** 2016  **Domain:** Internalized stigma,Stigma or discrimination in healthcare settings  **Measure name:** NA  https://www.ncbi.nlm.nih.gov/pubmed/27877022 | **Aim:** To examine, validate, and adapt measuring scales of internalized, personal, and occupational stigma developed in Africa into a Chinese context  **Definition:** HIV/AIDS stigma = discrimination and violation of human rights as “a mark of disgrace"  Internalized stigma = degree to which people living with HIV/AIDS endorse the negative beliefs and feelings associated with HIV/AIDS about themselves  Prejudice = negative emotions/feelings toward HIV-infected people  Discrimination = prejudiced behavioral expressions to people living with HIV/AIDS  Stereotyping = group-based beliefs about people living with HIV/AIDS  **Stigma subtype addressed:** Internalized, personal, occupational  **Underlying framework:** Earnshaw & Chaudoir, 2009; Visser et al., 2008; Stein & Li, 2008 | **Target population:** HIV-positive patients  **Surveyed participants:** HIV-positive patients, non-HIV patients, and health care providers  **Framework specific to HIV:** Yes | **Scale structure:** 3 scales; Internalized (HIV + patients) and personal stigma (non-HIV+ patients) scale factors: guilt/blaming and being refused/refusing service; occupational stigma scale factors: blaming, professionalism, and egalitarianism  **Number of items:** 31 items across 3 scales  **Answer mode:** Rating scale | **Reliability:** Internalized and personal stigma scales with reliability coefficients of 0.869 and 0.853; the occupational stigma scale had a 3-factor structure with a reliability coefficient of 0.839  **Validity**: Confirmatory factor analysis confirmed that the factors identified from the development samples fit the validation sample; however, all P-values from the chi-squared goodness-of-fit tests were p <0.001; among the 3 study groups, each of the subscales associated with measures of sample characteristics further validated the independence of each factor reflecting that they are representative of an independent sub-stigma mechanism |
| Stangl, 2019 [104]  **Year published:** 2019  **Domain:** Internalized stigma,Stigma or discrimination in healthcare settings  **Measure name:** NA  https://pubmed.ncbi.nlm.nih.gov/31840400/ | **Aim:** To develop a succinct set of measures to capture key domains of stigma for use in research on HIV prevention technologies  **Definition:** NA  **Stigma subtype addressed:** Internalized, experienced, perceived  **Underlying framework:** Health Stigma and Discrimination Framework (Stangl et al., 2019) | **Target population:** People living with HIV  **Surveyed participants:** People living with HIV, community members, healthcare workers  **Framework specific to HIV:** Yes | **Scale structure:** 7 scales and 2 experience measures (Fear and judgement, Internalized stigma, Perceived stigma in community, Experienced stigma in the community, Perceived stigma in the healthcare setting, Perceived co-worker stigma, Experienced stigma in healthcare settings)  **Number of items:** 35 questions in total  **Answer mode:** Rating scale,Dichotomous scale | **Reliability:** Acceptable to very good internal consistency (Cronbach’s alpha was <0.6 for the fear and judgement domain and the perceived stigma in the community domain)  **Validity**: Subgroup factor analysis confirmed acceptable reliability for all 3 scales by country, sex and type of health worker |
| Stigma Index, 2008 [82]  Chinouya, 2017 [121]; UNAIDS, 2011 [129]  **Year published:** 2008  **Domain:** Internalized stigma,Stigma or discrimination in healthcare settings,Stigma or discrimination in law  **Measure name:** Stigma Index  https://www.stigmaindex.org/ | **Aim:** Documents how people have experienced HIV-related stigma, and how they have been able to challenge and overcome stigma and discrimination relating to HIV.  **Definition:** NA  **Stigma subtype addressed:** Experiences of stigma and discrimination and their causes; Access to work and services; Internal stigma; Rights, laws and policies; Effecting change; HIV testing; Disclosure and confidentiality; Treatment; Having children; Problems and challenges for people living with HIV  **Underlying framework:** NA | **Target population:** People living with HIV  **Surveyed participants:** Varies  **Framework specific to HIV:** Yes | **Scale structure:** 10 areas: Experiences of stigma and discrimination and their causes; Access to work and services; Internal stigma; Rights, laws and policies; Effecting change; HIV testing; Disclosure and confidentiality; Treatment; Having children; Problems and challenges for people living with HIV  **Number of items:** NA  **Answer mode:** Answer mode varies by item | **Reliability:** NA  **Validity**: NA |
| Dos Santos, 2014 [75]  **Year published:** 2014  **Domain:** Internalized stigma,Stigma or discrimination in healthcare settings,Stigma or discrimination in law  **Measure name:** People Living with HIV Stigma Index (adaptation)  https://www.ncbi.nlm.nih.gov/pubmed/24461042 | **Aim:** To assess current and emerging HIV/AIDS stigma and discrimination trends in South Africa as experienced by people living with HIV/AIDS  **Definition:** Stigma = mark of disgrace associated with a particular circumstance, quality or person  **Stigma subtype addressed:** Internalized  **Underlying framework:** NA | **Target population:** People living with HIV/AIDS  **Surveyed participants:** People living with HIV/AIDS in South Africa  **Framework specific to HIV:** Yes | **Scale structure:** 3 subdomains covering perceptions of self and internal stigma and examples of stigma or discrimination in different settings such as the home, community, workplace, religious or healthcare settings; small adaptations were made to the Index, including the quantifying of all qualitative responses into nominal and ordinal scales and the inclusion of South Africa’s best-known national law and policy guidelines as per the Index directives  **Number of items:** NA  **Answer mode:** Rating scale,Dichotomous scale,Answer mode varies by item | **Reliability:** NA  **Validity**: NA |
| Biemba, 2019 [72]  **Year published:** 2019  **Domain:** Internalized stigma,Stigma or discrimination in healthcare settings,Stigma or discrimination in law  **Measure name:** NA  https://www.ncbi.nlm.nih.gov/pubmed/32257070 | **Aim:** To report on HIV related stigma based on results from an HIV legal environment assessment within the Churches Health Association of Zambia HIV/AIDS program  **Definition:** HIV related stigma = a process of devaluation of people either living with or associated with HIV and AIDS  Perceived stigma = refers to felt or imagined devaluation from individuals and/or institutions  Enacted stigma = refers to actual occurrences of discrimination  Internalized stigma = self-shaming or self-blaming narrative that is adopted by a stigmatized individual  **Stigma subtype addressed:** Internalized, perceived, enacted  **Underlying framework:** NA | **Target population:** People living with HIV  **Surveyed participants:** People living with HIV and health workers  **Framework specific to HIV:** Yes | **Scale structure:** Both quantitative and qualitative questions, narrower and modified form of legal environment assessment; questions separate for people living with HIV and health workers  **Number of items:** NR  **Answer mode:** Rating scale,Dichotomous scale,Free text,Answer mode varies by item | **Reliability:** NA  **Validity**: NA |
| Friedland, 2020 [81]  HIV Stigma Index 2.0[124]  **Year published:** 2020  **Domain:** Internalized stigma,Stigma or discrimination in healthcare settings,Stigma or discrimination in law  **Measure name:** People Living with HIV Stigma Index 2.0  https://pubmed.ncbi.nlm.nih.gov/32881790/ | **Aim:** To describe the process of updating the People Living with HIV Stigma Index to reflect current global treatment guidelines and to better measure intersecting stigmas and resilience  **Definition:** NA  **Stigma subtype addressed:** Internalized  **Underlying framework:** Adapted from the original People Living with HIV (PLHIV) Stigma Index | **Target population:** People living with HIV  **Surveyed participants:** People living with HIV at least 18 years old who had known their status for at least 1 year  **Framework specific to HIV:** Yes | **Scale structure:** Sections consist of: disclosure, your experience of stigma and discrimination, internalized stigma and resilience, interactions with healthcare services, human rights and effecting change, stigma and discrimination experienced for reasons other than your HIV status, personal experience related to stigma / discrimination  **Number of items:** 6 items  **Answer mode:** Rating scale,Free text,Answer mode varies by item | **Reliability:** Good internal consistency (Cronbach’s alphas for Cameroon, Senegal, and Uganda were 0.70, 0.65, 0.75)  **Validity**: Cognitive interview respondents indicated that most questions were well understood and focus group participants said that the Stigma Index 2.0 addressed issues that were relevant to their lives, good construct validity |
| Uys, 2009 [107]  Rosenburg, 2012 [135]; Franke, 2015 [80]  **Year published:** 2009  **Domain:** Stigma or discrimination in healthcare settings  **Measure name:** HIV/AIDS Stigma Instrument – Nurse (HASI-N)  https://www.ncbi.nlm.nih.gov/pubmed/19229683 | **Aim:** To develop and validate a linguistically and culturally appropriate measure of perceived HIV/AIDS stigma for nurses in five African countries  **Definition:** Stigma = significantly discrediting attribute  **Stigma subtype addressed:** NA  **Underlying framework:** Based on a process model of stigma derived from qualitative data | **Target population:** People living with HIV/AIDS  **Surveyed participants:** Nurses in five countries  **Framework specific to HIV:** Yes | **Scale structure:** 2 factors: nurses stigmatizing patients, and nurses being stigmatized  **Number of items:** 19 items  **Answer mode:** Rating scale | **Reliability:** Cronbach alpha 0.90  **Validity**: Concurrent validity (significant negative correlation between stigma and job satisfaction) |
| Varas-Diaz, 2009 [108]  **Year published:** 2009  **Domain:** Stigma or discrimination in healthcare settings  **Measure name:** Spanish HIV/AIDS Stigma Scale  https://www.ncbi.nlm.nih.gov/pubmed/20024702 | **Aim:** To develop and test the psychometric properties of a culturally appropriate HIV/AIDS Stigma Scale for Puerto Rican health care providers and to develop a reduced form of the scale suitable for use in time-limited clinical settings  **Definition:** NA  **Stigma subtype addressed:** NA  **Underlying framework:** Based on previous qualitative evidence gathered from Puerto Rican health professionals | **Target population:** People living with HIV/AIDS  **Surveyed participants:** Puerto Rican health care providers in training  **Framework specific to HIV:** Yes | **Scale structure:** 11 dimensions: fear of infection, emotions associated with HIV/AIDS, closeness to death, people living with HIV/AIDS as vectors of infection, lack of productivity of people living with HIV/AIDS, personal characteristics of people living with HIV/AIDS that foster infection, need to control people living with HIV/AIDS, rights of people living with HIV/AIDS, body signs of HIV/AIDS, responsibility over infection, and people living with HIV/AIDS as obliged to reveal serostatus  **Number of items:** 62 items  **Answer mode:** Rating scale | **Reliability:** Most subscales had adequate, good, or excellent reliabilities, though the factors tapping perceived responsibility for HIV/AIDS infections and closeness to death had modest reliability values; the structural concerns factor exhibited unsatisfactory reliability (0.43) in the original instrument and was dropped  **Validity**: HIV/AIDS stigma was positively correlated with homophobia (r=0.54) and drug user stigma (r=0.38); HIV/AIDS stigma was not associated with having recently obtained an HIV test (r=0.01); taken collectively, these results provide preliminary evidence that the Spanish HIV/ AIDS Stigma Scale has satisfactory convergent and divergent validity. |
| Tyer-Viola, 2010 [105]  **Year published:** 2010  **Domain:** Stigma or discrimination in healthcare settings  **Measure name:** Pregnant Women with HIV Attitude Scale (PWHAS)  https://www.ncbi.nlm.nih.gov/pubmed/20557397 | **Aim:** To carry out initial development and psychometric evaluation of the Pregnant Women with HIV Attitudes Scale  **Definition:** NA  **Stigma subtype addressed:** NA  **Underlying framework:** Attitude Representation Theory; the Attitudes about People with HIV Scale and the Attitudes toward Women with HIV Scale, were combined to create an initial item pool | **Target population:** Pregnant Women with HIV  **Surveyed participants:** Obstetric nurses attending a national conference in the United States of America  **Framework specific to HIV:** Yes | **Scale structure:** 2 components: mothering-choice and sympathy-rights  **Number of items:** 27 items  **Answer mode:** Rating scale | **Reliability:** Alpha 0.89  **Validity**: Principal component analysis yielded a 2-component structure that accounted for 45% of the total variance: Mothering-Choice (alpha estimates 0.89) and Sympathy-Rights (alpha estimates 0.72) |
| Rutledge, 2011 [99]  **Year published:** 2011  **Domain:** Stigma or discrimination in healthcare settings  **Measure name:** HIV/AIDS Provider Stigma Inventory (HAPSI)  https://www.ncbi.nlm.nih.gov/pubmed/21967495 | **Aim:** To describe the conceptual development and initial validation of a suite of measures known collectively as the HIV/AIDS Provider Stigma Inventory  **Definition:** NA  **Stigma subtype addressed:** Instrumental, symbolic, courtesy, and enacted  **Underlying framework:** Grounded dually in Link and Phelan’s social psychological stigma framework and the Awareness, Acceptance, and Action Model (based in principles of mindfulness) | **Target population:** People living with HIV/AIDS  **Surveyed participants:** Undergraduate third-year baccalaureate nursing students  **Framework specific to HIV:** Yes | **Scale structure:** 3 sub-scales: awareness, acceptance, and action  **Number of items:** 81 items  **Answer mode:** Rating scale | **Reliability:** Coefficients a for 16 of 19 resulting measures ranged from 0.80 to 0.98  **Validity**: Content validity was generally supported by expert panelists, and psychometric coefficients revealed strong evidence of reliability and confirmation of hypothesized factor structure |
| See, 2011 [101]  **Year published:** 2011  **Domain:** Stigma or discrimination in healthcare settings  **Measure name:** Professional attitude of health care workers toward serving HIV/AIDS patients and drug users  https://www.ncbi.nlm.nih.gov/pubmed/22022851 | **Aim:** To design a questionnaire and test its reliability and validity to facilitate future studies of professional attitudes among this population (toward serving HIV/ AIDS patients and drug users)  **Definition:** NA  **Stigma subtype addressed:** NA  **Underlying framework:** NA | **Target population:** HIV/AIDS patients and drug users  **Surveyed participants:** Health care workers in Taiwan (public health workers, physicians/registered nurses, and pharmacists)  **Framework specific to HIV:** Broader | **Scale structure:** 4 constructs: discrimination, acceptance of HIV/AIDS patients, acceptance of drug users, and fear  **Number of items:** 16 items  **Answer mode:** Rating scale | **Reliability:** Cronbach’s alpha for the four constructs of >0.7  **Validity**: The average content validity index was 85.6% based on 10 experts, healthcare workers who received training or education on harm reduction or AIDS prevention had higher scores for acceptance of HIV/AIDS patients, acceptance of drug users, and fear, as compared with those who did not receive the training |
| Feyissa, 2012 [78]  **Year published:** 2012  **Domain:** Stigma or discrimination in healthcare settings  **Measure name:** NA  https://www.ncbi.nlm.nih.gov/pubmed/22536080 | **Aim:** To validate items designed to measure HIV/AIDS-related stigma and discrimination among health care providers in a resource-poor setting  **Definition:** NA  **Stigma subtype addressed:** NA  **Underlying framework:** NA | **Target population:** People living with HIV  **Surveyed participants:** Health care providers in Ethiopia  **Framework specific to HIV:** Yes | **Scale structure:** 7 factors across 4 dimensions: fear, value-driven, discrimination, and disclosure  **Number of items:** NA  **Answer mode:** Rating scale | **Reliability:** Cronbach’s alphas of the scales ranged from 0.80 to 0.95  **Validity**: An in-depth knowledge of HIV, perceptions of institutional support, attendance of training on topics related to stigma/discrimination, degree or higher education levels, high HIV case loads, the availability of ART in the health care facility and claiming oneself as nonreligious were all negatively associated with stigma/discrimination, as measured by the 7 newly identified latent factors |
| Nyblade, 2013 [93]  Jain, 2015 [128]; Carr, 2015 [119]  **Year published:** 2013  **Domain:** Stigma or discrimination in healthcare settings  **Measure name:** Health facility questionnaire; Attitudes towards PLHIV  https://www.ncbi.nlm.nih.gov/pubmed/24242266 | **Aim:** To describe a multi-year process to develop a brief questionnaire to measure HIV stigma among health facility staff  **Definition:** NA  **Stigma subtype addressed:** Enacted  **Underlying framework:** Experts participated in a content-development workshop to review an item pool of existing measures, identify gaps and prioritize questions | **Target population:** People living with HIV  **Surveyed participants:** Clinical and non-clinical staff  **Framework specific to HIV:** Yes | **Scale structure:** Health facility questionnaire: 3 domains: actionable drivers of stigma within health facilities, health facility environment, enacted stigma  **Number of items:** Health facility questionnaire: 18 items  Attitudes towards people living with HIV: 5 items  **Answer mode:** Rating scale,Answer mode varies by item,Unclear : Mostly continuous ratings | **Reliability:** NA  **Validity**: Attitudes towards people living with HIV (5-item scale, factor loading alpha 0.78) |
| Windsor, 2013 [114]  **Year published:** 2013  **Domain:** Stigma or discrimination in healthcare settings  **Measure name:** Provider Perception Inventory (PPI)  https://www.ncbi.nlm.nih.gov/pubmed/23082899 | **Aim:** To develop a novel measure of service providers’ stigma about HIV, substance abuse, and MSM behavior  **Definition:** NA  **Stigma subtype addressed:** NA  **Underlying framework:** NA | **Target population:** Nongay identified men who have sex with men and women  **Surveyed participants:** HIV and substance abuse service providers  **Framework specific to HIV:** Broader | **Scale structure:** 2 dimensions: individual attitudes and agency environment  **Number of items:** 39 items  **Answer mode:** Rating scale | **Reliability:** Overall Cronbach's alpha 0.87  **Validity**: Structural equation modeling analysis supported the scale’s predictive validity |
| Smith, 2014 [102]  **Year published:** 2014  **Domain:** Stigma or discrimination in healthcare settings  **Measure name:** HIV/AIDS Stigma Scale  https://www.ncbi.nlm.nih.gov/pubmed/24347146 | **Aim:** To assess a theoretically and culturally informed multidimensional, HIV/AIDS-related stigma scale for the context of health care facilities, families of people living with HIV and  AIDS, and people living with HIV and AIDS  **Definition:** NA  **Stigma subtype addressed:** NA  **Underlying framework:** NA | **Target population:** People living with HIV and AIDS; families of people living with HIV and AIDS in South Africa  **Surveyed participants:** Family/community members, health care workers, and people living with HIV and AIDS living in South Africa  **Framework specific to HIV:** Yes | **Scale structure:** 3 sub-scales: Government Support, Shame and Rejection, and Individual Support  **Number of items:** 12 items  **Answer mode:** Rating scale | **Reliability:** Individual subscales were internally consistent with alpha coefficients ranging from 0.67 to 0.80  **Validity**: Fit indices suggest that the model fit is acceptable and supports the validity of the 3-factor model |
| Wagner, 2014 [111]  **Year published:** 2014  **Domain:** Stigma or discrimination in healthcare settings  **Measure name:** Health Care Provider HIV/AIDS Stigma Scale (HPASS)  https://www.ncbi.nlm.nih.gov/pubmed/24965675 | **Aim:** To describe the development and initial validation of a contextually appropriate HIV stigma scale for health care providers in North America  **Definition:** HIV stigma = prejudice, discounting, discrediting and discrimination directed at people perceived to have AIDS or HIV, their loved ones and associates and the groups and communities with which they are affiliated  **Stigma subtype addressed:** NA  **Underlying framework:** A ground-up qualitative approach was used to develop the scale; the scale supports a tripartite model of HIV stigma consisting of stereotyping, prejudice and discrimination | **Target population:** People living with HIV  **Surveyed participants:** Medical and nursing students from across Canada  **Framework specific to HIV:** Yes | **Scale structure:** 3 scales: prejudice, stereotyping, and discrimination  **Number of items:** 30 items  **Answer mode:** Rating scale | **Reliability:** Total scale Cronbach’s alpha 0.940, Prejudice subscale 0.913, Stereotypes subscale 0.871, Discrimination subscale 0.917; in the test–retest analysis (assessed with the original 47 scale items and with analyses conducted on the final 30 items), the time two measure of internal consistency reliability for the total scale score was Cronbach’s alpha 0.960  **Validity**: 3-factor model determined by exploratory factor analysis and confirmed by CFA; correlation and regression analyses supported convergent validity of the HPASS with a measure of AIDS-related stigma (the AAS Avoidance subscale), AAS Empathy and MC-SF measures demonstrated divergent validity |
| Franke, 2015 [80]  **Year published:** 2015  **Domain:** Stigma or discrimination in healthcare settings  **Measure name:** Opinions about HIV Scale  https://www.ncbi.nlm.nih.gov/pubmed/25294853 | **Aim:** To report the psychometric properties of 2 Spanish language scales (Opinions about HIV Scale; Adapted HIV/AIDS Stigma Instrument–Nurse)  **Definition:** NA  **Stigma subtype addressed:** Enacted  **Underlying framework:** Existing questionnaires and questions that aligned with 3 domains (empathy, avoidance, and policy); HASI-N was adapted | **Target population:** Health care providers and professionals  **Surveyed participants:** Diverse group of health professionals, community health workers, and HIV treatment supporters (a relative or a friend identified by the patient to support them in treatment adherence as a programmatic requisite to starting ART)  **Framework specific to HIV:** Yes | **Scale structure:** 3 components: policy, avoidance, and empathy  **Number of items:** 9 items  **Answer mode:** Rating scale | **Reliability:** Cronbach alpha 0.72, shorter subscales of avoidance, empathy, and policy 0.60, 0.69, and 0.63  **Validity**: Education level was a consistent correlate of stigmatizing opinions and of the reported acts of stigmatizing behavior by other health workers |
| Health Policy Project, 2013 [83]  **Year published:** 2015  **Domain:** Stigma or discrimination in healthcare settings  **Measure name:** Measuring HIV Stigma and Discrimination Among Health Facility Staff: Standardized Brief Questionnaire  https://www.healthpolicyproject.com/index.cfm?ID=publications&get=pubID&pubID=49 | **Aim:** This tool can help facilitate routine monitoring of HIV-related stigma, as well as the expansion and improvement of programming and policies at the health-facility level  **Definition:** NA  **Stigma subtype addressed:** NA  **Underlying framework:** NA | **Target population:** People living with HIV  **Surveyed participants:** Health facility staff  **Framework specific to HIV:** Yes | **Scale structure:** 5 sections (background information, infection control, health facility environment, health facility policies, and opinions about people with HIV), and 1 module (antenatal care, prevention of mother-to-child transmission, and labor and delivery wards)  **Number of items:** 25 items total  **Answer mode:** Rating scale,Dichotomous scale,Free text,Answer mode varies by item | **Reliability:** NA  **Validity**: NA |
| Ahmadi, 2016 [71]  **Year published:** 2016  **Domain:** Stigma or discrimination in healthcare settings  **Measure name:** N/A  https://www.ncbi.nlm.nih.gov/pubmed/27240562 | **Aim:** To develop a precise and concise measure of HIV/AIDS related stigma among health care professionals  **Definition:** N/A  **Stigma subtype addressed:** N/A  **Underlying framework:** Mokken scaling technique used for analysis | **Target population:** People living with HIV/AIDS  **Surveyed participants:** Healthcare students at the Monash University campuses in Malaysia and Australia  **Framework specific to HIV:** Yes | **Scale structure:** 1 scale  **Number of items:** 9 items  **Answer mode:** Rating scale | **Reliability:** Cronbach's alpha 0.89  **Validity**: The scale had intuitive face validity, convergent validity was also established for use with students in healthcare professions |
| Woldetsadik, 2016 [115]  **Year published:** 2016  **Domain:** Stigma or discrimination in healthcare settings  **Measure name:** NA  https://www.ncbi.nlm.nih.gov/pubmed/26487299 | **Aim:** Client and provider measure related to safer conception methods and safer conception counselling in serodiscordant relationships  **Definition:** NA  **Stigma subtype addressed:** NA  **Underlying framework:** NA | **Target population:** Clients in seroconcordant or serodiscordant relationships of people living with HIV  **Surveyed participants:** Adult married or in a committed heterosexual relationship who had intentions to have a child with their partner within the next 24 months and who also were in seroconcordant or serodiscordant relationships were eligible to participate.  **Framework specific to HIV:** Yes | **Scale structure:** Client belief measure: 4 client scales, total of 25 items  Provider belief measure: 6 provider scales, total of 28 items  **Number of items:** 25-items and 28-items from the client and provider surveys  **Answer mode:** Answer mode varies by item | **Reliability:** Client Measures: Cronbach’s a for the 3-item Self-efficacy for using SCM (safer contraception methods) measure was 0.50; the 3-item scales for Motivation to use SCM and Perceived partner’s willingness to use SCM Cronbach’s alphas of 0.88 and 0.85; Cronbach’s alpha for the 3-item for Perceived community stigma was 0.94  Provider Measures: Cronbach’s alpha for the 5-item Provider stigma of childbearing among PLWHIV scale was 0.61, Cronbach’s alpha for the 2-item scale for Interest in providing SCC (safer contraception counseling) to serodiscordant couples was 0.91, 0.68 for the 6-item Interest in providing SCC regarding specific SCM scale, and 0.83 for the 4-item Interest in providing SCC in the context of relational factors scale  **Validity**: Content validity was established during the iterative item development process conducted with six experts; face validity was explored during cognitive debriefing conducted during pilot testing with volunteers; construct validity was evaluated through exploring divergent validity for the client scales, but could not be evaluated in the provider scales due to the lack of a validated comparison measure |
| Aggarwal, 2017 [70]  **Year published:** 2017  **Domain:** Stigma or discrimination in healthcare settings  **Measure name:** NA  https://www.ncbi.nlm.nih.gov/pubmed/28099036 | **Aim:** To understand stigmatizing attitudes toward HIV-positive patients by healthcare students in Mwanza, Tanzania, including those who will be directly treating patients and those who will be indirectly involved through nonclinical roles, such as handling patient specimens and private health information  **Definition:** HIV stigma = negative beliefs, feelings, and attitudes toward people living with HIV that result in discriminatory behavior  **Stigma subtype addressed:** NA  **Underlying framework:** NA | **Target population:** HIV-positive patients  **Surveyed participants:** Healthcare students in Mwanza, Tanzania  **Framework specific to HIV:** Yes | **Scale structure:** 3 sub-domains: personal or cultural beliefs about HIV, HIV education, and clinical interactions with HIV-positive patients  **Number of items:** 18 items  **Answer mode:** Dichotomous scale | **Reliability:** NA  **Validity**: NA |
| Srithanaviboonchai, 2017 [103]  **Year published:** 2017  **Domain:** Stigma or discrimination in healthcare settings  **Measure name:** Brief health staff questionnaire  https://www.ncbi.nlm.nih.gov/pubmed/28284184 | **Aim:** To adapt and refine a standardized global health facility staff stigma and discrimination questionnaire for the context of Thailand  **Definition:** NA  **Stigma subtype addressed:** Enacted, anticipated, internalized, experienced  **Underlying framework:** NA | **Target population:** People living with HIV  **Surveyed participants:** Health facility staff in Thailand; People living with HIV in Thailand  **Framework specific to HIV:** Yes | **Scale structure:** Health facility staff questionnaire contains domains of: key drivers of stigma and manifestations of stigma. Brief PLHIV questionnaire contains 5 domains: health related stigma, disclosure and confidentiality, stigma & discrimination in relation to reproductive health, Experience of stigma among pregnant women  **Number of items:** Health facility staff questionnaire: 14 questions containing 26 items  Brief PLHIV questionnaire: 17 questions with 33 items  **Answer mode:** Answer mode varies by item | **Reliability:** NA  **Validity**: NA |
| Wagner, 2017 [112]  **Year published:** 2017  **Domain:** Stigma or discrimination in healthcare settings  **Measure name:** Sexually Transmitted and Blood-Borne Infections Stigma Scale (STBBI)  https://www.ncbi.nlm.nih.gov/pubmed/29120307 | **Aim:** To describe the adaptation of an HIV-related stigma scale and pilot testing of a new STBBI Stigma Scale and assessing the stigmatizing attitudes and beliefs of health and social service providers  **Definition:** Stigma = dynamic process of devaluation that significantly discredits an individual in the eyes of others  **Stigma subtype addressed:** NA  **Underlying framework:** Earnshaw and Chaudoir’s tripartite framework of stigma | **Target population:** Individuals with sexually transmitted and blood-borne infections  **Surveyed participants:** Health and social service providers in Canada  **Framework specific to HIV:** Broader | **Scale structure:** Each item is posed for four subgroups: HIV, hepatitis C, other viral STBBIs and bacterial STBBIs  **Number of items:** 84 items total, 21 items per subgroup  **Answer mode:** Rating scale | **Reliability:** Internal consistency reliability for each total score and all subscales for each STBBI category were all acceptable to excellent (hepatitis C ranging from Cronbach’s alpha 0.767 to 0.902; HIV 0.809 to 0.906; other viral 0.853 to 0.923; and bacterial 0.852 to 0.915)  **Validity**: Convergent validity was assessed using the AAS Avoidance subscale as a comparison. Scores on the AAS-Avoidance were correlated with total scale scores for the STBBI Stigma categories, indicating convergent validity; inter-factor correlations support the structure of the scale and the uniqueness of the subscales |
| Wouters, 2017 [116]  Wouters, 2016 [145]  **Year published:** 2017  **Domain:** Stigma or discrimination in healthcare settings  **Measure name:** Respondents' Stigma Towards HIV (HIVRES); Colleagues’ Stigmatizing Attitudes, Perceptions and Behaviors towards HIV (HIVOES)  https://www.ncbi.nlm.nih.gov/pubmed/29025481 | **Aim:** To test scales measuring different aspects of stigma  **Definition:** NA  **Stigma subtype addressed:** External  **Underlying framework:** Wouters et al., 2016 | **Target population:** People living with HIV or tuberculosis  **Surveyed participants:** Healthcare workers including patient staff and support staff in South Africa  **Framework specific to HIV:** Broader | **Scale structure:** 2 scales for HIV: respondent’s external stigma (HIVRES) and others’ external stigma (HIVOES), and the same two for tuberculosis  **Number of items:** 8 items total, 4 items per scale  **Answer mode:** Rating scale | **Reliability:** Both HIV scales displayed good reliability (Cronbach’s alpha 0. 7)  **Validity**: All 4 scales displayed adequate internal construct validity; all scales were metric-invariant, the OES scales were scalar-invariant across patient and support staff groups; confirmatory factor analysis demonstrated that the scales measuring external stigma towards HIV (1) and TB (2) among colleagues and the external stigma towards HIV (3) and (4) among respondents displayed acceptable internal construct validity; structural equation modeling supports the external construct validity of the scales |
| Xie, 2019 [117]  **Year published:** 2019  **Domain:** Stigma or discrimination in healthcare settings  **Measure name:** Health Care Provider HIV/AIDS Stigma Scale (HPASS) Adaptation  https://pubmed.ncbi.nlm.nih.gov/30306438/ | **Aim:** Cross-cultural validation of the Health Care Provider HIV/AIDS Stigma Scale  **Definition:** NA  **Stigma subtype addressed:** stigmatization by health providers  **Underlying framework:** Stigma framework by Earnshaw & Chardoier | **Target population:** People living with HIV/AIDS  **Surveyed participants:** Medical staff  **Framework specific to HIV:** Yes | **Scale structure:** 3 scales: discrimination, prejudice, stereotype  **Number of items:** 16 items  **Answer mode:** Rating scale | **Reliability:** Cronbach’s ?alpha of the scale was 0.88, and for the factors 0.89 (discrimination), 0.86 (prejudice) and 0.74 (stereotype); test-retest ICC 0.87, ICCs of items were between 0.20 and 0.75,  **Validity**: Content validity: Scale-Content Validity Index of Universal Agreement 0.44, Scale Content Validity Index Average 0.88, Item Content Validity Indexes ranged 0.33-1.00; 3 factors explained 59.61% of variance; correlations between factors ranged from 0.342 to 0.602 |
| UNAIDS, 2020 [106]  UNAIDS, WHO, 2018 [143]  **Year published:** 2020  **Domain:** Stigma or discrimination in healthcare settings,Stigma or discrimination in law  **Measure name:** National Commitments and Policy Instrument  https://www.unaids.org/sites/default/files/media_asset/global-aids-monitoring_en.pdf | **Aim:** NCPI is an integral component of Global AIDS Monitoring that aims to measure progress in developing and implementing policies, strategies and laws related to the HIV response  **Definition:** NA  **Stigma subtype addressed:** NA  **Underlying framework:** NA | **Target population:** People living with HIV  **Surveyed participants:** People living with HIV  **Framework specific to HIV:** Yes | **Scale structure:** 2 parts (Part A for national authorities, Part B for nongovernmental partners)  **Number of items:** 10 target areas  **Answer mode:** Unclear | **Reliability:** NA  **Validity**: NA |
